# Path Integrals for Electronic Densities, Reactivity Indices, and Localization Functions in Quantum Systems

**DOI:** 10.3390/ijms10114816

**Published:** 2009-11-10

**Authors:** Mihai V. Putz

**Affiliations:** Laboratory of Computational and Structural Physical Chemistry, Chemistry Department, West University of Timişoara, Pestalozzi Street No.16, Timişoara, RO-300115, Romania; E-Mails: mvputz@cbg.uvt.ro; mv_putz@yahoo.com; Website: www.mvputz.iqstorm.ro

**Keywords:** density matrix and functionals, Feynman integral, partition function, electronegativity, chemical action and hardness, Fokker-Planck equation, electronic localization

## Abstract

The density matrix theory, the ancestor of density functional theory, provides the immediate framework for Path Integral (PI) development, allowing the canonical density be extended for the many-electronic systems through the density functional closure relationship. Yet, the use of path integral formalism for electronic density prescription presents several advantages: assures the inner quantum mechanical description of the system by parameterized paths; averages the quantum fluctuations; behaves as the propagator for time-space evolution of quantum information; resembles Schrödinger equation; allows quantum statistical description of the system through partition function computing. In this framework, four levels of path integral formalism were presented: the Feynman quantum mechanical, the semiclassical, the Feynman-Kleinert effective classical, and the Fokker-Planck non-equilibrium ones. In each case the density matrix or/and the canonical density were rigorously defined and presented. The practical specializations for quantum free and harmonic motions, for statistical high and low temperature limits, the smearing justification for the Bohr’s quantum stability postulate with the paradigmatic Hydrogen atomic excursion, along the quantum chemical calculation of semiclassical electronegativity and hardness, of chemical action and Mulliken electronegativity, as well as by the Markovian generalizations of Becke-Edgecombe electronic focalization functions – all advocate for the reliability of assuming PI formalism of quantum mechanics as a versatile one, suited for analytically and/or computationally modeling of a variety of fundamental physical and chemical reactivity concepts characterizing the (density driving) many-electronic systems.

## 1. Introduction

In modern conceptual and computational chemistry the Density Functional Theory (DFT) [1–12] plays the central role since its capabilities in providing both structural and reactivity information about the atoms and of their interaction in molecules and nanostructures [13–20]. Yet, the main vehicle stays the electronic density, an observable quantity, which intimately relates with the more abstract quantum mechanical concept of wave function through the basic relationship [3]

(1) $$\rho (x_{1})=N{\int \left|\Psi \left({\left\{r_{i}\right\}}_{i=\bar{1,N}}\right)\right|}^{2}ds_{1}dr_{2}\ldots dr_{N}$$

written for a collection of *N*-many-electronic system with space-spin coordinates *dr_i_* = *dx_i_* *ds_i_*, in terms of space and spin coordinates, {*x_i_*}*_i_*_= 1*,N*_ and {*s_i_*}_*i*= 1,*N*_, respectively, in constructing the basic functional (integral) for the charge conservation

(2) $$N\left[\rho \right]=\int \rho (x_{1})dx_{1}$$

Besides the sub-script index, it is worth noting that with Equations (1) and (2) either the ground state or the valence state(s) of a many body system may be computed by applying the variational principle upon the total or valence density functional energy [7]

(3) $$E\left[\rho \right]=F_{HK}\left[\rho \right]+C_{A}\left[\rho \right]$$

which in terms of the so called Hohenberg-Kohn density functional [1]

(4) $$F_{HK}[\rho ]\;=\;T[\rho ]\;+\;V_{ee}[\rho ]$$

that sums the electronic kinetic *T*[*ρ*] and electronic repulsion *V_ee_*[*ρ*], and of the so called *chemical action* term [7–10]

(5) $$C_{A}\left[\rho \right]=\int \rho (x)V(x)dx$$

accounts in principle for all observable effects an electronic structure may manifest as the chemical reactivity.

Yet, although accessible experimentally [21], for a deeper comprehension of the physical-chemical phenomenology of bonding the electronic density should be “visualized” also within an analytical framework. In this regard the already consecrated attempt of density matrix made history over a half century by employing the so called *ensemble (statistical) density operator* [22–26]

(6) $$\hat{\rho }=\sum_{k}w_{k}|\phi_{k}\rangle \langle \phi_{k}|$$

which by means of its first order reduced one *ρ̂*^(1)^ (*x*_1_; *x*_1_) provides by integration the total number of electrons in the same way as the observable density does [27–31]

(7) $$\int \rho^{\left(1\right)}\left(x_{1};x_{1}\right)dx_{1}=N$$

that is formally equivalent with the “trace” operation on the bilocal density matrix *ρ̂*^(1)^ (*x*′_1_; *x*_1_)

(8) $$\text{Tr}\left[{\hat{\rho }}^{\left(1\right)}\left({x^{\prime }}_{1};x_{1}\right)\right]=N$$

From this perspective it is clear that having in hand a reliable method to express, for various applied potentials *V* (*x*) the bilocal density matrix, from here on called simple as *density matrix*, yields in fact the electronic density itself. Fortunately the quantum mechanically formalism had advanced a rigorous way of expressing the density matrix since recognizing it as a specialization of the more general *time evolution quantum amplitude*, a.k.a. *the quantum propagator* or the *Green function* [32–36]

(9) $$\left(x_{b}t_{b};x_{a}t_{a}\right)=\langle x_{b}|\text{exp}\left(-\beta \hat{H}\right)|x_{a}\rangle$$

Moreover, within the thermal-temporal (quantum mechanical-to quantum statistical) Wick transformation

(10) $$t_{b}-t_{a}=-i\hbar \beta =-\frac{i\hbar }{k_{B}T}$$

with *k_B_* the Boltzmann constant, *T* the sample temperature, and *ħ* - the Planck constant, there is immediate to introduce the partition function of a system as the analytically time continued integral

(11) $$Z=Tr\left[\text{exp}\left(-\beta \hat{H}\right)\right]=\int_{-\infty }^{+\infty }dx\langle x|\text{exp}\left(-\beta \hat{H}\right)|x\rangle =\int_{-\infty }^{+\infty }dx\left(xt_{b};xt_{a}\right)$$

to release the uni-particle density

(12) $$\rho (x)=\frac{N}{Z}\langle x|\text{exp}\left(-\beta \hat{H}\right)|x\rangle$$

while fulfilling the normalization constraint given by Equation (2).

The competition between the variation of density functional of energy (3) and the density itself (12), *i.e.*, the ratio between the global and local influences on a many-electronic system (equilibrium, aromaticity, etc.) is quantified by the modern electronegativity index and by its chemical hardness companion [3,5–12], given respectively as

(13a) $$\chi =-{\left(\frac{\delta E[\rho ]}{\delta \rho }\right)}_{V(x)=ct.}$$

(13b) $$\eta =-{\left(\frac{\delta \chi \left[\rho \right]}{\delta \rho }\right)}_{V(x)=ct.}$$

which may assume various analytical realizations within density functional theory [7,9], while playing the crucial roles in driving chemical bonding and reactivity [12,20]; this may be also immediately seen from Equations (13) when electronegativity is further identified with the negative of the chemical potential of a given system (*χ* =−*μ*) [6]: if electronegativity is assimilated with the chemical potential, the chemical hardness – as its derivative – corresponds with the chemical force; thus, they together constitute the minimal necessary set of indices to consistently model the complete scenario of chemical reactivity, from encountering adducts to the stabilized products [9]. For these reasons they will be systematically presented in this review as application for different levels of path integral approximations in either matrix density or density computations.

However, it is worth comment that the above factorization only apparently assumes the many-particle system without internal interaction (exchange and correlation), while all these effects are to be incorporated in the way the bare applied potential is replaced with an effective one or by performing a variational (upon the) perturbation procedure for optimizing the (bilocal) density matrix

(14) $$\begin{array}{c} \rho^{\left(1\right)}\left(x_{b};x_{a}\right)=\int_{x_{a}}^{x_{b}}Dx(\tau )\text{exp}\left\{-\frac{1}{\hbar }\int_{0}^{\beta }d\tau \hat{H}\left(x(\tau ),\dot{x}(\tau ),\tau \right)\right\}\\ =\int_{x_{a}}^{x_{b}}Dx(\tau )\text{exp}\left\{-\frac{1}{\hbar }\int_{0}^{\beta }d\tau \left[\frac{m{\dot{x}}^{2}(\tau )}{2}+V\left(x(\tau )\right)\right]\right\} \end{array}$$

in accordance with above recipe. Equation (14) is nevertheless nothing else than the so called *path integral* of the density matrix or of the time evolution quantum amplitude, while ∫ *Dx*(*τ*) is a complex symbol of integration over (parameterized, quantum) paths that when unfolded recovers the normal shape of integration, *i.e.*, with separated variables of integration, *e.g.*, Equations (117) or (316) and Refs. [37–39]. The way in which this path integral is computed for various working potentials will generate the analytical solution of the quantum amplitude, and implicitly to the density matrix, from where the electronic density is immediately found by the identity *ρ*(*x_a_*) = *ρ*^(1)^ (*x_b_* = *x_a_*; *x_a_*). Then, having the electronic density any known or approximated density functional may be evaluated and employed in describing the chemical structure and reactivity in an analytical manner, while allowing better conceptual understanding of the obtained models and predictions. With these, the motivation behind the present project becomes clear: computing the electronic density relays in fact on evaluating the associate path integral for a given potential; moreover, the many-body effects are to be resumed in rewriting the applied potential into an effective one (one way) or to advance the so called variational perturbation methodology in order the inter-particle effects be accommodated [39–48], being this latter approach left for further communications.

Consequently, the review unfolds on bigger scale the ideas here presented: it starts with the basic properties of the density matrix and showing how the path integral concept arises naturally in this framework. Then, a more formal introduction of path integral methodology is presented in the spirit of Richard Feynman, its main promoter; and the use of path integrals is exemplified in computing semi-classical time evolution amplitudes with application on atomic electronegativity and chemical hardness reactivity indices. The simplified many-body approach is then given through exposing the Feynman-Kleinert algorithm for effective potentials, with application on computing atomic Mulliken electronegativities, while the non-equilibrium Fokker-Planck approach is exposed and applied in the context of Markovian stochastic motion within the anharmonic potential and then extended to modeling the electronic localization through computing several Markovian electronic functions while comparing them with the circulating Becke-Edgecombe one [49,50]. This way, a fruitful step is hopefully made towards unifying the physical-chemical principles of electronic structure and reactivity on a meaningful quantum basis.

## 2. From Density Matrix to Path Integral

### 2.1. On Mono-, Many-, and Reduced- Electronic Density Matrices

Given a spectral representation {|*n〉*}*_n_*_∈**N**_ for a set of quantum mono-electronic states

(15) $$|\phi_{k}\rangle =\sum_{n}c_{kn}|n\rangle$$

one may employ its closure relation:

(16) $$\hat{1}=\sum_{n}|n\rangle \langle n|$$

to generally express the average of an observable (*i.e.*, the operator *Â*) on a selected state as

(17) $${\langle \hat{A}\rangle }_{k}=\frac{\langle \phi_{k}\left|\hat{A}\right|\phi_{k}\rangle }{\langle \phi_{k}|\phi_{k}\rangle }=\frac{\sum_{n,n^{\prime }}\langle \phi_{k}|n^{\prime }\rangle \langle n^{\prime }|\hat{A}|n\rangle \langle n|\phi_{k}\rangle }{\sum_{n}\langle \phi_{k}|n\rangle \langle n|\phi_{k}\rangle }=\frac{\sum_{n,n^{\prime }}c_{kn}c_{kn^{\prime }}^{*}\langle n^{\prime }|\hat{A}|n\rangle }{\sum_{n}{\left|c_{kn}\right|}^{2}}$$

while for the observable average over the entire sample the individual weight *w_k_* should be counted to provide the statistical result

(18) $$\langle \hat{A}\rangle =\frac{\sum_{k}w_{k}{\langle \hat{A}\rangle }_{k}}{\sum_{k}w_{k}}$$

Similarly, when rewriting the global average as

(19) $$\langle \hat{A}\rangle =\frac{\sum_{n,n^{\prime }}\langle n|\hat{\rho }|n^{\prime }\rangle \langle n^{\prime }|\hat{A}|n\rangle }{\sum_{k}w_{k}}$$

we recognize the *density matrix elements*

(20) $$\langle n\left|\hat{\rho }\right|n^{\prime }\rangle =\sum_{k}w_{k}\frac{c_{kn}c_{kn^{\prime }}^{*}}{\sum_{n}{\left|c_{kn}\right|}^{2}}$$

which provides the *density operator*

(21) $$\hat{\rho }=\sum_{n,n^{\prime }}|n\rangle \langle n|\hat{\rho }|n^{\prime }\rangle \langle n^{\prime }|=\sum_{k}\frac{w_{k}}{\sum_{n}{\left|c_{kn}\right|}^{2}}\left(\sum_{n}c_{kn}|n\rangle \right)\left(\sum_{n^{\prime }}\langle n^{\prime }|c_{kn^{\prime }}^{*}\right)=\sum_{k}\frac{w_{k}}{\sum_{n}{\left|c_{kn}\right|}^{2}}|\phi_{k}\rangle \langle \phi_{k}|$$

with the sum of diagonal matrix elements (yielding the “trace” function)

(22) $$\text{Tr}\hat{\rho }=\sum_{n}\langle n|\hat{\rho }|n\rangle =\sum_{k}w_{k}\frac{\sum_{n}{\left|c_{kn}\right|}^{2}}{\sum_{n}{\left|c_{kn}\right|}^{2}}=\sum_{k}w_{k}$$

while the searched operatorial average now becomes

(23) $$\langle \hat{A}\rangle =\frac{\sum_{n,n^{\prime }}\langle n|\hat{\rho }|n^{\prime }\rangle \langle n^{\prime }|\hat{A}|n\rangle }{\sum_{k}w_{k}}=\frac{\sum_{n}\langle n|\hat{\rho }\hat{A}|n\rangle }{\sum_{n}\langle n|\hat{\rho }|n\rangle }=\frac{\text{Tr}\left(\hat{\rho }\hat{A}\right)}{\text{Tr}\hat{\rho }}$$

Note that through the above deductions the double (independent) averages technique was adopted exploiting therefore the associate sums inter-conversions to produce the simplified results [51–53]. Yet, this technique is equivalent with quantum mechanically factorization of the entire Hilbert space into sub-spaces or, at the limit, into the subspace of interest (that selected to be measured, for instance) and the rest of the space, being thus this approach equivalent with a *system-bath* sample; this is useful for better understanding the stochastic phenomena – to be latter exposed – that underlay to open quantum systems. Therefore, such mechanism may be considered as belonging to the physical foundations for the chemical reactivity.

Next, in the case the concerned quantum states are *eigen-states*, they fulfill the normalization constraint

(24) $$\delta_{kk^{\prime }}=\langle \phi_{k}|\phi_{k^{\prime }}\rangle =\sum_{n}c_{kn}^{*}c_{k^{\prime }n}\Rightarrow \sum_{n}{\left|c_{kn}\right|}^{2}=1$$

on which base the above density operator now reads with the same form as presented din Introduction, Equation (6), from where there appears that the eigen-equation for it looks like

(25) $$\hat{\rho }|\phi_{k}\rangle =\sum_{k^{\prime }}w_{k^{\prime }}|\phi_{k^{\prime }}\rangle \underset{\delta_{k^{\prime }k}}{\underset{︸}{\langle \phi_{k^{\prime }}|\phi_{k}\rangle }}=w_{k}|\phi_{k}\rangle$$

giving with the eigen-values (as the diagonal elements) as

(26) $$\langle \phi_{k}|\hat{\rho }|\phi_{k}\rangle =w_{k}$$

as the observed values of the averaged density operator. Since they are weights of probability they have to naturally fulfill the closure probability relationship over the entire sample

(27) $$\sum_{k}w_{k}=1$$

from where follows the “normalization of density operator” through its above Trace property of Equation (22)

(28) $$\text{Tr}\hat{\rho }=1$$

Moreover, in these eigen-conditions, the operatorial average further reads from Equation (23)

(29) $$\langle \hat{A}\rangle =\text{Tr}\left(\hat{\rho }\hat{A}\right)$$

Now, there appears with better clarity the major role the density operator plays in quantum measurements, since it convolutes with a given operator to produce its (averaged) measured value on the prepared eigen-states. Nevertheless, when the so called *pure states* are employed or prepared, the preceding distinction between the subsystem and system vanishes, and the density operator takes the pure quantum mechanical form of an elementary projector

(30) $$\hat{\rho }=|\phi \rangle \langle \phi |\equiv \hat{\Lambda }$$

This is a very useful expression for considering it associated with the mono-density operators when many-fermionic systems are treated, although a similar procedure applies for mixed (sample) states as well. It is immediate to see that for *N* formally independent partitions the Hilbert space corresponding to the *N*-mono-particle densities on pure states, we have through Equations (22), (27), (28) and (30)

(31) $${\hat{\Lambda }}_{i}=|\phi_{i}\rangle \langle \phi_{i}|,\text{Tr}{\hat{\Lambda }}_{i}=1,i=\bar{1,N}$$

producing the total operator – projector constructed by the sum

(32) $${\hat{\Lambda }}_{N}=\sum_{i=1}^{N}{\hat{\Lambda }}_{i}$$

while correctly normalized to the total number of particles

(33) $$\text{Tr}{\hat{\Lambda }}_{N}=\text{Tr}\left(\sum_{i=1}^{N}{\hat{\Lambda }}_{i}\right)=\sum_{i=1}^{N}\underset{1}{\underset{︸}{\text{Tr}{\hat{\Lambda }}_{i}}}=N$$

Yet, the anti-symmetric restriction the *N*-fermionic state may be accounted from the mono-electronic states through considering Slater permutated (*P_α_*) products [8,35]

(34) $$|\Phi_{N}\rangle =\frac{1}{\sqrt{N!}}\sum_{P_{\alpha }}{\left(-1\right)}^{P_{\alpha }}P_{\alpha }\left[\prod_{i=1}^{N}|\phi_{i}\rangle \right]$$

for constructing the *N*-electronic density operator

(35) $${\hat{\rho }}^{\left(N\right)}=|\Phi_{N}\rangle \langle \Phi_{N}|$$

with which help the *N* × *N* density matrix writes as (in coordinate representation)

(36) $$\begin{array}{c} \rho^{\left(N\right)}({x^{\prime }}_{1}\;\ldots {x^{\prime }}_{N}\;;\;x_{1}\;\ldots x_{N})\;=\;〈{x^{\prime }}_{1}\;\ldots {x^{\prime }}_{N}\;\left|{\hat{\rho }}^{\left(N\right)}\right|\;x_{1}\;\ldots x_{N}〉\;=\;〈{x^{\prime }}_{1}\;\ldots {x^{\prime }}_{N}\;\left|\Phi_{N}〉〈\Phi_{N}\right|x_{1}\;\ldots x_{N}〉\\ =\;\frac{1}{N!}\sum_{{P^{\prime }}_{\alpha }}{\left(-1\right)}^{{P^{\prime }}_{\alpha }}{P^{\prime }}_{\alpha }\left[\prod_{i=1}^{N}〈{x^{\prime }}_{i}\;|\;\phi_{i}〉\right]\sum_{P_{\alpha }}{\left(-1\right)}^{P_{\alpha }}P_{\alpha }\left[\prod_{i=1}^{N}〈\phi_{i}\;|\;x_{i}〉\right]\\ =\;\frac{1}{N!}\sum_{{P^{\prime }}_{\alpha }}{\left(-1\right)}^{{P^{\prime }}_{\alpha }}{P^{\prime }}_{\alpha }\left[\prod_{i=1}^{N}\phi_{i}({x^{\prime }}_{i})\right]\sum_{P_{\alpha }}{\left(-1\right)}^{P_{a}}P_{\alpha }\left[\prod_{i=1}^{N}\phi_{i}^{*}(x_{i})\right] \end{array}$$

However, in practice, due to the fact the multi-particle operators associate with number of systemic properties less than the total number of particle, say of order *p* < *N*, worth working with the *p-order reduced density matrix* introduced as

(37) $$\rho^{\left(p\right)}({x^{\prime }}_{1}\;\ldots {x^{\prime }}_{p}\;;\;x_{1}\;\ldots x_{p})\;=\;\left(\begin{array}{c} N\\ p \end{array}\right)\int \Phi_{N}^{*}(x_{1}\;\ldots x_{N})\Phi_{N}({x^{\prime }}_{1}\;\ldots {x^{\prime }}_{N})\prod_{j=p+1}^{N}dx_{j}$$

with the following features [27].

○ Normalization
  (38) $$\int \rho^{\left(p\right)}\left({x^{\prime }}_{1}\;\ldots {x^{\prime }}_{p}\;;\;x_{1}\;\ldots x_{p}\right)\prod_{j=1}^{p}dx_{j}\;=\;\left(\begin{array}{c} N\\ p \end{array}\right)$$
○ Recursion
  (39) $$\int \rho^{\left(p\right)}\left({x^{\prime }}_{1}\;\ldots {x^{\prime }}_{p}\;;\;x_{1}\;\ldots x_{p}\right)dx_{p}\;=\;\frac{N\;+\;1\;-\;p}{p}\rho^{\left(p-1\right)}\left({x^{\prime }}_{1}\;\ldots {x^{\prime }}_{p-1}\;;\;x_{1}\;\ldots x_{p-1}\right)$$
○ First order Löwdin reduction
  (40) $$\begin{array}{c} \rho^{\left(p\right)}\left({x^{\prime }}_{1}\;\ldots {x^{\prime }}_{p}\;;\;x_{1}\;\ldots x_{p}\right)\;=\;\frac{1}{p!}\text{det}\left[\rho^{\left(1\right)}\left({x^{\prime }}_{k}\;;\;x_{k}\right)\right]\\ =\;\frac{1}{p!}\left|\begin{array}{cccc} \rho^{\left(1\right)}\left({x^{\prime }}_{1}\;;\;x_{1}\right) & \rho^{\left(1\right)}\left({x^{\prime }}_{1}\;;\;x_{2}\right) & \cdots & \rho^{\left(1\right)}\left({x^{\prime }}_{1}\;;\;x_{p}\right)\\ \rho^{\left(1\right)}\left({x^{\prime }}_{2}\;;\;x_{1}\right) & \rho^{\left(1\right)}\left({x^{\prime }}_{2}\;;\;x_{2}\right) & \cdots & \rho^{\left(1\right)}\left({x^{\prime }}_{2}\;;\;x_{p}\right)\\ \vdots & \vdots & & \vdots \\ \rho^{\left(1\right)}\left({x^{\prime }}_{p}\;;\;x_{1}\right) & \rho^{\left(1\right)}\left({x^{\prime }}_{p}\;;\;x_{2}\right) & \cdots & \rho^{\left(1\right)}\left({x^{\prime }}_{p}\;;\;x_{p}\right) \end{array}\right| \end{array}$$

where the first order density matrix casts as abstracted from general definition

(41) $$\rho^{\left(1\right)}\left({x^{\prime }}_{1}\;;\;x_{1}\right)\;=\;N\int \Phi_{N}^{*}\left(x_{1}\;\ldots x_{N}\right)\Phi_{N}\left({x^{\prime }}_{1}\;\ldots {x^{\prime }}_{N}\right)\prod_{j=2}^{N}dx_{j}$$

With these concepts it is worth noting the major importance that the first order density plays in computing the higher order reduced density matrices that in turn enter the operatorial averages, for instance

(42) $$〈\hat{A}〉\;=\;\sum_{p=1}^{N}{\text{Tr}}^{\left(p\right)}\left[\hat{A}\left(x_{1}\;\ldots x_{p}\right){\hat{\rho }}^{\left(p\right)}\right]$$

A special reference may be made in regard of the free-relativist treatment of many-electronic atoms, ions, bi- or poly- atomic molecules governed by the working Hamiltonian

(43) $$\hat{H}\;=\;e^{2}\;\sum_{G<H}\frac{Z_{G}Z_{H}}{R_{GH}}\;+\;\sum_{i=1}^{N}\frac{p_{i}^{2}}{2m}\;-\;e^{2}\sum_{i,G}\frac{Z_{G}}{r_{iG}}\;+\;e^{2}\sum_{i<j}\frac{1}{r_{ij}}$$

whose terms are represented the inter-nuclear repulsion (only for molecules), free electronic motion, electron-nuclei Coulombic attraction, and inter-electronic Coulombian repulsion, respectively. For such Hamiltonian the average value is computed through considering electronic density of the first or second order only there where the electronic influence is present, while the degree of matrix density is fixed by the type of electronic interaction

(44) $$\begin{array}{c} 〈\hat{H}〉\;=\;e^{2}\sum_{G<H}\frac{Z_{G}Z_{H}}{R_{GH}}\\ +\frac{1}{2m}\;{\int p_{1}^{2}\rho^{\left(1\right)}\left({x^{\prime }}_{1}\;;\;x_{1}\right)|}_{{x^{\prime }}_{1}\;=\;x_{1}}dx_{1}\;-\;e^{2}\sum_{G}Z_{G}{\int \frac{\rho^{\left(1\right)}\left({x^{\prime }}_{1}\;;\;x_{1}\right)}{r_{1G}}|}_{{x^{\prime }}_{1}\;=\;x_{1}}dx_{1}\\ +\;e^{2}\int \int \frac{\rho^{\left(2\right)}\left({x^{\prime }}_{1},\;{x^{\prime }}_{2}\;;\;x_{1},\;x_{2}\right)}{r_{12}}|{}_{\begin{array}{c} {x^{\prime }}_{1}\;=\;x_{1}\\ {x^{\prime }}_{2}\;=\;x_{2} \end{array}}dx_{1}dx_{2} \end{array}$$

It is obvious that although the second order reduced matrix has appeared, its general form

(45) $$\rho^{\left(2\right)}\left({x^{\prime }}_{1}\;,\;{x^{\prime }}_{2}\;;\;x_{1},\;x_{2}\right)\;=\;\left(\begin{array}{c} N\\ 2 \end{array}\right)\int \Phi_{N}^{*}\left(x_{1}\;\ldots x_{N}\right)\Phi_{N}\left({x^{\prime }}_{1}\;\ldots {x^{\prime }}_{N}\right)\prod_{j=3}^{N}dx_{j}$$

may be further reduced to the first one through the above determinant rule, see Equation (40)

(46) $$\begin{array}{c} \rho^{\left(2\right)}\left({x^{\prime }}_{1}\;,\;{x^{\prime }}_{2}\;;\;x_{1},\;x_{2}\right)\;=\;\frac{1}{2!}\left|\begin{array}{cc} \rho^{\left(1\right)}\left({x^{\prime }}_{1}\;;\;x_{1}\right) & \rho^{\left(1\right)}\left({x^{\prime }}_{1}\;;\;x_{2}\right)\\ \rho^{\left(1\right)}\left({x^{\prime }}_{2}\;;\;x_{1}\right) & \rho^{\left(1\right)}\left({x^{\prime }}_{2}\;;\;x_{2}\right) \end{array}\right|\\ =\;\frac{1}{2}\left[\rho^{\left(1\right)}\left({x^{\prime }}_{1}\;;\;x_{1}\right)\rho^{\left(1\right)}\left({x^{\prime }}_{2}\;;\;x_{2}\right)\;-\;\rho^{\left(1\right)}\left({x^{\prime }}_{1}\;;\;x_{2}\right)\rho^{\left(1\right)}\left({x^{\prime }}_{2}\;;\;x_{1}\right)\right] \end{array}$$

this way emphasizing on the importance of the first order reduced matrix knowledge.

The astonishing physical meaning behind this formalism relays in the fact that any multi-particle interaction (two-particle interaction included) may be reduced to the single particle behavior; in other terms, vice-versa, the appropriate perturbation (including strong-coupling) of the single particle evolution carries the equivalent information as that characterizing the whole many-body system.

In fact, the power of the density matrix formalism resides in reducing a many-body problem to the single particle density matrix, abstracted from the single Slater determinant of Equation (36) known as the *Fock-Dirac matrix*

(47) $$\rho_{FD}^{\left(1\right)}\left({x^{\prime }}_{1}\;;\;x_{1}\right)\;=\;\sum_{i=1}^{N}\phi_{i}^{*}\left(x_{1}\right)\phi_{i}\left({x^{\prime }}_{1}\right)$$

that along the associate operator

(48) $${\hat{\rho }}_{FD}^{\left(1\right)}\;=\;\sum_{i=1}^{N}\left|\phi_{i}〉〈\phi_{i}\right|$$

considerably simplifies the quantum problem to be solved. Let’s illustrate this by firstly quoting that Fock-Dirac density operator of Equation (48) has two fundamental properties, namely:

○ The idempotency
  (49) $${\hat{\rho }}_{FD}^{\left(1\right)}{\hat{\rho }}_{FD}^{\left(1\right)}\;=\;\sum_{i,\;j=1}^{N}|\phi_{i}〉\underset{\delta_{ij}}{\underset{︸}{〈\phi_{i}|\phi_{j}〉}}〈\phi_{j}|\;=\;\sum_{i=1}^{N}\left|\phi_{i}〉〈\phi_{i}\right|\;=\;{\hat{\rho }}_{FD}^{\left(1\right)}$$
○ The normal additivity, see Equations (33)
  (50) $$\text{Tr}{\hat{\rho }}_{FD}^{\left(1\right)}\;=\;\text{Tr}\left(\sum_{i=1}^{N}\left|\phi_{i}〉〈\phi_{i}\right|\right)\;=\;\text{Tr}\left(\sum_{i=1}^{N}{\hat{\Lambda }}_{i}\right)\;=\;N$$

while having the corresponding coordinate integral representations:

○ Kernel multiplicity
  (51) $$\int \rho_{FD}^{\left(1\right)}\left({x^{\prime }}_{1}\;;\;{x^{\prime \prime }}_{1}\right)\rho_{FD}^{\left(1\right)}\left({x^{\prime \prime }}_{1}\;;x_{1}\right)d{x^{\prime \prime }}_{1}\;=\;\rho_{FD}^{\left(1\right)}\left({x^{\prime }}_{1}\;;\;x_{1}\right)$$
○ Many-body normalization
  (52) $$\int \rho_{FD}^{\left(1\right)}\left(x_{1}\;;\;x_{1}\right)dx_{1}\;=\;N$$

Remarkably, the last two identities may serve as the constraints when minimizing the above Hamiltonian average, here appropriately rewritten employing Equations (44) and (46), and where all external applied potential, were resumed under generic *V* (*x*_1_) quantity producing the actual so called *Hartree-Fock trial density matrix energy functional*

(53) $$\begin{array}{c} E_{HF}\left[\rho_{FD}^{\left(1\right)}\right]\;=\;\int \left[-\frac{\hbar^{2}}{2m}\nabla_{1}^{2}\;+\;V(x_{1})\right]\rho_{FD}^{\left(1\right)}{\left({x^{\prime }}_{1}\;;\;x_{1}\right)|}_{{x^{\prime }}_{1}\;=\;x_{1}}dx_{1}\\ +\frac{e^{2}}{2}\int \int \frac{1}{r_{12}}\left[\rho_{FD}^{\left(1\right)}(x_{1}\;;\;x_{1})\rho_{FD}^{\left(1\right)}(x_{2}\;;\;x_{2})\;-\;\rho_{FD}^{\left(1\right)}(x_{1}\;;\;x_{2})\rho_{FD}^{\left(1\right)}(x_{2}\;;\;x_{1})\right]dx_{1}dx_{2} \end{array}$$

obeying the (Lagrange) variational principle

(54) $$\delta \left\{\begin{array}{l} E_{HF}\left[\rho_{FD}^{\left(1\right)}\right]\\ -\;\int \int \alpha \left({x^{\prime }}_{1}\;;\;x_{1}\right)\left[\int \rho_{FD}^{\left(1\right)}\left({x^{\prime }}_{1}\;;\;{x^{\prime \prime }}_{1}\right)\rho_{FD}^{\left(1\right)}\left({x^{\prime \prime }}_{1}\;;\;x_{1}\right)d{x^{\prime \prime }}_{1}\;-\;\rho_{FD}^{\left(1\right)}\left({x^{\prime }}_{1}\;;\;x_{1}\right)\right]d{x^{\prime }}_{1}dx_{1}\\ -\;\beta \left[\int \int \delta \left({x^{\prime }}_{1}\;-\;x_{1}\right)\rho_{FD}^{\left(1\right)}\left({x^{\prime }}_{1}\;;\;x_{1}\right)d{x^{\prime }}_{1}dx_{1}\;-\;N\right] \end{array}\right\}\;=\;0$$

Performing the functional derivative respecting the Fock-Dirac electron density in (54) one gets the equivalent expression

(55) $$\frac{\delta E_{HF}\left[\rho_{FD}^{\left(1\right)}\right]}{\delta \rho_{FD}^{\left(1\right)}\left({x^{\prime }}_{1}\;;\;x_{1}\right)}\;-\;\int \rho_{FD}^{\left(1\right)}\left({x^{\prime }}_{1}\;;\;\bar{x}\right)\alpha \left(\bar{x};\;x_{1}\right)d\bar{x}\;-\;\int \alpha \left({x^{\prime }}_{1}\;;\;\bar{\bar{x}}\right)\rho_{FD}^{\left(1\right)}\left(\bar{\bar{x}};\;x_{1}\right)d\bar{\bar{x}}\;+\;\alpha \left({x^{\prime }}_{1}\;;\;x_{1}\right)\;-\;\beta \delta \left({x^{\prime }}_{1}\;-\;x_{1}\right)\;=\;0$$

which eventually transcribes at the operatorial level as

(56) $$\hat{F}\;-\;{\hat{\rho }}_{FD}^{\left(1\right)}\hat{\alpha }\;-\;\hat{\alpha }{\hat{\rho }}_{FD}^{\left(1\right)}\;+\;\hat{\alpha }\;-\;\beta {\hat{1}}_{\delta }\;=\;0$$

with ${\hat{1}}_{\delta }$ staying for the operator of the delta-Dirac matrix *δ*(*x*′_1_ – *x*_1_), with *F̂* the Fock corresponding to the coordinate matrix representation [3]

(57) $$\begin{array}{c} F\left({x^{\prime }}_{1}\;;\;x_{1}\right)\;=\;\frac{\delta E_{HF}\left[\rho_{FD}^{\left(1\right)}\right]}{\delta \rho_{FD}^{\left(1\right)}\left({x^{\prime }}_{1}\;;\;x_{1}\right)}\\ =\;\left[-\;\frac{\hbar^{2}}{2m}\nabla_{1}^{2}\;+\;V(x_{1})\right]\delta \left({x^{\prime }}_{1}\;-\;x_{1}\right)+\underset{\text{EXCHANGE}\;\text{CONTRIBUTION}}{\underset{︸}{\delta \left({x^{\prime }}_{1}\;-\;x_{1}\right)e^{2}\;\int \frac{1}{r_{12}}\rho_{FD}^{\left(1\right)}\left(x_{2}\;;\;x_{2}\right)dx_{2}\;-\;\frac{e^{2}}{r_{1^{\prime }1}}\rho_{FD}^{\left(1\right)}\left({x^{\prime }}_{1}\;;\;x_{1}\right)}} \end{array}$$

Giving the idempotency property of Equation (49), through multiplying Equation (56) on its right side with the Fock-Dirac density operator

(58a) $$\hat{F}{\hat{\rho }}_{FD}^{\left(1\right)}\;-\;{\hat{\rho }}_{FD}^{\left(1\right)}\hat{\alpha }{\hat{\rho }}_{FD}^{\left(1\right)}\;-\;\hat{\alpha }\underset{{\hat{\rho }}_{FD}^{\left(1\right)}}{\underset{︸}{{\left({\hat{\rho }}_{FD}^{\left(1\right)}\right)}^{2}}}\;+\;\hat{\alpha }{\hat{\rho }}_{FD}^{\left(1\right)}\;-\;\beta {\hat{1}}_{\delta }{\hat{\rho }}_{FD}^{\left(1\right)}\;=\;0$$

while doing the same on left side

(58b) $${\hat{\rho }}_{FD}^{\left(1\right)}\hat{F}\;-\;\underset{{\hat{\rho }}_{FD}^{\left(1\right)}}{\underset{︸}{{\left({\hat{\rho }}_{FD}^{\left(1\right)}\right)}^{2}}}\hat{\alpha }\;-\;{\hat{\rho }}_{FD}^{\left(1\right)}\hat{\alpha }{\hat{\rho }}_{FD}^{\left(1\right)}\;+\;{\hat{\rho }}_{FD}^{\left(1\right)}\hat{\alpha }\;-\;\beta {\hat{\rho }}_{FD}^{\left(1\right)}{\hat{1}}_{\delta }\;=\;0$$

and subtracting the results, one gets the equation

(59a) $$\hat{F}{\hat{\rho }}_{FD}^{\left(1\right)}-{\hat{\rho }}_{FD}^{\left(1\right)}\hat{F}=0$$

that is equivalently of saying that Fock energy operator commutes with the Fock-Dirac density operator

(59b) $$\left\lfloor \hat{F},{\hat{\rho }}_{FD}^{\left(1\right)}\right\rfloor =0$$

meaning that they both admit the same set of eigen-functions. This is nevertheless the gate for obtaining the density (matrix) functional energy expressions by means of finding the density (matrix) eigen-solutions only.

Yet, condition (59b) is indeed a workable (reduced) condition raised from optimization of the averaged Hamiltonian of a many-electronic system, since the more general one referring to the whole Hamiltonian, known as the *Liouville or Neumann equation*, is obtained employing the temporal Schrödinger equation:

(60) $$i\hbar \frac{\partial }{\partial t}|\varphi_{i}\rangle =\hat{H}|\varphi_{i}\rangle$$

to the evolution equation of Fock-Dirac density operator evolution

(61a) $$\begin{array}{c} i\hbar \frac{\partial }{\partial t}{\hat{\rho }}_{FD}^{\left(1\right)}=\sum_{i=1}^{N}i\hbar \frac{\partial }{\partial t}\left(|\varphi_{i}\rangle \langle \varphi_{i}|\right)=\sum_{i=1}^{N}\underset{\hat{H}|\varphi_{i}\rangle }{\underset{︸}{i\hbar \left(\frac{\partial }{\partial t}|\varphi_{i}\rangle \right)}}\langle \varphi_{i}|+\sum_{i=1}^{N}|\varphi_{i}\rangle \underset{\langle \varphi_{i}|\hat{H}}{\underset{︸}{i\hbar \left(\frac{\partial }{\partial t}\langle \varphi_{i}|\right)}}\\ =\hat{H}\left(\sum_{i=1}^{N}|\varphi_{i}\rangle \langle \varphi_{i}|\right)-\left(\sum_{i=1}^{N}|\varphi_{i}\rangle \langle \varphi_{i}|\right)\hat{H}=\left[\hat{H},{\hat{\rho }}_{FD}^{\left(1\right)}\right] \end{array}$$

Lastly, it should be noted that all above properties may be rewritten since considering the *mixed p-order reduced matrix* with the form

(61b) $$\rho^{\left(p\right)}\left({x^{\prime }}_{1}\ldots {x^{\prime }}_{p};x_{1}\ldots x_{p}\right)=\sum_{k}w_{k}\rho_{k}^{\left(p\right)}\left({x^{\prime }}_{1}\ldots {x^{\prime }}_{p};x_{1}\ldots x_{p}\right)$$

as a natural extension of the pure states. However, the sample statistical effects may be better considered by further expressing the electronic density operator and its matrix, the associate equation and the properties for systems in thermodynamic equilibrium (with environment) – a matter addressed in next section.

### 2.2. Canonical Density, Bloch Equation, and the Need of Path Integral

For a quantum system obeying the *N*-mono-electronic eigen-equations

(62) $$\hat{H}|\varphi_{k}\rangle =E_{k}|\varphi_{k}\rangle$$

the probability of finding one particle in the state |φ*_k_*〉 at thermodynamical equilibrium with others, while all states are considered as a closed supra-system with no mass or charge transfer allowed, is given by the canonical distribution [36]

(63) $$w_{k}=\frac{1}{Z\left(\beta \right)}\text{exp}\left(-\beta E_{k}\right)$$

providing the *mixed Fock-Dirac density* with the form

(64) $$\begin{array}{c} {\hat{\rho }}_{N}\left(\beta \right)=\sum_{k=1}^{N}\frac{1}{Z\left(\beta \right)}\underset{\text{exp}\left(-\beta \hat{H}\right)|\varphi_{k}\rangle }{\underset{︸}{\text{exp}\left(-\beta E_{k}\right)|\varphi_{k}\rangle }}\langle \varphi_{k}|=\sum_{k=1}^{N}\frac{1}{Z\left(\beta \right)}\text{exp}\left(-\beta \hat{H}\right)|\varphi_{k}\rangle \langle \varphi_{k}|\\ =\frac{1}{Z\left(\beta \right)}\text{exp}\left(-\beta \hat{H}\right)\sum_{k=1}^{N}|\varphi_{k}\rangle \langle \varphi_{k}|=\frac{1}{Z\left(\beta \right)}\text{exp}\left(-\beta \hat{H}\right)\sum_{k=1}^{N}\left(\sum_{n}c_{kn}|n\rangle \right)\left(\sum_{n}\langle n|c_{kn}^{*}\right)\\ =\frac{1}{Z\left(\beta \right)}\text{exp}\left(-\beta \hat{H}\right)\sum_{k=1}^{N}\underset{1}{\underset{︸}{\left({\sum_{n}\left|c_{kn}\right|}^{2}\right)}}\underset{1}{\underset{︸}{\left(\sum_{n}|n\rangle \langle n|\right)}}=\frac{N}{Z\left(\beta \right)}\text{exp}\left(-\beta \hat{H}\right) \end{array}$$

This is a very interesting and important result motivating the quantum statistical approach in determining the density of states since it corresponds to the *N*-sample particles throughout a simple *N*-multiplication. Note that Equation (64) is in full agreement with that introduced in Equation (12), and very well suited for handling since respecting the DFT custom normalization of Equation (2), while its normalization factor, the partition function *Z*(*β*), follows from such constraint with the consecrated expression

(65) $$Z\left(\beta \right)=\text{Tr}\;\left[\text{exp}\left(-\beta \hat{H}\right)\right]=\int \langle x|e^{-\beta \hat{H}}|x\rangle dx$$

The recognized importance of partition functions in computing the internal energy as the average of the Hamiltonian of the system:

(66) $$U_{N}:=\langle \hat{H}\rangle =\text{Tr}\left[{\hat{\rho }}_{N}\left(\beta \right)\hat{H}\right]=\frac{N}{Z\left(\beta \right)}\text{Tr}\left[\hat{H}\text{exp}\left(-\beta \hat{H}\right)\right]=-N\frac{\partial }{\partial \beta }\text{ln}Z\left(\beta \right)$$

or to evaluate the free energy of the system

(67) $$F_{N}=-N\frac{1}{\beta }\text{ln}Z\left(\beta \right)$$

is thus transferred to the knowledge of the closed evolution amplitude 〈*x|e^–βĤ^*|*x〉*, that at its turn is based on the *genuine* (not-normalized) *density operator*

(68) $${\hat{\rho }}_{⊗}\left(\beta \right)=\text{exp}\left(-\beta \hat{H}\right)$$

sometimes called also like *canonic density operator*.

The great importance of density operator of Equation (68) is immediately visualized in three ways:

○ It identifies the evolution operator
  (69) $$\hat{U}\left(t_{b},t_{a}\right)=\text{exp}\left[-\frac{i}{\hbar }\hat{H}(t_{b}-t_{a})\right]$$
  on the ground of Wick equivalence relationship of Equation (10), which allows the transformation of the Schrödinger into Heisenberg or Interaction pictures for appropriately describing the quantum interactions [53];
○ It produces the so called *Bloch equation* [21] by taking its *β* derivative
  (70a) $$-\frac{\partial {\hat{\rho }}_{⊗}\left(\beta \right)}{\partial \beta }=\hat{H}{\hat{\rho }}_{⊗}\left(\beta \right)$$
  that identifies with the Schrödinger equation for genuine density operator
  (70b) $$i\hbar \frac{\partial }{\partial t}{\hat{\rho }}_{⊗}\left(\beta \right)=\hat{H}{\hat{\rho }}_{⊗}\left(\beta \right)$$
  through the same Wick transformation given by Equation (10), thus providing the quantum-mechanically to quantum-statistical equivalence;
○ Fulfills the (short times, higher temperature) so called Markovian limiting condition

(71) $$\underset{\beta \to 0}{\text{lim}}{\hat{\rho }}_{⊗}\left(\beta \right)=\hat{1}$$

a very useful constraint for developing either the perturbation or the variational formalism respecting electronic density and/or partition function, see below.

In the frame of coordinate representation the Bloch problem, *i.e.*, the differential equation together with the initial (Cauchy) condition, looks like

(72) $$\{\begin{array}{l} -\frac{\partial }{\partial \beta }\rho_{⊗}\left(x^{\prime };x;\beta \right)=\hat{H}\rho_{⊗}\left(x^{\prime };x;\beta \right)\\ \underset{\beta \to 0}{\text{lim}}\rho_{⊗}\left(x^{\prime };x;\beta \right)=\delta \left(x^{\prime }-x\right) \end{array}$$

Solution of this system is a great task in general, unless the perturbation method is undertaken for writing the Hamiltonian as the sum of free and small interaction components

(73) $$\hat{H}={\hat{H}}_{0}+{\hat{H}}_{1}$$

for which the free Hamiltonian solution is completely known, say

(74) $${\hat{\rho }}_{0}\left(\beta \right)=\text{exp}\left(-\beta {\hat{H}}_{0}\right)$$

In these conditions, one may firstly write

(75) $$\frac{\partial }{\partial \beta }\left(e^{\beta {\hat{H}}_{0}}{\hat{\rho }}_{⊗}\right)={\hat{H}}_{0}e^{\beta {\hat{H}}_{0}}{\hat{\rho }}_{⊗}+e^{\beta {\hat{H}}_{0}}\underset{-\hat{H}{\hat{\rho }}_{⊗}}{\underset{︸}{\frac{\partial {\hat{\rho }}_{⊗}}{\partial \beta }}}={\hat{H}}_{0}e^{\beta {\hat{H}}_{0}}{\hat{\rho }}_{⊗}-\left({\hat{H}}_{0}+{\hat{H}}_{1}\right)e^{\beta {\hat{H}}_{0}}{\hat{\rho }}_{⊗}=-e^{\beta {\hat{H}}_{0}}{\hat{H}}_{1}{\hat{\rho }}_{⊗}$$

where the inter-Hamiltonian components were considered to freely commute as *per wish*; then, the Equation (75) is integrated on the realm [0, *β*] to get

(76) $$e^{\beta {\hat{H}}_{0}}{\hat{\rho }}_{⊗}\left(\beta \right)-\hat{1}=-\int_{0}^{\beta }e^{\beta^{\prime }{\hat{H}}_{0}}{\hat{H}}_{1}\left(\beta^{\prime }\right){\hat{\rho }}_{⊗}\left(\beta^{\prime }\right)d\beta^{\prime }$$

that may be rearranged under the perturbative fashion

(77) $${\hat{\rho }}_{⊗}\left(\beta \right)={\hat{\rho }}_{0}\left(\beta \right)-\int_{0}^{\beta }{\hat{\rho }}_{0}\left(\beta -\beta^{\prime }\right){\hat{H}}_{1}\left(\beta^{\prime }\right){\hat{\rho }}_{⊗}\left(\beta^{\prime }\right)d\beta^{\prime }$$

in the form reminding by the *Lippmann-Schwinger equation* for the perturbed dynamical wave-function [32], with *ρ̂*_0_ (*β − β*′) playing the role of the retarded Green function *G*_0_ (*t_b_* – *t_a_*) [34]. Yet, expression (77) may be further generalized for the *p*-order approximation by choosing various *p*-paths of spanning the statistical realm [0, *β*] by intermediate sub-intervals

(78) $$\beta \;=\;\beta_{n+1}\;>\;\beta_{n}\;>\;\ldots \;>\;\beta_{2}\;>\;\beta_{1}\;>\;\beta_{0}\;=\;0$$

thus leaving with the expansion

(79) ρ^⊗(β) = ρ^0(β) + ∑l=1n(−1)l∫0βdβl∫0βldβl−1 … ∫0β2dβ1× ρ^0(β − βl)[H^1(βl)ρ^0(βl − βl−1)]⋯[H^1(β2)ρ^0(β2 − β1)]H^1(β1)ρ^0(β1)

correspondingly written in coordinate representation

(80) $$\begin{array}{c} \rho_{⊗}(x^{\prime };\;x;\;\beta )\;=\;\rho_{0}(x^{\prime };\;x;\;\beta )\;+\;\sum_{l=1}^{n}{\left(-1\right)}^{l}\left(\int_{0}^{\beta }d\beta_{l}\int_{0}^{\beta_{l}}d\beta_{l-1}\;\ldots \;\int_{0}^{\beta_{2}}d\beta_{1}\right)\times \left(\int_{-\infty }^{+\infty }\cdots \int_{-\infty }^{+\infty }\prod_{j=1}^{l}dx_{j}\right)\\ \times \;\rho_{0}(x^{\prime },\;x_{l};\;\beta \;-\;\beta_{l})\left[{\hat{H}}_{1}(\beta_{l})\rho_{0}(x_{l};x_{l-1};\;\beta_{l}\;-\;\beta_{l-1})\right]\cdots \left[{\hat{H}}_{1}(\beta_{2})\rho_{0}(x_{2};\;x_{1};\;\beta_{2}\;-\;\beta_{1})\right]{\hat{H}}_{1}(\beta_{1})\rho_{0}(x_{1}\;;\;x\;;\;\beta_{1}) \end{array}$$

once a parallel slicing of the spatial interval [*x*', *x*] is considered through the subdivisions

(81) $$x^{\prime }=x_{n+1}>x_{n}\ldots >x_{2}>x_{1}>x_{0}=x$$

Such slicing procedure in solving the Bloch equation (72) for canonic density solution (80) seems an elegant way of avoiding the self-consistent equation (77). Therefore, it may further employed through reconsidering the problem (72) in a slightly modified variant, namely within the temporal approach

(82) $$\{\begin{array}{l} -\hbar \frac{\partial }{\partial u}\rho_{⊗}(x^{\prime };x;u)=H(x^{\prime })\rho_{⊗}(x^{\prime };x;u)\\ \rho_{⊗}(x^{\prime };x;u=0)=1 \end{array}$$

where the variable *u* = *ħβ* was considered for the time dimension.

Now, in the first instance, the new problem (82) has the *formal* total solution

(83) $$\rho_{⊗}(x^{\prime };x;u)=\text{exp}\left[-\frac{1}{\hbar }H(x^{\prime })u\right]$$

that being of exponential type allows for direct slicing through factorization. That is, when considering the space partition given by coordinate cuts of (81), and assuming that the times flows equally on each sub-interval in quota of *ε*, *u* = (*n +* 1)*ε*, the density solution (83) may be written as a product of intermediary solutions towards the path integral representation

(84) $$\begin{array}{c} \rho_{⊗}(x^{\prime };x;u)=\prod_{j=0}^{n+1}\text{exp}\left[-\frac{1}{\hbar }H(x_{j})\varepsilon \right]\\ =\int \cdots \int \rho_{⊗}(x^{\prime };x_{l};\varepsilon )\rho_{⊗}(x_{l};x_{l-1};\varepsilon )\cdots \rho_{⊗}(x_{1};x;\varepsilon )\prod_{j=1}^{n}dx_{j}\\ \overset{n\to \infty }{\underset{\begin{array}{c} \varepsilon \to 0\\ (n+1)\varepsilon =u \end{array}}{\to }}\int \ldots \int \Lambda [x(u)]Dx(u) \end{array}$$

where the chained covariant density product was introduced

(85) $$\Lambda [x(u)]=\;\underset{\begin{array}{c} n\to \infty \\ \varepsilon \to 0\\ (n+1)\varepsilon =u \end{array}}{\text{lim}}\rho_{⊗}(x^{\prime };x_{l};\varepsilon )\rho_{⊗}(x_{l};x_{l-1};\varepsilon )\cdots \rho_{⊗}(x_{1};x;\varepsilon ),$$

along the extended integration metric

(86) $$Dx(u)=\underset{n\to \infty }{\text{lim}}\prod_{j=1}^{n}dx_{j}$$

The general canonic solution (84) is viewed as the path integral solution for the Bloch equation (82), being therefore as a necessity when looking to general solutions for a given Hamiltonian; it gives the general solution for electronic density (68) since accounting for all path connecting two end-points either in space and time (or temperatures) through in principle an infinite intermediary points; this way the resulted path integral comprises all quantum information contained by the particle’ evolution between two states in thermodynamical equilibrium with environment (or the other mono-particle states). However, once having the canonical density evaluated by its path integral, the associate mixed density matrix may be immediately written employing the operatorial form (64) to the actual spatial representation

(87) $$\rho_{N}(x^{\prime };x;u)=\frac{N}{Z(u)}\rho_{⊗}(x^{\prime };x;u)$$

with the path integral based partition function written in accordance with Equation (65)

(88) $$Z(u)\int \rho_{⊗}(x;x;u)dx$$

assuring the preservation of the general DFT normalization condition

(89) $${\int \rho }_{N}(x;x;u)dx=N$$

This way, the general algorithm linking the path integral to the density matrix and to the electronic density, most celebrated DFT quantity in computing various density functionals (energies, reactivity indices) for characterizing chemical structure and reactivity – was established, while emphasizing the basic role the path integral evaluation has towards a conceptual understanding of many-electronic quantum systems in their dynamics and interaction.

Being thus established the role and usefulness of path integral in density functional theory the next section will give more insight in appropriately defining (constructing) path integral such that to further facilitate its practical evaluation for electronic systems of physical-chemical interest.

## 3. Feynman’s Path Integral of Evolution Amplitude

### 3.1. Construction of the General Path Integral

Through reconsidering the slicing of (81) also for the time interval [*t_b_*, *t_a_*]

(90) $$t_{b}=t_{n+1}>t_{n}>\ldots >t_{2}>t_{1}>t_{0}=t_{a}$$

with the spatial ending points recalled as *x*′ = *x_b_*, *x* = *x_a_* the quantum propagator of Equation (9), within the Wick equivalence (10)

(91) $$(x_{b}t_{b};x_{a}t_{a})=\langle x_{b}|\text{exp}\left(-\frac{i}{\hbar }(t_{b}-t_{a})\hat{H}\right)|x_{a}\rangle$$

may be firstly rewritten in terms of associate evolution operator

(92) $$\hat{U}(t_{b}-t_{a})=\text{exp}\left(-\frac{i}{\hbar }(t_{b}-t_{a})\hat{H}\right)$$

to successively become

(93) $$\begin{array}{c} (x_{b}t_{b};x_{a}t_{a})=\langle x_{b}|\hat{U}(t_{b}-t_{a})|x_{a}\rangle \\ =\langle x_{b}|\hat{U}(t_{b},t_{n})\hat{U}(t_{n},t_{n-1})\ldots \hat{U}(t_{j},t_{j-1})\ldots \hat{U}(t_{2},t_{1})\hat{U}(t_{1},t_{a})|x_{a}\rangle \\ =\prod_{j=1}^{n}\left[\int_{-\infty }^{+\infty }dx_{j}\right]\prod_{j=1}^{n+1}\left(x_{j}t_{j};x_{j-1}t_{j-1}\right) \end{array}$$

where the last form was obtained when *n*-times the complete eigen-coordinate set

(94) $$\hat{1}=\int_{-\infty }^{+\infty }\left|x_{j}〉〈x_{j}\right|dx_{j},\;j=\bar{1,n}$$

was introduced for each pair of events with the elementary propagator between them

(95) $$(x_{j}t_{j};x_{j-1}t_{j-1})=\langle x_{j}|\text{exp}\left(-\frac{i}{\hbar }(t_{j}-t_{j-1})\hat{H}\right)|x_{j-1}\rangle =\langle x_{j}|\text{exp}\left(-\frac{i}{\hbar }\varepsilon \hat{H}\right)|x_{j-1}\rangle$$

on the elementary time interval

(96) $$\varepsilon =t_{j}-t_{j-1}=\frac{t_{b}-t_{a}}{n+1}>0$$

Now, the elementary quantum evolution amplitude (95) is to be evaluated, firstly by reconsidering the eigen-coordinate unitary operator, in the working form

(97) $${\hat{1}}_{x}=\int_{-\infty }^{+\infty }|x\rangle \langle x|dx$$

to separate the operatorial Hamiltonian contributions to the kinetic and potential ones

(98) $$\hat{H}=\hat{T}+\hat{V}$$

yielding:

(99) $$\begin{array}{c} (x_{j}t_{j};x_{j-1}t_{j-1})≅\langle x_{j}|e^{-\frac{i}{\hbar }\varepsilon \hat{V}(\hat{x},t_{j})}{\hat{1}}_{x}e^{-\frac{i}{\hbar }\varepsilon \hat{T}(\hat{p},t_{j})}|x_{j-1}\rangle \\ =\int_{-\infty }^{+\infty }\langle x_{j}|e^{-\frac{i}{\hbar }\varepsilon \hat{V}(\hat{x},t_{j})}|x\rangle {\langle x|e}^{-\frac{i}{\hbar }\varepsilon \hat{T}(\hat{p},t_{j})}|x_{j-1}\rangle dx \end{array}$$

where we have used the first order limitation of the Baker-Hausdorff formula

(100) $$e^{-\frac{i}{\hbar }\varepsilon [\hat{V}(\hat{x},t_{j})+\hat{T}(\hat{p},t_{j})]}=e^{-\frac{1}{\hbar }\varepsilon \hat{V}(\hat{x},t_{j})}e^{-\frac{i}{\hbar }\varepsilon \hat{T}(\hat{p},t_{j})}+\underset{≅0}{\underset{︸}{O(\varepsilon^{2})}}$$

by assuming the second order of elementary time intervals as vanishing

(101) $$\varepsilon^{2}≅0$$

Next, each obtained working energetic contribution is separately evaluated: for kinetic contribution the insertion of the momentum complete eigen-set

(102) $${\hat{1}}_{p_{j}}=\int_{-\infty }^{+\infty }|p_{j}\rangle \langle p_{j}|dp_{j}$$

yields

(103) $$\begin{array}{c} {\langle x|e}^{-\frac{i}{\hbar }\varepsilon \hat{T}(\hat{p},t_{j})}|x_{j-1}\rangle ={\int_{-\infty }^{+\infty }\langle x|e}^{-\frac{i}{\hbar }\varepsilon \hat{T}(\hat{p},t_{j})}|p_{j}\rangle \langle p_{j}|x_{j-1}\rangle dp_{j}\\ =\int_{-\infty }^{+\infty }e^{-\frac{i}{\hbar }\varepsilon T(p_{j},t_{j})}\underset{\frac{1}{\sqrt{2\pi \hbar }}e^{\frac{i}{\hbar }p_{j}x}}{\underset{︸}{\langle x|p_{j}\rangle }}\underset{\frac{1}{\sqrt{2\pi \hbar }}e^{-\frac{i}{\hbar }p_{j}x_{j-1}}}{\underset{︸}{\langle p_{j}|x_{j-1}\rangle }}dp_{j}=\int_{-\infty }^{+\infty }e^{\frac{i}{\hbar }p_{j}(x-x_{j-1})}e^{-\frac{i}{\hbar }\varepsilon T(p_{j},t_{j})}\frac{dp_{j}}{2\pi \hbar } \end{array}$$

while for potential elementary amplitude one gets

(104) $$\langle x_{j}|e^{-\frac{i}{\hbar }\varepsilon \hat{V}(\hat{x},t_{j})}|x\rangle =e^{-\frac{i}{\hbar }\varepsilon V(x,t_{j})}\underset{\delta (x_{j}-x)}{\underset{︸}{\langle x_{j}|x\rangle }}=\delta (x_{j}-x)e^{-\frac{i}{\hbar }\varepsilon V(x,t_{j})}$$

With relations (103) and (104) back in (100) the elementary propagator takes the form

(105) $$\begin{array}{c} (x_{j}t_{j};x_{j-1}t_{j-1})=\int_{-\infty }^{+\infty }\left(\int_{-\infty }^{+\infty }e^{\frac{i}{\hbar }p_{j}(x-x_{j-1})}e^{-\frac{i}{\hbar }\varepsilon T(p_{j},t_{j})}\frac{dp_{j}}{2\pi \hbar }\right)\left(\delta (x_{j}-x)e^{-\frac{i}{\hbar }\varepsilon V(x,t_{j})}\right)dx\\ =\int_{-\infty }^{+\infty }\frac{dp_{j}}{2\pi \hbar }\text{exp}\left[\frac{ip_{j}(x_{j}-x_{j-1})}{\hbar }-i\varepsilon \frac{T(p_{j},t_{j})+V(x_{j},t_{j})}{\hbar }\right]\\ =\int_{-\infty }^{+\infty }\frac{dp_{j}}{2\pi \hbar }\text{exp}\left\{\frac{i}{\hbar }[p_{j}(x_{j}-x_{j-1})-\varepsilon H(x_{j},p_{j},t_{j})]\right\} \end{array}$$

Replacing the elementary quantum amplitude (105) back into the global one given by Equation (93), it takes the form

(106) $$\begin{array}{c} (x_{b}t_{b};x_{a}t_{a})≅\prod_{j=1}^{n}\left[\int_{-\infty }^{+\infty }dx_{j}\right]\prod_{j=1}^{n+1}\int_{-\infty }^{+\infty }\frac{dp_{j}}{2\pi \hbar }\text{exp}\left\{\varepsilon \frac{i}{\hbar }\left[p_{j}\frac{x_{j}-x_{j-1}}{\varepsilon }-H(x_{j},p_{j},t_{j})\right]\right\}\\ =\left(\prod_{j=1}^{n}\left[\int_{-\infty }^{+\infty }dx_{j}\right]\right)\left(\prod_{j=1}^{n+1}\left[\int_{-\infty }^{+\infty }\frac{dp_{j}}{2\pi \hbar }\right]\right)\text{exp}\left\{\frac{i}{\hbar }\varepsilon \sum_{j=1}^{n+1}\left[p_{j}\frac{x_{j}-x_{j-1}}{\varepsilon }-H(x_{j},p_{j},t_{j})\right]\right\} \end{array}$$

which for an infinitesimal temporal partition, *i.e.*

(107) n→∞;ε→0

behaves like the *Feynman path integral* of the quantum propagator [37,40,41,54–60]

(108) $$(x_{b}t_{b};x_{a}t_{a})\equiv \int_{x(t_{a})=x_{a}}^{x(t_{b})=x_{b}}DxDp\text{exp}\left\{\frac{i}{\hbar }S[x,p,t]\right\}$$

through considering the limiting notations for the path integral measure

(109) $$\underset{n\to \infty }{\text{lim}}\left(\prod_{j=1}^{n}\left[\int_{-\infty }^{+\infty }dx_{j}\right]\right)\left(\prod_{j=1}^{n+1}\left[\int_{-\infty }^{+\infty }\frac{dp_{j}}{2\pi \hbar }\right]\right)\equiv \int_{x(t_{a})=x_{a}}^{x(t_{b})=x_{b}}DxDp$$

and for the involved action

(110) $$\begin{array}{c} \underset{\begin{array}{c} n\to \infty \\ \varepsilon \to 0 \end{array}}{\text{lim}}\varepsilon \sum_{j=1}^{n+1}\left[p_{j}\frac{x_{j}-x_{j-1}}{\varepsilon }-H(x_{j},p_{j},t_{j})\right]\\ \overset{\left(96\right)}{\equiv }\int_{t_{a}}^{t_{b}}[p(t)\dot{x}(t)-H(x(t),p(t),t)]dt=\int_{t_{a}}^{t_{b}}L(x(t),p(t),t)dt=S[x,p,t] \end{array}$$

Note that the results (108)–(110) give similar quantum information for the quantum evolution of a system as previously found with the mean of density matrix (84)–(86), yet in a more formal and general way throughout accounting all histories (possibilities for linking two events in time-space) for a quantum evolution [61–63]

(111) $$(x_{b}t_{b};x_{a}t_{a})=\sum_{\begin{array}{c} ALL\\ HISTORIES \end{array}}\text{exp}\left\{\frac{i}{\hbar }S[x,p,t]\right\}$$

thus being suitable to be implemented in the *N*-particle density functional scheme (87)–(89) once it is analytically computed.

For achieving such goal, a more practical form of the Feynman integral may be obtained once the Hamiltonian is implemented as

(112) $$H=\frac{p^{2}}{2m}+V(x,t)$$

leaving the action (110) unfolded as

(113) $$\begin{array}{c} S[x,p,t]=\sum_{j=1}^{n+1}\left[p_{j}(x_{j}-x_{j-1})-\varepsilon \frac{p_{j}^{2}}{2m}-\varepsilon V(x_{j},t_{j})\right]\\ =\sum_{j=1}^{n+1}\left[-\frac{\varepsilon }{2m}{\left(p_{j}-\frac{x_{j}-x_{j-1}}{\varepsilon }m\right)}^{2}+\varepsilon \frac{m}{2}{\left(\frac{x_{j}-x_{j-1}}{\varepsilon }\right)}^{2}-\varepsilon V(x_{j},t_{j})\right] \end{array}$$

from where the momentum integrals in (109) is immediately solved to be

(114) $$\int_{-\infty }^{+\infty }\frac{dp_{j}}{2\pi \hbar }\text{exp}\left\{-\frac{i}{\hbar }\frac{\varepsilon }{2m}{\left(p_{j}-\frac{x_{j}-x_{j-1}}{\varepsilon }m\right)}^{2}\right\}=\sqrt{\frac{m}{2\pi \hbar i\varepsilon }}$$

by formally applying the Poisson formula

(115) $$\int_{-\infty }^{+\infty }\text{exp}(-\Xi y^{2})dy=\sqrt{\frac{\pi }{\Xi }}.$$

The remaining quantum evolution amplitude reads as the spatial path integral only

(116) $$\begin{array}{c} (x_{b}t_{b};x_{a}t_{a})=\left(\prod_{j=1}^{n}\left[\int_{-\infty }^{+\infty }dx_{j}\right]\right)\left(\prod_{j=1}^{n+1}\left[\sqrt{\frac{m}{2\pi \hbar i\varepsilon }}\right]\right)\text{exp}\left\{\frac{i}{\hbar }\sum_{j=1}^{n+1}\left[\varepsilon \frac{m}{2}{\left(\frac{x_{j}-x_{j-1}}{\varepsilon }\right)}^{2}-\varepsilon V(x_{j},t_{j})\right]\right\}\\ =\frac{1}{\sqrt{2\pi \hbar i\varepsilon /m}}\prod_{j=1}^{n}\left[\int_{-\infty }^{+\infty }\frac{dx_{j}}{\sqrt{2\pi \hbar i\varepsilon /m}}\right]\text{exp}\left\{\frac{i}{\hbar }\varepsilon \sum_{j=1}^{n+1}\left[\frac{m}{2}{\left(\frac{x_{j}-x_{j-1}}{\varepsilon }\right)}^{2}-V(x_{j},t_{j})\right]\right\}\\ \overset{n\to \infty }{\underset{\varepsilon \to 0}{\to }}\int_{x(t_{a})=x_{a}}^{x(t_{b})=x_{b}}D^{\prime }x\text{exp}\left\{\frac{i}{\hbar }S[x,\dot{x},t]\right\} \end{array}$$

assuming the actual modified measure of integration

(117) $$\int_{x(t_{a})=x_{a}}^{x(t_{b})=x_{b}}D^{\prime }x\equiv \underset{\begin{array}{l} n\to \infty \\ \varepsilon \to 0 \end{array}}{\text{lim}}\left(\frac{1}{\sqrt{2\pi \hbar i\varepsilon /m}}\prod_{j=1}^{n}\left[\int_{-\infty }^{+\infty }\frac{dx_{j}}{\sqrt{2\pi \hbar i\varepsilon /m}}\right]\right)$$

and the working action

(118) $$S[x,\dot{x},t]=\int_{t_{a}}^{t_{b}}L(x,\dot{x},t)dt=\int_{t_{a}}^{t_{b}}\left[{\frac{m\dot{x}}{2}}^{2}-V(x,t)\right]dt$$

Note that when the partition function (88) is under consideration, other path integral out of (116) has to be introduced by means of closed space coordinates, namely

(119) $$Z(t_{b};t_{a})=\int_{-\infty }^{+\infty }(xt_{b};xt_{a})dx=\int_{x(t_{a})=x(t_{b})}D^{\prime \prime }x\text{exp}\left\{\frac{i}{\hbar }S[x,\dot{x},t]\right\}$$

noting the new integration measure

(120) $$\int_{x(t_{a})=x(t_{b})}D^{\prime \prime }x\equiv \underset{n\to \infty }{\text{lim}}\left(\prod_{j=1}^{n+1}\left[\int_{-\infty }^{+\infty }\frac{dx_{j}}{\sqrt{2\pi \hbar i\varepsilon /m}}\right]\right)$$

Therefore, at the first instance, some of the main advantages dealing with path integrals relay on following features:

○ Attractive conceptual representation of dynamical quantum processes without operatorial excursion;
○ Allows for quantum fluctuation description in analogy with thermic description, through changing the temporal intervals with the thermodynamical temperature by means of Wick transformation (10), *i.e.*, transforming quantum mechanical (QM) into quantum statistical (QS) propagators

(121a) $$\begin{array}{c} {\left(x_{b}t_{b};x_{a}t_{a}\right)}_{QM}=\int_{x(t_{a})=x_{a}}^{x(t_{b})=x_{b}}D^{\prime }x(t)\text{exp}\left\{\frac{i}{\hbar }\int_{t_{a}}^{t_{b}}\left[{\frac{m\dot{x}}{2}}^{2}-V(x(t),t)\right]dt\right\}\\ ↓|\begin{array}{c} t:=-i\tau ,\tau =\hbar \beta \\ dt=-id\tau \\ \frac{d}{dt}=i\frac{d}{d\tau } \end{array} \end{array}$$

(121b) $${\left(x_{b}\hbar \beta ;x_{a}0\right)}_{QS}=\int_{x(0)=x_{a}}^{x(\hbar \beta )=x_{b}}D^{\prime }x(\tau )\text{exp}\left\{-\frac{1}{\hbar }\int_{0}^{\hbar \beta }\left[\frac{m}{2}{\dot{x}}^{2}(\tau )+V(x(\tau ),\tau )\right]d\tau \right\}$$

from where the immediate writing of the associate QS-partition function

(121c) $$Z_{QS}(\beta )=\int_{-\infty }^{+\infty }{\left(x_{b}\hbar \beta ;x_{a}0\right)}_{QS}dx=\int_{x(0)=x(\hbar \beta )}D^{\prime \prime }x\text{exp}\left\{-\frac{1}{\hbar }\int_{0}^{\hbar \beta }L^{+}(x,\dot{x},\tau )d\tau \right\}$$

having both QS objects written as the effect of transforming the *canonical Lagrangean* of action into the so called *Euclidian* one

(121d) $$L^{+}(x,\dot{x},\tau )=\frac{m}{2}{\dot{x}}^{2}(\tau )+V(x(\tau ),\tau )$$

analogously with the fact the Euclidian metric has all its diagonal terms positively defined.

Yet, the connection of the path integrals of propagators with the Schrödinger quantum formalism is to be revealed next.

### 3.2. Schrödinger Equation from Path Integral

There are two ways for showing the propagator path integral links with Schrödinger equation.

#### 3.2.1. Propagator’s Equation

Firstly, by employing one of the above path integral, say that of Equation (116) with (118)

(122) $$(x_{b}t_{b};x_{a}t_{b})=\int_{x(t_{a})=x_{a}}^{x(t_{b})=x_{b}}D^{\prime }x\text{exp}\left\{\frac{i}{\hbar }S[x,\dot{x},t]\right\}$$

to perform the derivative

(123) ∂∂xb(xb,tb;xa,ta)=(xb+δx(tb),tb;xa,ta)−(xb,tb;xa,ta)δx(tb)=1δx(tb)[∫x(ta)=xax(tb)=xbD′xexp{iℏS[x(t)+δx(t)]}−∫x(ta)=xax(tb)=xbD′xexp{iℏS[x(t)]}]=1δx(tb)δS∫x(ta)=xax(tb)=xbD′x exp{iℏS[x(t)+δx(t)]}−∫x(ta)=xax(tb)=xbD′xexp{iℏS[x(t)]}δS=1δx(tb)δSδ(xb,tb;xa,ta)δS=1δx(tb)δSiℏ(xb,tb;xa,ta)=1δx(tb)[∫tatb(∂L∂xδx+∂L∂x˙δx˙)dt]iℏ(xa,tb;xa,ta)=1δx(tb)[∫tatb(∂L∂xδx+∂L∂x˙ddtδx)dt]1ℏ(xb,tb;xa,ta)=1δx(tb)[∂L∂x˙∂∂tδx|tatb+∫tatb(∂L∂x−ddt∂L∂x˙)︸=0Euler−Lagrange equationδxdt]iℏ(xb,tb;xa,ta)=1δx(tb)[∂L∂x˙(tb)∂∂tδx(tb)︸≠0−∂L∂x˙(ta)∂∂tδx(ta)︸=0]iℏ(xb,tb;xa,tb)=∂L∂x˙(tb)︸p(tb)iℏ(xb,tb;xa,ta)=[iℏp(tb)](xb,tb;xa,ta)

Similarly for the second derivative we have

(124) $$\frac{\partial }{\partial x_{b}^{2}}(x_{b},t_{b};x_{a},t_{a})={\left[\frac{i}{\hbar }p(t_{b})\right]}^{2}(x_{b},t_{b};x_{a},t_{a})=-\frac{p^{2}(t_{b})}{\hbar^{2}}(x_{b},t_{b};x_{a},t_{a})$$

while for time derivative we obtain

(125) $$\frac{\partial }{\partial t_{b}}(x_{b},t_{b};x_{a},t_{b})=\frac{\delta S}{\delta t_{b}}\frac{i}{\hbar }(x_{b},t_{b};x_{a},t_{a})=-\frac{i}{\hbar }H(t_{b})(x_{b},t_{b};x_{a},t_{a})$$

by recalling the Hamilton-Jacobi equation of motion in the form

(126) $$\frac{\delta S}{\delta t_{b}}=-H(t_{b})$$

Now, there is immediate that for a Hamiltonian of the form (112) one gets through multiplying both its side with the propagator (122) and then considering the relations (124) and (126), respectively, one leaves with the Schrödinger type equation for the path integral

(127) $$i\hbar \frac{\partial }{\partial t_{b}}(x_{b},t_{b};x_{a},t_{a})=\left[-\frac{\hbar^{2}}{2m}\frac{\partial }{\partial x_{b}^{2}}+V(x_{b})\right](x_{b},t_{b};x_{a},t_{a})$$

Remarkably, besides establishing the link with the Schrödinger picture, equation (127) tells something more important, namely that the wave function itself, *i.e.*, Ψ(*x_b_*, *t_b_*), may be replaced (and generalized as well) by the quantum propagator (*x_b_*, *t_b_*; *x_a_*, *t_a_*); this has a crucial consequence since the propagator is providing the *N*-electronic density in the directly and elegantly manner prescribed by the algorithm (87)–(89), here actualized as

(128) $$\rho_{N}(x;t_{b}-t_{a})=\frac{N}{Z(t_{b};t_{a})}(x_{b},t_{b};x_{a},t_{a}){|}_{x_{b}=x_{b}=x}$$

with partition function given as in (119), assuring for the correct *N*-representability (DFT) constraint:

(129) $$\int \rho_{N}(x;t_{b}-t_{a})dx=N$$

thus nicely replacing the complicated many-body wave function calculations.

Nevertheless, the path integral formalism is able to provide also *the exact* Schrödinger equation for the wave function, as will be shown in the sequel.

#### 3.2.2. Wave Function’s Equation

The starting point is the manifested *equivalence* between the path integral propagator and the Green function, with the role in transforming the wave-function registered on a space-time event into other one, either in the future of past quantum evolution. Here we consider only retarded phenomena modeled by the propagator

(130) $$\left(x_{2},t_{2};x_{1},t_{1})=iG^{+}(x_{2},t_{2};x_{1},t_{1}\right)$$

which in accordance with the very beginning path integral construction, the slicing (90) and the relation (91), implies the existence of the so called *quantum Huygens principle of wave-packet propagatio*n [64]

(131) $$\psi (x_{2},t_{2})=\int \left(x_{2},t_{2};x_{1},t_{1})\psi (x_{1},t_{1}\right)dx_{1},t_{2}>t_{1}$$

**Figure 1.**
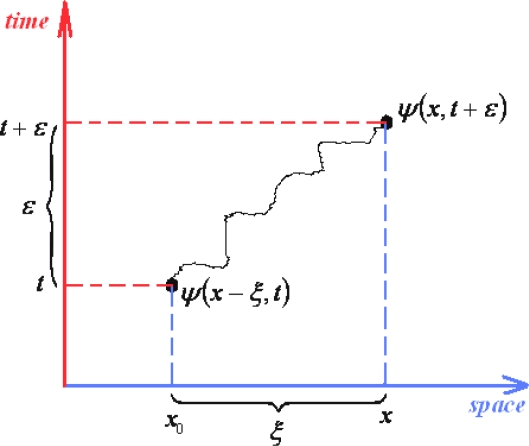
Depiction of the space-time elementary retarded path connecting two events characterized by their dynamic wave-functions.

Yet, we will employ Equation (131) for an *elementary* propagator, modeling the quantum evolution presented in Figure 1, thus behaving like

(132a) $$\psi (x,t+\varepsilon )=A\int \text{exp}\left[\frac{i}{\hbar }\varepsilon L\left(\frac{x+x_{0}}{2},\frac{x-x_{0}}{2},t+\frac{\varepsilon }{2}\right)\right]\psi (x-\xi ,t)dx_{0}$$

where *A* plays the role of the normalization constant in (132a) to assure the convergence of the wave function wave-packet. Equation (132a) may be still transformed employing the geometrical relation

(133a) $$x=x_{0}+\xi$$

to compute the space and velocity averages

(133b) $$\frac{x+x_{0}}{2}=\frac{2x-\xi }{2}=x-\frac{\xi }{2}$$

(133c) $$\frac{x-x_{0}}{\varepsilon }=\frac{\xi }{\varepsilon }$$

respectively, while changing the variable

(133d) $$dx_{0}=-d\xi$$

to furnish the actual form

(132b) $$\begin{array}{c} \psi (x,t+\varepsilon )=\tilde{A}\int \text{exp}\left\{\frac{i}{\hbar }\varepsilon \left[\frac{m}{2}\frac{\xi^{2}}{\varepsilon^{2}}-V\left(x-\frac{\xi }{2},t+\frac{\varepsilon }{2}\right)\right]\right\}\psi (x-\xi ,t)d\xi \\ =\tilde{A}\int \text{exp}\left[\frac{im}{2\hbar \varepsilon }\xi^{2}\right]\text{exp}\left[-\frac{i}{\hbar }\varepsilon V\left(x-\frac{\xi }{2},t+\frac{\varepsilon }{2}\right)\right]\psi (x-\xi ,t)d\xi \end{array}$$

where Lagrangean was considered with its canonical form, as in (118), and the new constant factor was considered assimilating the minus sign of (133d).

Next, since noting the square dependence of *ξ* in (132b) there will be assumed the series expansion in coordinate (*ξ*) and time (*ε*) elementary steps restrained to the second and first order, respectively, being the time interval cut-off in accordance with the general (101) prescription. Thus we firstly have

(134a) $$\psi (x-\xi ,t)≅\psi (x,t)-\xi {\left[\frac{\partial }{\partial x}\psi (x,t)\right]}_{\xi \to 0}+\frac{\xi^{2}}{2}{\left[\frac{\partial^{2}}{\partial x^{2}}\psi (x,t)\right]}_{\xi \to 0}$$

(134b) $$\psi (x,t+\varepsilon )≅\psi (x,t)+\varepsilon {\left[\frac{\partial }{\partial t}\psi (x,t)\right]}_{\varepsilon \to 0}$$

(134c) $$\text{exp}\left[-\frac{i}{\hbar }\varepsilon V\left(x-\frac{\xi }{2},t+\frac{\varepsilon }{2}\right)\right]≅1-\frac{i}{\hbar }\varepsilon V(x,t)$$

and the form (132b) successively rearranges:

(135) $$\begin{array}{c} \psi (x,t)+\varepsilon \left[\frac{\partial }{\partial t}\psi (x,t)\right]\\ =\tilde{A}\int e^{-\frac{m}{2i\hbar \varepsilon }\xi^{2}}\left[1-\frac{i}{\hbar }\varepsilon V(x,t)\right]\left\{\psi (x,t)-\xi \left[\frac{\partial }{\partial x}\psi (x,t)\right]+\frac{\xi^{2}}{2}\left[\frac{\partial^{2}}{\partial x^{2}}\psi (x,t)\right]\right\}d\xi \\ =\tilde{A}\psi (x,t)\int e^{-\frac{m}{2i\hbar \varepsilon }\xi^{2}}d\xi -\tilde{A}\left[\frac{\partial }{\partial x}\psi (x,t)\right]\int \xi e^{-\frac{m}{2i\hbar \varepsilon }\xi^{2}}d\xi +\tilde{A}\frac{1}{2}\left[\frac{\partial^{2}}{\partial x^{2}}\psi (x,t)\right]\int \xi^{2}e^{-\frac{m}{2i\hbar \varepsilon }\xi^{2}}\;d\xi \\ -\tilde{A}\frac{i}{\hbar }\varepsilon V(x,t)\psi (x,t)\int e^{-\frac{m}{2i\hbar \varepsilon }\xi^{2}}d\xi +\tilde{A}\frac{i}{\hbar }\varepsilon V(x,t)\left[\frac{\partial }{\partial x}\psi (x,t)\right]\int \xi e^{-\frac{m}{2i\hbar \varepsilon }\xi 2}d\xi \end{array}$$

where the mixed orders producing a total order beyond the maximum equal two have been neglected, *e.g.*, *εξ*^2^ ≅ 0, and were we arranged the exponentials under integrals of Gaussian type (*i.e.*, employing the identity –*i* =1/ *i*). Now, the integrals appearing on (135) are of Poisson type of various orders and, assuming the notation:

(136) $$\frac{m}{2\hbar \varepsilon i}\equiv \Xi$$

are solved as:

(137a) $$\int e^{-\frac{m}{2i\hbar \varepsilon }\xi^{2}}d\xi \to \int_{-\infty }^{+\infty }\text{exp}\left(-\Xi \xi^{2}\right)d\xi =\sqrt{\frac{\pi }{\Xi }}=\sqrt{\frac{2\pi \hbar \varepsilon i}{m}}$$

(137b) $$\int \xi e^{-\frac{m}{2i\hbar \varepsilon }\xi^{2}}d\xi \to \int_{-\infty }^{+\infty }\text{exp}\left(-\Xi \xi^{2}\right)d\xi =0$$

(137c) $$\int \xi^{2}e^{-\frac{m}{2i\hbar \varepsilon }\xi^{2}}d\xi \to \int_{-\infty }^{+\infty }\xi^{2}\text{exp}\left(-\Xi \xi^{2}\right)d\xi =\frac{1}{2\Xi }\sqrt{\frac{\pi }{\Xi }}=\frac{\hbar \varepsilon i}{m}\sqrt{\frac{2\pi \hbar \varepsilon i}{m}}$$

With these the expression (135) simplifies to

(138) $$\psi (x,t)+\varepsilon \left[\frac{\partial }{\partial t}\psi (x,t)\right]=\tilde{A}\sqrt{\frac{2\pi \hbar \varepsilon i}{m}}\left[1+\frac{1}{2}\frac{\hbar \varepsilon i}{m}\frac{\partial^{2}}{\partial x^{2}}-\frac{i}{\hbar }\varepsilon V(x,t)\right]\psi (x,t)$$

which in the limit *ε* → 0, commonly for path integrals, leaves with identity

(139a) $$\psi (x,t)=\underset{\varepsilon \to 0}{\text{lim}}\left(\tilde{A}\sqrt{\frac{2\pi \hbar \varepsilon i}{m}}\right)\psi (x,t)$$

from where the convergence constant of path integral (132b) is found

(139b) $$\tilde{A}(\varepsilon )=\sqrt{\frac{m}{2\pi \hbar \varepsilon i}}$$

recovering the previous form, see Equation (114), thus confirming the consistency of the present approach. Nevertheless, with the constant (139b) back in (138) we get the equivalent forms

(140) $$\begin{array}{c} \psi (x,t)+\varepsilon \left[\frac{\partial }{\partial t}\psi (x,t)\right]=\psi (x,t)+\frac{1}{2}\frac{\hbar \varepsilon i}{m}\frac{\partial^{2}}{\partial x^{2}}\psi (x,t)-\frac{i}{\hbar }\varepsilon V(x,t)\psi (x,t)\\ \Leftrightarrow \frac{\partial }{\partial t}\psi (x,t)=\frac{1}{2}\frac{\hbar i}{m}\frac{\partial^{2}}{\partial x^{2}}\psi (x,t)-\frac{i}{\hbar }V(x,t)\psi (x,t)\\ \Leftrightarrow i\hbar \frac{\partial }{\partial t}\psi (x,t)=\left[-\frac{1}{2}\frac{\hbar^{2}}{m}\frac{\partial^{2}}{\partial x^{2}}+V(x,t)\right]\psi (x,t) \end{array}$$

being the last one identical with the Schrödinger wave function equation.

Thus it was therefore thoroughly proven that the Feynman path integral may be reduced to the quantum wave-packet motion while carrying also the information that connects coupled events across the paths’ evolution, being by all of these a general approach of quantum mechanics and statistics.

The next section will deal with presenting practical application/calculation of the path integrals for fundamental quantum systems, *e.g.*, the free and harmonic oscillator motions.

### 3.3. Calculation of Path Integrals. Basic Applications

#### 3.3.1. Path Integrals’ Properties

There are three fundamental properties most useful for path integral calculations [65].

- Firstly, one may combine the two above Schrödinger type bits of information about path integrals: the fact that propagator itself (*x_b_*, *t_b_*; *x_a_*, *t_a_*) obeys the Schrödinger equation, see Equation (127), thus behaving like a sort of wave-function, and the fact that Schrödinger equation of the wave-function is recovered by the quantum Huygens principle of wave-packet propagation, see Equation (131). Thus it makes sense to rewrite Equation (131) with the propagator instead of wave-function obtaining the so called *group property for propagators*
  (141) $$(x_{3},t_{3};x_{1},t_{1})=\int \left(x_{3},t_{3};x_{2},t_{2}\right)(x_{2},t_{2};x_{1},t_{1})dx_{2},t_{3}>t_{2}>t_{1}$$
  which, nevertheless, may be recursively applied until covering the entire time slicing of the interval [*t_a_*, *t_b_*] as given in (90)
  (142) $$(x_{b},t_{b};x_{a},t_{a})=\int \left(x_{b},t_{b};x_{n},t_{n})(x_{n},t_{n};x_{n-1},t_{n-1})\cdots (x_{1},t_{1};x_{a},t_{a}\right)\prod_{j=1}^{n}dx_{j}$$
  while remarking the absence of time intermediate integration.
- Secondly, from the Huygens principle (131) there is abstracted also the limiting delta Dirac-function for a propagator connecting two space events simultaneously
  (143) $$\left(x,t_{1};x_{1},t_{1})=\delta (x-x_{1}\right)$$
  that is immediately proofed out
  (144) $$\psi (x,t_{1})=\int \left(x,t_{1};x_{1},t_{1}\right)\psi (x_{1},t_{1})dx_{1}=\int \delta (x-x_{1})\psi (x_{1},t_{1})dx_{1}$$
  This property is often used as the analytical check once a path integral propagator is calculated for a given system.
- Thirdly, and perhaps most practically, one would like to be able to solve the path integrals, say with canonical Lagrangean form (121a), in more direct way than to consider all multiple integrals involved by the measure (117).

Hopefully, this is possible working out the quantum fluctuations along the classical path connecting two space-time events. In other words, worth to disturb the classical path *x_cl_* (*t*) by the quantum fluctuations *δx*(*t*) to obtain the quantum evolution path

(145a) $$x(t)=x_{cl}(t)+\delta x(t)$$

with its temporal derivation

(145b) $$\dot{x}(t)={\dot{x}}_{cl}(t)+\delta \dot{x}(t)$$

**Figure 2.**
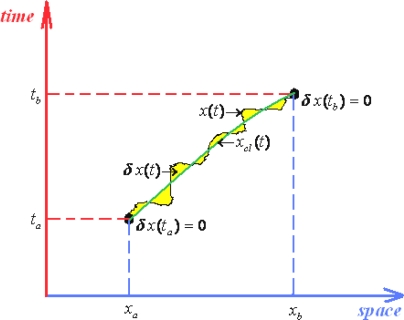
Illustration of the quantum fluctuations *δx*(*t*) around the classical path *x_cl_* (*t*) producing the space-time evolution of Figure 1.

Very important, note that the quantum fluctuation vanishes at the end-points of the evolution path since “meeting” with the classical (observed) path, see Figure 2

(145c) $$\delta x(t_{a})=0=\delta x(t_{b})$$

being these constraints known as the *Dirichlet boundary conditions*.

Now, one aims to separate the classical by the quantum fluctuation contributions also in the path integral propagator. Fortunately, this is possible for enough large class of potentials, more precisely for quadratic Lagrangeans of general type

(146) $$L(x,\dot{x},t)=\alpha (t)x^{2}+\beta (t)x\dot{x}+\gamma (t){\dot{x}}^{2}+\lambda (t)x+\chi (t)\dot{x}+\sigma (t).$$

Actually, expanding the path integral action (118) around the classical path requires the expansion of its associate Lagrangean (146); so we get accordingly

(147a) $$\begin{array}{c} L(x,\dot{x},t)=L(x_{cl},{\dot{x}}_{cl},t)\\ +{\left[\frac{\partial L}{\partial x}\delta x+\frac{\partial L}{\partial \dot{x}}\delta \dot{x}\right]}_{x_{cl},{\dot{x}}_{cl}}+\frac{1}{2}{\left[\frac{\partial^{2}L}{\partial x^{2}}\delta x+2\frac{\partial^{2}L}{\partial x\partial \dot{x}}\delta x\delta \dot{x}+\frac{\partial^{2}L}{\partial {\dot{x}}^{2}}\delta {\dot{x}}^{2}\right]}_{x_{cl},{\dot{x}}_{cl}} \end{array}$$

(147b) $$\begin{array}{c} S[x,\dot{x},t]=\int_{t_{a}}^{t_{b}}L(x,\dot{x},t)dt\\ =\int_{t_{a}}^{t_{b}}L(x_{cl},{\dot{x}}_{cl},t)dt+\int_{t_{a}}^{t_{b}}{\left[\frac{\partial L}{\partial x}\delta x+\frac{\partial L}{\partial \dot{x}}\delta \dot{x}\right]}_{x_{cl},{\dot{x}}_{cl}}dt+\int_{t_{a}}^{t_{b}}[\alpha (t)\delta x+\beta (t)\delta x\delta \dot{x}+\gamma (t)\delta {\dot{x}}^{2}]dt\\ =S_{cl}[x_{cl},{\dot{x}}_{cl},t]+\underset{\begin{array}{c} =0\\ \delta x(t_{a})=0=\delta x(t_{b}) \end{array}}{\underset{︸}{{\frac{\partial L}{\partial \dot{x}}\delta x|}_{t_{a}}^{t_{b}}}}+\int_{t_{a}}^{t_{b}}\underset{\begin{array}{c} =0\\ Euler\;-Lagrange \end{array}}{\underset{︸}{\left(\frac{\partial L}{\partial x}-\frac{d}{dt}\frac{\partial L}{\partial \dot{x}}\right)}}\delta xdt+\int_{t_{a}}^{t_{b}}[\alpha (t)\delta x+\beta (t)y\delta \dot{x}+\gamma (t)\delta {\dot{x}}^{2}]dt\\ S_{cl}[x_{cl},{\dot{x}}_{cl},t]+\int_{t_{a}}^{t_{b}}[\alpha (t)\delta x+\beta (t)y\delta \dot{x}+\gamma (t)\delta {\dot{x}}^{2}]dt \end{array}$$

With the action (147b) one observes it practically *separates* into the classical and quantum fluctuation contributions; this has two major consequences:

○ The classical action goes outside of the path integration by simply becoming the multiplication factor exp[(*i* / ħ)*S_cl_*];
○ Since the remaining contribution since depends only on quantum fluctuation *δx*(*t*) it allows the changing of the integration measure
  (148) $$\int_{x(t_{a})=x_{a}}^{x(t_{b})=x_{b}}D^{\prime }x(t)\to \int_{\delta x(t_{a})=0}^{\delta x(t_{b})=0}\underset{eq.(145a)}{\underset{=1}{\underset{︸}{\left|\frac{\delta x(t)}{\delta (\delta x(t))}\right|}}}D^{\prime }\delta x(t)=\int_{\delta x(t_{a})=0}^{\delta x(t_{b})=0}D^{\prime }\delta x(t)$$
  In these circumstances the path integral propagator factorizes as
  (149) $$(x_{b},t_{b};x_{a},t_{a})=\underset{\begin{array}{l} classical\\ contribution \end{array}}{\underset{︸}{\text{exp}\left\{\frac{i}{\hbar }S_{cl}[x_{cl},{\dot{x}}_{cl},t]\right\}}}\underset{quantum\;fluctutaions}{\underset{︸}{\int_{\delta x(t_{a})=0}^{\delta x(t_{b})=0}D^{\prime }\delta x(t)\text{exp}\left\{\frac{i}{\hbar }\int_{t_{a}}^{t_{b}}\left[\alpha (t)\delta x+\beta (t)y\delta \dot{x}+\gamma \delta \dot{x}+\gamma (t)\delta {\dot{x}}^{2}\right]dt\right\}}}$$

Few conceptual comments are now compulsory based on the path integral form (149):

○ It is clear that the quantum fluctuation term does not depend on ending space coordinates but only on their time coordinates, so that in the end will depend only on the time difference (*t_b_* –*t_a_*) since by means of energy conservation all the quantum fluctuation is a time-translation invariant, see for instance the Hamilton-Jacobi Equation (126); therefore it may be further resumed under the *fluctuation factor*
  (150) $$F(t_{b}-t_{a})\equiv \int_{\delta x(t_{a})=0}^{\delta x(t_{b})=0}D^{\prime }\delta x(t)\text{exp}\left\{\frac{i}{\hbar }\int_{t_{a}}^{t_{b}}[\alpha (t)\delta x+\beta (t)y\delta \dot{x}+\gamma (t)\delta {\dot{x}}^{2}]dt\right\}$$
○ Looking at the terms appearing in the whole Lagrangean (146) and to those present on the factor (150) it seems that once the last is known for a given Lagrangean, say *L*, then the same is characterizing also the modified one with the terms that are not present in the forms (150), namely
  (151) $$\tilde{L}=L+\lambda (t)x+\chi (t)\dot{x}+\sigma (t)$$
○ The resulting working path integral of the propagator now simply reads
  (152) $$(x_{b},t_{b};x_{a},t_{a})=F(t_{b}-t_{a})\text{exp}\left\{\frac{i}{\hbar }S_{cl}[x_{cl},{\dot{x}}_{cl},t]\right\}$$
  and gives intuitive inside of what path integral formalism of quantum mechanics really does: corrects the classical paths by the *quantum fluctuations resumed as the amplitude of the (semi) classical wave*.

Next, the big challenge is to compute the above fluctuation factor (150); here there are two possible approaches. One is considering the fluctuations as a Fourier series expansion so that directly (although through enough involving procedure) solving the multiple integrals appearing in (150). This route was originally proposed by Feynman in his quantum mechanically devoted monograph [41], and recently refined by Kleinert in an extended textbook [39].

The second way is trickier, although with limitations, but it avoids performing the direct integration prescribed by (150), while being instructive since computing the quantum fluctuation again in terms of classical path action [65], however through employing the present first two propagator properties, the group property (141) and the delta-Dirac limit (143), upon the quantum wave (152).

As such, combining the stipulated propagator properties, one starts by equivalently writing

(153) $$\begin{array}{c} \delta (x_{b}-x_{a})=(x_{a},t;x_{a},t)\\ =\int \left(x_{b},t;x,0)(x,0;x_{a},t\right)dx=\int (x_{b},t;x,0){\left(x_{a},t;x,0\right)}^{*}dx \end{array}$$

where the last identity follows since using the identity between the retarded (+) and advanced (–) Green functions [64]

(154) $$G^{+}(x_{b},t_{b};x_{a},t_{a})={\left[G^{-}(x_{a},t_{a};x_{b},t_{b})\right]}^{*}$$

combined with the propagator-Green function relationship (130), while supplemented with the advanced propagator version

(155) $$\left(x_{a},t_{a};x_{b},t_{b})=-iG^{-}(x_{a},t_{a};x_{b},t_{b}\right)$$

Now, the propagators from (153) may be written immediately under the general form (152)

(156a) $$(x_{b},t;x,0)=F(t)\text{exp}\left\{\frac{i}{\hbar }S_{cl}(x_{b},t;x,0)\right\}$$

(156b) $${\left(x_{a},t;x,0\right)}^{*}=F^{*}(t)\text{exp}\left\{\frac{i}{\hbar }S_{cl}(x_{a},t;x,0)\right\}$$

contributing in rewriting (153) as

(157) $$\begin{array}{c} \delta (x_{b}-x_{a})=\int dx{\left|F(t)\right|}^{2}\text{exp}\left\{\frac{i}{\hbar }[S_{cl}(x_{b},t;x,0)-S_{cl}(x_{a},t;x,0)]\right\}\\ \overset{x_{b}=x_{a}+\Delta x}{\underset{\Delta x=x_{b}-x_{a}}{=}}{\left|F(t)\right|}^{2}\int dx\text{exp}\left\{\frac{i}{\hbar }[S_{cl}(x_{a}+\Delta x,t;x,0)-S_{cl}(x_{a},t;x,0)]\right\}\\ ={\left|F(t)\right|}^{2}\int dx\text{exp}\left\{\frac{i}{\hbar }\frac{\partial S_{cl}(x_{a},t;x,0)}{\partial x_{a}}\Delta x\right\}\\ ={\left|F(t)\right|}^{2}\int dx\text{exp}\left\{\frac{i}{\hbar }\frac{\partial S_{cl}(x_{a},t;x,0)}{\partial x_{a}}(x_{b}-x_{a})\right\} \end{array}$$

Next, assuming the notation

(158) $$s(x)\equiv \frac{\partial S_{cl}(x_{a},t;x,0)}{\partial x_{a}}$$

in the case its derivative *ds*(*x*) / *dx* is independent of *x* - then it goes out the integral (157) with the *x*-variable changed to *s*, leaving with the identity

(159) $$\delta (x_{b}-x_{a})=\frac{2\pi \hbar {\left|F(t)\right|}^{2}}{\left|ds/dx\right|}\underset{\delta (x_{b}-x_{a})}{\underset{︸}{\int \frac{ds}{2\pi \hbar }\text{exp}\left\{\frac{i}{\hbar }(x_{b}-x_{a})s\right\}}}$$

from where the quantum fluctuation factor immediately follows with the analytical general form

(160) $$F(t)=\sqrt{\frac{1}{2\pi \hbar }\left|\frac{ds}{dx}\right|}\underset{eq.(158)}{=}\frac{1}{\sqrt{2\pi \hbar }}{\left|\frac{\partial^{2}S_{cl}(x_{a},t;x,0)}{\partial x\partial x_{a}}\right|}^{1/2}.$$

With expression (160) the propagator (152) is fully expressed in terms of classical action as

(161a) $$(x_{b},t_{b};x_{a},t_{a})=\frac{1}{\sqrt{2\pi \hbar }}{\left|\frac{\partial^{2}S_{cl}(x_{a},t;x,0)}{\partial x\partial x_{a}}\right|}^{1/2}\text{exp}\left\{\frac{i}{\hbar }S_{cl}(x_{b},t_{b};x_{a},t_{a})\right\},t=t_{b}-t_{a}$$

or in the more appealing form

(161b) $$(x_{a},t_{b};x_{a},t_{a})=\frac{1}{\sqrt{2\pi \hbar }}{\left|\frac{\partial^{2}S_{cl}(x_{b},t_{b};x_{a},t_{b})}{\partial x_{b}\partial x_{a}}\right|}^{1/2}\text{exp}\left\{\frac{i}{\hbar }S_{cl}(x_{b},t_{b};x_{a},t_{a})\right\}$$

usually referred to as the *Van Vleck-Pauli-Morette formula*, emphasizing on the importance of solving the classical problem for a given canonical Lagrangean [60,66].

However, the path integral solution (161b) has to be used with two amendments:

○ the procedure is valid only when the quantity (158), here rewritten in the spirit of (161b) as ∂*S_cl_* (*x_b_*, *t_b_*; *x_a_*, *t_a_*)/∂*x_a_*, performed respecting one end-point coordinate remains linear in the other space (end-point) coordinate *x_b_*, so that the identity (159) holds; this is true for the quadratic Lagrangeans of type (146) but not when higher orders are involved, when the previously stipulated Fourier analysis has to be undertaken (one such case will be in foregoing sections presented).
○ In the case the formula (161b) is applicable, *i.e.*, when previous condition are fulfilled, the obtained result has to be still verified in recovering the delta-Dirac function by the limit
  (162) $$\underset{t_{b}\to t_{a}}{\text{lim}}(x_{b},t_{b},x_{a},t_{a})=\delta (x_{b}-x_{a})$$
  in accordance with the implemented recipe, see Equation (153); usually this step is providing additional phase correction to the solution (161b).

The present algorithm is in next exemplified on two paradigmatic quantum problems: the free motion and the motion under harmonic oscillator influence. In each case the knowledge of the classical action will almost solve the entire path integral problem.

#### 3.3.2. Path Integral for Free Particle

Given a free particle with the Lagrangean

(163) $$L_{\left(0\right)}(x,\dot{x},t)=\frac{m}{2}{\dot{x}}^{2}$$

it leads by means of Euler-Lagrange equation

(164) $$\frac{d}{dt}\left(\frac{\partial L}{\partial \dot{x}}\right)=\frac{\partial L}{\partial x}$$

to the classical (Newtonian) motion

(165a) $${\ddot{x}}_{cl}(t)=0$$

with the obvious solution

(165b) $$x_{cl}(t)=x_{a}+\frac{x_{b}-x_{a}}{t_{b}-t_{a}}(t-t_{a})$$

fulfilling the boundary conditions

(165c) $$x_{cl}(t_{a})=x_{a}$$

(165d) $$x_{cl}(t_{b})=x_{b}$$

being these endpoints the states where the system is observable, *i.e.*, where the quantum fluctuations vanishes, see Equation (145c) and Figure 2.

Replacing solution (165b) back in Lagrangean (163) the classical action is immediately found

(166) $$S_{(0)cl}(x_{b},t_{b};x_{a},t_{a})=\int_{t_{a}}^{t_{b}}L_{\left(0\right)}(x_{cl},{\dot{x}}_{cl},t)dt=\frac{m}{2}\int_{t_{a}}^{t_{b}}{\dot{x}}_{cl}^{2}(t)dt=\frac{m}{2}\frac{{\left(x_{b}-x_{a}\right)}^{2}}{t_{b}-t_{a}}$$

Next, the quantity (158) is firstly evaluated in the spirit of (161b) as

(167) $$s_{\left(0\right)}(x)=\frac{\partial S_{(0)cl}(x_{b},t_{b};x_{a},t_{a})}{\partial x_{a}}=\frac{m}{t_{b}-t_{a}}(x_{b}-x_{a})$$

and recognized as linear in the other end-point space coordinate *x_b_*. Thus, the formula (161b) may be applied, with the actual yield

(168) $${\left(x_{b},\;t_{b};x_{a},t_{a}\right)}_{\left(0\right)}\;=\;\sqrt{\frac{m}{2\pi \hbar \left(t_{b\;}\;-\;t_{a}\right)}}\text{exp}\left\{\frac{i}{\hbar }\;\frac{m}{2}\;\frac{{\left(x_{b}-\;x_{a}\right)}^{2}}{t_{b\;}\;-\;t_{a}}\right\}$$

Finally, the result (168) has to be arranged so that to satisfy the limit (162) as well. For that we use the delta-Dirac representation

(169) $$\delta \left(x_{b}\;-\;x_{a}\right)\;=\;\frac{1}{\sqrt{\pi }}\underset{T\to 0}{\text{lim}}\left\{\frac{1}{\sqrt{\pi }}\text{exp}\left[-\frac{{\left(x_{b\;}-\;x_{a}\right)}^{2}}{T}\right]\right\}$$

Comparison between (168) and (169) leads with identification

(170) $$\frac{1}{T}\;=\;-\frac{im}{2\hbar \left(t_{b}\;-\;t_{a}\right)}\;=\;\frac{m}{2i\hbar \left(t_{b}\;-\;t_{b}\right)}$$

thus correcting the factor of (168) towards the correct limiting path integral solution

(171) $${\left(x_{b},\;t_{b};\;x_{a},\;t_{a}\right)}_{\left(0\right)}\;=\;\sqrt{\frac{m}{2\pi i\hbar \left(t_{b}\;-\;t_{a}\right)}}\text{exp}\left\{\frac{i}{\hbar }\;\frac{m}{2}\;\frac{{\left(x_{b}\;-\;x_{a}\right)}^{2}}{t_{b}\;-\;t_{a}}\right\}$$

Remarkably, this solution is indeed identical with the Green function of the free particle, up to the complex factor of (130), thus confirming the reliability of the path integral approach. Moreover, beside of its foreground character in quantum mechanics, the present path integral of the free particle can be further used in regaining the energy quantification of free electrons in solid state (motion within the infinite high box) as well as the Bohr quantification for the continuous deformation of the path on the circle [39,65].

Yet, these cases appeal the spectral representation of the quantum propagators and will not be treated here, being more suited for a dedicated monograph [67].

#### 3.3.3. Path Integral for Harmonic Oscillator

The characteristic Lagrangean of the harmonic oscillator

(172) $$L_{\left(\omega \right)}\;\left(x,\;\dot{x},\;t\right)\;=\;\frac{m}{2}\;{\dot{x}}^{2}\;-\;\frac{m}{2}\;\omega^{2\;}\;x^{2}$$

provides, when considered in the Euler-Lagrange equation (164), the classical equation of motion

(173a) $${\ddot{x}}_{cl}\;(t)\;+\;\omega^{2\;}x_{cl}\;(t)\;=\;0$$

with the well known solution

(173b) $$x_{cl\;}(t)\;=\;C\;\text{sin}\left(\omega t\;+\;\phi \right)$$

specialized for the end-point events of motion as

(174a) $$x_{a}\;=\;x_{cl}\;\left(t_{a}\right)\;=\;C\text{sin}\;\left(\omega t_{a}\;+\;\phi \right)$$

(174b) $$x_{b}\;=\;x_{cl}\left(t_{b}\right)\;=\;C\;\text{sin}\left(\omega t_{b}\;+\;\phi \right)$$

In the same way as done for the free motion, see solution (165b), worth rewritten the actual classical solution (173b) in terms of relations (174), for instance as

(175a) $$\begin{array}{c} x_{cl}(t)\;=\;C\text{sin}\left[\omega \left(t\;-\;t_{a}\right)\;+\;\left(\omega t_{a}\;+\;\phi \right)\right]\\ =\;\underset{{\dot{x}}_{a}\;/\omega }{\underset{︸}{C\;\text{cos}\left(\omega t_{a\;}\;+\;\phi \right)}}\text{sin}\left[\omega \left(t\;-\;t_{a}\right)\right]\;+\;\underset{x_{a}}{\underset{︸}{C\;\text{sin}\left(\omega t_{a\;}\;+\phi \right)}}\text{cos}\left[\omega \left(t\;-\;t_{a}\right)\right]\\ =\;\frac{1}{\omega }\;{\dot{x}}_{a\;}\;\text{sin}\left[\omega \left(t\;-\;t_{a}\right)\right]\;+\;x_{a\;}\text{cos}\left[\omega \left(t\;-\;t_{a}\right)\right] \end{array}$$

or similarly as:

(175b) $$x_{cl}(t)\;=\;\frac{1}{\omega }{\dot{x}}_{b\;}\;\text{sin}\left[\omega \left(t\;-\;t_{b}\right)\right]\;+\;x_{b\;}\;\text{cos}\;\left[\omega \left(t\;-\;t_{b}\right)\right]$$

On the other hand the classical action of the Lagrangean (172) looks like

(176) S(ω)cl (xb, tb; xa, ta) = ∫tatbL(ω)(xcl, x˙cl, t)dt = m2 ∫tatb[(dxcldt)(dxcldt) − ω2xcl2]dt= m2 ∫tatb[ddt(xclx˙cl) − xclx¨cl  − ω2xcl2]dt = [m2 xcl(t)x˙cl(t)]tatb −m2 ∫tatb(x¨cl + ω2xcl)︸=0eq.  (173a)xcldt= m2 [xb(tb)x˙b(tb) − xa(ta)x˙a(ta)]

Now, in order to have classical action in terms of only space-time coordinate of the ending points, one has to replace the end-point velocities in (176) by the aid of relations (175a) and (175b) in which the current time is taken as the *t* = *t_b_* and *t* = *t_a_*, respectively; thus we firstly get

(177a) $${\dot{x}}_{a}\;=\;\frac{\omega }{\text{sin}\left[\omega \left(t_{b}\;-\;t_{a}\right)\right]}\left\{x_{b}\;-\;x_{a}\;\text{cos}\left[\omega \left(t_{b}\;-\;t_{a}\right)\right]\right\}$$

(177b) $${\dot{x}}_{b}\;=\;\frac{\omega }{\text{sin}\left[\omega \left(t_{b}\;-\;t_{a}\right)\right]}\left\{-\;x_{a}\;+\;x_{b}\;\text{cos}\left[\omega \left(t_{b}\;-\;t_{a}\right)\right]\right\}$$

then we form the working products

(178a) $$x_{b}{\dot{x}}_{b}\;=\;\frac{\omega }{\text{sin}\left[\omega \left(t_{b}\;-\;t_{a}\right)\right]}\left\{x_{b}^{2}\;\text{cos}\left[\omega \left(t_{b}\;-\;t_{a}\right)\right]\;-\;x_{a}x_{b}\right\}$$

(178b) $$x_{a}{\dot{x}}_{a}\;=\;\frac{\omega }{\text{sin}\left[\omega \left(t_{b}\;-\;t_{a}\right)\right]}\left\{-\;x_{a}^{2}\text{cos}\left[\omega \left(t_{b}\;-\;t_{a}\right)\right]+x_{a}x_{b}\right\}\;\;$$

to finally replace them in expression (176) to obtain the computed classical action

(179) $$S_{(\omega )cl}\left(x_{b},\;t_{b};\;x_{a},t_{a}\right)\;=\;\frac{m\omega }{2\;\text{sin}\left[\omega \left(t_{b}\;-\;t_{a}\right)\right]}\;\left\{\left(x_{a\;}^{2}\;+\;x_{b}^{2}\right)\text{cos}\left[\omega \left(t_{b}\;-\;t_{a}\right)\right]-2x_{a}x_{b}\right\}$$

Note that the correctness of Equation (179) may also be checked by imposing the limit *ω →* 0 in which case the previous free motion has to be recovered; indeed by employing the consecrated limit

(180) $$\underset{y\to 0}{\text{lim}}\;\frac{\text{sin}y}{y}\;=\;1$$

one immediately gets

(181) $$\begin{array}{c} \underset{\omega \to 0}{\text{lim}}S_{(\omega )cl}\left(x_{b},t_{b};\;x_{a},\;t_{a}\right)\\ =\;\frac{m}{2\left(t_{b}-\;t_{a}\right)}\underset{=1}{\underset{︸}{\underset{\omega \to 0}{\text{lim}}\frac{\omega \left(t_{b}\;-\;t_{a}\right)}{\text{sin}\left[\omega \left(t_{b}\;-\;t_{a}\right)\right]}}}\;\underset{{\left(x_{b}-x_{a}\right)}^{2}}{\underset{︸}{\underset{\omega \to 0}{\text{lim}}\left\{\left(x_{a}^{2}+\;x_{b}^{2}\right)\text{cos}\left[\omega \left(t_{b}\;-\;t_{a}\right)\right]\;-\;2x_{a}x_{b}\right\}}}\\ =\;\frac{m}{2}\;\frac{{\left(x_{b}\;-\;x_{a}\right)}^{2}}{t_{b}\;-\;t_{a}}\\ =\;S_{(0)cl\;}\left(x_{b},\;t_{b};\;x_{a},\;t_{a}\right) \end{array}$$

Such a kind of check is most useful and has to hold also for the quantum propagator as a whole. Going to determine it one has to reconsider the classical action (179) so that the quantity (158) is directly evaluated in the spirit of (161b) as

(182) $$\frac{\partial S_{(\omega )cl\;}\left(x_{b},\;t_{b};\;x_{a},\;t_{a}\right)}{\partial x_{a}}\;=\;\frac{m\omega }{\text{sin}\;\left[\omega \left(t_{b}\;-\;t_{a}\right)\right]}\left\{x_{a}\;\text{cos}\left[\omega \left(t_{b}\;-\;t_{a}\right)\right]-x_{b}\right\}$$

thus again encountering the linearity case in the other end-point coordinate *x_b_*. Being this the fortunate situation in which the previous expression (161b) for path integral computation may be applied with the harmonic oscillator result

(183) $$\left(x_{b},\;t_{b};\;x_{a},\;t_{a}\right)\;=\;\sqrt{\frac{m\omega }{2\pi \hbar \;\text{sin}\left[\omega \left(t_{b}\;-\;t_{a}\right)\right]}}\text{exp}\left\{\frac{i}{\hbar }\;\frac{m\omega }{2\;\text{sin}\left[\omega \left(t_{b}\;-\;t_{a}\right)\right]}\left\{\left(x_{a}^{2}\;+\;x_{b}^{2}\right)\text{cos}\left[\omega \left(t_{b}\;-\;t_{a}\right)\right]\;-\;2x_{a}x_{b}\right\}\right\}$$

Yet, as above was the case for the classical action itself, also the pre-exponential quantum fluctuation factor of (183a) has to overlap with that appearing in the path integral of free motion of Equation (171) for the limit *ω →* 0

(184) $$\underset{\omega \to 0}{\text{lim}}\;\sqrt{\frac{m\omega }{2\pi \hbar \;\text{sin}\;\left[\omega \left(t_{b}\;-\;t_{a}\right)\right]}}\;\underset{eq.\;(180)\;}{=}\sqrt{\frac{m}{2\pi \hbar \left(t_{b}\;-\;t_{a}\right)}}$$

Thus we have to adjust the propagator (183) with the exponential pre-factor corrected with the complex factor “*i*”

(185) $${\left(x_{b},\;t_{b};\;x_{a},\;t_{a}\right)}_{\left(\omega \right)}\;=\;\sqrt{\frac{m\omega }{2\pi i\hbar \;\text{sin}\left[\omega \left(t_{b}\;-\;t_{a}\right)\right]}}\text{exp}\left\{\frac{i}{\hbar }\;\frac{m\omega }{2\;\text{sin}\left[\omega \left(t_{b}\;-\;t_{a}\right)\right]}\left\{\left(x_{a}^{2}\;+\;x_{b}^{2}\right)\text{cos}\left[\omega \left(t_{b}\;-\;t_{a}\right)\right]\;-\;2x_{a}x_{b}\right\}\right\}$$

This is the sought propagator of the (electronic) motion under the harmonic oscillating potential, computed by means of path integral; it provides the canonical density to be implemented in the DFT algorithm (128), (129)

(186) $$\rho_{⊗}\left(x,t_{b}\;-\;t_{a}\right)\;=\;{\left(x,\;t_{b\;};\;x,\;t_{a}\right)}_{\left(\omega \right)}\;=\;\sqrt{\frac{m\omega }{2\pi i\hbar \;\text{sin}\left[\omega \;\left(t_{b}\;-\;t_{a}\right)\right]}}\text{exp}\left\{\frac{i}{\hbar }\;\frac{m\omega \left(\text{cos}\left[\omega \left(t_{b}\;-\;t_{a}\right)\right]-1\right)}{\text{sin}\left[\omega \left(t_{b}\;-t_{a}\right)\right]}x^{2}\right\}$$

Yet, for practical implementations, the passage from quantum mechanics (QM) to quantum statistics (QS) is to be considered based on the Wick transformation (10) here rewritten as

(187) $$\left(t_{b}\;-\;t_{a}\right)\to \;-i\hbar \beta \;\equiv \;-i\left(\tau_{b}\;-\;\tau_{a}\right)$$

providing the Euler trigonometric to hyperbolic function conversions (by analytical continuations)

(188a) $$\text{sin}\omega \left(t_{b}\;-\;t_{a}\right)\;=\;\frac{1}{i}\;\frac{e^{i\omega \left(t_{b\;}-\;t_{a}\right)}\;-e^{-i\omega \left(t_{b}\;-\;t_{a}\right)}}{2}\;\to \;\frac{1}{i}\;\frac{e^{\omega \left(\tau_{b}\;-\;\tau_{a}\right)}\;-e^{-\omega \left(\tau_{b}\;-\;\tau_{a}\right)}}{2}\;=\;\frac{1}{i}\text{sinh}\;\omega \left(\tau_{b}\;-\;\tau_{a}\right)$$

(188b) $$\text{cos}\omega \left(t_{b}\;-\;t_{a}\right)\;=\;\;\frac{e^{i\omega \left(t_{b\;}-\;t_{a}\right)}\;+e^{-i\omega \left(t_{b}\;-\;t_{a}\right)}}{2}\;\to \;\;\frac{e^{\omega \left(\tau_{b}\;-\;\tau_{a}\right)}\;+e^{-\omega \left(\tau_{b}\;-\;\tau_{a}\right)}}{2}\;=\;\text{cosh}\;\omega \left(\tau_{b}\;-\;\tau_{a}\right)$$

finally displaying for density (186) the counterpart formulation

(189) $$\rho_{⊗}\left(x,\tau_{b}\;-\;\tau_{a}\right)\;=\;\sqrt{\frac{m\omega }{2\pi \hbar \text{sinh}\left[\omega \left(\tau_{b}-\tau_{a}\right)\right]}}\text{exp}\left\{-\frac{1}{\hbar }\;\frac{m\omega \left(\text{cosh}\left[\omega \left(\tau_{b}-\tau_{a}\right)\right]-1\right)}{\text{sinh}\left[\omega \left(\tau_{b}-\tau_{a}\right)\right]}x^{2}\right\}$$

The uni-particle (electronic) density (189) is then used for computing the harmonic oscillator partition function

(190) $$\begin{array}{c} Z_{\omega }\;=\;\int_{-\infty }^{+\infty }\rho_{⊗}\left(x,\tau_{b}-\tau_{a}\right)dx\\ =\;\sqrt{\frac{m\omega }{2\pi \hbar \text{sinh}\left[\omega \left(\tau_{b}-\tau_{a}\right)\right]}}\int_{-\infty }^{+\infty }\text{exp}\;\left\{-\frac{1}{\hbar }\frac{m\omega \left(\text{cosh}\left[\omega \left(\tau_{b}-\tau_{a}\right)\right]-1\right)}{\text{sinh}\left[\omega \left(\tau_{b}-\tau_{a}\right)\right]}x^{2}\right\}dx\\ =\;\sqrt{\frac{m\omega }{2\pi \hbar \;\text{sinh}\;\left[\omega \left(\tau_{b}-\tau_{a}\right)\right]}}\;\sqrt{\frac{\pi \hbar \;\text{sinh}\left[\omega \left(\tau_{b}-\tau_{a}\right)\right]}{m\omega \left(\text{cosh}\left[\omega \left(\tau_{b}-\tau_{a}\right)\right]-1\right)}}\\ =\;\sqrt{\frac{1}{2\left(\text{cosh}\left[\omega \left(\tau_{b}-\tau_{a}\right)\right]-1\right)}} \end{array}$$

Now, using the “double angle” formula

(191) $$\text{cosh}\;2y\;=\;{\text{cosh}}^{2}\;y\;+{\text{sinh}}^{2}y=2{\text{cosh}}^{2}\;y-1=2{\text{sinh}}^{2}y+1$$

the partition function (190) further becomes

(192) $$Z_{\omega }=\frac{1}{2\text{sinh}\left[\frac{\omega \left(\tau_{b}-\tau_{a}\right)}{2}\right]}\underset{eq.\;(187)}{=}\;\frac{1}{2\text{sinh}\left[\frac{\omega \hbar \beta }{2}\right]}$$

Remarkably, the result (192) recovers also the energy quantification of the quantum motion under the harmonic oscillator influence, as seen by the successive transformations

(193a) $$\begin{array}{c} Z_{\omega }=\frac{1}{\text{exp}\left(\omega \hbar \beta /2\right)-\text{exp}\left(-\omega \hbar \beta /2\right)}=\text{exp}\left(-\omega \hbar \beta /2\right)\;\frac{1}{1-\text{exp}\left(-\omega \hbar \beta \right)}\\ =\;\text{exp}\left(-\omega \hbar \beta /2\right)\sum_{n=0}^{\infty }{\left[\text{exp}\left(-\omega \hbar \beta \right)\right]}^{n}=\text{exp}\left(-\omega \hbar \beta /2\right)\sum_{n=0}^{\infty }\left[\text{exp}\left(-n\omega \hbar \beta \right)\right]\\ =\;\sum_{n=0}^{\infty }\text{exp}\left[-\beta \hbar \omega \left(n+\frac{1}{2}\right)\right] \end{array}$$

When comparing the expression (193a) with the canonical formulation of the partition function

(193b) $$Z\equiv \sum_{n=0}^{\infty }\text{exp}\left[-\beta E_{n}\right]$$

there follows immediately the harmonic oscillator energy quantification

(194) $$E_{n}(\omega )=\hbar \omega \left(n+\frac{1}{2}\right)$$

in perfect agreement with the consecrated expression.

The results of these two sections suggest the following rules for using path integrals propagator for density computations:

○ The reliable application of the density computation upon the partition function algorithm, see Equations (128) and (129), prescribes the transformation of the obtained quantum result to the quantum statistical counterpart by means of Wick transformation (10), while supplemented by the functions (188) conversions;
○ In computation of the path integral propagator the workable *Van Vleck-Pauli-Morette formula* looks like
  (195) $$\left(x_{b},\;t_{b};\;x_{a},\;t_{a}\right)\;=\sqrt{\frac{1}{2\pi i\hbar }\left|\frac{\partial^{2}S_{cl}\left(x_{b},t_{b};x_{a},t_{a}\right)}{\partial x_{b}\partial x_{a}}\right|}\text{exp}\left\{\frac{i}{\hbar }S_{cl}\left(x_{b},t_{b};x_{a},t_{a}\right)\right\}$$
  with the complex factor “*i*” included, as confirmed by both the free and harmonic oscillator quantum motions; it may be used for linear classical actions in one of the end-point space coordinates upon derivation respecting the other one; yet the formula (195) should be always checked for fulfilling the limiting (162) delta-Dirac function for simultaneous events for any applied potential.

Nevertheless, recognizing the major role the classical action plays in the path integral representation of the quantum propagation (and propagator), the question whether it is possible to consider the semi-classical expansion of the propagator in general case, without being under any constraint except the semiclassical (higher temperatures) limit itself, naturally arises. Such an approach is exposed and its reliability tested in the next sections.

## 4. Semiclassical Path Integral of Evolution Amplitude

### 4.1. Semiclassical Expansion

Semiclassical derivation of the evolution amplitude employs some of the previously Feynman path integral ideas refined due to the works of Kleinert and collaborators [39,41,68–71]. They are bellow summarized.

○ The real time dependency is “rotated” into the imaginary time
  (196a) τ = it =ℏβ
  or in finite differences as
  (196b) $$\tau_{b}-\tau_{a}=\hbar \beta =i\left(t_{b}-t_{a}\right)$$
  according with the Wick transformation (10).
○ The quantum paths of (145a) are re-parameterized as
  (197) $$x\left(\tau \right)\;=\;\bar{x}\;+\eta \left(\tau \right)$$
  where the classical path of (145a) is replaced by the fixed (non time-dependent) average
  (198) $$x_{cl}\left(t\right)\;\to \bar{x}\;=\;\frac{x_{a}+\;x_{b}}{2}$$
  while the fluctuation path *η*(*τ*) remains to carry the whole path integral information, yet being departed at the end of integration frontier from previously Dirichlet boundary conditions (145c), where it vanished at the domain frontiers, to the actual different endpoint values
  (199a) $$\eta_{a}=\eta \left(\tau_{a}\right)=-\frac{\Delta x}{2}$$
  (199b) $$\eta_{b}=\eta \left(\tau_{b}\right)=+\frac{\Delta x}{2}$$
  in terms of the length of the “traveled” space
  (200) $$\Delta x=x_{b}-x_{a}$$

In these conditions the quantum statistical path integral representation of quantum propagator becomes

(201) $${\left(x_{b}\tau_{b};x_{a}\tau_{a}\right)}_{QS}=\int_{\eta \left(\tau_{a}\right)=-\Delta x/2}^{\eta \left(\tau_{b}\right)=+\Delta x/2}D\eta (\tau )\text{exp}\left\{-\frac{1}{\hbar }\int_{\tau_{a}}^{\tau_{b}}\left[\frac{m}{2}{\dot{\eta }}^{2}\left(\tau \right)+V\left(\bar{x}+\eta (\tau )\right)\right]d\tau \right\}$$

since we immediately noted the immediate transformations

(202a) $$D^{\prime }x\left(\tau \right)\to D\eta \left(\tau \right)$$

(202b) $${\dot{x}}^{2}\left(\tau \right)\to {\dot{\eta }}^{2}\left(\tau \right)$$

(202c) $$V\left(x(\tau )\right)\to V\left(\bar{x}+\eta \left(\tau \right)\right)$$

based on the above (197)–(200) parameterization.

It should be pointed out that the used re-parameterization is not modifying the value of the path integral but is intended to better visualize its properties, towards evaluating it. As such, from expression (201) it now appears clearer than before that for the systems governed by smooth potentials, the series expansion may be applied respecting the path fluctuation, here in the second order truncation

(203) $$V\left(\bar{x}+\eta (\tau )\right)≅V\left(\bar{x}\right)+\partial_{i}V\left(\bar{x}\right)\eta_{i}\left(\tau \right)+\frac{1}{2}\partial_{i}\partial_{j}V\left(\bar{x}\right)\eta_{i}\left(\tau \right)\eta_{j}\left(\tau \right)$$

where the covariant notation for products was assumed for maintaining the generality of the *D*-dimensioned approach. This way, a (truncated) series of path integral evolution amplitude of Equation (201) it is at once obtained

(204) $$\begin{array}{ll} {\left(x_{b}\tau_{b};x_{a}\tau_{a}\right)}_{QS} & =\int_{\eta \left(\tau_{a}\right)=-\Delta x/2}^{\eta \left(\tau_{b}\right)=+\Delta x/2}D\eta \left(\tau \right)e^{-\frac{1}{\hbar }\int_{\tau_{a}}^{\tau_{b}}\left[\frac{m}{2}{\dot{\eta }}^{2}(\tau )\right]d\tau }e^{-\frac{1}{\hbar }\int_{\tau_{a}}^{\tau_{b}}V\left(\bar{x}\right)d\tau }e^{-\frac{1}{\hbar }\int_{\tau_{a}}^{\tau_{b}}\left[\partial_{i}V(\bar{x})\eta_{i}(\tau )+\frac{1}{2}\partial_{i}\partial_{j}V(\bar{x})\eta_{i}(\tau )\eta_{j}(\tau )+\ldots \right]d\tau }\\ & =\text{exp}\left[-\frac{\tau_{b}-\tau_{a}}{\hbar }V\left(\bar{x}\right)\right]\int_{\eta \left(\tau_{a}\right)}^{\eta \left(\tau_{b}\right)}D\eta \left(\tau \right)\text{exp}\left\{-\frac{1}{\hbar }\int_{\tau_{a}}^{\tau_{b}}\left[\frac{m}{2}{\dot{\eta }}^{2}\left(\tau \right)\right]d\tau \right\}\\ & \times \left\{\begin{array}{c} 1-\frac{1}{\hbar }\int_{\tau_{a}}^{\tau_{b}}d\tau \left[\partial_{i}V\left(\bar{x}\right)\eta_{i}\left(\tau \right)+\frac{1}{2}\partial_{i}\partial_{j}V\left(\bar{x}\right)\eta_{i}\left(\tau \right)\eta_{j}\left(\tau \right)+\ldots \right]\\ +\frac{1}{2\hbar^{2}}\int_{\tau_{a}}^{\tau_{b}}d\tau \int_{\tau_{a}}^{\tau_{b}}d\tau^{\prime }\left[\partial_{i}V\left(\bar{x}\right)\partial_{j}V\left(\bar{x}\right)\eta_{i}\left(\tau \right)\eta_{j}\left(\tau^{\prime }\right)+\ldots \right]+\ldots \end{array}\right\} \end{array}$$

as being driven by the quantum fluctuation’ various orders contributions, up to the second order. This is a natural approach since the very quantum nature of the path integral is given by the quantum fluctuations themselves, from where the systematic approximations of path integrals over the quantum fluctuations. The series is known as the *semiclassical expansion* since is formally done in the “powers of *ħ*-Planck”.

Now, looking on Equation (204) as compared with the previously used quantum mechanical form of Equation (149) the present propagator representation would be resumed as

(205a) $${\left(x_{b}\tau_{b};x_{a}\tau_{a}\right)}_{QS}=\underset{\begin{array}{c} \text{CLASSICAL}\\ \text{FACTOR} \end{array}}{\underset{︸}{\text{exp}\left[-\beta V\left(\bar{x}\right)\right]}}\underset{\text{SEMI}-\text{CLASSICAL}\;\text{CONTRIBUTION}}{\underset{︸}{\int_{\eta \left(\tau_{a}\right)}^{\eta \left(\tau_{b}\right)}D\eta (\tau )F_{SC}[\eta ]\text{exp}\left\{-\frac{1}{\hbar }\int_{\tau_{a}}^{\tau_{b}}\left[\frac{m}{2}{\dot{\eta }}^{2}(\tau )\right]d\tau \right\}}}$$

where we have used (196b) along identifying the semiclassical factor *F_SC_*[*η*]. However, the expression (205a) may be further formally cast

(205b) $${\left(x_{b}\tau_{b};x_{a}\tau_{a}\right)}_{QS}=\text{exp}\left[-\beta V\left(\bar{x}\right)\right]{\left(\frac{\Delta x}{2}\tau_{b};-\frac{\Delta x}{2}\tau_{a}\right)}_{\left(0\right)}\langle F_{SC}[\eta ]\rangle$$

by introducing the so called *free* imaginary time amplitude

(206a) $${\left(\frac{\Delta x}{2}\tau_{b};-\frac{\Delta x}{2}\tau_{a}\right)}_{\left(0\right)}=\int_{\eta \left(\tau_{a}\right)=-\Delta x/2}^{\eta \left(\tau_{b}\right)=+\Delta x/2}D\eta (\tau )\text{exp}\left\{-\frac{1}{\hbar }\int_{\tau_{a}}^{\tau_{b}}\left[\frac{m}{2}{\dot{\eta }}^{2}(\tau )\right]d\tau \right\}$$

readily given by the free-propagator solution (171) accommodated by the present statistical and boundary transformations Equations (196b) and (199) to the form

(206b) $${\left(\frac{\Delta x}{2}\tau_{b};-\frac{\Delta x}{2}\tau_{a}\right)}_{\left(0\right)}=\sqrt{\frac{m}{2\pi \hbar^{2}\beta }}\text{exp}\left\{-\frac{m}{2\hbar^{2}\beta }{\left(\Delta x\right)}^{2}\right\}$$

while having the normalization role for averaging the semiclassical factor contribution

(207) $$\langle F_{SC}[\eta ]\rangle =\frac{\int_{\eta \left(\tau_{a}\right)=-\Delta x/2}^{\eta \left(\tau_{b}\right)=+\Delta x/2}D\eta (\tau )F_{SC}[\eta ]\text{exp}\left\{-\frac{1}{\hbar }\int_{\tau_{a}}^{\tau_{b}}\left[\frac{m}{2}{\dot{\eta }}^{2}(\tau )\right]d\tau \right\}}{{\left(\frac{\Delta x}{2}\tau_{b};-\frac{\Delta x}{2}\tau_{a}\right)}_{\left(0\right)}}$$

Therefore, the semiclassical form of path integral representation of evolution amplitude looks like

(208) $$\begin{array}{c} {\left(x_{b}\tau_{b};x_{a}\tau_{a}\right)}_{QS}=\sqrt{\frac{m}{2\pi \hbar^{2}\beta }}\text{exp}\left\{-\frac{m}{2\hbar^{2}\beta }{\left(\Delta x\right)}^{2}-\beta V\left(\bar{x}\right)\right\}\\ \times \left\{\begin{array}{c} 1-\frac{1}{\hbar }\partial_{i}V\left(\bar{x}\right)\int_{\tau_{a}}^{\tau_{b}}d\tau \langle \eta_{i}\left(\tau \right)\rangle -\frac{1}{2\hbar }\partial_{i}\partial_{j}V\left(\bar{x}\right)\int_{\tau_{a}}^{\tau_{b}}d\tau \langle \eta_{i}\left(\tau \right)\eta_{j}\left(\tau \right)\rangle \\ +\frac{1}{2\hbar^{2}}\partial_{i}V\left(\bar{x}\right)\partial_{j}V\left(\bar{x}\right)\int_{\tau_{a}}^{\tau_{b}}d\tau \int_{\tau_{a}}^{\tau_{b}}d\tau^{\prime }\langle \eta_{i}\left(\tau \right)\eta_{j}\left(\tau^{\prime }\right)\rangle +... \end{array}\right\} \end{array}$$

The remaining problem is that of expressing the averaged values of the fluctuation paths in single or multiple time connection, *i.e.*, 〈*η_i_*(*τ*)〉, 〈*η_i_*(*τ*)*η_j_*(*τ*)〉, 〈*η_i_*(*τ*)*η_j_*(*τ*')〉, etc.

From the heuristic point of view it is normal to arrive at the form (208) because it tells us that the quantum fluctuation is firstly averaged along the quantum evolution and then averaged by time in order that the evolution amplitude is determined. Observe also that the present semiclassical approach is not using the previously employed properties of the classical action, avoiding therefore the limitation of the derivative behavior at edge of the space domain of integration, while posing now the limitation in what respect the quantum fluctuation power. It is also useful to remark that the present semiclassical approach may use the *interplay* between the previous solved *free-and-harmonic quantum motions*, since the path integral (206a) may equally be regarded as the free motion of the quantum fluctuations (naturally since they are not known *a priori* or with some possibility of instantaneously observation); at the same time, if one formally counts the kinetic term as the perturbative (a.k.a. fluctuation) oscillatory motion

(209) $$\frac{m}{2}{\dot{\eta }}^{2}(\tau )=\frac{m}{2}{\left(\frac{d}{dt}\eta \right)}^{2}\approx \frac{m}{2}\underset{\partial_{t}}{\underset{︸}{\omega^{2}}}\eta^{2}$$

a more complex picture of quantum fluctuation is obtained; in conclusion, quantum fluctuating paths may be (or should be) treated as being a kind of *harmonically free* motion: *harmonic* since as fluctuations may be expanded in Fourier series (as originally perceived by Feynman), but also *free* since their unknown of instantaneous feature. Therefore, an appropriate use of both these manifestations will conduct to the reliable path integral representation. Yet, since we have already used the free-motion character of fluctuation paths, the harmonic one is next entering the analysis.

### 4.2. Connected Correlation Functions

For calculating the average of quantum fluctuation paths one has to understand their inner nature: in order reconciliation of free and harmonic features be achieved the so called *quantum current j*(*τ*) is introduced (and presumed to appear in reality too as causing/driving the quantum fluctuations), so that the propagator of this current, known as *the generating functional*, is formed [39,66]

(210) $$\left(\frac{\Delta x}{2}\tau_{b};-\frac{\Delta x}{2}\tau_{a}\right)[j(\tau )]=\int_{\eta (\tau_{a})=-\Delta x/2}^{\eta (\tau_{b})=+\Delta x/2}D\eta (\tau )\text{exp}\left\{-\frac{1}{\hbar }\int_{\tau_{a}}^{\tau_{b}}\left[\frac{m}{2}{\dot{\eta }}^{2}(\tau )-j(\tau )\eta (x)\right]d\tau \right\}$$

with which help one can recognized the equivalence

(211) $$\begin{array}{c} \langle \eta (\tau )\rangle =\frac{{\hbar \frac{\delta }{\delta j(\tau )}\left(\frac{\Delta x}{2}\tau_{b};\;-\;\frac{\Delta x}{2}\tau_{a}\right)\left[j(\tau )\right]|}_{j(\tau )=0}}{{\left(\frac{\Delta x}{2}\tau_{b};\;-\;\frac{\Delta x}{2}\tau_{a}\right)}_{\left(0\right)}}\\ =\;\frac{\int_{\eta (\tau_{a})=-\Delta x/2}^{\eta (\tau_{b})=+\Delta x/2}D\eta (\tau )[\eta (x)]\text{exp}\left\{-\frac{1}{\hbar }\int_{\tau_{a}}^{\tau_{b}}\left[\frac{m}{2}{\dot{\eta }}^{2}(\tau )-j(\tau )\eta (x)\right]d\tau \right\}}{{\left(\frac{\Delta x}{2}\tau_{b};\;-\;\frac{\Delta x}{2}\tau_{a}\right)}_{\left(0\right)}} \end{array}$$

in accordance with general definition (207). One can nevertheless see that the quantum current appearance in (211) is under the perturbation form, so that it readily accounts for the deviation from the free fluctuation motion towards the harmonically one. Therefore, although general correlation definition may be advanced by the ordering rule

(212) $$\langle \eta (\tau_{1})\cdots \eta (\tau_{n})\rangle =\frac{{\hbar \frac{\delta }{\delta j(\tau_{1})}\cdots \hbar \frac{\delta }{\delta j(\tau_{n})}\left(\frac{\Delta x}{2}\tau_{b};\;-\;\frac{\Delta x}{2}\tau_{a}\right)\left[j(\tau_{1}),\cdots ,j(\tau_{n})\right]|}_{\begin{array}{l} j(\tau_{1})=0,\\ ...\\ j(\tau_{n})=0 \end{array}}}{{\left(\frac{\Delta x}{2}\tau_{b};\;-\;\frac{\Delta x}{2}\tau_{a}\right)}_{\left(0\right)}}$$

the problem of practically evaluation still remains. Aiming for solving it one observes that the form (212) is analogous with the partition function based electronic density, see Equation (12) for instance; consequently, the alternative formulation looks at the canonical (*N* = 1, mono-particle) level like

(213) $$\langle \eta (\tau_{1})\cdots \eta (\tau_{n})\rangle ={\left\{Z^{-1}[j]\hbar \frac{\delta }{\delta j\left(\tau_{1}\right)}\cdots \hbar \frac{\delta }{\delta j\left(\tau_{n}\right)}Z[j]\right\}}_{j=0}$$

where, now, the quantity *Z*[*j*] plays the role of the generating functional of the quantum fluctuation correlation (or connection) average. Yet, the writing (213) may suffer from disconnecting character due to the presence of simple *Z*[*j*]; this may be better visualized when re-expressing (213) under the so called *n-point (correlation) functions*

(214a) $$\langle \eta (\tau_{1})\cdots \eta (\tau_{n})\rangle =Z^{-1}\int D\eta (\tau )[\eta (\tau_{1})\cdots \eta (\tau_{n})]\text{exp}\left\{-\frac{1}{\hbar }S_{+}\right\}$$

(214b) $$=\prod_{i=1}^{n}\left[\int_{-\infty }^{+\infty }d\eta \left(\tau_{i}\right)\right](\eta_{b}\tau_{b};\eta_{n}\tau_{n}).\eta (\tau_{n}).(\eta_{n}\tau_{n};\eta_{n-1}\tau_{n-1}).\eta (\tau_{n-1})\cdots \eta (\tau_{1})(\eta_{1}\tau_{1};\eta_{a}\tau_{a})$$

with *S*_+_ being the Euclidian action, see Equation (121c), while space and time slicing intervals are those introduced by (81) and (90), respectively.

The disconnected character of correlations (213)–(214) may be overcome remembering that the logarithm of the partition function provides the thermodynamic free energy, see relation (67), here under canonical (*N* = 1) form

(215) lnZ[j]=−βF[j]

which, as a measurable-observable energy, it compulsory contains the connected parts of *Z*[*j*](*i.e.*, energy’s pieces combines towards the total energy). Therefore, this leaves with the idea that through introducing another generating functional, namely

(216) W[j]=lnZ[j]

Equation (213) may be rewritten as the connected part of correlation 〈*η*(*τ*_1_)⋯*η*(*τ_n_*)〉 that it can be naturally identified with a sort of generalized n-points (events) Green function

(217) $$G_{con}^{\left(n\right)}\left(\tau_{1},\cdots ,\tau_{n}\right)={\left\{\frac{\delta }{\delta j(\tau_{1})}\cdots \frac{\delta }{\delta j(\tau_{n})}W\left[j\right]\right\}}_{j=0}$$

Nevertheless, aiming to have a better “feeling” on how the connected and disconnected correlation (fluctuation) functions (217) and (213) are linked, let’s start evaluating some orders of them.

As such, absorbing the constants in the involved functionals, the first order of (213) reads correlation of (213) we successively have

(218) $$\langle \eta \left(\tau \right)\rangle =Z^{-1}[j]\frac{\delta }{\delta j(\tau )}Z[j]=\frac{\delta }{\delta j(\tau )}W[j]=G_{con}^{\left(1\right)}(\tau )=\eta_{cl}(\tau )$$

as the single connected path (remember that the fluctuation was already averaged out) that is just the classical path connecting the ending points of the quantum evolution, see Figure 2.

Now, going to the second order of correlation of (213) one has

(219a) $$\begin{array}{c} \langle \eta (\tau_{1})\eta (\tau_{2})\rangle =Z^{-1}[j]\frac{\delta }{\delta j(\tau_{1})}\frac{\delta }{\delta j(\tau_{2})}Z[j]\\ =Z^{-1}[j]\frac{\delta }{\delta j(\tau_{1})}\left\{Z[j]\left(\frac{\delta }{\delta j(\tau_{2})}W[j]\right)\right\}\\ =\;\frac{\delta }{\delta j(\tau_{1})}\frac{\delta }{\delta j(\tau_{2})}W[j]+Z^{-1}[j]\frac{\delta Z[j]}{\delta j(\tau_{1})}\frac{\delta W[j]}{\delta j(\tau_{2})}\\ =\;\frac{\delta }{\delta j(\tau_{1})}\frac{\delta }{\delta j(\tau_{2})}W[j]+\frac{\delta W[j]}{\delta j(\tau_{1})}\frac{\delta W[j]}{\delta j(\tau_{2})} \end{array}$$

a result that can be wisely rearranged as

(219b) $$\begin{array}{ll} 〈\eta \left(\tau_{1}\right)\eta \left(\tau_{2}\right)〉\; & =\;G_{\text{con}}^{\left(2\right)}\left(\tau_{1},\;\tau_{2}\right)\;+\;G_{\text{con}}^{\left(1\right)}\left(\tau_{1}\right)G_{\text{con}}^{\left(1\right)}\left(\tau_{2}\right)\\ =\underset{\begin{array}{c} \mathrm{connected}\\ \mathrm{events} \end{array}}{\underset{︸}{G_{\text{con}}^{\left(2\right)}\left(\tau_{1},\tau_{2}\right)}}+\underset{\begin{array}{c} \mathrm{dis}–\mathrm{connected}\\ \text{events} \end{array}}{\underset{︸}{\langle \eta \left(\tau_{1}\right)\rangle \langle \eta \left(\tau_{2}\right)\rangle }} \end{array}$$

or, even more practically for our purpose, as

(219c) $$\langle \eta \left(\tau_{1}\right)\eta \left(\tau_{2}\right)\rangle =G_{con}^{\left(2\right)}\left(\tau_{1},\tau_{2}\right)+\eta_{cl}\left(\tau_{1}\right)\eta_{cl}\left(\tau_{2}\right)$$

In similar manner, while applying a kind of recursive rule, sometimes denoted as the *cluster decomposition* or *cumulant expansion*

(220a) $$\langle \eta \left(\tau_{1}\right)\cdots \eta \left(\tau_{n}\right)\rangle =G_{\text{con}}^{\left(n\right)}\left(\tau_{1},\cdots ,\tau_{n}\right)+\langle \eta \left(\tau_{2}\right)\cdots \eta \left(\tau_{n}\right)\rangle G_{\text{con}}^{\left(1\right)}\left(\tau_{1}\right),n\geq 2$$

while involving the pair-wise (Wick) decomposition of the *n*-points correlated function

(220b) $$G_{\text{con}}^{\left(n\right)}\left(\tau_{1},\cdots ,\tau_{n}\right)=\sum_{j=2}^{n}G_{\text{con}}^{\left(2\right)}\left(\tau_{1},\tau_{j}\right){\underset{\left(n-2\right)order\;function}{\underset{︸}{\langle \eta \left(\tau_{k}\right)\cdots \eta \left(\tau_{1}\right)\rangle }}}_{\begin{array}{l} k\neq 1,j\\ l\neq 1,j,k \end{array}}$$

one can easily obtain the higher orders of correlations, however observing that all connected orders of events reduce to the combinations of *pair-connected events*. For instance, we get for the third order fluctuations the average contribution

(221a) $$\begin{array}{c} \langle \eta \left(\tau_{1}\right)\eta \left(\tau_{2}\right)\eta \left(\tau_{3}\right)\rangle \\ =\langle \eta \left(\tau_{1}\right)\rangle \langle \eta \left(\tau_{2}\right)\eta \left(\tau 3\right)\rangle +G_{\text{con}}^{\left(2\right)}\left(\tau_{1},\tau_{2}\right)\langle \eta \left(\tau_{3}\right)\rangle +G_{\text{con}}^{\left(2\right)}\left(\tau_{1},\tau_{3}\right)\langle \eta \left(\tau_{2}\right)\rangle \\ =\eta_{cl}\left(\tau_{1}\right)\left[\eta_{cl}\left(\tau_{2}\right)\eta_{cl}\left(\tau_{3}\right)+G_{\text{con}}^{\left(2\right)}\left(\tau_{2},\tau_{3}\right)\right]+G_{\text{con}}^{\left(2\right)}\left(\tau_{1},\tau_{2}\right)\eta_{cl}\left(\tau_{3}\right)+G_{\text{con}}^{\left(2\right)}\left(\tau_{1},\tau_{3}\right)\eta_{cl}\left(\tau_{2}\right)\\ =\eta_{cl}\left(\tau_{1}\right)\eta_{cl}\left(\tau_{2}\right)\eta_{cl}\left(\tau_{3}\right)+\eta_{cl}\left(\tau_{1}\right)G_{\text{con}}^{\left(2\right)}\left(\tau_{2},\tau_{3}\right)+\eta_{cl}\left(\tau_{2}\right)G_{\text{con}}^{\left(2\right)}\left(\tau_{1},\tau_{3}\right)+\eta_{cl}\left(\tau_{3}\right)G_{\text{con}}^{\left(2\right)}\left(\tau_{1},\tau_{2}\right) \end{array}$$

or with more terms involved for the fourth order

(221b) $$\begin{array}{c} 〈\eta (\tau_{1})\eta (\tau_{2})\eta (\tau_{3})\eta (\tau_{4})〉\\ =\;〈\eta (\tau_{1})〉〈\eta (\tau_{2})\eta (\tau_{3})\eta (\tau_{3})〉\;+\;G_{\text{con}}^{\left(2\right)}\left(\tau_{1},\;\tau_{2}\right)〈\eta (\tau_{3})\eta (\tau_{4})〉\;+\;G_{\text{con}}^{\left(2\right)}\left(\tau_{1},\;\tau_{3}\right)〈\eta (\tau_{2})\eta (\tau_{4})〉\;+\;G_{\text{con}}^{\left(2\right)}(\tau_{1},\;\tau_{4})〈\eta (\tau_{2})\eta (\tau_{3})〉\\ =\;\eta_{cl}(\tau_{1})\left[\eta_{cl}(\tau_{1})\eta_{cl}(\tau_{2})\eta_{cl}(\tau_{3})\;+\;\eta_{cl}(\tau_{1})G_{\text{con}}^{\left(2\right)}\left(\tau_{2},\;\tau_{3}\right)\;+\;\eta_{cl}(\tau_{2})G_{\text{con}}^{\left(2\right)}\left(\tau_{1},\;\tau_{3}\right)\;+\;\eta_{cl}(\tau_{3})G_{\text{con}}^{\left(2\right)}\left(\tau_{1},\;\tau_{2}\right)\right]\\ +\;G_{\text{con}}^{\left(2\right)}\left(\tau_{1},\;\tau_{2}\right)\left[G_{\text{con}}^{\left(2\right)}\left(\tau_{3},\;\tau_{4}\right)\;+\;\eta_{cl}(\tau_{3})\eta_{cl}(\tau_{4})\right]\\ +\;G_{\text{con}}^{\left(2\right)}\left(\tau_{1},\;\tau_{3}\right)\left[G_{\text{con}}^{\left(2\right)}\left(\tau_{2},\;\tau_{4}\right)\;+\;\eta_{cl}(\tau_{2})\eta_{cl}(\tau_{4})\right]\\ +\;G_{\text{con}}^{\left(2\right)}\left(\tau_{1},\;\tau_{4}\right)\left[G_{\text{con}}^{\left(2\right)}\left(\tau_{2},\;\tau_{3}\right)\;+\;\eta_{cl}(\tau_{2})\eta_{cl}(\tau_{3})\right]\\ =\;\eta_{cl}(\tau_{1})\eta_{cl}(\tau_{2})\eta_{cl}(\tau_{3})\eta_{cl}(\tau_{4})\\ +\;\eta_{cl}(\tau_{1})\eta_{cl}(\tau_{2})G_{\text{con}}^{\left(2\right)}\left(\tau_{3},\;\tau_{4}\right)\;+\;\eta_{cl}(\tau_{1})\eta_{cl}(\tau_{3})G_{\text{con}}^{\left(2\right)}\left(\tau_{2},\;\tau_{4}\right)\;+\;\eta_{cl}(\tau_{1})\eta_{cl}(\tau_{4})G_{\text{con}}^{\left(2\right)}\left(\tau_{2},\;\tau_{3}\right)\\ +\;\eta_{cl}(\tau_{2})\eta_{cl}(\tau_{3})G_{\text{con}}^{\left(2\right)}\left(\tau_{1},\;\tau_{4}\right)\;+\;\eta_{cl}(\tau_{2})\eta_{cl}(\tau_{4})G_{\text{con}}^{\left(2\right)}\left(\tau_{1},\;\tau_{3}\right)\;+\;\eta_{cl}(\tau_{3})\eta_{cl}(\tau_{4})G_{\text{con}}^{\left(2\right)}\left(\tau_{1},\;\tau_{2}\right)\\ +\;G_{\text{con}}^{\left(2\right)}\left(\tau_{1},\;\tau_{2}\right)\;G_{\text{con}}^{\left(2\right)}\left(\tau_{3},\;\tau_{4}\right)\;+\;\;G_{\text{con}}^{\left(2\right)}\left(\tau_{1},\;\tau_{3}\right)\;G_{\text{con}}^{\left(2\right)}\left(\tau_{2},\;\tau_{4}\right)\;+\;\;G_{\text{con}}^{\left(2\right)}\left(\tau_{1},\;\tau_{4}\right)\;G_{\text{con}}^{\left(2\right)}\left(\tau_{2},\;\tau_{3}\right) \end{array}$$

Next, having these examples in hand, one tries to re-deriving them by an appropriate generating functional (210) worked with the connected function definition (212). At this moment one uses the previously emphasized “free-harmonic motion” dual nature of fluctuation paths – see Equation (209), to reconsider the free imaginary time amplitude (206a) contribution

(222a) $${\left(\frac{\Delta x}{2}\tau_{b};-\frac{\Delta x}{2}\tau_{a}\right)}_{\left(\omega \right)}=\int_{\eta \left(\tau_{a}\right)=-\Delta x/2}^{\eta \left(\tau_{b}\right)=+\Delta x/2}D\eta \left(\tau \right)\text{exp}\left\{-\frac{1}{\hbar }S_{\omega }^{+}\left(\eta ,\dot{\eta },\tau \right)\right\}$$

with harmonic Euclidian action of fluctuations

(222b) $$S_{\omega }^{+}\left(\eta ,\dot{\eta },\tau \right)=\int_{\tau_{a}}^{\tau_{b}}\left[\frac{m}{2}{\dot{\eta }}^{2}\left(\tau \right)+\frac{m}{2}\omega^{2}\eta^{2}\left(\tau \right)\right]d\tau$$

while fulfilling (at the end of calculation) the constraint

(222c) $$\underset{\omega \to 0}{\text{lim}}{\left(\frac{\Delta x}{2}\tau_{b};-\frac{\Delta x}{2}\tau_{a}\right)}_{\left(\omega \right)}=\left(\frac{\Delta x}{2}\tau_{b};-\frac{\Delta x}{2}\tau_{a}\right)\left[j\right]$$

We like to rearrange the action (222b) so that the quantum current contribution to clearly appear; in achieving this one firstly rewrites it by performing the integration by parts

(223) $$\begin{array}{c} S_{\omega }^{+}\left(\eta ,\dot{\eta },\tau \right)=\int_{\tau_{a}}^{\tau_{b}}\left\{\frac{m}{2}\left[\frac{d}{d\tau }\left(\eta \frac{d\eta }{d\tau }\right)-\eta \frac{d^{2}\eta }{d\tau^{2}}\right]+\frac{m}{2}\omega^{2}\eta^{2}\left(\tau \right)\right\}d\tau \\ =\frac{m}{2}\underset{0}{\underset{︸}{{\left(\eta \dot{\eta }\right)}_{\tau_{a}}^{\tau_{b}}}}+\int_{\tau_{a}}^{\tau_{b}}\left\{\frac{m}{2}\eta \left(\tau \right)\left[-\partial_{\tau }^{2}+\omega^{2}\right]\eta \left(\tau \right)\right\}d\tau \\ =\int_{\tau_{a}}^{\tau_{b}}\left\{\frac{m}{2}\eta \left(\tau \right)D_{\omega }\left(\tau \right)\eta \left(\tau \right)\right\}d\tau \end{array}$$

where we have recognized the harmonic differential operator

(224) $$D_{\omega }\left(\tau \right)=-\partial_{\tau }^{2}+\omega^{2}$$

The form (223) is very useful through employing the Green equation of harmonic motion

(225a) $$D_{\omega }\left(\tau \right)G_{\omega }\left(\tau ,\tau \prime \right)=\delta \left(\tau -\tau \prime \right)$$

for the integral property

(225b) $$\int D_{\omega }\left(\tau \right)G_{\omega }\left(\tau ,\tau \prime \right)f\left(\tau ,\tau \prime \right)d\tau =\int f\left(\tau ,\tau \prime \right)\delta \left(\tau -\tau \prime \right)d\tau =f\left(\tau \prime ,\tau \prime \right)$$

to perform the path shifting of fluctuations by the transformation

(226) $$\eta \left(\tau \right)\to \eta \prime \left(\tau \right)=\eta \left(\tau \right)+\frac{1}{m}G_{\omega }\left(\tau ,\tau \prime \right)j\left(\tau \right)$$

on the combined term

(227) $$\begin{array}{c} \frac{m}{2}\eta \left(\tau \right)D_{\omega }\left(\tau \right)\eta \left(\tau \right)\to \frac{m}{2}\eta^{\prime }\left(\tau \right)D_{\omega }\left(\tau \right)\eta^{\prime }\left(\tau \right)\\ -\left[\int \eta^{\prime }\left(\tau \right)D_{\omega }\left(\tau \right)G_{\omega }\left(\tau ,\tau^{\prime }\right)j\left(\tau^{\prime }\right)\right]d\tau^{\prime }+\frac{1}{2m}\iint \left[j\left(\tau \right)D_{\omega }\left(\tau \right)G_{\omega }\left(\tau ,\tau^{\prime }\right)G_{\omega }\left(\tau^{\prime },\tau^{\prime \prime }\right)j\left(\tau^{\prime \prime }\right)\right]d\tau^{\prime }d\tau^{\prime \prime } \end{array}$$

so that the prescribed action of (223) takes the form

(228) $$\begin{array}{c} S_{\omega }^{+}\left(\eta ,\dot{\eta },\tau \right)\to \int_{\tau_{a}}^{\tau_{b}}\left\{\frac{m}{2}\eta \prime \left(\tau \right)D_{\omega }\left(\tau \right)\eta \prime \left(\tau \right)\right\}d\tau \\ -\int_{\tau_{a}}^{\tau_{b}}\eta \prime \left(\tau \prime \right)j\left(\tau \prime \right)d\tau \prime +\frac{1}{2m}\int_{\tau_{a}}^{\tau_{b}}\int_{\tau_{a}}^{\tau_{b}}j\left(\tau \prime \right)G_{\omega }\left(\tau \prime ,\tau \prime \prime \right)j\left(\tau \prime \prime \right)\frac{d\tau \prime }{i}\frac{d\tau \prime \prime }{i} \end{array}$$

under the assumption the *physical* integration interval (*τ_a_*,*τ_b_*) assimilates the entirely evolution universe of the concerned problem, *i.e.*, the *mathematical* interval (–∞,+∞), so that the delta-Dirac integration property (225b) is consistent. Also note that in expression (228) since the statistical Green function should come from its associate original real time quantum mechanically problem, see below, it tracks also the temporal Wick “rotation *t* = *τ*/*i* ” in the integration measure, explaining therefore the complex factors in the last term of (228).

With these, the harmonic fluctuation action of (228) may be reconsidered with the working form

(229) $$\begin{array}{c} {\tilde{S}}_{\omega }^{+}(\eta ,\;\dot{\eta },\;\tau )\;=\;\int_{\tau_{a}}^{\tau_{b}}\left\{\frac{m}{2}\eta (\tau )\left[-\partial_{\tau }^{2}\;+\;\omega^{2}\right]\eta (\tau )\right\}d\tau \\ -\int_{\tau_{a}}^{\tau_{b}}\eta (\tau )j(\tau )d\tau \;-\;\frac{1}{2m}\int_{\tau_{a}}^{\tau_{b}}\int_{\tau_{a}}^{\tau_{b}}G_{\omega }\left(\tau ,\;\tau^{\prime }\right)j(\tau )j(\tau^{\prime })d\tau d\tau^{\prime } \end{array}$$

such that it can be further rearranged so that the free terms action to appear distinctively under the condition *ω →* 0 as

(230a) $${\tilde{S}}_{\omega \to 0}^{+}\left(\eta ,\dot{\eta },\tau \right)=S_{0}^{+}\left(\eta ,\dot{\eta },\tau \right)-\int_{\tau_{a}}^{\tau_{b}}\eta \left(\tau \right)j\left(\tau \right)d\tau -\frac{1}{2m}\int_{\tau_{a}}^{\tau_{b}}\int_{\tau_{a}}^{\tau_{b}}G_{\omega \to 0}\left(\tau ,\tau \prime \right)j\left(\tau \right)j\left(\tau \prime \right)d\tau d\tau \prime$$

with the free Euclidian action

(230b) $$S_{0}^{+}\left(\eta ,\dot{\eta },\tau \right)=\int_{\tau_{a}}^{\tau_{b}}\left[\frac{m}{2}{\dot{\eta }}^{2}\left(\tau \right)\right]d\tau$$

recovered though considering on expression (229) the reverse integration by parts - as unfolded from (222b) to (223).

Finally, back with the identifications

(231a) $$\eta \left(\tau \right)\to \eta_{cl}\left(\tau \right)$$

(231b) $$\frac{\hbar }{m}G_{\omega \to 0}\left(\tau ,\tau \prime \right)\to G_{\text{con}}^{\left(2\right)}\left(\tau ,\tau \prime \right)$$

in the action (230), there follows that its last two terms are no longer displaying quantum fluctuations upon integration since they were comprised under averaged forms, see Equations (218) and (219c), respectively; thus they release for the searched current-dependent amplitude of (222c) the actual solution

(232a) $$\begin{array}{c} \left(\frac{\Delta x}{2}\tau_{b};-\frac{\Delta x}{2}\tau_{a}\right)\left[j\right]=\underset{\omega \to 0}{\text{lim}}\int_{\eta \left(\tau_{a}\right)=-\Delta x/2}^{\eta \left(\tau_{b}\right)=+\Delta x/2}D\eta \left(\tau \right)\text{exp}\left\{-\frac{1}{\hbar }S_{\omega }^{+}\left(\eta ,\dot{\eta },\tau \right)\right\}\\ =\int_{\eta \left(\tau_{a}\right)=-\Delta x/2}^{\eta \left(\tau_{b}\right)=+\Delta x/2}D\eta \left(\tau \right)\text{exp}\left\{-\frac{1}{\hbar }{\tilde{S}}_{\omega \to 0}^{+}\left(\eta ,\dot{\eta },\tau \right)\right\}\\ =\left(\int_{\eta \left(\tau_{a}\right)}^{\eta \left(\tau_{b}\right)}D\eta \left(\tau \right)\text{exp}\left\{-\frac{1}{\hbar }S_{0}^{+}\left(\eta ,\dot{\eta },\tau \right)\right\}\right)\\ \times \text{exp}\left(\frac{1}{\hbar }\int_{\tau_{a}}^{\tau_{b}}\eta_{cl}\left(\tau \right)j\left(\tau \right)d\tau +\frac{1}{2\hbar^{2}}\int_{\tau_{a}}^{\tau_{b}}\int_{\tau_{a}}^{\tau_{b}}G_{\text{con}}^{\left(2\right)}\left(\tau ,\tau \prime \right)j\left(\tau \right)j\left(\tau \prime \right)d\tau d\tau \prime \right) \end{array}$$

which ultimately simply re-writes like the current dependent propagator amplitude

(232b) $$\begin{array}{c} \left(\frac{\Delta x}{2}\tau_{b};-\frac{\Delta x}{2}\tau_{a}\right)\left[j\right]\\ ={\left(\frac{\Delta x}{2}\tau_{b};-\frac{\Delta x}{2}\tau_{a}\right)}_{\left(0\right)}\text{exp}\left(\frac{1}{\hbar }\int_{\tau_{a}}^{\tau_{b}}\eta_{cl}\left(\tau \right)j\left(\tau \right)d\tau +\frac{1}{2\hbar^{2}}\int_{\tau_{a}}^{\tau_{b}}\int_{\tau_{a}}^{\tau_{b}}G_{\text{con}}^{\left(2\right)}\left(\tau ,\tau \prime \right)j\left(\tau \right)j\left(\tau \prime \right)d\tau d\tau \prime \right) \end{array}$$

as a wave-perturbative form of the free fluctuation amplitude (206a), being intermediated by the harmonic towards free limiting motion of quantum fluctuations. Let’s further comment that the actual form (232b) generalizes the previously “guessed” form (210), which provided the first order fluctuation correlation, however having in addition the power to recover all other superior orders of correlation, for instance those given by Equations (219c) and (221), by successive application of the formula (212); this way, the transformation factor *ħ*/ *m* in (231b) is as well justified.

With these the connected correlation function algorithm was proofed in detail, being at disposition to be implemented for whatever order of semiclassical expansion of the path integral evolution amplitude (208); as exemplification, the next section will expose the analytic solution for the second order case.

### 4.3. Classical Fluctuation Path and Connected Green Function

We have already seen that aiming to evaluate any of the above connected correlation functions one imperatively needs to know the analytical forms of classical fluctuation path

(233a) $$\langle \eta_{i}\left(\tau \right)\rangle =\eta_{\left(cl\right)i}\left(\tau \right)$$

and for the connected Green function - identified from (231b) as

(233b) $$G_{\text{con}}^{\left(2\right)}\left(\tau ,\tau \prime \right)=\frac{\hbar }{m}G_{\omega \to 0}\left(\tau ,\tau \prime \right)$$

while depending on its turn of the knowledge of the Green function for the harmonic oscillator problem, Equation (225a) with (224), to be finally specialized towards the “free harmonic” limit *ω →* 0.

Therefore, with the quantities of Equations (233) any semiclassical problem can be solved analytically. Yet, for the quantum objects in question the computing procedure consists by three major stages, as unfolded in the sequel.

- Solving the associate real time harmonic problem;
- Rotating the solution into imaginary time picture;
- Taking the “free harmonic limit”*ω →* 0.

#### 4.3.1. Calculation of Classical Fluctuation Path

As discussed above, the classical path for quantum fluctuation will not be written directly from the ordinary path free motion (Section 3.3.2) but using the similar result of harmonic motion (Section 3.3.3) upon which the free-harmonic condition *ω →* 0 will be imposed; actually, the procedure is unfolded as follows.

The result (175a) is combined with (177a) to provide the real time classical path

(234) $$\begin{array}{c} x_{\left(\omega \right)cl}\left(t\right)=\frac{1}{\omega }\frac{\omega }{\text{sin}\left[\omega \left(t_{b}-t_{a}\right)\right]}\left\{x_{b}-x_{a}\text{cos}\left[\omega \left(t_{b}-t_{a}\right)\right]\right\}\text{sin}\left[\omega \left(t-t_{a}\right)\right]+x_{a}\text{cos}\left[\omega \left(t-t_{a}\right)\right]\\ =\frac{x_{b}\text{sin}\left[\omega \left(t-t_{a}\right)\right]+x_{a}\left\{\text{sin}\left[\omega \left(t_{b}-t_{a}\right)\right]\text{cos}\left[\omega \left(t-t_{a}\right)\right]-\text{cos}\left[\omega \left(t_{b}-t_{a}\right)\right]\text{sin}\left[\omega \left(t-t_{a}\right)\right]\right\}}{\text{sin}\left[\omega \left(t_{b}-t_{a}\right)\right]}\\ =\frac{x_{b}\text{sin}\left[\omega \left(t-t_{a}\right)\right]+x_{a}\text{sin}\left[\omega \left(t_{b}-t\right)\right]}{\text{sin}\left[\omega \left(t_{b}-t_{a}\right)\right]} \end{array}$$

The real to imaginary time rotation is performed on the result (234) according with the Wick rule prescription of (196a), being this equivalently of directly rewriting of expression (234) replacing the trigonometric functions by their hyperbolic counterparts, according with the previously explained conversion, see Equation (188a)

(235) $$x_{cl}\left(\tau \right)=\frac{x_{b}\text{sinh}\left[\omega \left(\tau -\tau_{a}\right)\right]+x_{a}\text{sinh}\left[\omega \left(\tau_{b}-\tau \right)\right]}{\text{sinh}\left[\omega \left(\tau_{b}-\tau_{a}\right)\right]}$$

The “free-harmonic” (*ω →* 0) limit is performed upon the expression (235) through employing the ordinary hyperbolic limit

(236) $$\underset{\omega \to 0}{\text{lim}}\text{sinh}\left[\omega \Xi \right]=\omega \Xi$$

This gives

(237) $$x_{\left(\omega \to 0\right)cl}\left(\tau \right)=\frac{x_{b}\left(\tau -\tau_{a}\right)+x_{a}\left(\tau_{b}-\tau \right)}{\tau_{b}-\tau_{a}}$$

which evidently does the same job as the classical free-motion result of (165b), although not identical, since derived from a generalized perspective here.

The result (237) is implemented in the formula (197) to finally produce the classical fluctuation path

(238a) $$\begin{array}{c} \langle \eta \left(\tau \right)\rangle =\eta_{cl}\left(\tau \right)\equiv \eta_{\left(\omega \to 0\right)cl}\left(\tau \right)=x_{\left(\omega \to 0\right)cl}\left(\tau \right)-\bar{x}\\ =\frac{x_{b}\left(\tau -\tau_{a}\right)+x_{a}\left(\tau_{b}-\tau \right)}{\tau_{b}-\tau_{a}}-\frac{x_{a}+x_{b}}{2}\\ =\left[\frac{\tau }{\tau_{b}-\tau_{a}}-\frac{1}{2}\frac{\tau_{b}+\tau_{a}}{\tau_{b}-\tau_{a}}\right]\Delta x \end{array}$$

which takes even the simpler form

(238b) $$\langle \eta \left(\tau \right)\rangle =\left(\frac{\tau }{\hbar \beta }-\frac{1}{2}\right)\Delta x$$

when rewritten within the thermodynamic picture

(239a) $$\tau_{a}=0$$

(239b) $$\tau_{b}=\hbar \beta$$

#### 4.3.2. Calculation of the Connected Green Function

Now, going to the evaluation of the expression (233b) we need the Green function of the harmonic oscillator from the equation (225a); when rewritten in real time picture

(240a) $$\left[-\partial_{t}^{2}+\omega^{2}\right]G_{\omega }\left(t,t\prime \right)=\delta \left(t-t\prime \right),t>t\prime \in \left(t_{a},t_{b}\right)$$

it has the advantage of having the frontier values fixed by the Dirichlet boundary conditions

(240b) $$G_{\omega }\left(t,t\prime \right)=0\{\begin{array}{ll} t=t_{b} & ,\forall t\prime \\ \forall t & ,t\prime =t_{a} \end{array}$$

in the same manner as the fluctuation paths in real time are set to vanish at the endpoint frontier, see Equation (145c). Such double boundary condition fixes the type of solution as being of the double trigonometric form

(240c) $$G_{\omega }\left(t,t\prime \right)=C\text{sin}\left[\omega \left(t_{b}-t\right)\right]\text{sin}\left[\omega \left(t\prime -t_{a}\right)\right]$$

In the same manner the temporal alternative ordering problem of (240)

(241a) $$\left[-\partial_{t\prime }^{2}+\omega^{2}\right]G_{\omega }\left(t\prime ,t\right)=\delta \left(t\prime -t\right),t\prime >t\in \left(t_{a},t_{b}\right)$$

with the Dirichlet boundary conditions

(241b) $$G_{\omega }\left(t\prime ,t\right)=0\{\begin{array}{ll} t\prime =t_{b} & ,\forall t\\ \forall t\prime & ,t=t_{a} \end{array}$$

produces the variant Green function

(241c) $$G_{\omega }\left(t\prime ,t\right)=C\text{sin}\left[\omega \left(t_{b}-t\prime \right)\right]\text{sin}\left[\omega \left(t-t_{a}\right)\right]$$

with the same constant as for the solution (240c) since recognizing that both formally belong to the same homogeneous equation of type

(242) $$\left[-\partial_{t_{>}}^{2}+\omega^{2}\right]G_{\omega }\left(t_{>},t_{<}\right)=0,t_{>}>t_{<}\in \left(t_{a},t_{b}\right)$$

Now, looking for appropriate identification in the inhomogeneous equation

(243) $$-\partial_{t}^{2}G_{\omega }\left(t,t\prime \right)=\delta \left(t-t\prime \right)+\omega^{2}G_{\omega }\left(t,t\prime \right)$$

one notes that its left side is formed from the difference of the first derivatives of the solutions (240c) and (241c) approaching each other for the concerned times

(244) $$\begin{array}{c} -\partial_{t}^{2}G_{\omega }\left(t,t\prime \right)=-\underset{t\to t\prime }{\text{lim}}\frac{\partial_{t}G_{\omega }\left(t,t\prime \right)-\partial_{t}G_{\omega }\left(t\prime ,t\right)}{t-t\prime }\\ =C\omega \underset{t\to t\prime }{\text{lim}}\frac{1}{t-t\prime }\left\{\text{cos}\left[\omega \left(t_{b}-t\right)\right]\text{sin}\left[\omega \left(t\prime -t_{a}\right)\right]+\text{sin}\left[\omega \left(t_{b}-t\prime \right)\right]\text{cos}\left[\omega \left(t-t_{a}\right)\right]\right\}\\ =C\omega \left\{\text{cos}\left[\omega \left(t_{b}-t\prime \right)\right]\text{sin}\left[\omega \left(t\prime -t_{a}\right)\right]+\text{sin}\left[\omega \left(t_{b}-t\prime \right)\right]\text{cos}\left[\omega \left(t\prime -t_{a}\right)\right]\right\}\underset{t\to t\prime }{\text{lim}}\frac{1}{t-t\prime }\\ =C\omega \text{sin}\left[\omega \left(t_{b}-t_{a}\right)\right]\delta \left(t-t\prime \right) \end{array}$$

which, through comparing with the right side first term of (243) gives the searched constant

(245) $$C=\frac{1}{\omega \text{sin}\left[\omega \left(t_{b}-t_{a}\right)\right]}$$

It leaves with the real time Green function solution of the harmonic oscillator

(246) $$G_{\omega }\left(t,t\prime \right)=\frac{\Theta \left(t-t\prime \right)\text{sin}\left[\omega \left(t_{b}-t\right)\right]\text{sin}\left[\omega \left(t\prime -t_{a}\right)\right]+\Theta \left(t\prime -t\right)\text{sin}\left[\omega \left(t_{b}-t\prime \right)\right]\text{sin}\left[\omega \left(t-t_{a}\right)\right]}{\omega \text{sin}\left[\omega \left(t_{b}-t_{a}\right)\right]}$$

that nevertheless combines both above solution with the help of Heaviside step-function

(247) $$\Theta \left(t-t\prime \right)=\{\begin{array}{l} 1,t>t\prime \\ \frac{1}{2},t=t\prime \\ 0,t<t\prime \end{array}$$

Next, as previously done with the fluctuation paths, the change to the imaginary time picture is done automatically through trigonometric-to-hyperbolic recipe (188a) to give

(248) $$G_{\omega }\left(\tau ,\tau \prime \right)=\frac{\Theta \left(\tau -\tau \prime \right)\text{sinh}\left[\omega \left(\tau_{b}-\tau \right)\right]\text{sinh}\left[\omega \left(\tau \prime -\tau_{a}\right)\right]+\Theta \left(\tau \prime -\tau \right)\text{sinh}\left[\omega \left(\tau_{b}-\tau \prime \right)\right]\text{sinh}\left[\omega \left(\tau -\tau_{a}\right)\right]}{\omega \text{sinh}\left[\omega \left(\tau_{b}-\tau_{a}\right)\right]}$$

noting that in the course of transformation the factor

(249a) $$i\left(i\frac{1}{i\cdot i}\right)=1$$

was tacitly absorbed, with the parenthesis complex indices coming from the trigonometric to hyperbolic rotation (188a), while the outside index complex assures the equivalence of Green function contribution for the canonic-to-Euclidian path integrals action exponents’ transformation

(249b) $$\underset{\text{CANONIC}}{\underset{︸}{\text{exp}\left(\frac{i}{\hbar }\left[\cdots G_{\omega }\left(t,t\prime \right)\cdots \right]\right)}}\to \text{exp}\left(\frac{i}{\hbar }\left[\cdots iG_{\omega }\left(t\to \tau ,t\prime \to \tau \prime \right)\cdots \right]\right)=\underset{\text{EUCLIDIAN}}{\underset{︸}{\text{exp}\left(-\frac{1}{\hbar }\left[\cdots G_{\omega }\left(\tau ,\tau \prime \right)\cdots \right]\right)}}$$

Expression (248) is finally employed to the “free harmonic” limit (236) providing the result

(250) $$G_{\omega }\left(\tau ,\tau \prime \right)=\frac{\Theta \left(\tau -\tau \prime \right)\left(\tau_{b}-\tau \right)\left(\tau \prime -\tau_{a}\right)+\Theta \left(\tau \prime -\tau \right)\left(\tau_{b}-\tau \prime \right)\left(\tau -\tau_{a}\right)}{\tau_{b}-\tau_{a}}$$

which being free of harmonic influence it remains identically also from the quantity *G_ω→_*_0_ (*τ*, *τ*'). Still, it has to be converted into the searched connected Green function (233b), leaving with the time imaginary form

(251a) $$G_{\text{con}}^{\left(2\right)}\left(\tau ,\tau^{\prime }\right)=\frac{\hbar }{m}\frac{\Theta \left(\tau -\tau^{\prime }\right)\left(\tau_{b}-\tau \right)\left(\tau^{\prime }-\tau_{a}\right)+\Theta \left(\tau^{\prime }-\tau \right)\left(\tau_{b}-\tau \prime \right)\left(\tau -\tau_{a}\right)}{\tau_{b}-\tau_{a}}$$

or with its equivalent statistical one

(251b) $$G_{\text{con}}^{\left(2\right)}\left(\tau ,\tau^{\prime }\right)=\frac{\Theta \left(\tau -\tau^{\prime }\right)\left(\hbar \beta -\tau \right)\tau^{\prime }+\Theta \left(\tau^{\prime }-\tau \right)\left(\hbar \beta -\tau^{\prime }\right)\tau }{m\beta }$$

when the thermodynamical picture (239) is considered.

### 4.4. Second Order Semiclassical Propagator, Partition Function and Density

Returning to evaluate the second order truncated expansion (208) one needs the evaluation of the quantities 〈*η_i_*(*τ*)〉, 〈*η_i_*(*τ*)*η_j_*(*τ*)〉, 〈*η_i_*(*τ*)*η_j_*(*τ*')〉 and of their integration. Given the previous discussions, see for instance the Equations (218) and (219c), one immediately has

(252a) $$\langle \eta_{i}\left(\tau \right)\rangle =\eta_{\left(cl\right)i}\left(\tau \right)$$

(252b) $$\langle n_{i}\left(\tau \right)\eta_{j}\left(\tau^{\prime }\right)\rangle =\eta_{\left(cl\right)i}\left(\tau \right)\eta_{\left(cl\right)j}\left(\tau^{\prime }\right)+G_{\text{con}}^{\left(2\right)}\left(\tau ,\tau^{\prime }\right)\delta_{ij}$$

With the help of expression (238) the first order averaged fluctuation integral appearing on (208) becomes

(253) $$\int_{\tau_{a}}^{\tau_{b}}\langle \eta \left(\tau \right)\rangle d\tau =\int_{\tau_{a}}^{\tau_{b}}\eta_{cl}\left(\tau \right)d\tau =0$$

Going now to the double connected correlation functions, one has the working analytical expression

(254) $$\begin{array}{c} \langle \eta_{i}\left(\tau \right)\eta_{j}\left(\tau \prime \right)\rangle =\left(\frac{\tau }{\tau_{b}-\tau_{a}}-\frac{1}{2}\frac{\tau_{b}+\tau_{a}}{\tau_{b}-\tau_{a}}\right)\left(\frac{\tau \prime }{\tau_{b}-\tau_{a}}-\frac{1}{2}\frac{\tau_{b}+\tau_{a}}{\tau_{b}-\tau_{a}}\right)\Delta x_{i}\Delta x_{j}\\ +\frac{\hbar }{m}\frac{\Theta \left(\tau -\tau \prime \right)\left(\tau_{b}-\tau \right)\left(\tau \prime -\tau_{a}\right)+\Theta \left(\tau \prime -\tau \right)\left(\tau_{b}-\tau \prime \right)\left(\tau -\tau_{a}\right)}{\tau_{b}-\tau_{a}}\delta_{ij} \end{array}$$

obtained by replacing into the expression (252b) the classical fluctuation paths and the connected Green function components, Equations (238a) and (251a), respectively.

Now, the second order averaged fluctuation integrals are computed as following:

- At coincident times
  (255a) $$\begin{array}{c} \int_{\tau_{a}}^{\tau_{b}}\langle \eta_{i}\left(\tau \right)\eta_{j}\left(\tau \right)\rangle d\tau =\delta_{ij}\frac{\hbar }{m\left(\tau_{b}-\tau_{a}\right)}\int_{\tau_{a}}^{\tau_{b}}\left(\tau_{b}-\tau \right)\left(\tau -\tau_{a}\right)d\tau +\Delta x_{i}\Delta x_{j}\int_{\tau_{a}}^{\tau_{b}}{\left(\frac{\tau }{\tau_{a}-\tau_{b}}-\frac{1}{2}\frac{\tau_{b}+\tau_{a}}{\tau_{b}-\tau_{a}}\right)}^{2}d\tau \\ =\delta_{ij}\frac{\hbar }{m}\frac{{\left(\tau_{b}-\tau_{a}\right)}^{2}}{6}+\Delta x_{i}\Delta x_{j}\frac{\tau_{b}-\tau_{a}}{12} \end{array}$$
  or re-written as
  (255b) $$\int_{0}^{\hbar \beta }\langle \eta_{i}\left(\tau \right)\eta_{j}\left(\tau \right)\rangle d\tau =\delta_{ij}\frac{\hbar^{3}\beta^{2}}{6m}+\Delta x_{i}\Delta x_{j}\frac{\hbar \beta }{12}$$
  within the thermodynamical environment given by Equation (239).
- At different times
  (256a) $$\begin{array}{c} \int_{\tau_{a}}^{\tau_{b}}d\tau \int_{\tau_{a}}^{\tau_{b}}d\tau^{\prime }\langle \eta_{i}\left(\tau \right)\eta_{j}\left(\tau^{\prime }\right)\rangle =\delta_{ij}\frac{\hbar }{m\left(\tau_{b}-\tau_{a}\right)}\left[\int_{\tau_{a}}^{\tau_{b}}d\tau \int_{\tau_{a}}^{\tau }d\tau^{\prime }\left(\tau_{b}-\tau \right)\left(\tau^{\prime }-\tau_{a}\right)+\int_{\tau_{a}}^{\tau_{b}}d\tau^{\prime }\int_{\tau_{a}}^{\tau^{\prime }}d\tau \left(\tau_{b}-\tau^{\prime }\right)\left(\tau -\tau_{a}\right)\right]\\ +\left[\int_{\tau_{a}}^{\tau_{b}}\left(\frac{\tau }{\tau_{b}-\tau_{a}}-\frac{1}{2}\frac{\tau_{b}+\tau_{a}}{\tau_{b}-\tau_{a}}\right)d\tau \right]\left[\int_{\tau_{a}}^{\tau_{b}}\left(\frac{\tau^{\prime }}{\tau_{b}-\tau_{a}}-\frac{1}{2}\frac{\tau_{b}+\tau_{a}}{\tau_{b}-\tau_{a}}\right)d\tau^{\prime }\right]\Delta x_{i}\Delta x_{j}\\ =\delta_{ij}\frac{\hbar }{m}\frac{{\left(\tau_{b}-\tau_{a}\right)}^{3}}{12} \end{array}$$
  with its quantum thermodynamical counterpart
  (256b) $$\int_{\tau_{a}}^{\tau_{b}}d\tau \int_{\tau_{a}}^{\tau_{b}}d\tau^{\prime }\langle \eta_{i}\left(\tau \right)\eta_{j}\left(\tau^{\prime }\right)\rangle =\delta_{ij}\frac{\hbar^{4}\beta^{3}}{12m}$$

Finally, while replacing the values of Equations (253), (255b), and (256b) back in the second order truncated semiclassical expression of imaginary time amplitude (208) one gets the analytical result

(257) $$\begin{array}{c} {\left(x_{b}\hbar \beta ;x_{a}0\right)}_{QS}^{\left[II\right]}=\sqrt{\frac{m}{2\pi \hbar^{2}\beta }}\text{exp}\left\{-\frac{m}{2\hbar^{2}\beta }{\left(\Delta x\right)}^{2}-\beta V\left(\bar{x}\right)\right\}\\ \times \left\{\begin{array}{l} 1-\frac{1}{\hbar }\partial_{i}V\left(\bar{x}\right)\int_{0}^{\hbar \beta }d\tau \langle \eta_{i}\left(\tau \right)\rangle -\frac{1}{2\hbar }\partial_{i}\partial_{j}V\left(\bar{x}\right)\int_{0}^{\hbar \beta }d\tau \langle \eta_{i}\left(\tau \right)\eta_{j}\left(\tau \right)\rangle \\ +\frac{1}{2\hbar^{2}}\partial_{i}V\left(\bar{x}\right)\partial_{j}V\left(\bar{x}\right)\int_{0}^{\hbar \beta }d\tau \int_{0}^{\hbar \beta }d\tau^{\prime }\langle \eta_{i}\left(\tau \right)\eta_{j}\left(\tau^{\prime }\right)\rangle \end{array}\right\}\\ =\sqrt{\frac{m}{2\pi \hbar^{2}\beta }}\text{exp}\left\{-\frac{m}{2\hbar^{2}\beta }{\left(\Delta x\right)}^{2}-\beta V\left(\bar{x}\right)\right\}\\ \times \left\{1-\frac{1}{2\hbar }\partial_{i}\partial_{j}V\left(\bar{x}\right)\left[\delta_{ij}\frac{\hbar^{3}\beta^{2}}{6m}+\Delta x_{i}\Delta x_{j}\frac{\hbar \beta }{12}\right]+\frac{1}{2\hbar^{2}}\partial_{i}V\left(\bar{x}\right)\partial_{j}V\left(\bar{x}\right)\delta_{ij}\frac{\hbar^{4}\beta^{3}}{12m}\right\};\\ {\left(x_{b}\hbar \beta ;x_{a}0\right)}_{QS}^{\left[II\right]}=\sqrt{\frac{m}{2\pi \hbar^{2}\beta }}\text{exp}\left\{-\frac{m}{2\hbar^{2}\beta }{\left(\Delta x\right)}^{2}-\beta V\left(\bar{x}\right)\right\}\\ \times \left\{1-\frac{\hbar^{2}\beta^{2}}{12m}\nabla^{2}V\left(\bar{x}\right)-\frac{\beta }{24}{\left(\Delta x\nabla \right)}^{2}V\left(\bar{x}\right)+\frac{\hbar^{2}\beta^{3}}{24m}{\left[\nabla V\left(\bar{x}\right)\right]}^{2}\right\} \end{array}$$

Note that the expression (257) plays the role of the semiclassical canonical density in PI-DFT algorithm given by Equations (128) and (129)

(258) $$\begin{array}{c} {\rho_{⊗QS}^{\left[II\right]}\left(\bar{x},\beta \right)={\left(x_{b}\hbar \beta ;x_{a}0\right)}_{QS}^{\left[II\right]}|}_{x_{a}=x_{b}}\\ =\sqrt{\frac{m}{2\pi \hbar^{2}\beta }}\left\{1-\frac{\hbar^{2}\beta^{2}}{12m}\nabla^{2}V\left(\bar{x}\right)+\frac{\hbar^{2}\beta^{3}}{24m}{\left[\nabla V\left(\bar{x}\right)\right]}^{2}\right\}\text{exp}\left\{-\beta V\left(\bar{x}\right)\right\} \end{array}$$

to construct the *N*-body density at thermodynamic equilibrium

(259) $$\rho_{N-QS}^{\left[II\right]}\left(\bar{x},\beta \right)=\frac{N}{Z^{\left[II\right]}\left(\beta \right)}\rho_{⊗QS}^{\left[II\right]}\left(\bar{x},\beta \right)$$

by means of partition function

(260) $$\begin{array}{c} Z^{\left[II\right]}\left(\beta \right)=\int \rho_{⊗QS}^{\left[II\right]}\left(\bar{x},\beta \right)d\bar{x}\\ =\sqrt{\frac{m}{2\pi \hbar^{2}\beta }}\int \left\{1-\frac{\hbar^{2}\beta^{2}}{12m}\nabla^{2}V\left(\bar{x}\right)+\frac{\hbar^{2}\beta^{3}}{24m}{\left[\nabla V\left(\bar{x}\right)\right]}^{2}\right\}\text{exp}\left\{-\beta V\left(\bar{x}\right)\right\}d\bar{x} \end{array}$$

At this point, the expression (260) may be elegantly transformed through considering the Gauss theorem of integrated divergence that written in a general *D*-dimensional case

(261a) $$\int \nabla \left\{\left[\nabla V\left(\bar{x}\right)\right]\text{exp}\left[-\beta V\left(\bar{x}\right)\right]\right\}d^{D}\bar{x}=0$$

leaves with the useful differential relationship

(261b) $$\int {\left[\nabla V\left(\bar{x}\right)\right]}^{2}\text{exp}\left[-\beta V\left(\bar{x}\right)\right]d^{D}\bar{x}=\frac{1}{\beta }\int \nabla^{2}V\left(\bar{x}\right)\text{exp}\left[-\beta V\left(\bar{x}\right)\right]d^{D}\bar{x}$$

helping in rearranging the partition function (260) firstly as

(262a) $$Z^{\left[II\right]}\left(\beta \right)=\sqrt{\frac{m}{2\pi \hbar^{2}\beta }}\int \left[1-\frac{\hbar^{2}\beta^{2}}{24m}\nabla^{2}V\left(\bar{x}\right)\right]\text{exp}\left\{-\beta V\left(\bar{x}\right)\right\}d\bar{x}$$

and finally, after the exponential resume, equivalently as

(262b) $$Z^{\left[II\right]}\left(\beta \right)=\sqrt{\frac{m}{2\pi \hbar^{2}\beta }}\int \text{exp}\left[-\beta V\left(\bar{x}\right)-\frac{\hbar^{2}\beta^{2}}{24m}\nabla^{2}V\left(\bar{x}\right)\right]d\bar{x}$$

In the same manner also the higher orders of semiclassical expansion of density matrix (204) or (208) can be constructed by following the cumulant expansion (220), its fluctuation path and connected Green function components, as given by Equations (238) and (251), respectively, towards producing the analytical canonical density, the partition function and finally the many-body density to be used in density functional theory and of its (chemical) applications [71]. Such an application is to be in next presented for electronegativity and chemical hardness indices’ computations.

### 4.5. Fourth Order Semiclassical Electronegativity and Chemical Hardness

Here, we assess electronegativity (EN) as the convolution of the imaginary time conditional probability (*r*, *τ*|0,0), *τ* = Im(*it*), with the radial valence shell potential*V* (*r*) [72]

(263) χ=∫V(r)(r,τ;0,0)dr

so representing the power of the entire atom (nucleus + core electrons + valence shell) to attract electrons of the outer shell (fixed by radius *r*) to its center (*r* = 0). This way, the current EN definition is refined to account for the whole stability of the atom with its electronic and nuclear subsystems.

Having an analytical EN quantum formulation, the chemical hardness, *η*, its natural companion, is re-expressed from Equation (13b) under the explicitly working form [73]

(264) $$\eta \;\equiv \;-\;{\left(\frac{\partial \chi }{\partial N}\right)}_{V(r)}$$

playing a major role in establishing the main chemical principles of reactivity towards stability: the hard-and-soft-acids-and-bases (HSAB) and the maximum hardness (MH) [74–78].

The general radial one-dimensional probability amplitude connecting the space-time events (*r_a_*, 0) and (*r_b_*, *τ*) for electronic evolution amplitude in an atom can be derived from the semiclassical expansion, by extending the expansion (208) up to the fourth order through including the terms of type (221), whose calculation leads to the result [71]

(265) $$\begin{array}{c} (r_{b},\tau ;r_{a},0)=\sqrt{\frac{m}{2\pi \hbar \tau }}\text{exp}\left\{-\frac{m}{2\hbar \tau }{\left(\Delta r\right)}^{2}-\frac{\tau }{\hbar }V\left(\bar{r}\right)\right\}\\ \times \{1-\frac{1}{\hbar }\left[\frac{1}{2}V^{\prime \prime }\left(\bar{r}\right)\left(\frac{\tau }{12}\Delta r+\frac{\hbar }{m}\frac{\tau^{2}}{6}\right)+\frac{1}{24}{V^{\prime \prime }}^{\prime \prime }\left(\bar{r}\right)\left(\frac{\tau }{80}{\left(\Delta r\right)}^{4}+\frac{\hbar }{m}\frac{\tau^{2}}{20}{\left(\Delta r\right)}^{2}+\frac{\hbar^{2}}{m^{2}}\frac{\tau^{3}}{10}\right)\right]\\ +\frac{1}{2\hbar^{2}}[\frac{\hbar }{m}V^{\prime }{\left(\bar{r}\right)}^{2}\frac{\tau^{3}}{12}+\frac{1}{4}V^{\prime \prime }{\left(\bar{r}\right)}^{2}\left(\frac{\tau^{2}}{144}{\left(\Delta r\right)}^{4}+\frac{\hbar }{m}\frac{\tau^{3}}{30}{\left(\Delta r\right)}^{2}+\frac{\hbar^{2}}{m^{2}}\frac{\tau^{4}}{20}\right)\\ +V^{\prime \prime \prime }\left(\bar{r}\right)V^{\prime }\left(\bar{r}\right)\left(\frac{\hbar }{m}\frac{\tau^{3}}{240}{\left(\Delta r\right)}^{2}+\frac{\hbar^{2}}{m^{2}}\frac{\tau^{4}}{60}\right)]\\ -\frac{1}{6\hbar^{3}}\left[\frac{3}{2}V^{\prime \prime }\left(\bar{r}\right)V^{\prime }{\left(\bar{r}\right)}^{2}\left(\frac{\hbar }{m}\frac{\tau^{4}}{144}{\left(\Delta r\right)}^{2}+\frac{11}{360}\frac{\hbar^{2}}{m^{2}}\tau^{5}\right)\right]\\ +\frac{1}{1152\hbar^{4}}V^{\prime }{\left(\bar{r}\right)}^{4}\frac{\hbar^{2}}{m^{2}}\tau^{6}\} \end{array}$$

Further on, we recognize that such density matrix becomes uniformly in the valence shell radius variable *r_b_* by fixing the atomic origin in *r_a_*

(266) $$r_{a}=0\Rightarrow \;\{\begin{array}{l} \bar{r}=\frac{r_{a}+r_{b}}{2}=\frac{r_{b}}{2}\\ \Delta r=r_{b}-r_{a}=r_{b} \end{array}$$

With these, the EN definition (263) acquires the atomic representation through the central field potential

(267) $$\left|V\left(\bar{r}\right)\right|=\frac{Z_{\text{eff}}}{\bar{r}}$$

where *Z_eff_* stands for the Slater effective atomic number specific for the multi-electronic atoms, being derived from the standard atomic number *Z* by subtracting the shielding effects of the inner electrons [79]. Nevertheless, worth mentioning that the usual negative sign in attractive potentials was formally abolished because:

▪ we retain the positive values of electronegativity (263) since EN is evaluated as a stability measure of such nuclear-electronic system;
▪ the sign is in accordance with the electric field orientation that drives the sense of the electronic conditional probability of the imaginary evolution amplitude evaluated from the center of atom (*r_a_* = 0) to the current valence shell radius (*r_b_*).

Therefore, the electronegativity can be seen also as *power of holding electrons in the valence shell reciprocal to that exercised upon them from the center of atom*. This way, the present EN definition and equation stand for the reconciliation of the two opposite phenomena acting upon the valence electrons: attraction to nucleus and repulsion from the other atomic inner electrons.

Next, within the Bohr description of the electrons moving in a central potential [80], while adopting the atomic units, *m* = *ħ* = *e*^2^ / 4*πε*_0_ = 1, further atomic dependency is acquired by the Bohr-Slater quantifications

(268a) $$r_{b}=\frac{n^{2}}{Z_{\text{eff}}}$$

(268b) $$\tau =2\pi \frac{n^{3}}{Z_{\text{eff}}^{2}}$$

in terms of the Slater charge *Z_eff_* and the principal quantum numbers of the atomic shell *n*. These relations are consistent with the above stipulated driven sense of the electronic evolution amplitude (or waves across the orbits), since for the center of the atom they specialize to *r_a_* = 0 and *τ* = 0 in the absence of any orbit (*n* = 0). Yet, this is the Bohr semiclassical level of the present approach.

However, for keeping the analyticity of the present approach, the computation of the integral (263) with the replacements (265)–(268) may use the saddle-point recipe, very well accommodated for the present semiclassical context; thus we implement the approximation rule [81]

(269) $$I=\;\int g\left(r_{b}\right)\text{exp}\left[f\left(r_{b}\right)\right]dr_{b}\;≅\;g\left(r_{b}^{0}\right)\text{exp}\left[f\left(r_{b}^{0}\right)\right]\sqrt{-\frac{2\pi }{f^{\prime \prime }\left(r_{b}^{0}\right)}}$$

with

(270) $$r_{b}^{0}=\frac{2\pi^{2/3}n^{2}}{Z_{\text{eff}}}$$

corresponding to the valence shell saddle radius expressed out by the optimization condition $f^{\prime }\left(r_{b}^{0}\right)=0$.

According with the exposed strategy, one finds the fourth order semiclassical expansions for electronegativity is given [72]

(271a) $$\chi_{\left[IV\right]}^{SC}(n,Z_{\text{eff}})\;≅\;\chi_{0}^{SC}+\chi_{1}^{SC}+\chi_{2}^{SC}+\chi_{3}^{SC}+\chi_{4}^{SC}$$

with the components

(271b) $$\chi_{0}^{SC}=\frac{Z_{\text{eff}}^{2}\text{exp}\left(-3n\pi^{1/3}\right)}{2\sqrt{3}\pi^{2/3}n^{2}}$$

(271c) $$\chi_{1}^{SC}=-\frac{Z_{\text{eff}}^{2}\left[12+22\pi^{1/3}n+6\pi^{2/3}n^{2}+5Z_{\text{eff}}\right]\text{exp}\left(-3n\pi^{1/3}\right)}{30\sqrt{3}\pi n^{3}}$$

(271d) $$\chi_{2}^{SC}=\frac{Z_{\text{eff}}^{2}\left(9+2\pi^{1/3}n\right)\left(6+5\pi^{1/3}n\right)\text{exp}\left(-3n\pi^{1/3}\right)}{90\sqrt{3}\pi^{2/3}n^{2}}$$

(271e) $$\chi_{3}^{SC}=\;-\;\frac{Z_{\text{eff}}^{2}\left(11+5\pi^{1/3}n\right)\text{exp}\left(-3n\pi^{1/3}\right)}{45\sqrt{3}\pi^{1/3}n}$$

(271f) $$\chi_{4}^{SC}=\frac{Z_{\text{eff}}^{2}\text{exp}\left(-3n\pi^{1/3}\right)}{36\sqrt{3}}$$

while for the chemical hardness evaluation the relationship (264) is employed to yield the expansion

(272a) $$\eta_{\left[IV\right]}^{SC}(n,Z_{\text{eff}})\;=\;{\left[\frac{\partial }{\partial Z}\chi^{SC}(n,Z)\right]}_{z\to z_{\text{eff}}}\;≅\;\eta_{0}^{SC}+\eta_{1}^{SC}+\eta_{2}^{SC}+\eta_{3}^{SC}+\eta_{4}^{SC}$$

with the components

(272b) $$\eta_{0}^{SC}=\frac{Z_{\text{eff}}\text{exp}\left(-3n\pi^{1/3}\right)}{\sqrt{3}\pi^{2/3}n^{2}}$$

(272c) $$\eta_{1}^{SC}=-\frac{Z_{\text{eff}}\left[24+44\pi^{1/3}n+12\pi^{2/3}n^{2}+15Z_{\text{eff}}\right]\text{exp}\left(-3n\pi^{1/3}\right)}{30\sqrt{3}\pi n^{3}}$$

(272d) $$\eta_{2}^{SC}=\frac{Z_{\text{eff}}\left(9+2\pi^{1/3}n\right)\left(6+5\pi^{1/3}n\right)\text{exp}\left(-3n\pi^{1/3}\right)}{45\sqrt{3}\pi^{2/3}n^{2}}$$

(272e) $$\eta_{3}^{SC}=-\frac{Z_{\text{eff}}2\left(11+5\pi^{1/3}n\right)\text{exp}\left(-3n\pi^{1/3}\right)}{45\sqrt{3}\pi^{1/3}n}$$

(272f) $$\eta_{4}^{SC}=\frac{Z_{\text{eff}}\text{exp}\left(-3n\pi^{1/3}\right)}{18\sqrt{3}}$$

Aiming to unfold the electronegativity and chemical hardness atomic scales, through applying the Equations (271) and (272), the input parameters of the Table 1, along the calibration step between the theoretically and experimentally values of the electronegativity and chemical hardness for the H atom, 7.18 eV and 6.45 eV, are employed, respectively; they provide the numerically energetic pre-factors

(273a) $$\chi^{SC}:27.21\times 251.14$$

(273b) $$\eta^{SC}:27.21\times 137.576$$

**Figure 3.**
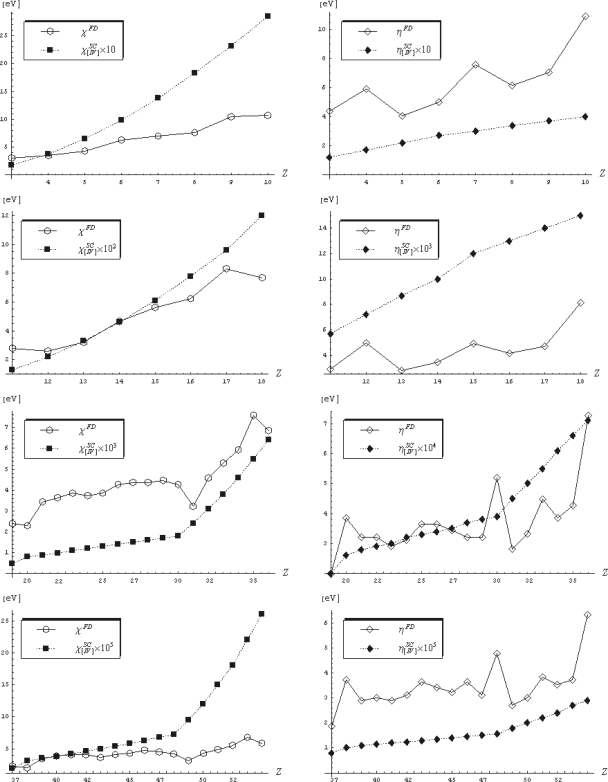
Comparative trend of rescaled fourth order semiclassical (SC) values of electronegativity (left panel) and chemical hardness (right panel) as given in Table 2 respecting their finite-difference (FD) counterparts of Table 1, for the second, third, fourth, and fifth periods of elements, from top to bottom, respectively [72].

The numerical fourth order semiclassical electronegativity and chemical hardness atomic scales are reported in Table 2 and represented in Figure 3, where the comparison with the finite-difference counterparts of Table 1 was also emphasized.

The striking difference in terms of orders of magnitudes observed between elements down groups is the main characteristic of the actual atomic scales of electronegativity and chemical hardness; however, due to the fact the actual definition of electronegativity and chemical hardness reflects the holding power with which the whole atom attracts valence electrons to its center - this is not a surprising behavior.

It is therefore natural to observe that as the atom is richer in core electrons down groups lesser is the attractive force on the outer electrons from the center of the atom. In this regard, the actual scales mirror the atomic stability of the valence shell at the best.

Nevertheless, a better regularization of their increasing trend along periods it is observed in Figure 3 for both semiclassical electronegativity and chemical hardness fourth order scales, a feature more apparent for the actual chemical hardness scale. Moreover, since the chemical hardness controls the secondary order effects throughout its definition as the derivative of the electronegativity a phenomenological rule would demand to have lower values than that of the associated electronegativity.

However, this rule, while being not always obeyed for the finite difference *η^FD^* values (*e.g.*, He, Ne, Ar, Kr, Xe) is well satisfied with the present semiclassical ones $\eta_{\left[IV\right]}^{SC}$ as compared with their counterpart electronegativities, *χ^FD^* and $\chi_{\left[IV\right]}^{SC}$, respectively.

Going to electronegativity discussion, the present $\chi_{\left[IV\right]}^{SC}$ values seem to respect almost all empirical criteria for acceptability [83]:

- the atoms N, O, F, Ne, and He have the highest electronegativities among the main groups;
- the electronegativity of N is by far greater than that of Cl - a situation that is not met in the finite-difference approach;
- the *Silicium rule* demanding that most metals to have EN values less than or equal to that of Si, is as well widely satisfied;
- the metalloid band (B, Si, Ge, As, Sb, Te) clearly separates the metals by nonmetals’ EN values;
- along periods the highest EN values belong to the noble elements – a rule not fulfilled by the couples (Cl, Ar), (Br, Kr), and (I, Xe) within the finite difference representation, see Table 1;
- the recorded electronegativity values of the chalcogens (O, S, Se, Te) reveal great distinction between the chemistry of oxygen and the rest elements of VIA group;
- the transitional metals are grouped in a distinct contracted region of EN values – this way closely emphasizing on the d-orbitals effects, a criteria almost not fulfilled by the finite-difference scheme, see Table 1 and the Figure 3.

Finally, we have to point out that the systematic decrease of orders of magnitude of electronegativity and hardness semiclassical scales of Table 2 and Figure 3 has a fundamental consequence, namely it stands as the computational proof that the electronegativity and hardness behave like pure quantum/structural indices. As such, they are not manifesting with the same intensity among all elements of the Periodic Table by having values that tend to considerably diminish as the frontier electrons are farer and feel less and less the quantum influence (potential and force) of the nucleus and of the core electrons. These results are in accordance with the electronic localization principles in an atom [3,84].

## 5. Effective Classical Path Integral of Evolution Amplitude

### 5.1. Effective Classical Partition Function

As previously shown, see Equations (121c) and (208), for instance, considering the path integral propagator that underlies the canonical density in the quantum statistical algorithm, see Equations (87)–(89), accounts for the quantum effects (fluctuations) induced on single particle paths by the presence of an external potential, while being analytically computed by averaging these over all possible configurations. Yet, one could observe that for periodic paths, *i.e.*, when the final and initial space-points coincide

(274) $$x_{a}=x_{b}$$

the particle travels in very short time not far away from the initial position and then is back on the initial point; such picture has the physical measurable consequence a particle is observed on the initial point, *i.e.*, it is found on a stationary state/orbit, while the quantum fluctuations are oscillating around the equilibrium (initial = final) space-point. Even clearer, the situation corresponds to the classical picture in which a particle behaves, being accommodated in an equilibrium state/stationary orbit under external potential influence. This means that the external influence itself is observable in (initial = final) concerned/measured state, thus being no longer a path parameterized function, but a constant:

(275) $$V\left(x(\tau ),\tau \right)\to V\left(x_{a}\right)$$

Therefore, the associated periodic propagator (density matrix) becomes

(276) $$\begin{array}{c} (x_{a}\hbar \beta ;x_{a}0)=e^{-\beta V(x_{a})}\underset{\text{FREE}\;\text{MOTION}\;\text{PROPAGATOR}}{\underset{︸}{\int_{x(0)=x_{a}}^{x(\hbar \beta )=x_{a}}D^{\prime }x(\tau )\text{exp}\left\{-\frac{1}{\hbar }\int_{0}^{\hbar \beta }\left[\frac{m}{2}{\dot{x}}^{2}(\tau )\right]d\tau \right\}}}\\ =e^{-\beta V(x_{a})}\sqrt{\frac{m}{2\pi \beta \hbar^{2}}} \end{array}$$

where the recognized path integral of free motion was solved by plugging into its quantum mechanical solution (171) the present conditions (274) and (196b), for accounting of the path periodicity and quantum statistics, respectively.

At the same time there is clear that the periodic path condition (274) is not arbitrarily but a compulsory step since characteristic in passing from density matrix to partition function and then to the real (measurable or workable) canonical and *N*-particle density, according with the density matrix algorithm (87)–(89). Therefore, the resulting partition function built from the un-normalized canonical density (276) assumes the simple form

(277) $$Z_{cl}=\int (x_{a}\hbar \beta ;x_{a}0)dx_{a}=\sqrt{\frac{m}{2\pi \beta \hbar^{2}}}\int e^{-\beta V(x_{a})}dx_{a}$$

while being susceptible of universal reliability if not limited by the degree the periodicity between the final and initial space-point is achieved through condition (274). However, looking to free motion path integral solution (171) we see that the classical observation is readily valid for the coordinate departure not exceeding the critical value

(278) $$x_{b}-x_{a}=\Delta x_{cl}=\hbar \sqrt{\frac{\beta }{m}}$$

in which case the exponential limit

(279) $$\text{exp}\left\{-\frac{m}{2\hbar^{2}\beta }{\left(x_{b}-x_{a}\right)}^{2}\right\}\overset{x_{b}-x_{a}=\Delta x_{cl}}{\to }e^{-1/2}=0.607$$

is approximated with unity in expression (276), thus with an error of 40% at the maximum displacement of (278) value; as the classical displacement (278) tends to zero as the expressions (276) and (277) become more accurate. Following the Feynman standard example, for a crystal with atoms of typical atomic mass (A) about 20, at room temperature, the classical limit of displacement (278) gives about 0.1 Å; this is the maximum displacement of those atoms around their equilibrium position in the lattice when the thermodynamic properties of the solid can be evaluated through considering the classical form of partition function (277). Just in passing worth noting that the partition function (277) is called “classical” despite carrying the exponential pre-factor with the quantum Planck constant since the configuration integral ∫exp(– *βV*) was historically anticipated and worked out by Boltzmann, in the pre-quantum era with a non-specified multiplying constant, known today as the inverse of the so called *thermal length*

(280) $$\lambda_{th}=\sqrt{\frac{2\pi \beta \hbar^{2}}{m}}$$

With these considerations there appears as natural the generalization of the classical partition form (277) into the more comprehensive one known as the effective classical partition function [85**–**90]

(281a) $$Z_{\text{eff}-cl}=\int_{-\infty }^{+\infty }\frac{dx_{0}}{\sqrt{2\pi \hbar^{2}\beta /m}}\text{exp}\left[-{\beta V}_{\text{eff}-\text{cl}}(x_{0})\right]$$

with the integration variable defined as the thermal average of the periodic quantum paths

(281b) $$x_{0}\;\equiv \;\bar{x}\;=\;\frac{1}{\hbar \beta }\int_{0}^{\hbar \beta }x\left(\tau \right)d\tau$$

sometimes called as the *Feynman centroid*, while the notation is to be right bellow justified.

Moreover, the search for the best approximation of effective-classical partition function (280) will be conducted as such the quantum fluctuations be not dependent on the classical displacement (278), abstracted from the free motion, but being driven by the quantum harmonic oscillations – through they constitute a generalization of the free motion itself, see for instance the equivalence of classical paths or propagators of free with harmonic motion in the zero-frequency limit, see the Section 3.3.3.

However, the periodicity condition (274) for paths is to be maintained and properly implemented in approximating the effective-classical partition function (281) being, nevertheless, closely and powerfully related with the quantum beloved concept of stationary orbits defined/described by periodic quantum waves/paths. This way, the effective-classical path integral approach appears as the true quantum justification of the quantum atom and of the quantum stabilization of matter in general, providing reliable results without involving observables or operators relaying on special quantum postulates other than the variational principles – with universal (classical or quantum) value.

### 5.2. Periodic Path Integrals

#### 5.2.1. Matsubara Frequencies and the Quantum Periodic Paths

As always done when a new type of path integral is under consideration the reconsideration of the quantum paths, and in fact the quantum fluctuations, is undertaken so that facilitating the best way for solving it. Yet, this time due to the periodicity condition of paths the propagator is hidden by the associated partition function. Therefore, the optimum approximation for the effective classical potential in (281) will provide the periodic evolution amplitude as well, *i.e.*, the un-normalized density, which by normalization with partition function will lead with the searched canonical density counterpart.

**Figure 4.**
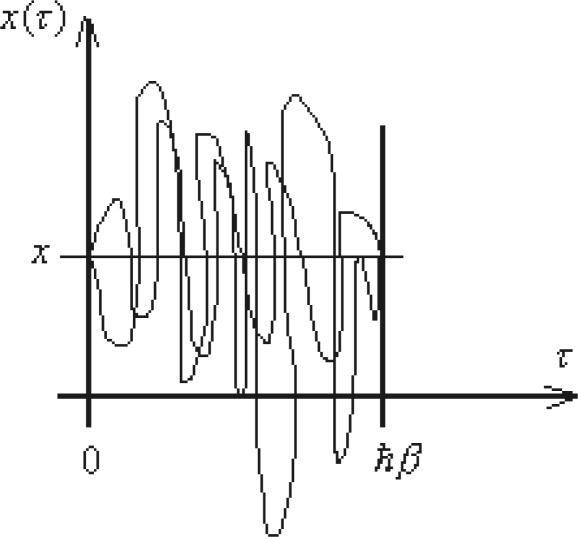
The representation of the periodic paths.

Going to characterize the periodic paths, they will be seen as the Fourier series [34,39,41,91,92]

(282a) $$x\left(\tau \right)=\sum_{m=-\infty }^{+\infty }x_{m}\text{exp}\left(i\omega_{m}\tau \right)$$

in terms of the so called Matsubara frequencies *ω_m_*; they are explicitly found through specializing the condition (274) into the actual statistical one, see Figure 4

(282b) $$x_{a}=x(0)=x(\hbar \beta )=x_{b}$$

resulting in the equality

(282c) $$1=\text{exp}(i\omega_{m}\hbar \beta )$$

with the solution

(283) $$\omega_{m}=\frac{2\pi }{\hbar \beta }m,m\in \text{Z}$$

which certifies the quantization of the paths (282a).

Moreover, under the condition the quantum paths (282a) are real

(284) $$x^{*}(\tau )=x(\tau )$$

the equivalent expanded form with the conjugated path

(285) $$x^{*}(\tau )=\sum_{m=-\infty }^{+\infty }x_{m}^{*}\text{exp}(-i\omega_{m}\tau )=\sum_{m=-\infty }^{+\infty }x_{m}\text{exp}(i\omega_{m}\tau )$$

yields for the coefficients of the periodical paths the relationship

(286) $$x_{m}^{*}=x_{-m}=x_{m}$$

With this, the quantified form of periodic path frequencies, Equation (283), allows separating the paths (282a) into the constant and complex conjugated oscillating contributions

(282d) $$\begin{array}{c} x\left(\tau \right)=x_{0}+\sum_{m=1}^{+\infty }x_{m}\text{exp}\left(i\omega_{m}\tau \right)+\sum_{m=-1}^{-\infty }x_{m}\text{exp}\left(i\omega_{m}\tau \right)\\ =x_{0}\;+\;\sum_{m=1}^{+\infty }x_{m}\text{exp}(i\omega_{m}\tau )+\sum_{m=1}^{+\infty }x_{-m}\text{exp}\left(-i\omega_{m}\tau \right)\\ =x_{0}+\sum_{m=1}^{+\infty }x_{m}\text{exp}\left(i\omega_{m}\tau \right)+c.c. \end{array}$$

with the 0^th^ terms viewed more than the “zero-oscillating” or free motion path but the thermal averaged path over entire quantum paths (282a)

(282e) $$\begin{array}{c} \frac{1}{\hbar \beta }\int_{0}^{\hbar \beta }x\left(\tau \right)d\tau =\frac{1}{\hbar \beta }\int_{0}^{\hbar \beta }\left[x_{0}+\sum_{m=1}^{+\infty }x_{m}\text{exp}\left(i\omega_{m}\tau \right)+c.c.\right]d\tau \\ =x_{0}+\sum_{m=1}^{+\infty }x_{m}\frac{1}{\hbar \beta }\int_{0}^{\hbar \beta }\text{exp}\left(i\omega_{m}\tau \right)d\tau +\sum_{m=1}^{+\infty }x_{-m}\frac{1}{\hbar \beta }\int_{0}^{\hbar \beta }\text{exp}\left(-i\omega_{m}\tau \right)d\tau \\ =x_{0}+\frac{1}{\hbar \beta }\sum_{m=1}^{+\infty }x_{m}\int_{0}^{\hbar \beta }\left[\text{exp}\left(i\omega_{m}\tau \right)+\text{exp}\left(-i\omega_{m}\tau \right)\right]d\tau \\ =x_{0}+\frac{2}{\hbar \beta }\sum_{m=1}^{+\infty }x_{m}\int_{0}^{\hbar \beta }\text{cos}\left(\omega_{m}\tau \right)d\tau \\ =x_{0}+\frac{2}{\hbar \beta }\sum_{m=1}^{+\infty }\frac{x_{m}}{\omega_{m}}\text{sin}\left(\omega_{m}\hbar \beta \right)\\ \underset{0}{\underset{︸}{=x_{0}+\frac{2}{\hbar \beta }\sum_{m=1}^{+\infty }\frac{x_{m}}{\omega_{m}}\underset{0}{\underset{︸}{\text{sin}{\left(2\pi m\right)}_{m\in \text{Z}}}}}} \end{array}$$

thus resulting in the Feynman centroid formula (281b).

However, beside revealing the integration variable of the classical partition function (281a) as being of averaged nature, the result (282e) emphasizes on the actual periodic path decomposition (282d) integral featuring another level for parameterization of quantum paths that goes beyond characterizing them as quantum fluctuations around classical motion; they are here constructed as periodic oscillations (back and forth – due to their complex form, in analogy with conjugated plane waves traveling in opposite directions) around the averaged path value (interpreted as thermic average, or, more plastic, as centroid of the quantum fluctuations themselves). Therefore, with such path parameterization perspective the present level seems involving quite complex quantum phenomenology to be further enriched in the sections to follow.

#### 5.2.2. Matsubara Harmonic Partition Function

The quantum path decomposition (282d) imposes the factorization of the Feynman path integral measure (120) accordingly

(287) $$\int_{x(0)=x(\hbar \beta )}D^{\prime \prime }x\left(\tau \right)\equiv \left(C_{0}\int_{-\infty }^{+\infty }dx_{0}\right)\left[\prod_{m=1}^{\infty }C_{m}\int_{-\infty }^{+\infty }d\text{Re}\left(x_{m}\right)\int_{-\infty }^{+\infty }d\text{Im}\left(x_{m}\right)\right]$$

with the integration constants *C*_0_ and *C_m_* to be determined from identifying the known partition function of the harmonic oscillator, Equation (192), with the path integral representation of the counterpart partition function written by the measure (287) and paths (282)

(288a) $$\begin{array}{c} Z_{\Omega }=\int_{x(0)=x(\hbar \beta )}D^{\prime \prime }x\left(\tau \right)\text{exp}\left\{-\frac{1}{\hbar }\int_{0}^{\hbar \beta }d\tau \left[\frac{m}{2}{\dot{x}}^{2}\left(\tau \right)+\frac{m}{2}\Omega^{2}x^{2}\left(\tau \right)\right]\right\}\\ =\left(C_{0}\int_{-\infty }^{+\infty }dx_{0}\right)\left[\prod_{m=1}^{\infty }C_{m}\int_{-\infty }^{+\infty }d\text{Re}\left(x_{m}\right)\int_{-\infty }^{+\infty }d\text{Im}\left(x_{m}\right)\right]\text{exp}\left\{-\frac{1}{\hbar }\int_{0}^{\hbar \beta }d\tau \left[\frac{m}{2}{\dot{x}}^{2}\left(\tau \right)+\frac{m}{2}\Omega^{2}x^{2}\left(\tau \right)\right]\right\} \end{array}$$

(288b) $$\overset{!}{=}\frac{1}{2\text{sinh}\left(\hbar \beta \Omega /2\right)}$$

To this end, let’s begin with the computation of the kinetic term appearing under the integral (288a)

(289a) $$\begin{array}{c} \frac{m}{2}\int_{0}^{\hbar \beta }d\tau {\dot{x}}^{2}\left(\tau \right)=\frac{m}{2}\int_{0}^{\hbar \beta }d\tau \left[\sum_{m=-\infty }^{+\infty }ix_{m}\omega_{m}\text{exp}\left(i\omega_{m}\tau \right)\right]\left[\sum_{m^{\prime }=-\infty }^{+\infty }ix_{m^{\prime }}\omega_{m^{\prime }}\text{exp}\left(i\omega_{m^{\prime }}\tau \right)\right]\\ =-\frac{m}{2}\sum_{m=-\infty }^{+\infty }\sum_{m^{\prime }=-\infty }^{+\infty }x_{m}x_{m^{\prime }}\omega_{m}\omega_{m^{\prime }}\int_{0}^{\hbar \beta }d\tau \text{exp}\left[i\left(\omega_{m}+\omega_{m^{\prime }}\right)\tau \right]\\ =-\frac{m}{2}\hbar \beta \sum_{m=-\infty }^{+\infty }\sum_{m^{\prime }=-\infty }^{+\infty }x_{m}x_{m^{\prime }}\omega_{m}\omega_{m^{\prime }}\delta_{m+m^{\prime },0}\\ =-\frac{m}{2}\hbar \beta \sum_{m=-\infty }^{+\infty }x_{m}x_{-m}\omega_{m}\omega_{-m}\\ \overset{\omega_{-m}={-\omega }_{m}}{=}\frac{m}{2}\hbar \beta \sum_{m=-\infty }^{+\infty }\omega_{m}^{2}{\left|x_{m}\right|}^{2}\\ =m\hbar \beta \sum_{m=1}^{+\infty }\omega_{m}^{2}\left[{\left(\text{Re}x_{m}\right)}^{2}{\left(\text{Im}x_{m}\right)}^{2}\right] \end{array}$$

while for the harmonic contribution we similarly get

(289b) $$\begin{array}{c} \frac{m}{2}\Omega^{2}\int_{0}^{\hbar \beta }d\tau x^{2}\left(\tau \right)=\frac{m}{2}\Omega^{2}\int_{0}^{\hbar \beta }d\tau \left[\sum_{m=-\infty }^{+\infty }x_{m}\text{exp}\left(i\omega_{m}\tau \right)\right]\left[\sum_{m^{\prime }=-8}^{+\infty }x_{m^{\prime }}\text{exp}\left(i\omega_{m^{\prime }}\tau \right)\right]\\ =\frac{m}{2}\Omega^{2}\sum_{m=-\infty }^{+\infty }\sum_{m^{\prime }=-\infty }^{+\infty }x_{m}x_{m^{\prime }}\int_{0}^{\hbar \beta }d\tau \text{exp}\left[i\left(\omega_{m}+\omega_{m^{\prime }}\right)\tau \right]\\ =\frac{m}{2}\Omega^{2}\sum_{m=-\infty }^{+\infty }\sum_{m^{\prime }=-\infty }^{+\infty }x_{m}x_{m^{\prime }}\hbar \beta \delta_{m+m^{\prime },0}\\ =\frac{m}{2}\Omega^{2}\hbar \beta \sum_{m=-\infty }^{+\infty }x_{m}x_{-m}\\ =\frac{m}{2}\Omega^{2}\hbar \beta \sum_{m=-\infty }^{+\infty }x_{m}x_{m}^{*}\\ =\frac{m}{2}\Omega^{2}\hbar \beta \left\{x_{0}^{2}+2\sum_{m=1}^{+\infty }\left[{\left(\text{Re}x_{m}\right)}^{2}+{\left(\text{Im}x_{m}\right)}^{2}\right]\right\} \end{array}$$

all together be combined in the partition function (288a)

(290) $$\begin{array}{c} Z_{\Omega }=\left(C_{0}\int_{-\infty }^{+\infty }{dx}_{0}\right)\left[\prod_{m=1}^{\infty }C_{m}\int_{-\infty }^{+\infty }d\text{Re}\left(x_{m}\right)\int_{-\infty }^{+\infty }d\text{Im}\left(x_{m}\right)\right]\\ \times \text{exp}\left\{-m\beta \sum_{m=1}^{+\infty }\omega_{m}^{2}\left[{\left(\text{Re}x_{m}\right)}^{2}+{\left(\text{Im}x_{m}\right)}^{2}\right]-\frac{m}{2}\Omega^{2}\beta x_{0}^{2}-m\Omega^{2}\beta \sum_{m=1}^{+\infty }\left[{\left(\text{Re}x_{m}\right)}^{2}+{\left(\text{Im}x_{m}\right)}^{2}\right]\right\}\\ =\left[C_{0}\int_{-\infty }^{+\infty }dx_{0}\text{exp}\left(-\frac{m}{2}\Omega^{2}\beta x_{0}^{2}\right)\right]\prod_{m=1}^{\infty }C_{m}{\left\{\int_{-\infty }^{+\infty }d\text{Re}\left(x_{m}\right)\text{exp}\left[-m\beta \sum_{m=1}^{+\infty }\left(\omega_{m}^{2}+\Omega^{2}\right){\left(\text{Re}x_{m}\right)}^{2}\right]\right\}}^{2}\\ =C_{0}\sqrt{\frac{2\pi }{m\beta \Omega^{2}}}\prod_{m=1}^{\infty }C_{m}\frac{\pi }{m\beta \left(\omega_{m}^{2}+\Omega^{2}\right)} \end{array}$$

Next, with the frequency choice

(291) $$C_{m}=C\omega_{m}^{2}$$

the partition function (290) further resumes as

(292) $$Z_{\Omega }=C_{0}\frac{1}{\Omega }\sqrt{\frac{2\pi }{m\beta }}\frac{\pi }{m\beta }Cf\left(\Omega \right)$$

noting the newly introduced function

(293a) $$f\left(\Omega \right)=\prod_{m=1}^{\infty }\frac{\omega_{m}^{2}}{\omega_{m}^{2}+\Omega^{2}}$$

like a series of Matsubara frequencies. Therefore, it remains that in order the partition function (292) be solved the product series (293a) has to be evaluated; this new issue may be accomplished through three more transformations, namely, by firstly rewriting the series (293a) as

(293b) f(Ω)=exp[g(Ω)]

With:

(294) $$g\left(\Omega \right)=\sum_{m=1}^{\infty }\text{ln}\frac{\omega_{m}^{2}}{\omega_{m}^{2}+\Omega^{2}}$$

followed by considering its derivative

(295) $$g^{\prime }\left(\Omega \right)=\sum_{m=1}^{\infty }\frac{\omega_{m}^{2}+\Omega^{2}}{\omega_{m}^{2}}\frac{-\omega_{m}^{2}}{{\left(\omega_{m}^{2}+\Omega^{2}\right)}^{2}}2\Omega =-2\Omega R\left(\Omega \right)$$

leaving with the final evaluation of the Riemann generalized series

(296) $$R\left(\Omega \right)=\sum_{m=1}^{\infty }\frac{1}{\Omega^{2}+\omega_{m}^{2}}$$

Going back, once calculated, the Riemann series (296) is replaced in (295) which, at its turn, is employed for the integral evaluation

(297) $$g\left(\Omega \right)=g\left(0\right)+\int_{0}^{\Omega }d\tilde{\Omega }g^{\prime }\left(\tilde{\Omega }\right)$$

for ending with unfolding of the searched function (293). These steps will be systematically exposed in next sections.

#### 5.2.3. The Generalized Riemann’ Series

**Figure 5.**
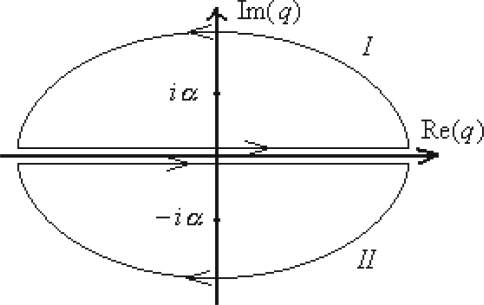
The integration outlines around the poles *q* = ±*iα*.

Computation of the generalized Riemann series (296) requires few intermediate operations:

○ Writing it under the form
  (298a) $$R\left(\Omega \right)=\frac{1}{2}\left[F\left(\Omega \right)-\frac{1}{\Omega^{2}}\right]$$
  in terms of the extended series
  (298b) $$F\left(\Omega \right)=\sum_{m=-\infty }^{\infty }\frac{1}{\Omega^{2}+\omega_{m}^{2}}=\frac{\hbar^{2}\beta^{2}}{4\pi^{2}}\sum_{m=-\infty }^{\infty }\frac{1}{\alpha^{2}+m^{2}},\alpha \equiv \frac{\hbar^{2}\beta^{2}\Omega^{2}}{4\pi^{2}}$$
○ Applying the Poisson formula, see Appendix (A8), on series (298b)
  (299) $$F\left(\Omega \right)=\frac{\hbar^{2}\beta^{2}}{4\pi^{2}}\sum_{n=-\infty }^{\infty }\left[\int_{-\infty }^{+\infty }dq\frac{1}{q^{2}+\alpha^{2}}\text{exp}\left(-i2\pi qn\right)\right]$$
○ Computing the integral under the sum of (299) by the complex integration, according with the contours of integration identified in Figure 5 around the poles *q* = ±*iα* throughout applying the residues’ theorem
  (300) $$\oint_{C\left(z_{0}\right)}f(z)dz=2\pi i\underset{z=z_{0}}{\text{Rez}}f(z)=2\pi i\underset{z->z_{0}}{\text{lim}}(z-z_{0})f(z)$$
  while summing upon the convergent cases
  (301a) $$\begin{array}{l} n\geq 0\Rightarrow \text{Im}q<0\Rightarrow \text{contour}\text{II}\\ n<0\Rightarrow \text{Im}q>0\Rightarrow \text{contour}I \end{array}$$
  arisen from the observation that
  (301b) |exp[−i2π(Req+iImq)n]|=exp(2πnImq)
  Note that the contour (*I*) is considered completed with trigonometric positive direction, while for the (*II*) contour the anti-trigonometric sense results, as being equivalent with the minus sign in front of its integral, which, explicitly gives
  (302) $$\begin{array}{c} \int_{-\infty (II)}^{+\infty }dq\frac{1}{q^{2}+\alpha^{2}}\text{exp}(-i2\pi qn)\\ =(-1)\oint_{C(II)}dq\frac{1}{q^{2}+\alpha^{2}}\text{exp}(-i2\pi qn)=-2\pi i\underset{q=-ia}{\text{Rez}}\frac{\text{exp}(-i2\pi qn)}{q^{2}+\alpha^{2}}\\ =-2\pi i\underset{\begin{array}{c} q=-ia\\ \alpha =\frac{\hbar \beta \Omega }{2\pi } \end{array}}{\text{lim}}(q+i\alpha )\frac{\text{exp}(-i2\pi qn)}{\left(q+i\alpha )(q-i\alpha \right)}=\frac{2\pi^{2}}{\hbar \beta \Omega }\text{exp}(-\hbar \beta \Omega n) \end{array}$$
  while the other contour integration leads with similar result.
○ Insertion of these integration results in the expression (299) is done by attributing to each contour and integration the (series) summing range according with the constraints of (301a) to successively yield
  (303) $$\begin{array}{c} F(\Omega )=\frac{\hbar^{2}\beta^{2}}{4\pi^{2}}\left[\sum_{n=1}^{\infty }\int_{-\infty (I)}^{+\infty }dq\frac{\text{exp}(i2\pi qn)}{q^{2}+\alpha^{2}}+\sum_{n=0}^{\infty }\int_{-\infty (II)}^{+\infty }dq\frac{\text{exp}(-i2\pi qn)}{q^{2}+\alpha^{2}}\right]\\ =\frac{\hbar^{2}\beta^{2}}{4\pi^{2}}\frac{2\pi^{2}}{\hbar \beta \Omega }\left[\sum_{n=1}^{\infty }\text{exp}(-\hbar \beta \Omega n)+\sum_{n=0}^{\infty }\text{exp}(-\hbar \beta \Omega n)\right]\\ =\frac{\hbar \beta }{2\Omega }\left\{2\sum_{n=0}^{\infty }{\left[\text{exp}(-\hbar \beta \Omega )\right]}^{n}-1\right\}\\ =\frac{\hbar \beta }{2\Omega }\text{coth}\left(\frac{\hbar \beta \Omega }{2}\right) \end{array}$$
  ○ With expression (303) the Riemann series (298a) finally reads as
  (304) $$R(\Omega )=\sum_{m=1}^{\infty }\frac{1}{\Omega^{2}+\omega_{n}^{2}}=\frac{\hbar^{2}\beta^{2}}{4\pi^{2}}\sum_{m=1}^{\infty }\frac{1}{{\left(\hbar \beta \Omega /2\right)}^{2}+m^{2}}=\frac{1}{4}\frac{\hbar \beta }{\Omega }\left[\text{coth}\left(\frac{\hbar \beta \Omega }{2}\right)-\frac{2}{\hbar \beta \Omega }\right]$$
○ The cross-check with the usual Riemann series is performed by means of turning the harmonic to free motion picture, as the already consecrated free to harmonic motion interplay; That is to evaluate the limit
  (305) $$\underset{\Omega \to 0}{\text{lim}}R\left(\Omega \right)=\frac{\hbar^{2}\beta^{2}}{4\pi^{2}}\sum_{m=1}^{\infty }\frac{1}{m^{2}}=\underset{\Omega \to 0}{\text{lim}}\frac{1}{4}\frac{\hbar \beta }{\Omega }\left[\text{coth}\left(\frac{\hbar \beta \Omega }{2}\right)-\frac{2}{\hbar \beta \Omega }\right]$$
  within the hyperbolic cotangent function approximation
  (306) $${\left[\text{coth}\left(\Xi \right)\right]}_{\Xi \to 0}≅\frac{1}{\Xi }+\frac{\Xi }{3}$$
  to give the identity
  (307) $$\frac{\hbar^{2}\beta^{2}}{4\pi^{2}}\sum_{m=1}^{\infty }\frac{1}{m^{2}}=\frac{1}{4}\frac{\hbar^{2}\beta^{2}}{6}$$
  leaving with the classical Riemann series limit
  (308) $$\sum_{m=1}^{\infty }\frac{1}{m^{2}}=\frac{\pi^{2}}{6}$$

We have now clarified all prerequisites to readily compute the Matsubara harmonic partition function (292), used as a tool to find out the Matsubara normalization of periodic path integrals. This will be addressed in the sequel.

#### 5.2.4. Periodic Path Integral Measure

The Matsubara harmonic partition function algorithm (292)–(297) may be now unfolded successively as:

○ Computing the function (295) by inserting the above Riemann generalized series (304):
  (309) $$g^{\prime }\left(\Omega \right)=-2\Omega R\left(\Omega \right)=-\frac{\hbar \beta }{2}\left[\text{coth}\left(\frac{\hbar \beta \Omega }{2}\right)-\frac{2}{\hbar \beta \Omega }\right]$$
○ Evaluating the function (294) by the aid of (297) rule through considering the variable change *z* = *ħβ*Ω/ 2 in (309)
  (310) $$\begin{array}{c} g\left(\Omega \right)=-\int_{0}^{\hbar \beta \Omega /2}dz\left(\text{coth}z-\frac{1}{z}\right)=-{\left[\text{ln}\left(\text{sinh}z\right)-\text{ln}z\right]}_{0}^{\hbar \beta \Omega /2}=-{\left[\text{ln}\left(\frac{\text{sinh}z}{z}\right)\right]}_{0}^{\hbar \beta \Omega /2}\\ =\text{ln}\left[\frac{\hbar \beta \Omega }{2\text{sinh}\left(\hbar \beta \Omega /2\right)}\right] \end{array}$$
○ Obtaining the function (293a) with the help of (293b) and (310)
  (311) $$f\left(\Omega \right)=\prod_{m=1}^{\infty }\frac{\omega_{m}^{2}}{\omega_{m}^{2}+\Omega^{2}}=\text{exp}\left[g\left(\Omega \right)\right]=\frac{\hbar \beta \Omega }{2\text{sinh}\left(\hbar \beta \Omega /2\right)}$$
○ Releasing the Matsubara partition function for the harmonic motion by replacing function (311) into expression (292)
  (312) $$Z_{\Omega }=C_{0}C\sqrt{\frac{2\pi }{m\beta }}\frac{\pi \hbar }{m}\frac{1}{2\text{sinh}\left(\hbar \beta \Omega /2\right)}$$
○ Comparing the form (312) with the consecrated results (192) or (288b), thus getting the condition
  (313) $$1=C_{0}C\sqrt{\frac{2\pi }{m\beta }}\frac{\pi \hbar }{m}$$
○ Choosing for the Feynman centroid normalization factor the inverse of the thermal length (280)
  (314) $$C_{0}=\frac{1}{\lambda_{th}}=\frac{1}{\sqrt{2\pi \hbar^{2}\beta /m}}$$
○ Plugging expression (314) in (313) to yield the constant
  (315a) $$C=\frac{\beta m}{\pi }$$
  and then by considering it into the relation (291) to get the Matsubara constants
  (315b) $$C_{m}=C\omega_{m}^{2}=\frac{\beta m_{0}\omega_{m}^{2}}{\pi }$$
○ Replacing the constants (314) and (315b) in (287) to provide the normalized measure of the periodic integrals in terms of the Matsubara quantum frequencies (283)

(316) $$\int_{x\left(0\right)=x\left(\hbar \beta \right)}D^{\prime \prime }x\left(\tau \right)\equiv \left(\int_{-\infty }^{+\infty }\frac{dx_{0}}{\sqrt{2\pi \hbar^{2}\beta /m}}\right)\left[\prod_{m=1}^{\infty }\int_{-\infty }^{+\infty }\int_{-\infty }^{+\infty }\frac{d\text{Re}x_{m}d\text{Im}x_{m}}{\pi /\left(m\beta \omega_{m}^{2}\right)}\right]$$

Note that the measure given in (316) is rather universal for periodic paths, while the involvement of the harmonic oscillator was only a tool (and always an inspiring exercise) for determining it since the complete quantum and statistical solution at hand. As such, once more, the harmonic motion proofs its versatile properties respecting the fluctuation over – or perturbation of – the free motion by modeling the quantum displacements from classical equilibrium or observed path.

### 5.3. Feynman-Kleinert Variational Formalism

#### 5.3.1. Feynman-Kleinert Partition Function

Being equipped with the periodic path integral technique we can present one of the most efficient ways for approximate the effective-classical partition function (281a); it starts with the general path integral

(317) $$\begin{array}{c} Z=\int_{x\left(0\right)=x\left(\hbar \beta \right)}D^{\prime \prime }x\left(\tau \right)\text{exp}\left\{-\frac{1}{\hbar }S^{+}\left[x,\dot{x},\tau \right]\right\}\\ =\int_{x\left(0\right)=x\left(\hbar \beta \right)}D^{\prime \prime }x\left(\tau \right)\text{exp}\left\{-\frac{1}{\hbar }\int_{0}^{\hbar \beta }d\tau \left[m\frac{{\dot{x}}^{2}\left(\tau \right)}{2}+V\left(x\left(\tau \right)\right)\right]\right\}\\ =\left(\int_{-\infty }^{+\infty }\frac{dx_{0}}{\sqrt{2\pi \hbar^{2}\beta /m}}\right)\left[\prod_{m=1}^{\infty }\int_{-\infty }^{+\infty }\int_{-\infty }^{+\infty }\frac{d\text{Re}x_{m}d\text{Im}x_{m}}{\pi /\left(m\beta \omega_{m}^{2}\right)}\right]\text{exp}\left[-\beta m\sum_{m=1}^{\infty }\omega_{m}^{2}{\left|x_{m}\right|}^{2}-\frac{1}{\hbar }\int_{0}^{\hbar \beta }d\tau V\left(x\left(\tau \right)\right)\right] \end{array}$$

Since the analytical solution for expression (317) is hard to be conceived for an unspecified potential form, it may be eventually reformulated in a workable from by involving another partition function, the so called *Feynman-Kleinert partition function Z_FK_*

(318a) $$\begin{array}{c} Z=\int_{x\left(0\right)=x\left(\hbar \beta \right)}D^{\prime \prime }x\left(\tau \right)\text{exp}\left\{-\frac{1}{\hbar }S_{FK}^{+}\left[x,\dot{x},\tau \right]\right\}\text{exp}\left\{-\frac{1}{\hbar }\left(S^{+}\left[x,\dot{x},\tau \right]-S_{FK}^{+}\left[x,\dot{x},\tau \right]\right)\right\}\\ =Z_{FK}{\langle \text{exp}\left\{-\frac{1}{\hbar }\left(S^{+}\left[x,\dot{x},\tau \right]-S_{FK}^{+}\left[x,\dot{x},\tau \right]\right)\right\}\rangle }_{FK} \end{array}$$

and its special average recipe

(318b) $${\langle O\left[x\right]\rangle }_{FK}=Z_{FK}^{-1}\int_{x\left(0\right)=x\left(\hbar \beta \right)}D^{\prime \prime }x\left(\tau \right)O\left[x\right]\text{exp}\left\{-\frac{1}{\hbar }S_{FK}^{+}\left[x,\dot{x},\tau \right]\right\}$$

In Equations (318a) and (318b) the Feynman-Kleinert partition function writes as [85]

(319a) $$Z_{FK}=\int_{x\left(0\right)=x\left(\hbar \beta \right)}D^{\prime \prime }x\left(\tau \right)\text{exp}\left\{-\frac{1}{\hbar }S_{FK}^{+}\left[x,\dot{x},\tau \right]\right\}$$

with the working action ansatz

(319b) $$S_{FK}^{+}\left[x,\dot{x},\tau \right]=\int_{0}^{\hbar \beta }d\tau \left[m\frac{{\dot{x}}^{2}\left(\tau \right)}{2}+\frac{m}{2}\Omega^{2}\left(x_{0}\right){\left(x\left(\tau \right)-x_{0}\right)}^{2}+L_{FK}\left(x_{0}\right)\right]$$

The Feynman-Kleinert partition function is constructed as such, unlike the general partition function (317), to explicitly account for the path fluctuations around the Feynman centroid (281b) through the term (*x*(*τ*) − *x*_0_)^2^, driven harmonically by the frequency Ω^2^ (*x*_0_), with a role in optimizing the quantum fluctuations in order state equilibrium be achieved. The supplementary Feynman-Kleinert perturbation function *L_FK_* (*x*_0_) assures the global optimization for the action, and implicitly for the Feynman-Kleinert partition function, so approaching at the best the exact partition function (317) and its associate total ground state energy of the system given by the free energy

(320) $$F=-\beta^{-1}\text{ln}Z$$

In fact, the Feynman-Kleinert action (319b) is to be involved in two-fold optimization algorithm for providing the best approximation of the partition function (317). This will favor a close analogy with the double search for electronic density, in density functional theory (DFT), as will be discussed later.

Yet, the Feynman-Kleinert partition function is to be unfolded within the actual periodic path integral representation

(321) $$\begin{array}{c} Z_{FK}=\int_{x\left(0\right)=x\left(\hbar \beta \right)}D^{\prime \prime }x\left(\tau \right)\text{exp}\left\{-\frac{1}{\hbar }\int_{0}^{\hbar \beta }d\tau \left[m\frac{{\dot{x}}^{2}\left(\tau \right)}{2}+\frac{m}{2}\Omega^{2}\left(x_{0}\right){\left(x\left(\tau \right)-x_{0}\right)}^{2}+L_{FK}\left(x_{0}\right)\right]\right\}\\ =\left(\int_{-\infty }^{+\infty }\frac{dx_{0}\text{exp}\left[-\beta L_{FK}\left(x_{0}\right)\right]}{\sqrt{2\pi \hbar^{2}\beta /m}}\right)\underset{f\left(\Omega \right)\;see\;eqs.\left(292\right)\&\left(311\right)}{\underset{︸}{\left[\prod_{m=1}^{\infty }\int_{-\infty }^{+\infty }\int_{-\infty }^{+\infty }\frac{d\text{Re}x_{m}d\text{Im}x_{m}}{\pi /\left(m\beta \omega_{m}^{2}\right)}\right]\text{exp}\left\{-m\beta \sum_{m=1}^{\infty }\left[\omega_{m}^{2}+\Omega^{2}\left(x_{0}\right)\right]{\left|x_{m}\right|}^{2}\right\}}}\\ =\int_{-\infty }^{+\infty }\frac{dx_{0}}{\sqrt{2\pi \hbar^{2}\beta /m}}\frac{\hbar \beta \Omega \left(x_{0}\right)/2}{\text{sinh}\left(\hbar \beta \Omega \left(x_{0}\right)/2\right)}\text{exp}\left[-\beta L_{FK}\left(x_{0}\right)\right] \end{array}$$

while being resumed under the effective-classical form (281a)

(322) $$Z_{FK}=\int_{-\infty }^{+\infty }\frac{dx_{0}}{\sqrt{2\pi \hbar^{2}\beta /m}}\text{exp}\left[-\beta W_{FK}\left(x_{0}\right)\right]$$

by means of the Feynman-Kleinert potential [85]

(323) $$W_{FK}\left(x_{0}\right)=\frac{1}{\beta }\text{ln}\left[\frac{\text{sinh}\left(\hbar \beta \Omega \left(x_{0}\right)/2\right)}{\hbar \beta \Omega \left(x_{0}\right)/2}\right]+L_{FK}\left(x_{0}\right)$$

to be optimized respecting its harmonic frequency (for equilibrium optimization) and for ground state perturbation (optimization) in what follows.

#### 5.3.2. Feynman-Kleinert Optimum Potential

**Figure 6.**
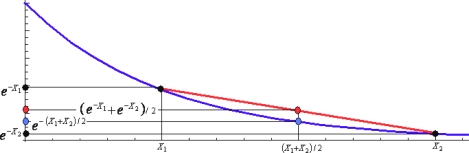
Graphical illustration of the Jensen-Peierls averages’ inequality of Equation (324).

The optimization of the Feynman-Kleinert partition function (322) is performed employing the Jensen-Peierls inequality

(324) 〈exp[O]〉≥exp[〈O〉]

whose the phenomenological proof is given in the Figure 6, on the partition function relationship (318a) leading to the lower bounded partition function

(325) $$Z\geq Z_{FK}\text{exp}\left\{-\frac{1}{\hbar }{\langle S^{+}\left[x,\dot{x},\tau \right]-S_{FK}^{+}\left[x,\dot{x},\tau \right]\rangle }_{FK}\right\}$$

or, by calling the Equation (320), to the higher bounded free energy

(326) $$F\leq F_{FK}+\frac{1}{\hbar \beta }{\langle S^{+}\left[x,\dot{x},\tau \right]-S_{FK}^{+}\left[x,\dot{x},\tau \right]\rangle }_{FK}$$

When rewritten the last inequality with the help of Euclidian actions for general partition function and the Feynman-Kleinert specialization, Equations (317) and (319), respectively, one notes the cancellation of the kinetic (free motion) terms, while the resulting expression

(327) $$F\leq F_{FK}+\frac{1}{\hbar \beta }\int_{0}^{\hbar \beta }d\tau {\langle V\left(x\left(\tau \right)\right)-\frac{m}{2}\Omega^{2}\left(x_{0}\right){\left(x\left(\tau \right)-x_{0}\right)}^{2}-L_{FK}\left(x_{0}\right)\rangle }_{FK}$$

provides the searched variational architecture

(328) $$\delta F=0\Rightarrow {\langle V\left(x\left(\tau \right)\right)-\frac{m}{2}\Omega^{2}\left(x_{0}\right){\left(x\left(\tau \right)-x_{0}\right)}^{2}-L_{FK}\left(x_{0}\right)\rangle }_{FK}=0$$

where all involved terms combine the external, perturbation and quantum fluctuation influences.

Having the variational problem formulated it remains to individually compute the terms appearing in the Feynman-Kleinert average (328), by using the associate definition (318b) with the action (319b).

Going to evaluate the most general term containing the external potential average, we have in the first instance its periodic path integral representation

(329) $$\begin{array}{c} {\langle V(x(\tau ))\rangle }_{FK}=Z_{FK}^{-1}\int_{x(0)=x(\hbar \beta )}D\prime \prime x(\tau )V(x(\tau ))\text{exp}\left\{-\frac{1}{\hbar }S_{FK}^{+}[x,\dot{x},\tau ]\right\}\\ =Z_{FK}^{-1}\left(\int_{-\infty }^{+\infty }\frac{dx_{0}\text{exp}[-\beta l_{FK}(x_{0})]}{\sqrt{2\pi \hbar^{2}\beta /m}}\right)\left[\prod_{m=1}^{\infty }\int_{-\infty }^{+\infty }\int_{-\infty }^{+\infty }\frac{d\text{Re}x_{m}d\text{Im}x_{m}}{\pi /\left(m\beta \omega_{m}^{2}\right)}\right]\text{exp}\left\{-m\beta \sum_{m=1}^{\infty }\left[\omega_{m}^{2}+\Omega^{2}+(x_{0})\right]{x_{m}|}^{2}\right\}\\ \times \int_{-\infty }^{+\infty }\frac{dk}{2\pi }V(k)\text{exp}\left\{ik\left[x_{0}+\sum_{n=1}^{\infty }x_{n}\text{exp}(-i\omega_{n}\tau )+c.c.\right]\right\}\\ =Z_{FK}^{-1}\int_{-\infty }^{+\infty }\frac{dx_{0}\text{exp}\left[-\beta L_{FK}(x_{0})\right]}{\sqrt{2\pi \hbar^{2}\beta /m}}\int_{-\infty }^{+\infty }\frac{dk}{2\pi }V(k)\text{exp}(ikx_{0})\\ \times \left[\prod_{m=1}^{\infty }\int_{-\infty }^{+\infty }\int_{-\infty }^{+\infty }\frac{d\text{Re}x_{m}d\text{Im}x_{m}}{\pi /\left(m\beta \omega_{m}^{2}\right)}\right]\text{exp}\left\{-m\beta \sum_{m=1}^{\infty }\left[\omega_{m}^{2}+\Omega^{2}(x_{0})\right]{x_{m}|}^{2}+ik\left[\sum_{n=1}^{\infty }x_{n}\text{exp}(-i\omega_{n}\tau )+c.c.\right]\right\}\\ =Z_{FK}^{-1}\int_{-\infty }^{+\infty }\frac{dx_{0}\text{exp}\left[-\beta L_{FK}(x_{0})\right]}{\sqrt{2\pi \hbar^{2}\beta /m}}\int_{-\infty }^{+\infty }\frac{dk}{2\pi }V(k)\text{exp}\left[ikx_{0}-\frac{1}{2}a^{2}(x_{0})k^{2}\right]\\ \underset{f(\Omega )\;see\;eqs.(290),(292),(293a),(311)\&(315b)}{\underset{︸}{\times \left[\prod_{m=1}^{\infty }\int_{-\infty }^{+\infty }\int_{-\infty }^{+\infty }\frac{d\text{Re}x_{m}d\text{Im}x_{m}}{\pi /\left(m\beta \omega_{m}^{2}\right)}\right]\text{exp}\left\{-m\beta \sum_{m=1}^{\infty }[\omega_{m}^{2}+\Omega^{2}(x_{0})]\left[\begin{array}{l} {\left(\text{Re}x_{m}-ik\frac{1/(m\beta )}{\omega_{m}^{2}+\Omega^{2}(x_{0})}\text{cos}\omega_{m}\tau \right)}^{2}\\ +{\left(\text{Im}x_{m}-ik\frac{1/(m\beta )}{\omega_{m}^{2}+\Omega^{2}(x_{0})}\text{sin}\omega_{m}\tau \right)}^{2} \end{array}\right]\right\}}}\\ =Z_{FK}^{-1}\int_{-\infty }^{+\infty }\frac{dx_{0}\text{exp}[-\beta L_{FK}(x_{0})]}{\sqrt{2\pi \hbar^{2}\beta /m}}\frac{\hbar \beta \Omega (x_{0})/2}{\text{sinh}(\hbar \beta \Omega (x_{0})/2)}\int_{-\infty }^{+\infty }\frac{dk}{2\pi }V(k)\text{exp}\left[ikx_{0}-\frac{1}{2}a^{2}(x_{0})k^{2}\right] \end{array}$$

where the Fourier *k*- (wave vector) representation was implemented for external potential so that the quantum path to explicitly appear in evaluation; this technique had helped for performing the quadratic completion of paths in the view of harmonic-like integration of type (290) with the result (311); nevertheless, in course of these operations the new quantity was introduced, namely the fluctuation width

(330a) $$a^{2}(x_{0})\;=\;2\frac{1}{m\beta }\sum_{m=1}^{\infty }\frac{1}{\omega_{m}^{2}\;+\;\Omega^{2}(x_{0})}$$

which can be immediately recognized as directly related with generalized Riemann series (304), thus having the form [85]

(330b) $$a^{2}(x_{0})\;=\;\;2\frac{1}{m\beta }R(\Omega )\;=\;\frac{1}{m\beta \Omega^{2}}\left[\frac{\hbar \beta \Omega }{2}\text{coth}\left(\frac{\hbar \beta \Omega }{2}\right)-1\right]$$

Expression (329) can be even more simplified when solving out the *k*-integral by back considering the Fourier transformation for the potential and then proceeding with the quadratic completion toward the Poisson standard integration

(331) $$\begin{array}{c} V_{a^{2}(x_{0})}(x_{0})\;=\;\int_{-\infty }^{+\infty }\frac{dk}{2\pi }V(k)\text{exp}\left[ikx_{0}-\frac{1}{2}a^{2}(x_{0})k^{2}\right]\\ =\int_{-\infty }^{+\infty }\frac{dk}{2\pi }\int_{-\infty }^{+\infty }dxV(x)\text{exp}\left(-ikx\right)\text{exp}\left[ikx_{0}\;-\frac{1}{2}a^{2}(x_{0})k^{2}\right]\\ =\;\frac{1}{2\pi }\;\int_{-\infty }^{+\infty }dxV(x)\text{exp}\left[-\frac{{\left(x\;-\;x_{0}\right)}^{2}}{2a^{2}(x_{0})}\right]\int_{-\infty }^{+\infty }dk\text{exp}\left[{\left(\frac{x\;-\;x_{0}}{\sqrt{2}a(x_{0})}\;-\;i\;\frac{a(x_{0})}{\sqrt{2}}k\right)}^{2}\right]\\ =\;\frac{1}{2\pi }\int_{-\infty }^{+\infty }dxV(x)\text{exp}\left[-\frac{{\left(x\;-\;x_{0}\right)}^{2}}{2a^{2}(x_{0})}\right]\int_{-\infty }^{+\infty }dk^{\prime }\text{exp}\left[-\frac{a^{2}(x_{0})}{2}{k^{\prime }}^{2}\right]\\ =\;\frac{1}{\sqrt{2\pi a^{2}(x_{0})}}\;\int_{-\infty }^{+\infty }dxV(x)\text{exp}\left[-\frac{{\left(x\;-\;x_{0}\right)}^{2}}{2a^{2}(x_{0})}\right]\\ \equiv \;{〈V(x)〉}_{a^{2}(x_{0})} \end{array}$$

The potential (331) is known as the smeared out potential and has a major role in explaining the quantum stabilization of matter, as will be largely discussed in the next section. For the moment it is regarded jus as the integral transformation of the original applied potential by convolution with a Gaussian packet with the width *a*^2^(*x*_0_) that accounts for the existing quantum fluctuation in the system.

Nevertheless, Equation (331) leaves the Feynman-Kleinert average of external potential with the result [85]

(332) $${〈V(x(\tau ))〉}_{FK}\;=\;Z_{FK}^{-1}\;\int_{-\infty }^{+\infty }\frac{dx_{0}}{\sqrt{2\pi \hbar^{2}\beta /m}}{〈V(x)〉}_{a^{2}(x_{0})}\frac{\hbar \beta \Omega (x_{0})/2}{\text{sinh}(\hbar \beta \Omega (x_{0})/2)}\text{exp}[-\beta L_{FK}(x_{0})]$$

For the rest of the averaged terms in (328) the evaluations are considerably easier since for each of them we have only to compute their smeared out version (331), with the yield for the trial harmonic

(333) $$\begin{array}{c} {\langle \frac{m}{2}\Omega^{2}(x_{0}){\left(x\;-\;x_{0}\right)}^{2}\rangle }_{a^{2}(x_{0})}\\ =\;\frac{m}{2}\Omega^{2}(x_{0})\frac{1}{\sqrt{2\pi a^{2}(x_{0})}}\int_{-\infty }^{+\infty }dx{\left(x\;-\;x_{0}\right)}^{2}\;\text{exp}\left[-\;\frac{{\left(x\;-\;x_{0}\right)}^{2}}{2a^{2}(x_{0})}\right]\;=\;\frac{m}{2}\Omega^{2}(x_{0})a^{2}(x_{0}) \end{array}$$

and respectively for the Feynman-Kleinert trial function

(334) $${〈L_{FK}(x_{0})〉}_{a^{2}(x_{0})}\;=\;L_{FK}(x_{0})\frac{1}{\sqrt{2\pi a^{2}(x_{0})}}\;\int_{-\infty }^{+\infty }dx\text{exp}\left[-\;\frac{{\left(x\;-\;x_{0}\right)}^{2}}{2a^{2}(x_{0})}\right]\;=\;L_{FK}(x_{0})$$

When replaced in average form (332) the forms (333) and (334) cumulate with the smeared out potential (331) in the final Feynman-Kleinert average equation (328), now featuring the form

(335) $$0\;=\;\int_{-\infty }^{+\infty }\frac{dx_{0}}{\sqrt{2\pi \hbar^{2}\;\beta /m}}\;\left\{V_{a^{2}(x_{0})}(x_{0})-\frac{m}{2}\Omega^{2}(x_{0})a^{2}(x_{0})\;-\;L_{FK}(x_{0})\right\}\frac{\hbar \beta \Omega (x_{0})/2}{\text{sinh}(\hbar \beta \Omega (x_{0})/2)}\text{exp}[-\beta L_{FK}(x_{0})]$$

from where the first stage of variational algorithm is fulfilled by the obvious choice

(336) $$L_{FK}(x_{0})\;=\;V_{a^{2}(x_{0})}(x_{0})\;-\;\frac{m}{2}\Omega^{2}(x_{0})a^{2}(x_{0})$$

With Equation (336) the Feynman-Kleinert potential (323) now displays as [85]

(337) $$W_{FK}(x_{0})\;=\;\frac{1}{\beta }\text{ln}\left[\frac{\text{sinh}(\hbar \beta \Omega (x_{0})/2)}{\hbar \beta \Omega (x_{0})/2}\right]\;+\;V_{a^{2}(x_{0})}(x_{0})\;-\;\frac{m}{2}\Omega^{2}(x_{0})a^{2}(x_{0})$$

There remains only to finally optimize the explicit potential (337) for the harmonic (trial) frequency assuring therefore the equilibrium of the gained lowest approximation of the ground state for the concerned system. This is simply achieved through the chain derivative

(338) $$0\;=\;\frac{dW_{FK}(x_{0})}{d\Omega^{2}(x_{0})}\;=\;\frac{\partial W_{FK}(x_{0})}{\partial \Omega^{2}(x_{0})}\;+\;\frac{\partial W_{FK}(x_{0})}{\partial a^{2}(x_{0})}\frac{\partial a^{2}(x_{0})}{\partial \Omega^{2}(x_{0})}$$

seeing that also the fluctuation width (330) depends on harmonic frequency. Moreover, due to the derivative equivalence 2(∂/ ∂Ω^2^)Ξ = (1/ Ω)(∂/ ∂Ω)Ξ the first term in (338) is arranged to emphasize on its vanishing nature when recalling the Equation (337) and the fluctuation width (330b)

(339) $$\frac{\partial W_{FK}(x_{0})}{\partial \Omega^{2}(x_{0})}\;=\;\frac{m}{2}\;\left\{\frac{1}{m\beta \Omega^{2}}\left[\frac{\hbar \beta \Omega }{2}\text{coth}\left(\frac{\hbar \beta \Omega }{2}\right)\;-\;1\right]\;-\;a^{2}(x_{0})\right\}\;=\;0$$

This way, from Equation (338) it remains only the simple condition

(340) $$\frac{\partial W_{FK}(x_{0})}{\partial a^{2}(x_{0})}\;=\;0$$

that provides for (337) the optimum (stabilization) frequency of quantum fluctuation

(341) $$\Omega^{2}(x_{0})\;=\;\frac{2}{m}\;\frac{\partial V_{a^{2}(x_{0})}(x_{0})}{\partial a^{2}(x_{0})}$$

Nevertheless, through observing the huge role both the smeared out potential (331) and the fluctuation width (330) play in deriving the approximated equilibrium ground state they deserve be further analyzed and commented in relation with matter stability.

#### 5.3.3. Quantum Smeared Effects and the Stability of Matter

The intriguing role the smeared potential in special and the smearing effect in general play in optimization of the total energy and partition function of a quantum system opens the possibility analyzing the “smearing” phenomenon of the quantum fluctuation in a more fundamental way.

**I.** Firstly, it was noted that the smearing potential (331) appears as a Gaussian convolution of the applied potential, although modeling the evolution of a wave-packet under that potential influence; in other terms, it appears the fundamental question whether the Gaussian and wave function “kernels” behave in similar way throughout the smearing effect of quantum fluctuations; analytically, one likes to see whether the next smearing average equality readily holds

(342) $${\langle \text{exp}(-ikx)\rangle }_{a^{2}(x_{0})}\overset{?}{=}{\langle \text{exp}(-k^{2}x^{2})\rangle }_{a^{2}(x_{0})}$$

In order to check (342) one separately computes each of its sides by the aid of *k*-form of (331) and successively gets the smearing average for the wave-function

(343a) $$\begin{array}{c} {\langle \text{exp}(-ikm)\rangle }_{a^{2}(x_{0})}\;=\;\int_{-\infty }^{+\infty }\frac{dk}{2\pi }\text{exp}\left[-ikx\;+\;ikx_{0}\;-\;\frac{1}{2}a^{2}(x_{0})k^{2}\right]\\ =\;\int_{-\infty }^{+\infty }\frac{dk}{2\pi }\text{exp}\left[-ik(x\;-\;x_{0})\;-\;\frac{1}{2}a^{2}(x_{0})k^{2}\right]\\ =\;\text{exp}\left[-\frac{{\left(x\;-\;x_{0}\right)}^{2}}{2a^{2}(x_{0})}\right]\;\int_{-\infty }^{+\infty }\frac{dk}{2\pi }\text{exp}\left\{-\frac{a^{2}(x_{0})}{2}{\left[k\;+\;i\frac{x\;-\;x_{0}}{a^{2}(x_{0})}\right]}^{2}\right\}\\ =\;\frac{1}{2\pi }\;\text{exp}\left[-\frac{{\left(x\;-\;x_{0}\right)}^{2}}{2a^{2}(x_{0})}\right]\;\int_{-\infty }^{+\infty }dk^{\prime }\text{exp}\left\{-\frac{a^{2}(x_{0})}{2}{k^{\prime }}^{2}\right\}\\ =\;\frac{1}{\sqrt{2\pi a^{2}(x_{0})}}\text{exp}\left[-\frac{{\left(x\;-\;x_{0}\right)}^{2}}{2a^{2}(x_{0})}\right] \end{array}$$

and for the Gaussian packet

(343b) $$\begin{array}{c} {\langle \text{exp}\left(-k^{2}x^{2}\right)\rangle }_{a^{2}(x_{0})}\;=\;\int_{-\infty }^{+\infty }\frac{dk}{2\pi }\text{exp}\left[-k^{2}x^{2}\;+\;ikx_{0}\;-\;\frac{1}{2}a^{2}(x_{0})k^{2}\right]\\ =\int_{-\infty }^{+\infty }\frac{dk}{2\pi }\text{exp}\left[-k^{2}\;\left(x^{2}\;+\;\frac{a^{2}(x_{0})}{2}\right)\;+\;ikx_{0}\right]\\ =\text{exp}\left[-\frac{x_{0}^{2}}{4\left(x^{2}\;+\;a^{2}(x_{0})/2\right)}\right]\;\int_{-\infty }^{+\infty }\frac{dk}{2\pi }\text{exp}\left\{-\left(x^{2}\;+\;a^{2}(x_{0})/2\right){\left[k\;-\;i\frac{x_{0}}{2\left(x^{2}\;+\;a^{2}(x_{0})/2\right)}\right]}^{2}\right\}\\ =\frac{1}{2\pi }\text{exp}\left[-\frac{x_{0}^{2}}{4\left(x^{2}\;+\;a^{2}(x_{0})/2\right)}\right]\;\int_{-\infty }^{+\infty }dk^{\prime }\text{exp}\left\{-\left(x^{2}\;+\;a^{2}(x_{0})/2\right){k^{\prime }}^{2}\right\}\\ =\;\frac{1}{\sqrt{2\pi \left[2x^{2}\;+\;a^{2}(x_{0})\right]}}\text{exp}\left[-\frac{x_{0}^{2}}{2\left(2x^{2}\;+\;a^{2}(x_{0})\right)}\right] \end{array}$$

Now, for closely comparison of the expressions (343a) and (343b) the most elegant way is to make once more recourse to the smearing procedure, this time referring both to the entire paths and Feynman centroid; to this end, the previous result (333) is here used in the variant:

(344a) $${\langle {\left(x\;-\;x_{0}\right)}^{2}\rangle }_{a^{2}}\;=\;a^{2}$$

It allows the additional similar relationships

(344b) $${〈x^{2}〉}_{a^{2}}\;=\;{〈x_{0}^{2}〉}_{a^{2}}\;=\frac{a^{2}}{2}$$

Note that the equality (344b) is due to the symmetry of the smearing average formula (331) at the interchange *x* ↔ *x*_0_, while the mixed term of (344a) expansion vanishes, 〈*xx*_0_〈 _*a*^2^(*x*_0_)_ = 0, in any path representation. With these rules, one can reconsider Equations (343a) and (243b) by performing the formal equivalences

(345a) $${\left(x\;-\;x_{0}\right)}^{2}\;\approx \;a^{2}$$

(345b) $$x^{2}\;\approx \;\frac{a^{2}}{2}$$

(345c) $$x_{0}^{2}\;\approx \;\frac{a^{2}}{2}$$

yielding with

(346a) $${\langle \text{exp}(-ikx)\rangle }_{a^{2}}\;\approx \;\frac{1}{\sqrt{2\pi a^{2}}}\text{exp}\left[-\frac{1}{2}\right]$$

(346b) $${\langle \text{exp}\left(-k^{2}x^{2}\right)\rangle }_{a^{2}}\;\approx \;\frac{1}{\sqrt{4\pi a^{2}}}\text{exp}\left[-\frac{1}{8}\right]\;=\;{\langle \text{exp}(-ikx)\rangle }_{a^{2}}\;\frac{\text{exp}(3/8)}{\sqrt{2}}$$

Since the difference between these expressions is numerically proportionally with the factor

(347) $$\frac{\text{exp}(3/8)}{\sqrt{2}}\;≅\;1.029$$

they can be considered as identical in quantum smearing effects and Equation (342) as valid.

Yet, the quantum identity between the plane-wave and Gaussian packet has profound quantum implication, while revealing for instance the de Broglie – Born identity in Gaussian normalization of the de Broglie moving wave-packet. It may express as well the observational Gaussian character of the wave-function evolution in Hilbert space. Finally, and very important, it leads with *explanation* of the Bohr first postulate, *i.e.*, it is able to explain the stationary wave on orbits under singular (Coulombic) potential thus explaining the matter stabilization on rigorous quantum base, rather than to admit it by the power of a postulate. This is to be proved next [39,85].

**II.** Let’s consider a quantum system evolving under the influence of the Yukawa potential, as a generalization of the Coulomb interaction, available also for the sub-nuclear world

(348) $$V_{Yuk}(r)\;=\;\frac{A}{r}\text{exp}(-\alpha r)$$

with *r* = *x* − *x*_0_, which goes to the celebrated Hydrogen Coulomb central potential in the limit

(349) $$\underset{\begin{array}{l} a\to 0\\ A=-e_{0}^{2} \end{array}}{\text{lim}}\;V_{Yuk}(r)\;=\;V_{H}(r)\;=\;-\frac{e_{0}^{2}}{r}$$

with $e_{0}^{2}=-e^{2}/4\pi {ɛ}_{0}$. Now, we like to investigate the smeared version of the Yukawa potential (348). In 3D towards radial framework the general definition (331) specializes for (348) with the form

(350a) $$\begin{array}{c} {\langle V_{Yuk}(r\;=\;x\;-\;x_{0})\rangle }_{a^{2}}\;=\;A4\pi \;\int_{0}^{+\infty }\frac{r^{2}dr}{{\left(\sqrt{2\pi a^{2}}\right)}^{3}}\;\frac{e^{-ar}}{r}\text{exp}\left[-\frac{{\left(x\;-\;x_{0}\right)}^{2}}{2a^{2}}\right]\\ =\;A2\pi e^{\alpha x_{0}}\;\int_{0}^{+\infty }\frac{d(r^{2})}{{\left(\sqrt{2\pi a^{2}}\right)}^{3}}e^{-\alpha x}\text{exp}\left[-\frac{r^{2}}{2a^{2}}\right] \end{array}$$

In the last expression one can recognize that the squared integration variable is of the same nature as fluctuation width, see Equation (345a) with *r* = *x* − *x*_0_, so that the passage to integration upon the variable *a*^2^ seems natural. This means that the path dependent terms becomes smeared respecting the fluctuations, and the integration (lower) limit changes accordingly

(350b) $${\langle V_{Yuk}(r_{0})\rangle }_{a^{2}}\;=\;A2\pi {\langle e^{\alpha x_{0}}\rangle }_{a^{2}}\;\int_{a^{2}}^{+\infty }\frac{d\left({\tilde{a}}^{2}\right)}{{\left(\sqrt{2\pi {\tilde{a}}^{2}}\right)}^{3}}{\langle e^{-\alpha x}\rangle }_{{\tilde{a}}^{2}}e^{-\frac{r_{0}^{2}}{2{\tilde{a}}^{2}}}$$

In this new integral form only one smeared term is truly of the compulsory form (343a), namely

(351a) $${\langle e^{ax_{0}}\rangle }_{a^{2}}\;=\;{\langle \text{exp}\left[-i(i\alpha )x_{0}\right]\rangle }_{a^{2}}\;≅\;{\langle \text{exp}\left[-{\left(i\alpha \right)}^{2}x_{0}^{2}\right]\rangle }_{a^{2}}$$

where also the proofed identity (342) was considered upon it. Yet, the form (351a) may be further approximated by the application of the Jensen-Peierls equality limit of the Equation (324) to yield

(351b) $${\langle e^{\alpha x_{0}}\rangle }_{a^{2}}\;≅\;{\langle \text{exp}\left[\alpha^{2}x_{0}^{2}\right]\rangle }_{a^{2}}\;\approx \;\text{exp}\left\lfloor \alpha^{2}{\langle x_{0}^{2}\rangle }_{a^{2}}\right\rfloor \;=\;\text{exp}\left(\alpha^{2}a^{2}/2\right)$$

when the smeared rules (344b) was counted as well. The other similar term in (350b) is however evaluated by the approximated inverse identity

(352a) $${\langle e^{-\alpha x}\rangle }_{{\tilde{a}}^{2}}\;≅\;\frac{1}{{\langle e^{\alpha x}\rangle }_{{\tilde{a}}^{2}}}\;≅\;\text{exp}\left(-\alpha^{2}{\tilde{a}}^{2}/2\right)$$

however, based on the unconnected version of the second order Wick cumulant in (219c)

(352b) $${\langle e^{\alpha x}\rangle }_{{\tilde{a}}^{2}}{\langle e^{-\alpha x}\rangle }_{{\tilde{a}}^{2}}\;≅\;{\langle e^{\alpha x}e^{-\alpha x}\rangle }_{{\tilde{a}}^{2}}\;=\;{\langle 1\rangle }_{{\tilde{a}}^{2}}\;=\;1$$

The expressions (351b) and (352a) produce for the smeared Yukawa potential (350b) the form

(353) $${\langle V_{Yuk}\left(r_{0}\right)\rangle }_{a^{2}}\;=\;A2\pi e^{\frac{\alpha^{2}a^{2}}{2}}\;\int_{-\infty }^{+\infty }\frac{d\left({\tilde{a}}^{2}\right)}{{\left(\sqrt{2\pi {\tilde{a}}^{2}}\right)}^{3}}\;e^{-\frac{\alpha^{2}{\tilde{a}}^{2}}{2}-\frac{r_{0}^{2}}{2{\tilde{a}}^{2}}}$$

Now, through considering the variable changing

(354a) $$\zeta \;=\;\frac{r_{0}}{\sqrt{2{\tilde{a}}^{2}}}$$

the resulted transformations

(354b) $${\tilde{a}}^{2}\;=\;\frac{r_{0}^{2}}{2\zeta^{2}};\;\frac{d\left({\tilde{a}}^{2}\right)}{{\left(\sqrt{{\tilde{a}}^{2}}\right)}^{3}}\;=\;-\frac{2\sqrt{2}}{r_{0}}d\zeta$$

are combined so that the smeared potential (353) finally casts as

(355a) $${\langle V_{Yuk}\left(r_{0}\right)\rangle }_{a^{2}}\;=\;A\;\frac{\text{exp}\left(\alpha^{2}a^{2}/2\right)}{r_{0}}\;\frac{2}{\sqrt{\pi }}\;\int_{0}^{r_{0}/\sqrt{2a^{2}}}d\zeta \;\text{exp}\left[-\left(\zeta^{2}\;+\;\frac{\alpha^{2}r_{0}^{2}}{4\zeta^{2}}\right)\right]$$

Expression (355a) has no longer singularity at origin, since the integral is behaving like its integration interval for the limit *r*_0_ → 0, which gives

(355b) $${\langle V_{Yuk}(0)\rangle }_{a^{2}}\;=\;A\frac{2\;\text{exp}\left(\alpha^{2}a^{2}/2\right)}{\sqrt{2\pi a^{2}}}$$

Now, there is clear that under the Coulombic limit (349) the resulting atomic (say for Hydrogen case) smeared effect is expressed by the form

(356a) $${\langle V_{H}\left(r_{0}\right)\rangle }_{a^{2}}\;=\;-\frac{e_{0}^{2}}{r_{0}}\;\frac{2}{\sqrt{\pi }}\;\int_{0}^{r_{0}/\sqrt{2a^{2}}}d\zeta \text{exp}\left(-\zeta^{2}\right)\;=\;-\frac{e_{0}^{2}}{r_{0}}\text{erf}\left(r_{0}/\sqrt{2a^{2}}\right)$$

while its value on origin is of the finite value

(356b) $${\langle V_{H}(0)\rangle }_{a^{2}}\;=\;-\frac{2e_{0}^{2}}{\sqrt{2\pi a^{2}}}$$

thus assuring (and explaining) why the atomic electron(s) do not fall onto the nucleus.

Therefore, the smearing procedure plays a kind of renormalization role in transforming singular potential in finite interactions by means of quantum fluctuation effects. Such picture strongly advocates for the powerful path integral formalism in general and of that of Feynman-Kleinert in special for explicitly accounting of the fluctuation width in optimizing the quantum equilibrium states. Nevertheless, worth particularizing the Feynman-Kleinert formalism to the ground and excited states for better capture its reliability and limits.

#### 5.3.4. Ground State (β→∞, T→0K) Case

The basic ground state conditions in terms of thermodynamic factor (*β*) or the temperature (*T*):

(357) β → ∞ ⇔ T → 0

aims to bring the Feynman-Kleinert formalism, through its working potential (337), closer to the ground state as usually provided by the consecrated quantum variational principle; for this purpose it will be firstly specialized within the general limit (357) and then tested for the paradigmatic Hydrogen ground state case for investigating upon the accuracy of the formalism itself.

As such, the first component of the Feynman-Kleinert potential (337) has the ground state limit

(358a) $$\begin{array}{c} \underset{\beta \to \infty }{\text{lim}}\left\{\frac{1}{\beta }\text{ln}\left[\frac{\text{sinh}(\hbar \beta \Omega (x_{0})/2)}{\hbar \beta \Omega (x_{0})/2}\right]\right\}\\ =\;\frac{\hbar \Omega }{2}\;\underset{\beta \to \infty }{\text{lim}}\frac{\text{cosh}(\hbar \beta \Omega (x_{0})/2)}{\text{sinh}(\hbar \beta \Omega (x_{0})/2)}\;-\;\underset{\beta \to \infty }{\text{lim}}\frac{1}{\beta }\\ =\;\frac{\hbar \Omega }{2} \end{array}$$

recovering the ground state of harmonic motion for the trial fluctuations, while the ground state of the fluctuation width (330b) reads as

(358b) $$\begin{array}{c} \underset{\beta \to \infty }{\text{lim}}a^{2}(x_{0})\;=\;\underset{\beta \to \infty }{\text{lim}}\left\{\frac{1}{m\beta \Omega^{2}}\left[\frac{\hbar \beta \Omega }{2}\text{coth}\left(\frac{\hbar \beta \Omega }{2}\right)-1\right]\right\}\\ =\;\frac{\hbar }{2m\Omega }\underset{\beta \to \infty }{\text{lim}}\frac{\text{cosh}(\hbar \beta \Omega (x_{0})/2)}{\text{sinh}(\hbar \beta \Omega (x_{0})/2)}\;-\;\underset{\beta \to \infty }{\text{lim}}\frac{1}{m\beta \Omega^{2}}\\ =\;\frac{\hbar }{2m\Omega } \end{array}$$

from where also the asymptotic trial fluctuations’ frequency springs as

(358c) $$\underset{\beta \to \infty }{\text{lim}}\Omega \;=\;\frac{\hbar }{2ma^{2}(x_{0})}$$

Next, by combining relations (358) with the working general effective-classical approximation potential (337) the general ground state limit looks like

(359) $$\begin{array}{c} W_{FK}^{T\to 0}(x_{0})\;=\;\underset{\beta \to \infty }{\text{lim}}\left\{\frac{1}{\beta }\text{ln}\left[\frac{\text{sinh}(\hbar \beta \Omega (x_{0})/2)}{\hbar \beta \Omega (x_{0})/2}\right]\;-\;\frac{m}{2}\;\Omega^{2}(x_{0})a^{2}(x_{0})\right\}\;+\;V_{a^{2}(x_{0})}^{T\to 0}(x_{0})\\ =\;\frac{\hbar \Omega }{4}\;+\;V_{a^{2}(x_{0})}^{T\to 0}(x_{0})\\ =\;\frac{\hbar^{2}}{8ma^{2}}\;+\;V_{a^{2}(x_{0})}^{T\to 0}(x_{0}) \end{array}$$

The ground state smeared out potential remains to be individuated in solving the associated ground state problem.

Very interestingly, the expression (359) entirely corresponds to the smeared out effect applied on the ordinary quantum Hamiltonian

(360) $$\hat{H}\;=\;-\frac{\hbar^{2}}{2m}\partial_{x}^{2}\;+\;V(x),$$

as one can immediately check out through applying the general smearing averaging definition (331) on it

(361) $$\begin{array}{c} {\langle \hat{H}\rangle }_{a^{2}(x_{0})}\;=\;\frac{1}{\sqrt{2\pi a^{2}(x_{0})}}\;\int_{-\infty }^{+\infty }dx\left[-\frac{\hbar^{2}}{2m}\partial_{x}^{2}\;+\;V(x)\right]\text{exp}\left[-\frac{{\left(x\;-\;x_{0}\right)}^{2}}{2a^{2}(x_{0})}\right]\\ =\;-\frac{\hbar^{2}}{2m}\frac{1}{\sqrt{2\pi a^{2}(x_{0})}}\;\int_{-\infty }^{+\infty }dx\left\{\frac{\partial^{2}}{\partial x^{2}}\text{exp}\left[-\frac{{\left(x\;-\;x_{0}\right)}^{2}}{2a^{2}(x_{0})}\right]\right\}\;+\;V_{a^{2}(x_{0})}^{T\to 0}(x_{0})\\ =\;-\frac{\hbar^{2}}{2m}\;\frac{1}{\sqrt{2\pi a^{2}(x_{0})}}\;\int_{-\infty }^{+\infty }dx\left\{\left[\frac{{\left(x\;-\;x_{0}\right)}^{2}}{2a^{2}(x_{0})}\;-\;1\right]\text{exp}\left[-\frac{{\left(x\;-\;x_{0}\right)}^{2}}{2a^{2}(x_{0})}\right]\right\}\;+\;V_{a^{2}(x_{0})}^{T\to 0}(x_{0})\\ =\;\frac{\hbar^{2}}{8ma^{2}}\;+\;V_{a^{2}(x_{0})}^{T\to 0}(x_{0}) \end{array}$$

The identity between expressions (359) and (361) leaves with the important confirmation that the smearing operation produces in fact the average of quantum fluctuation for the ground state equilibrium. For the Coulomb interaction, say for the Hydrogen atom, either of above expressions produces the working form

(362) $$W_{FK-H}^{T\to 0}(x_{0})\;=\;{\langle {\hat{H}}_{H}\rangle }_{a^{2}(x_{0})}\;=\;\frac{3\hbar^{2}}{8ma^{2}}\;-\;\frac{2e_{0}^{2}}{\sqrt{2\pi a^{2}}}$$

where the 3D version of the kinetic term of (361) was here considered aside the smearing out potential in the origin (356b); they turn out the ready form for ordinary minimization respecting the fluctuation width

(363) $$\frac{\partial }{\partial a^{2}(x_{0})}{\langle {\hat{H}}_{H}\rangle }_{a^{2}(x_{0})}\;=\;0$$

The solution of the Equation (363) represents the optimum width for the quantum fluctuations in Hydrogen atom

(364a) $$a_{FK}^{opt}\;=\;\frac{3\hbar^{2}\sqrt{2\pi }}{8me_{0}^{2}}$$

which, in terms of the standard first Bohr radius

(364b) $$a_{0}\;=\;\frac{\hbar^{2}}{me_{0}^{2}}$$

reads as

(364c) $$a_{FK}^{opt}\;=\;\sqrt{\frac{9\pi }{32}}a_{0}\;≅\;0.94a_{0}$$

thus producing only a 6% error in predicting the radial localization in stabilizing the electronic ground state orbit, *i.e.*, being closer to the nucleus respecting the exact Bohr-Schrödinger solution. However, for predicted approximated ground state energy the error is a bit higher due to the specific dependency

(365) $$E_{FK-H}^{\text{min}}\;=\;{\langle {\hat{H}}_{H}\rangle }_{a^{2}(x_{0})}\left(a_{FK}^{opt}\right)\;=\;-\frac{e_{0}^{2}}{\sqrt{2\pi }a_{FK}^{opt}}\;=\;-\;\frac{8}{3\pi }\left(\frac{e_{0}^{2}}{2a_{0}}\right)\;=\;\frac{8}{3\pi }E_{0}^{H}\;≅\;0.84E_{0}^{H}$$

predicting the spectral localization with an error about 16% higher than the exact ground state of Hydrogen atom.

Nevertheless, besides the approximated character of the formalism, the Feynman-Kleinert approach adapts very well to the singular potential, having the advantage of being compatible with a wide class of electronic potentials in atoms and molecules; moreover, it can be particularized to the ground state in the same degree it accounts for higher temperature cases, the other extreme of thermodynamic limit – see the next section, spanning this way in principle the entire range of statistical systems at equilibrium; note that such “universal” quantum statistical picture of equilibrium is hard to found in the quantum theory, at the same level of elegance, analyticity and complex ideas [93–97].

#### 5.3.5. Excited State (β→0, T→∞) Case. Wigner Expansion

As before, the components of the Feynman-Kleinert potential (337) are to be now evaluated in the excited states or the *valence* limit

(366) β → 0 ⇔ T → ∞

This limit is applied on terms of Feynman-Kleinert potential reduced to the hyperbolic functions on which the approximations of type (306) may be applied.

With this recipe we firstly evaluate

(367) $${\left[\frac{\hbar \beta \Omega }{2}\text{coth}\left(\frac{\hbar \beta \Omega }{2}\right)\;-\;1\right]}_{\beta \to 0}\;≅\;\frac{\hbar^{2}\beta^{2}\Omega^{2}}{12}$$

leaving for the fluctuation width the valence approximation

(368) $$a_{\beta \to 0}^{2}(x_{0})\;≅\;\frac{1}{m\beta \Omega^{2}}\frac{\hbar^{2}\beta^{2}\Omega^{2}}{12}\;=\;\frac{\hbar^{2}\beta }{12m}$$

while observing the limitation to the first order in *β* expansion.

The same methodology holds also for the harmonic fluctuation term

(369) $$\Lambda \;=\;\text{ln}\left[\frac{\text{sinh}(\hbar \beta \Omega (x_{0})/2)}{\hbar \beta \Omega (x_{0})/2}\right]$$

in what performing the *β* McLaurin expansion up to the second order truncation

(370) $$\Lambda_{\beta \to 0}\;≅\;\underset{\beta \to 0}{\text{lim}}\Lambda \;+\;\beta \underset{\beta \to 0}{\text{lim}}\left(\frac{\partial \Lambda }{\partial \beta }\right)\;+\;\frac{1}{2}\;\beta^{2}\underset{\beta \to 0}{\text{lim}}\left(\frac{\partial^{2}\Lambda }{\partial \beta^{2}}\right)$$

with the components

(371a) $$\underset{\beta \to 0}{\text{lim}}\;\Lambda \;=\;\text{ln}\left[\underset{\beta \to 0}{\text{lim}}\;\frac{\text{sinh}(\hbar \beta \Omega (x_{0})/2)}{\hbar \beta \Omega (x_{0})/2}\right]\;=\;0$$

(371b) $$\underset{\beta \to 0}{\text{lim}}\left(\frac{\partial \Lambda }{\partial \beta }\right)\;=\;\underset{\beta \to 0}{\text{lim}}\left[\frac{\hbar \Omega }{2}\text{coth}\left(\frac{\hbar \beta \Omega }{2}\right)\;-\;\frac{1}{\beta }\right]\;=\;\underset{\beta \to 0}{\text{lim}}\left(\frac{\hbar \beta \Omega }{6}\right)\;=\;0$$

(371c) $$\begin{array}{c} \underset{\beta \to 0}{\text{lim}}\left(\frac{\partial^{2}\Lambda }{\partial \beta^{2}}\right)\;=\;\underset{\beta \to 0}{\text{lim}}\left\{\frac{1}{\beta^{2}}\;-\;\frac{\hbar^{2}\Omega^{2}}{4}{\left[\text{csch}\left(\frac{\hbar \beta \Omega }{2}\right)\right]}^{2}\right\}\\ =\;\underset{\beta \to 0}{\text{lim}}\left\{\frac{1}{\beta^{2}}\;-\;\frac{\hbar^{2}\Omega^{2}}{4}{\left[\frac{2}{\hbar \beta \Omega }\;-\;\frac{\hbar \beta \Omega }{12}\right]}^{2}\right\}\\ =\;\underset{\beta \to 0}{\text{lim}}\left\{\frac{h^{2}\Omega^{2}}{12}\;-\;\frac{\hbar^{2}\beta^{2}\Omega^{2}}{4\;\times \;144}\right\}\;=\;\frac{\hbar^{2}\Omega^{2}}{12} \end{array}$$

where, in the last expression, the hyperbolic cosecant was approximated, in the spirit of Equation (306), with the form

(372) $${\left[\text{csc}\text{h}\left(\Xi \right)\right]}_{\Xi \to 0}\;≅\;\frac{1}{\Xi }\;-\;\frac{\Xi }{6}$$

With the partial limits (371) the expansion (370) becomes

(373) $$\Lambda_{\beta \to 0}\;≅\;\beta^{2}\frac{\hbar^{2}\Omega^{2}}{24}$$

providing on its turn the harmonic term approximation

(374) $${\left\{\frac{1}{\beta }\text{ln}\left[\frac{\text{sinh}(\hbar \beta \Omega (x_{0})/2)}{\hbar \beta \Omega (x_{0})/2}\right]\right\}}_{\beta \to 0}\;≅\;\beta \frac{\hbar^{2}\Omega^{2}}{24}$$

with the same first *β* -order dependency as the limit (368) of the fluctuation width.

The remaining term for evaluation in higher temperature limit is the smeared potential (331); for it the next change of variable will be done by considering

(375) $$z(x)\;=\;\frac{x\;-\;x_{0}}{\sqrt{2a^{2}(x_{0})}},\;dz(x)\;=\;\frac{dx}{\sqrt{2a^{2}(x_{0})}}$$

allowing for the successively series formulation known as the Wigner expansion [98]

(376) $$\begin{array}{c} V_{a^{2}(x_{0})}^{\beta \to 0}(x_{0})\;=\;\frac{1}{\sqrt{2\pi a^{2}(x_{0})}}\;\sqrt{2a^{2}(x_{0})}\;\int_{-\infty }^{+\infty }dzV\left(x_{0}\;+\;\sqrt{2a^{2}(x_{0})}z\right)\text{exp}\left[-z^{2}\right]\\ \overset{\beta \to 0}{≅}\;\frac{1}{\sqrt{\pi }}\;\int_{-\infty }^{+\infty }dz\left\{V(x_{0})+\left[\sqrt{2a^{2}(x_{0})}z\right]V^{\prime }(x_{0})+\frac{1}{2}{\left[\sqrt{2a^{2}(x_{0})}z\right]}^{2}V^{\prime \prime }(x_{0})\right\}\text{exp}\left[-z^{2}\right]\\ =\;\frac{1}{\pi }\;\int_{-\infty }^{+\infty }dz\left\{V(x_{0})+a^{2}(x_{0})z^{2}V^{\prime \prime }(x_{0})\right\}\text{exp}\left[-z^{2}\right]\\ =\;V(x_{0})\;+\;\frac{1}{2}a^{2}(x_{0})V^{\prime \prime }(x_{0}) \end{array}$$

The expression (376) was obtained due to the small fluctuation width at higher temperatures, see the limit (368) and Feynman’s discussion in Section 5.1 when introducing the idea of effective-classical potential and partition function; it features the small perturbation in terms of fluctuation width around the applied potential “centered” on the Feynman centroid, therefore behaving as a sort of semi-classical expansion.

Indeed, by recalling the trial fluctuation frequency optimal definition (341) it specializes in the high temperature limit potential (376) to the working expression

(377) $$\Omega_{\beta \to 0}^{2}(x_{0})\;=\;\frac{2}{m}\frac{\partial V_{a^{2}(x_{0})}^{\beta \to 0}\;(x_{0})}{\partial a^{2}\;(x_{0})}\;=\;\frac{1}{m}V^{\prime \prime }(x_{0})$$

Finally, by plugging the optimum frequency (377) into the limit (374), and along the limits (368) and (376), back in Feynman-Kleinert potential (337) it acquires the high temperature or the valence form

(378) $$W_{FK}^{T\to \infty }(x_{0})\;=\;V(x_{0})\;\;+\;\hbar^{2}\frac{\beta }{24m}V^{\prime \prime }(x_{0})$$

Most remarkably, the last form is nothing than the semiclassical potential appearing in the second order partition function (262b), thus providing the identical Feynman-Kleinert partition function (322)

(379) $$Z_{FK}^{T\to \infty }\;=\;\int_{-\infty }^{+\infty }\frac{dx_{0}}{\sqrt{2\pi \hbar^{2}\beta /m}}\text{exp}\left[-\beta V(x_{0})-\frac{\hbar^{2}\beta^{2}}{24m}V^{\prime \prime }(x_{0})\right]$$

From such identity one may re-affirm the important conceptual achievement according which the Feynman centroid (281b) for periodic path approach corresponds with the end-point coordinate average (198) in semiclassical expansion.

Moreover, there is clear that the higher temperature or the valence limit of the Feynman-Kleinert periodic path integral approach regains the semiclassical expansion of the non periodic paths of a quantum particle; they become nevertheless periodic at higher temperatures due to higher oscillations around the equilibrium, while possessing not sufficient kinetic energy to break the equilibrium by traveling too far away.

We are now fully convinced that Feynman-Kleinert path integral formulation works fine either at low and higher temperature limits, while recovering both the (Hydrogen) ground state and the semiclassical valence expansion with reliable fidelity, respectively. Nevertheless, it remains to stress on further connection with the electronic density and consequently with the density functional theory towards the quantum chemical applications. These aspects will be addressed in the next section.

### 5.4. Path Integral Connection with Density Functional Theory

#### 5.4.1. Feynman-Kleinert Electronic Density. Analogy with Levy’s Search Mechanism

While reviewing the Feynman-Kleinert algorithm, one can say it is based on two-fold optimization steps:

○ The variational approach for effective-classical potential partition function provides the energy approximation for the ground state, leaving with the Feynman-Kleinert potential, see Equations (328) and (337);
○ The optimization of the Feynman-Kleinert potential respecting the trial harmonic frequency to achieving the thermodynamical equilibrium of the ground state, see Equations (338) & (341).

With these, the working form for the canonical electronic density can be written in terms of the Feynman centroid (281b) by the expression

(380a) $$\rho_{FK}^{\left(I\right)}(x_{0})\;=\;Z_{FK}^{-1}\;\frac{1}{\sqrt{2\pi \hbar^{2}\;\beta /m}}\text{exp}[-\beta W_{FK}(x_{0})]$$

satisfying the centroid normalization

(380b) $$\int_{-\infty }^{+\infty }\rho_{FK}^{\left(I\right)}(x_{0})dx_{0}\;=\;1$$

or, more sophisticatedly, in terms of the quantum path (282a)

(381a) $$\rho_{FK}^{\left(II\right)}(x)\;=\;Z_{FK}^{-1}\int_{-\infty }^{+\infty }\frac{dx_{0}}{\sqrt{2\pi a{\left(x_{0}\right)}^{2}}}\left\{\frac{1}{\sqrt{2\pi \hbar^{2}\beta /m}}\text{exp}[-\beta W_{FK}(x_{0})]\right\}\text{exp}\left[-\frac{{\left(x-x_{0}\right)}^{2}}{2a{\left(x_{0}\right)}^{2}}\right]$$

with the respective normalization to unity

(381b) $$\int_{-\infty }^{+\infty }\rho_{FK}^{\left(II\right)}(x)dx\;=\;1$$

Remarkably, the Feynman-Kleinert variational algorithm in path integrals may be viewed as providing the calculation of the effective electronic density by constructing the constraint-searched partition function picture as the Levy constraint-search formalism [99] does in seeking the electronic density from the trial wave function. More clearly, Levy’s recipe prescribes that the ground state energy minimization scheme (within the second *Hohenberg-Kohn* theorem [1]) involves, in fact, two steps: one over all wave functions (Ψ) that give the same density (the inner minimization step) followed by the minimization throughout all density classes (the outer minimization step)

(382) $$\begin{array}{c} E_{0}\;=\;\underset{\rho }{\text{min}}\left[\underset{\Psi \to \rho }{\text{min}}〈\Psi |(T+V_{ee}+V)\Psi |〉\right]\\ =\;\underset{\rho }{\text{min}}\left[\underset{\Psi \to \rho }{\text{min}}〈\Psi |(T+V_{ee})|\Psi 〉\;+\;\int \rho (x)V(x)dx\right]\\ =\;\underset{\rho }{\text{min}}(F_{HK}[\rho ])\;+\;C_{A}[\rho ]\\ =\;\underset{\rho }{\text{min}}(E[\rho ]) \end{array}$$

through recalling the density functional basic functionals (3)–(5).

Such equivalence between the path integral Feynman-Kleinert formalism and the density functional Levy’s one recommends the use of the Feynman-Kleinert density/densities for being implemented in density functionals with chemical relevance, the electronegativity for instance. In this regard, the density functional for Mulliken electronegativity is in next reviewed and exemplified for atomic scales.

#### 5.4.2. Mulliken Density Functional Electronegativity

For an *N*-electronic system placed into an external potential *V* (*x*) the general (first order) equation of the change in electronegativity, *χ* = *χ*[*N*,*V* (*x*)], can be written as [3,7,9,100–102]

(383) −dχ = ηdN + ∫f(x)dV(x)dx

in which the variation of the electronegativity *χ* (or the negative chemical potential in the Parr definition *μ* = − *χ* [103]) for an electronic state correlates with the number of electrons and potential variation through the chemical hardness (*η*) (264), and the Fukui function (*f*), [3,5]

(384) $$f(x)\;=\;-\;{\left(\frac{\delta \chi }{\delta V(x)}\right)}_{N}\;\equiv \;{\left(\frac{\partial \rho (x)}{\partial N}\right)}_{V}$$

being *x* the position vector, a.k.a. the quantum path or its average as the Feynman centroid.

Next, let us re-express the hardness and Fukui function through the relations [3,104]

(385) $$\eta \;=\;\frac{1}{S}$$

(386) $$f(x)\;=\;\frac{s(x)}{S}$$

where *S* and *s*(*x*) represent the global and the local softness defined respectively as

(387) $$S\;=\;-{\left(\frac{\partial N}{\partial \chi }\right)}_{V}$$

(388) $$s(x)\;=\;-{\left(\frac{\partial \rho (x)}{\partial \chi }\right)}_{V}$$

while being connected by the integral relation

(389) $$S\;=\;\int_{-\infty }^{+\infty }s(x)dx$$

in turn relaying on the assumed *N*-normalized density, see Equation (2).

Now, with Equations (385) and (386), we can integrate the equation (383) for the electronegativity to obtain

(390) $$\chi (N)\;=\;-\int_{0}^{N}\frac{1}{S}dN-\frac{1}{S}\int_{-\infty }^{+\infty }s(x)V(x)dx$$

under the initial natural zero electronegativity value as *V*(*x*)→0. The integrals in (390) can be carried out once the local and global softness *s*(*x*) and *S*, respectively, are analytically known. This can be achieved by assuming the *quasi* independent-particle model within density functional theory providing the softness kernel expression [104]

(391) $$s(x,\;x^{\prime })\;=\;\frac{\nabla \rho (x^{\prime })\cdot [-\nabla V(x^{\prime })]}{{\left[\nabla V(x^{\prime })\right]}^{2}}\delta (x-x^{\prime })+\rho (x)\rho (x^{\prime })$$

The softness kernel (391) allows expressing the local softness *s*(*x*) by the direct integration with the effect of transforming the bi-local into the local density behavior

(392) $$s(x)\;=\;\int_{-\infty }^{+\infty }s(x,\;x^{\prime })dx\;=\;\frac{\nabla \rho (x)\cdot [-\nabla V(x)]}{{\left[\nabla V(x)\right]}^{2}}+N\rho (x)$$

where the well-known delta-Dirac integration rule (225b) and the density normalization condition (2) were used. Consequently, the global softness *S* of (389) can be immediately analytically expressed by further integrating of the local softness (392), with the result

(393) $$S\;=\;\int_{-\infty }^{+\infty }\frac{\nabla \rho (x)\cdot [-\nabla V(x)]}{{\left[\nabla V(x)\right]}^{2}}dx\;+\;N^{2}$$

where the condition (2) accounted once more.

By introducing local and global softness expressions (392) and (393) into the Equation (390) the so called *absolute* electronegativity can be analytically solved yielding the formulation

(394) $$\chi (N)\;=\;-\frac{1}{\sqrt{a}}\text{arctan}\left(\frac{N}{\sqrt{a}}\right)-\frac{b}{a+N^{2}}-NC_{A}\frac{1}{a+N^{2}}$$

in terms of the additionally introduced response density functionals

(395a) $$a\;=\;\int_{-\infty }^{+\infty }\frac{\nabla \rho (x)\cdot [-\nabla V(x)]}{{\left[\nabla V(x)\right]}^{2}}dx$$

(395b) $$b\;=\;\int_{-\infty }^{+\infty }\frac{\nabla \rho (x)\cdot [-\nabla V(x)]}{{\left[\nabla V(x)\right]}^{2}}V(x)\;dx$$

However, since electronegativity (394) may be identified with Parr’s differential electronegativity

(396) $$\chi \;=\;-\mu \;=\;-{\left(\frac{\partial E}{\partial N}\right)}_{V}$$

which, at its turn can be related with the correspondent chemical Mulliken one by means of the finite difference successive transformations

(397) $$\begin{array}{c} \chi \;≅\;\frac{-E(N\;+\;1)\;+E(N\;-\;1)}{2}\\ =\;\frac{\left[E(N\;-\;1)\;-\;E(N)]\;+\;[E(N)\;-\;E(N\;+\;1)\right]}{2}\\ =\;\frac{IP\;+\;EA}{2}\;=\;\chi_{M} \end{array}$$

in terms of ionization potential (*IP*) and electronic affinity (*EA*); Yet, the integral generalization of (397) may be revealed by performing the reverse writing [7]

(398) $$\begin{array}{c} \chi_{M}\;=\;\frac{E(N\;-\;1)\;-\;E(N\;+\;1)}{2}\;=\;-\frac{1}{2}\int_{|N-1〉}^{|N+1〉}dE_{|N〉}[N,V(x)]\\ =\;-\frac{1}{2}\int_{|N-1〉}^{|N+1〉}\left[{\left(\frac{\partial E_{|N〉}}{\partial N}\right)}_{V}dN\;+\;\int \rho_{|N〉}(x)dV_{|N〉}(x)dx\right]\\ =\frac{1}{2}\int_{N-1}^{N+1}\chi (N)dN\;-\;\frac{1}{2}\left[\int \rho_{|N〉}(x)V_{|N+1〉}(x)dx-\int \rho_{|N〉}(x)V_{|N-1〉}(x)dx\right] \end{array}$$

Now, according with the Hohenberg-Kohn first theorem [1] and of the chemical action principle [7,9,18,20] the two terms in the right hand side bracket of equation (398) identically vanish since does not optimize the associations of the electronic density of one state with the external potential applied on that state, thus leaving with the identity [102]

(399) $$\chi_{M}(N)\;=\;\frac{1}{2}\int_{N-1}^{N+1}\chi \left(\tilde{N}\right)d\tilde{N}$$

Upon the insertion of the *absolute* electronegativity (394) in (399) it provides the *chemical* Mulliken density functional [7,100,101]

(400) $$\chi_{M}(N)\;=\;\frac{b\;+\;N\;-\;1}{2\sqrt{a}}\text{arctan}\left(\frac{N\;-\;1}{\sqrt{a}}\right)\;-\;\frac{b\;+\;N\;+\;1}{2\sqrt{a}}\text{arctan}\left(\frac{N\;+\;1}{\sqrt{a}}\right)\;+\;\frac{C_{A}\;-\;1}{4}\text{ln}\left[\frac{a\;+\;{\left(N\;-\;1\right)}^{2}}{a\;+\;{\left(N\;+\;1\right)}^{2}}\right]$$

The exposed density functional formulation of electronegativity features reach physical contents, since the derivation appeals on fundamental quantum principles as the Hohenberg-Kohn and the chemical action theorems, while complementing somehow at the valence (*β →* 0) level the previously density matrix one (263) – worked out in the context of (the fourth order) semiclassical expansion (271); nevertheless, this valence character of the chemical Mulliken electronegativity will be in the following tested for atomic scale through Feynman-Kleinert density implementation.

As a note, it should be mentioned that Yang has shown [105,106] how the integral formulation of the Kohn-Sham density functional theory arrives to the electronic density expression performing the Wigner semiclassical expansion combined with the short time approximation, *i.e.*, the valence approximation, regarding the *β* parameter. For these reasons the present density functional application the Wigner or semiclassical limit of Feynman-Kleinert path integral approach will be used.

#### 5.4.3. Atomic Electronegativity by Feynman Centroid Path Integral

The electrons of the atomic system are distinguished as the core- and the valence- ones within pseudopotential theory of atoms and molecules [100,101,107,108]; this, because it aims to provide a “valence-only” theory for these systems, while assuring the simplification of the computations. Certainly, the all-electrons picture is also possible through facing with the serious technical problem to assure the *orthogonality* constrains among all wave functions of all electrons of an atom. Moreover, having the valence shell treated separately is relevant for computing electronegativity, because of its definition regarding the added electron to the valence shell under the core influence. Therefore, a wise step is provided by the transformation of a many-valence electronic problem into an one-valence electronic system, so that the canonical density formulations can be at once considered. For achieving this, the link between the exact and density dependent pseudopotential is enforced by the latter’s satisfying the *virial theorem* releasing with the radial scaling of the pseudo-orbital [108]

(401a) $$\rho_{PO}^{1/2}(q,\;r)\;=\;\psi (q,\;r)\;=\;q^{3/2}\psi (qr)$$

with the scaling factor *q*. Therefore, the scaling factor *q* is searched in relation with the number of valence electrons, but such to fulfill the normalization condition

(401b) $$\int |\psi (q,r){|}^{2}\;dr\;=\;1$$

Next, the effective potential of the core is represented as a pseudo-potential employing the Stuttgart/Bonn wave function expansion [109]

(402) $$\psi (r)\;=\;\sum_{i}{\text{A}}_{i}\text{exp}\left(-\alpha_{i}r^{2}\right)$$

while the Mulliken electronegativity is computed starting from lithium [109], to assure the existence of the core electrons. For H and He systems the corresponding electronegativity values can be added from other methods of computation [110, 111].

Within the pseudopotential methods we arrive at two possibilities for the electronic density and, consequently, for the electronegativity evaluations.

○ The first one considers only the pseudo-potentials into the path integral formalism that gives the electronic density in the quantum statistical manner as it was described in the previous Section 5.4.1. This way, a strong physical meaning is assured because all the information about the electronic density and electronegativity are comprised (and dictated) only by the pseudopotential. Yet, the problem that arises in this approach is that the electronic density depends on the *β* parameter. This parameter will be fixed so that the electronic density to fulfill the *path integral* normalization condition. Additionally, the search of the *β* parameter must be done in the semiclassical (high temperature) limit (*β →* 0) for which the path integral formalism corresponds to the excited (valence) states of atoms.
○ The second approach takes beyond to the pseudopotential data also the valence basis and the electronic densities are then computed in the accustomed quantum manner. At this point we need to consider the working orbital type for the atomic systems and we will chose the s-basis set because its spherical symmetry.

Accordingly, it follows that both electronic density approaches have their own parametric dependency. This implies that also the computed electronegativity will feature the scaling effect on the electronic density raised due to the one effective valence electronic approach. With this assumption at the background of density computation we should recover in the provided electronegativity the real (many) electronic valence state by an adequate nomination of the specific values for the *β* and *q* parameters.

At this point we need a criterion in order to properly control the re-scaling procedure. In order to unveil this criterion we are looking back on differential electronegativity formula (394) that should be seen as the kernel function for the Mulliken electronegativity functional (400). If we observe the analytical places the introduced chemical response indices *a*, *b* and the chemical action index *C_A_* appear, respectively, it can be easily seen that only the chemical action is coupled with the total number of electrons in the concerned state.

In this respect, while noting the above scaling condition (401a) as being quite restrictive for the scaling factor *q*, an additional constrain that takes into consideration the number of valence electrons is to be regarded. This aim is to be accomplished observing that the previous atomic electronegativity semiclassical (valence) formulation (263) fits with the definition of chemical action of Equation (5) for the Coulombic potential [115]

(403) $$\chi (N,Z)\;=\;\langle \frac{1}{r}\rangle \;=\;\int \left\{\rho (N,Z,r)\frac{1}{r}\right\}dr\;=\;-\int \{\rho (N,Z,r)V_{Clb}(r)\}dr\;\equiv \;-C_{A}^{Clb}$$

with *Z* being the nuclear charge.

**Figure 7.**
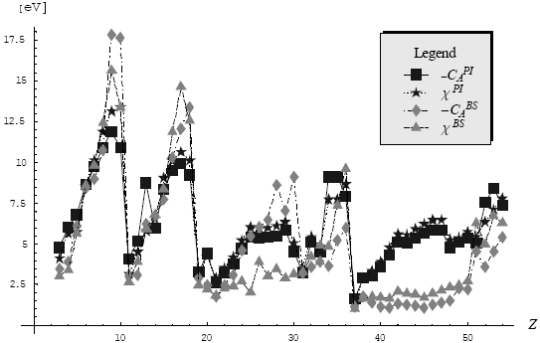
The comparative representation of the atomic electronegativities’ values computed upon Equation (400) and the corresponding absolute chemical actions – given by Equation (5) – using the path integral (PI) and basis set (BS) methods.

**Figure 8.**
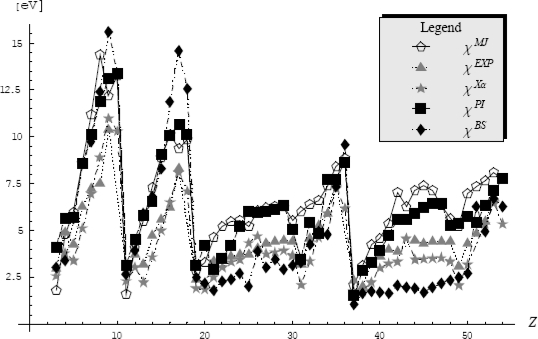
The comparative representation of the atomic electronegativities of Mulliken-Jaffe (MJ), experimental (EXP), and transition state (Xα) respecting the chemical Mulliken one (400) with the recorded values of Table 4 by path integral (PI) and basis set (BS) methods.

The results for the chemical action (403) computed by the two above-mentioned quantum computational schemes are collected in Table 3 with those for the electronegativity (400) in Table 4, among other significant scales. All data are comparatively represented in Figures 7 and 8.

Analyzing these results, it is clear that for the path integral approach better correlation between the electronegativity and the chemical action trends is obtained as comparing with those arising from the s-basis set implementation. The highest discrepancy between the chemical action and the associated electronegativity within the s-basis set computation appears mostly for the first transitional row, see Figure 7.

However, we can not exclude the s-basis set electronegativity scale just through comparison between scales since other similar discrepancies appear (even in the main groups) when the Mulliken-Jaffe and the Xα methods are compared, for instance (see the Table 4). In any case, the present s-basis set results may help in judging also the various criteria of validity for an electronegativity scale, see Section 4.5.

**Figure 9.**
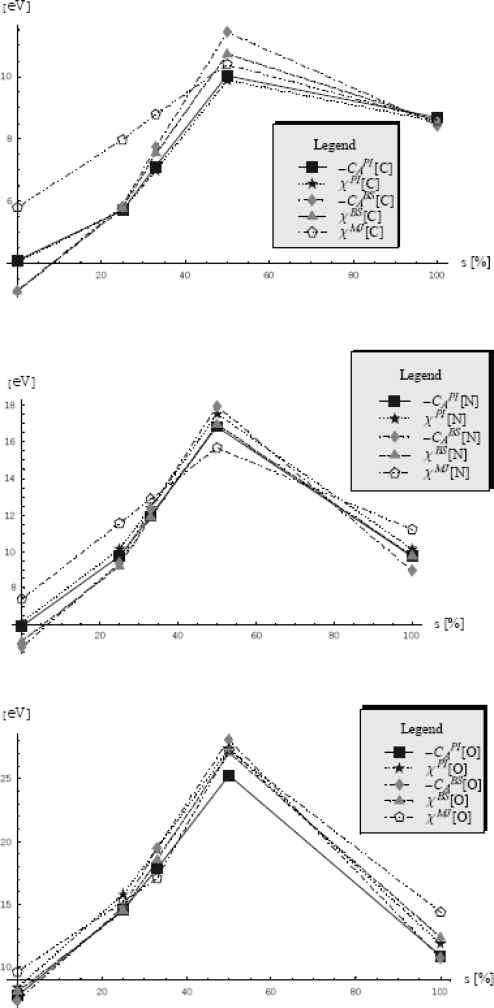
From top to bottom, the representations of the orbital electronegativities and of the absolute chemical actions for C, N and O atoms versus the different percent contribution of s orbital (p: 0%, sp^3^: 25%, sp^2^: 33%, sp: 50% and s: 100%) in pseudo-potentials and basis set frameworks of electronic densities computation with path integral (PI), basis set (BS), and Mulliken-Jaffe (MJ) results of Table 5.

However, it remains to show that the employed chemical action criteria (403) do not enter in conflict with the type of the orbital choice, when this is properly done. For instance, we consider the atomic systems of C, N, O with the s- and p- orbital type basis set and also the sp, sp^2^ and sp^3^ hybridization states. Then, by applying the re-scaling procedure according with the chemical action - electronegativity rule (403) we get the respective electronegativities and chemical actions for both the path integral and basis set implementations using the pseudopotential data [109], as shown in Table 5 and drawn in Figure 9.

They, nevertheless reveal how close the values of the chemical actions and corresponding orbital electronegativities are, in general. Such feature is susceptible for extension in treating the chemical bonds as was recently employed [20,116].

## 6. Non-equilibrium Path Integral of Evolution Amplitude

### 6.1. Levels of Non-equilibrium Dynamics

The electronic states associated to the cyclic reactions, oscillatory chemical phenomena, systems with instabilities, etc., are modeled by the dynamics of discrete states subjected to the general master equation [117–121]

(404a) $$\frac{\partial W_{n}}{\partial t_{b}}\;=\;\sum_{m}\left[w(m\;\to \;n)W_{m}\;-\;w(n\;\to \;m)W_{n}\right]$$

or, for the continuous transformation of states – by the quantum evolution equation

(404b) $$\frac{\partial W(x_{b},\;t_{b})}{\partial t_{b}}\;=\;\int [w(x_{b}^{\#}\;\to \;x_{b})W(x_{b}^{\#},t_{b})\;-\;w(x_{b}\;\to \;x_{b}^{\#})W(x_{b},\;t_{b})]dx_{b}^{\#}$$

where *W_n_* *or W* represent the discrete or continuous probability density, respectively, while *w* describes the transition probability; yet, when it is given by the (diffusion) distribution

(405) $$w(x_{b}^{\#}\;\to \;x_{b})\;=\;\left[-\frac{\partial }{\partial x_{b}}D^{\left(1\right)}(x_{b})\;+\;\frac{\partial^{2}}{\partial x_{b}^{2}}D^{\left(2\right)}(x_{b})\right]\delta (x_{b}\;-\;x_{b}^{\#})$$

the filtration property of Dirac functions (225b) and the obvious relationships

(406) $$\frac{\partial }{\partial x_{b}^{\#}}W(x_{b},\;t_{b})\;=\;\frac{\partial^{2}}{\partial x_{b}^{{\#}^{\prime }2}}W(x_{b},\;t_{b})\;=\;0$$

specialize the Equation (404b) into the celebrated *Fokker-Planck (FP) equation* with the form [122]

(407a) $$\frac{\partial W(x_{b},\;t_{b})}{\partial t_{b}}\;=\left[-\frac{\partial }{\partial x_{b}}D^{\left(1\right)}(x_{b})+\frac{\partial^{2}}{\partial x_{b}^{2}}D^{\left(2\right)}(x_{b})\right]W(x_{b},t_{b})$$

In essence, Fokker-Planck equation reflects the evolution of the quantum fluctuations (or of the probability density) under the external applied potential *V* (*x*) by individuating the drift factor

(407b) $$D^{\left(1\right)}(x_{b})\;=\;-\frac{d}{dx_{b}}V(x_{b})$$

while being accompanied by the diffusion factor *D*^(2)^ (*x*) also known as the stochastic noise [123–125].

Such description of open electronic systems corresponds with the probability density level of non-equilibrium evolution. It originates in modeling of the so called Markovian processes by the reactive chain

(408) $$\bar{\text{A}}\text{(}x_{a},\;t_{a}\text{)}\overset{e^{-}}{\to }\bar{\text{B}}(x_{b},t_{b})\overset{e^{-}}{\to }\bar{\text{C}}(x_{c},t_{c})\overset{e^{-}}{\to }\ldots$$

along which, the electronic evolution is equivalent with the transformation of the probability density converted into extended propagators

(409) $$W_{1}(x_{a},t_{a})\;\to \;W_{2}(x_{b},t_{b};x_{a},t_{a})\;\to \;W_{3}(x_{c},t_{c};x_{b},t_{b};x_{a},t_{a})\to \ldots$$

with correlated successively events. The pairing of the neighboring events is accommodated within the density matrix, the quantum propagator, or the *conditioned probability density ρ*(*x_b_*, *t_b_*; *x_a_*, *t_a_*) featuring the basic characteristics

(410a) $$\rho (x_{b},t_{b};x_{a},t_{a})\;\geq \;0$$

(410b) $$\int dx_{a}\rho (x_{b},t_{b};x_{a},t_{a})\;=\;1$$

(410c) $$W_{1}(x_{b},t_{b})\;=\;\int dx_{a}\rho (x_{b},t_{b};x_{a},t_{a})W_{1}(x_{a},t_{a})$$

(410d) $$W_{2}(x_{b},t_{b};x_{a},t_{a})\;=\;\rho (x_{b},t_{b};x_{a},t_{a})W_{1}(x_{a},t_{a})$$

It is worth noting that for the Markovian processes all relevant information is contained in the first two probability functions *W*_1_ & *W*_2_ being these involved in all successive correlated events; as such, with the properties (410) they may produce any desired extended propagator. For example, one could be immediately write that

(411a) $$\begin{array}{c} W_{2}(x_{c},t_{c};x_{a},t_{a})\;=\;\int dx_{b}W_{3}((x_{c},t_{c};x_{b},t_{b};x_{a},t_{a})\\ =\;\int dx_{b}\rho (x_{c},t_{c};x_{b},t_{b})\rho (x_{b},t_{b};x_{a},t_{a})W_{1}(x_{a},t_{a}) \end{array}$$

which, while equated with the equivalent one

(411b) $$W_{2}(x_{c},t_{c};x_{a},t_{a})\;=\;\rho (x_{c},t_{c};x_{a},t_{a})W_{1}(x_{a},t_{a})$$

provides the so called Chapman-Kolmogorov-Smoluchowski (CKS) equation

(412) $$\rho (x_{c},t_{c};x_{a},t_{a})\;=\;\int dx_{b}\rho (x_{c},t_{c};x_{b},t_{b})\rho (x_{b},t_{b};x_{a},t_{a})$$

in close correspondence with the group property of propagators (141).

Now, in terms of conditioned probability density, *i.e.*, by using the property (410c), the Fokker-Planck equation (407a) rewrites as

(413) ∂ρ(xb,tb;xa,ta)∂tb = [−∂∂xbD(1)(xb)+∂2∂xb2D(2)(xb)]ρ(xb,tb;xa,ta)

Very interesting, the Fokker-Planck equation (413) may be resumed under the hydrodynamic form

(414) $$\frac{\partial \rho (x_{b},t_{b};x_{a},t_{a})}{\partial t_{b}}+\frac{\partial }{\partial x_{b}}j_{FP}(x_{b},t_{b};x_{a},t_{a})\;=\;0$$

through introducing the current of probability density

(415) $$j_{FP}(x_{b},t_{b};x_{a},t_{a})\;=\;D^{\left(1\right)}(x_{b})\rho (x_{b},t_{b};x_{a},t_{a})-\frac{\partial }{\partial x_{b}}[D^{\left(2\right)}(x_{b})\rho (x_{b},t_{b};x_{a},t_{a})]$$

Next, going to analyze the boundary conditions, under the natural assumption

(416) $${\rho (x_{b},t_{b};x_{a},t_{a})|}_{x_{b}\to \pm \infty }\;=\;0$$

the conditioned current limit automatically results from Equation (415)

(417) $${j_{FP}(x_{b},t_{b};x_{a},t_{a})|}_{x_{b}\to \pm \infty }\;=\;0$$

leaving the Equation (414) with the conservative form

(418) $$\frac{\partial }{\partial t_{b}}\int_{-\infty }^{+\infty }dx_{b}\rho (x_{b},t_{b};x_{a},t_{a})\;=\;0$$

the last equation is solved for the non-equilibrium propagator canonical normalization

(419) $$\int_{-\infty }^{+\infty }dx_{b}\rho (x_{b},t_{b};x_{a},t_{a})\;=\;1$$

in accordance with the previous property (410b) for the conditioned probability density. Therefore, the Fokker-Planck equation contains the entire information of the quantum evolution of the open systems, with drift and diffusion.

On the other hand, for the initial condition of probability density identified with the Dirac distribution

(420a) $$W_{1}(x_{a}^{\#},t_{a})\;=\;\delta (x_{a}\;-\;x_{a}^{\#})$$

the property (410c) yields the predicted probability density

(420b) $$W_{1}(x_{b},t_{b})\;=\;\rho (x_{b},t_{b};x_{a},t_{a})$$

as the working propagator form.

Alternatively, if the initial condition is somehow relaxed to the Gaussian form

(421a) $$W_{1}(x_{a},t_{a})\;=\;\frac{1}{\sqrt{2\pi }}\text{exp}\left(-\frac{1}{2}x_{a}^{2}\right)$$

to which any continuous distribution is reduced according with the central limit theorem, then, the same general property (410c) prescribes the Markovian probability density of the type

(421b) $$W_{1}(x_{b},t_{b})\;=\frac{1}{\sqrt{2\pi }}\int dx_{a}\rho (x_{b},t_{b};x_{a},t_{a})\text{exp}\left(-\frac{1}{2}x_{a}^{2}\right)$$

as a sort of smearing for the quantum propagator, being this behavior the specific characteristic for the non-equilibrium systems where fluctuations should be averaged in order to produce predicted probabilities. In either of cases, Equations (420b) or (421b), the working Markovian probability current (415) unfolds like

(422) $$j_{FP}(x_{b},t_{b})\;=\;D^{\left(1\right)}(x_{b})W_{1}(x_{b},t_{b})-\frac{\partial }{\partial x_{b}}[D^{\left(2\right)}(x_{b})W_{1}(x_{b},t_{b})]$$

staying as the basis for the analytical representation of the non-equilibrium electro-reactive dynamics in open environment, as atoms-in-molecules are, for instance.

### 6.2. Non-equilibrium Lagrangean

Once identified with the quantum propagator, the above probability density may be readily expressed by a special form of the Feynman path integral; namely it may be viewed as the quantum stochastic Fokker-Planck (FP) propagator [126,127]

(423) $$\rho (x_{b}t_{b};x_{a}t_{a})\;\to \;{\left(x_{b}t_{b};x_{a}t_{a}\right)}_{FP}\;=\;\int_{x(t_{a})=x_{a}}^{x(t_{b})=x_{b}}D^{\prime }x(t)L_{FP}(x(t),\dot{x}(t),t)$$

marking the appearance of the third form of path integral representation, after those specific to the quantum mechanics and quantum statistics, see Equations (121a) and (121b), respectively. Nevertheless, the stochastic Lagrangean in (423) assumes the general ansatz [48]

(424) $$L_{FP}(x(t),\;\dot{x}(t),t)\;=\;A{\left[\dot{x}(t)+\frac{1}{m\gamma }\;\frac{d}{dx}V\left(x(t)\right)\right]}^{2}\;-\;B\frac{d^{2}}{dx^{2}}V\left(x(t)\right)$$

to be justified in what follows.

To this end, firstly, the density (423) with the Lagrangean (424) is considered within the temporal slicing

(425a) $$ɛ\;=\;t_{n}-t_{n-1}$$

and the coordinate averaging respecting *α* ∈[0,1]

(425b) $${\bar{x}}_{n}\;=\;\alpha x_{n}\;+\;(1-\alpha )x_{n-1}\;=\;x_{n}\;-\;(1-\alpha )(x_{n}-x_{n-1})$$

to produce the elementary propagator

(426) $${\left(x_{n},ɛ;x_{n-1},0\right)}_{FP}\;=\;n_{FP}\;\text{exp}\left\{-Aɛ{\left[\frac{x_{n}\;-\;x_{n-1}}{ɛ}\;+\;\frac{V^{\prime }({\bar{x}}_{n})}{m\gamma }\right]}^{2}\;+\;BɛV^{\prime \prime }({\bar{x}}_{n})\right\}$$

Now, employing the (425b) expansion the potential dependencies in (426) may be as well considered until the second order cut-off series

(427a) $$V^{\prime }({\bar{x}}_{n})\;=\;V^{\prime }[x_{n}\;-\;(1-\alpha )(x_{n}\;-\;x_{n-1})]\;≅\;V^{\prime }(x_{n})\;-\;(1-\alpha )V^{\prime \prime }(x_{n})(x_{n}\;-\;x_{n-1})$$

(427b) $$V^{\prime \prime }({\bar{x}}_{n})\;≅\;V^{\prime \prime }(x_{n})$$

so that the probability density property (410c), forgetting the subscript – since no reason for confusion, becomes:

(428) $$\begin{array}{c} W(x_{n},t_{n})\;=\;\int_{-\infty }^{+\infty }dx_{n-1}{\left(x_{n},ɛ;x_{n-1},0\right)}_{FP}W(x_{n-1},t_{n-1})\\ =\int_{-\infty }^{+\infty }d(\Delta x_{n})n_{FP}W(x_{n}\;-\;\Delta x_{n},t_{n}\;-\;ɛ)\text{exp}\left\{-\text{A}ɛ{\left[\frac{\Delta x_{n}}{ɛ}+\frac{V^{\prime }(x_{n})-(1-\alpha )V^{\prime \prime }(x_{n})\Delta x_{n}}{m\gamma }\right]}^{2}\;+\;\text{B}ɛV^{\prime \prime }(x_{n})\right\} \end{array}$$

where we used the notations

(429a) $$t_{n-1}\;=\;t_{n}\;-\;ɛ$$

(429b) $$x_{n-1}\;=\;x_{n}\;-\;\Delta x_{n}$$

Next, based on the observation that

(430) $$\text{exp}\left\{-A\int_{t_{a}}^{t_{b}}dt{\dot{x}}^{2}\right\}\;≅\;\text{exp}\left\{-Aɛ\frac{{\left(\Delta x\right)}^{2}}{{ɛ}^{2}}\right\}$$

it follows the coordinate displacement proportionality

(431a) $$\Delta x_{n}\;\propto \;\sqrt{ɛ}$$

which, along the temporal abstracted counterpart from Equation (425a)

(431b) Δt ∝ ɛ

allows the first order expansion in *ε* for the elementary probability density

(432) $$W(x_{n}\;-\;\Delta x_{n},t_{n}\;-\;ɛ)\;≅\;W(x_{n},t_{n})-\Delta x_{n}\frac{\partial W(x_{n},t_{n})}{\partial x_{n}}+\frac{1}{2}{\left(\Delta x_{n}\right)}^{2}\frac{\partial^{2}W(x_{n},t_{n})}{\partial x_{n}^{2}}-ɛ\frac{\partial W(x_{n},t_{n})}{\partial t_{n}}$$

Through keeping only the terms until the first order in*ε*, with Equation (432) back in identity (428), one successively gets

(433) $$\begin{array}{c} W(x_{n},t_{n})\;≅\;\int_{-\infty }^{+\infty }d(\Delta x_{n})n_{FP}\text{exp}\left[-A\frac{{\left(\Delta x_{n}\right)}^{2}}{ɛ}\right]\\ \times \;\left[1-2\frac{A}{m\gamma }V^{\prime }(x_{n})\Delta x_{n}\;-\;Aɛ\frac{V^{\prime }{\left(x_{n}\right)}^{2}}{m^{2}\gamma^{2}}\;+\;\frac{2A(1-\alpha )V^{\prime \prime }(x_{n}){\left(\Delta x_{n}\right)}^{2}}{m\gamma }\;+\;BɛV^{\prime \prime }(x_{n})\;+\;\frac{2A^{2}}{m^{2}\gamma^{2}}V^{\prime }{\left(x_{n}\right)}^{2}{\left(\Delta x_{n}\right)}^{2}\right]\\ \times \left[W(x_{n},t_{n})-\Delta x_{n}\frac{\partial W(x_{n},t_{n})}{\partial x_{n}}\;+\;\frac{1}{2}{\left(\Delta x_{n}\right)}^{2}\frac{\partial^{2}W(x_{n},t_{n})}{\partial x_{n}^{2}}-ɛ\frac{\partial W(x_{n},t_{n})}{\partial t_{n}}\right]\\ =\;\int_{-\infty }^{+\infty }d(\Delta x_{n})n_{FP}\;\text{exp}\left[-A\frac{{\left(\Delta x_{n}\right)}^{2}}{ɛ}\right]\\ \times \;[W(x_{n},t_{n})-2\frac{A}{m\gamma }V^{\prime }(x_{n})W(x_{n},t_{n})\Delta x_{n}-Aɛ\frac{V^{\prime }{\left(x_{n}\right)}^{2}}{m^{2}\gamma^{2}}W(x_{n},t_{n})\\ +\;\frac{2A(1-\alpha )V^{\prime \prime }(x_{n})}{m\gamma }{\left(x_{n}\right)}^{2}W(x_{n},t_{n})+BɛV^{\prime \prime }(x_{n})W(x_{n},t_{n})+\frac{2A^{2}}{m^{2}\gamma^{2}}V^{\prime }{\left(x_{n}\right)}^{2}{\left(\Delta x_{n}\right)}^{2}W(x_{n},t_{n})\\ -\Delta x_{n}\frac{\partial W(x_{n},t_{n})}{\partial x_{n}}\;+\;2\frac{A}{m\gamma }V^{\prime }(x_{n}){\left(\Delta x_{n}\right)}^{2}\frac{\partial W(x_{n},t_{n})}{\partial x_{n}}+\frac{1}{2}{\left(\Delta x_{n}\right)}^{2}\frac{\partial^{2}W(x_{n},t_{n})}{\partial x_{n}^{2}}\;-\;ɛ\frac{\partial W(x_{n},t_{n})}{\partial t_{n}} \end{array}$$

Some of the terms in (433) vanish due to the odd powers of integrand under the Poisson integrals

(434a) $$\int_{-\infty }^{+\infty }d(\Delta x_{n})\Delta x_{n}\text{exp}\left[-\frac{A}{ɛ}{\left(\Delta x_{n}\right)}^{2}\right]\;=\;0$$

while those integrating over the even powers of integrand leads with the solutions

(434b) $$1\;=\;\int_{-\infty }^{+\infty }d(\Delta x_{n})n_{FP}\text{exp}\left[-\frac{A}{ɛ}{\left(\Delta x_{n}\right)}^{2}\right]\;=\;n_{FP}\sqrt{\frac{ɛ\pi }{A}}\;\Rightarrow \;n_{FP}\;=\;\sqrt{\frac{A}{ɛ\pi }}$$

(434c) $$\int_{-\infty }^{+\infty }d(\Delta x_{n})n_{FP}{\left(\Delta x_{n}\right)}^{2}\text{exp}\left[-\frac{A}{ɛ}{\left(\Delta x_{n}\right)}^{2}\right]\;=\;\frac{ɛ}{2A}$$

With these rules, the expression (433) simplifies to

(435) $$\begin{array}{c} W(x_{n},t_{n})\;=\;W(x_{n},t_{n})-Aɛ\frac{V^{\prime }{\left(x_{n}\right)}^{2}}{m^{2}\gamma^{2}}W(x_{n},t_{n})+\frac{ɛ}{2A}\;\frac{2A(1-\alpha )V^{\prime \prime }(x_{n})}{m\gamma }W(x_{n},t_{n})\\ +\;BɛV^{\prime \prime }(x_{n})W(x_{n},t_{n})+\frac{ɛ}{2A}\frac{2A^{2}}{m^{2}\gamma^{2}}V^{\prime }{\left(x_{n}\right)}^{2}W(x_{n},t_{n})+2\frac{A}{m\gamma }V^{\prime }(x_{n})\frac{ɛ}{2A}\frac{\partial W(x_{n},t_{n})}{\partial x_{n}}\\ +\frac{1}{2}\;\frac{ɛ}{2A}\;\frac{\partial^{2}W(x_{n},t_{n})}{\partial x_{n}^{2}}\;-\;ɛ\frac{\partial W(x_{n},t_{n})}{\partial t_{n}} \end{array}$$

or even more as

(436) $$\frac{\partial W(x_{n},t_{n})}{\partial t_{n}}\;=\;\frac{V^{\prime }(x_{n})}{m\gamma }\frac{\partial W(x_{n},t_{n})}{\partial x_{n}}+\left[BV^{\prime \prime }(x_{n})+\frac{\left(1-\alpha )V^{\prime \prime }(x_{n}\right)}{m\gamma }\right]W(x_{n},t_{n})+\frac{\partial^{2}}{\partial x_{n}^{2}}\left[\frac{1}{4A}W(x_{n},t_{n})\right]$$

While noting the striking correspondence between the Equations (436) and (407a) the system (437) is immediately established

(437) $$\{\begin{array}{l} \frac{1}{4A}\;=\;D\\ D^{\left(1\right)}(x_{n})\;=\;-\frac{V^{\prime }(x_{n})}{m\gamma }\\ D^{\left(1\right)}\prime (x_{n})=-BV^{\prime \prime }(x_{n})-\frac{\left(1-\alpha )V^{\prime \prime }(x_{n}\right)}{m\gamma } \end{array}$$

The unique solution of the system (437) stands as

(438) $$\{\begin{array}{l} A\;=\;\frac{1}{4D}\\ B\;=\;\frac{\alpha }{m\gamma } \end{array}$$

while the midpoint case *α* = 1/ 2 providing the stochastic Lagrangean (424) explicitly written in terms of the friction and diffusion parameters *γ* & *D*, respectively

(439) $$L_{FP}(x(t),\;\dot{x}(t),t)\;=\;\frac{1}{4D}{\left[\dot{x}(t)\;+\;\frac{1}{m\gamma }V^{\prime }(x(t))\right]}^{2}\;-\;\frac{1}{2m\gamma }V^{\prime \prime }(x(t))$$

Finally, worth remarking that because the quantum Planck constant *ħ* is apparently absent in the Fokker-Planck approach - it seems more appropriately called as *mesoscopic picture*, thus laying between the quantum microscopic and Newtonian macroscopic pictures of natural systems. It nevertheless works for assessing new insight of the electronic behavior at the atomic and molecular levels by means of the averaged harmonic and anharmonic fluctuations – as is to be revealed below.

### 6.3. Harmonic Markovian Density Matrix

Harmonic solution of non-equilibrium propagator is to be found by using the method of path integrals, starting from the associated Lagrangean explicated in the previous paragraph. It starts from the expression of conditioned density of probability in the form of path integral (423) with (439) [48]

(440) $${\left(x_{b},t_{b};x_{a},t_{a}\right)}_{FP}\;=\;\int_{x(t_{a})\;=\;x_{a}}^{x(t_{b})\;=\;x_{b}}D^{\prime }x(t)\text{exp}\left\{-\frac{1}{4D}\int_{t_{a}}^{t_{b}}dt{\left[\dot{x}(t)\;+\;\frac{1}{m\gamma }V^{\prime }(x(t))\right]}^{2}\;+\;\frac{1}{2m\gamma }\int_{t_{a}}^{t_{b}}dtV^{\prime \prime }(x(t))\right\}$$

that may be formally simplified by considering the spectral shift of eigen-FP values introducing the potential transformation

(441) V(x) → mγU(x)

to become

(442) $${\left(x_{b},t_{b};x_{a},t_{a}\right)}_{FP}\;=\;\int_{x(t_{a})=x_{a}}^{x(t_{b})=x_{b}}D^{\prime }x(t)\text{exp}\left\{-\frac{1}{4D}\int_{t_{a}}^{t_{b}}dt{\left[\dot{x}(t)\;+\;U^{\prime }(x(t))\right]}^{2}\;+\;\frac{1}{2}\int_{t_{a}}^{t_{b}}dtU^{\prime \prime }(x(t))\right\}$$

The form (442) is next employed for the Onsanger-Matchlup harmonic potential (equivalent with the Brownian motion) [128]

(443) $$U(x)\;=\;\frac{\gamma }{2}x^{2}$$

to equivalently become

(444) $$\begin{array}{c} {\left(x_{b},t_{b};x_{a},t_{a}\right)}_{FP}^{x^{2}}\;=\;\int_{x(t_{a})=x_{a}}^{x(t_{b})=x_{b}}D^{\prime }x(t)\text{exp}\left\{-\frac{1}{4D}\int_{t_{a}}^{t_{b}}dt[{\dot{x}}^{2}(t)+2\gamma \dot{x}(t)x(t)+\gamma^{2}x^{2}(t)]\right\}\text{exp}\left[\frac{\gamma }{2}(t_{b}-t_{a})\right]\\ =\text{exp}\left[\frac{\gamma }{2}(t_{b}-t_{a})\right]\text{exp}\left[-\frac{\gamma }{4D}(x_{b}^{2}-x_{a}^{2})\right]\int_{x(t_{a})=x_{a}}^{x(t_{b})=x_{b}}D^{\prime }x(t)\text{exp}\left\{-\frac{1}{2D}\int_{t_{a}}^{t_{b}}dt\left[\frac{1}{2}{\dot{x}}^{2}(t)+\frac{1}{2}\gamma^{2}x^{2}(t)\right]\right\}\\ =\;\text{exp}\left[\frac{\gamma }{2}(t_{b}\;-\;t_{a})\right]\text{exp}\left[-\frac{\gamma }{4D}(x_{b}^{2}\;-\;x_{a}^{2})\right]{\left(x_{b},t_{b};x_{a},t_{a}\right)}_{FP}^{x^{2}[0]} \end{array}$$

If the harmonic correspondences are advanced

(445) $$\{\begin{array}{l} \gamma \;↔\;\omega \\ \frac{1}{2D}\;↔\frac{m}{\hbar } \end{array}$$

the remained path integral ${\left(x_{b},t_{b};x_{a},t_{a}\right)}_{FP}^{x^{2}\left[0\right]}$ may be at once evaluated by considering them in the form (185) along the transformations (188) with the result

(446) (xb,tb;xa,ta)FPx2 = exp[γ2(tb−ta)]exp[−γ4D(xb2−xa2)]×γ4Dπsinh[γ(tb − ta)]exp{−γ[(xb2+xa2)cosh[γ(tb−ta)]−2xaxb]4Dsinh[γ(tb−ta)]}

Doing the elementary algebraic transformation the hyperbolic functions become exponentials so leaving the harmonic solution of the non-equilibrium path integral with the form

(447) $${\left(x_{b},t_{b};x_{a},t_{a}\right)}_{FP}^{x^{2}}\;=\;\frac{1}{\sqrt{2\pi \frac{D}{\gamma }\{1\;-\;\text{exp}[-2\gamma (t_{b}-t_{a})]\}}}\text{exp}\left\{-\frac{{\left[x_{b}-x_{a}\;\text{exp}[-\gamma (t_{b}-t_{a})]\right]}^{2}}{2\frac{D}{\gamma }[1-\text{exp}[-2\gamma (t_{b}-t_{a})]]}\right\}$$

that readily satisfies the reduced (by the constant factor *mγ*) FP equation

(448) $$\frac{\partial {\left(x_{b},t_{b};x_{a},t_{a}\right)}_{FP}^{x^{2}}}{\partial t_{b}}\;=\;\frac{\partial }{\partial x_{b}}\;\left[(\gamma x_{b}){\left(x_{b},t_{b};x_{a},t_{a}\right)}_{FP}^{x^{2}}\right]\;+\;D\frac{\partial^{2}}{\partial x_{b}^{2}}\left[{\left(x_{b},t_{b};x_{a},t_{a}\right)}_{FP}^{x^{2}}\right]$$

obtained from equation (413) with the drift Equation (407b) re-shaped with the potential (443), while considering for the diffusion coefficient *D*^(2)^ (*x*_b_) the constant *D*.

### 6.4. Anharmonic Markovian Density Matrix

The next challenge stays the finding of non-equilibrium propagator for the anharmonic generalized potential

(449) $$U(x)\;=\;\gamma \frac{x^{2}}{2}\;+\;g\frac{x^{4}}{4}$$

Viewed as a perturbation, the transition probability or the stochastic propagator (442) will be calculated by neglecting all the contributions *g^n^*, *n* ≥ 2, successively as [48]

(450) $$\begin{array}{c} {\left(x_{b},t_{b};x_{a},t_{a}\right)}_{FP}^{x^{4}}\;=\;\int_{x(t_{a})=x_{a}}^{x(t_{b})=x_{b}}D^{\prime }x(t)\text{exp}\left\{-\frac{1}{4D}\int_{t_{a}}^{t_{b}}dt{\left[\dot{x}(t)+\gamma x(t)+gx{\left(t\right)}^{3}\right]}^{2}+\frac{1}{2}\int_{t_{a}}^{t_{b}}dt\left[\gamma +3gx{\left(t\right)}^{2}\right]\right\}\\ =\int_{x(t_{a})=x_{a}}^{x(t_{b})=x_{b}}D^{\prime }x(t)\text{exp}\{-\frac{1}{4D}\int_{t_{a}}^{t_{b}}dt\left[{\dot{x}}^{2}(t)+2\gamma \dot{x}(t)x(t)+\gamma^{2}x^{2}(t)+2g\dot{x}(t)x^{3}(t)+2g\gamma x^{4}(t)\right]\\ +\frac{1}{2}\int_{t_{a}}^{t_{b}}dt\left[3gx^{2}(t)\right]+\frac{1}{2}\gamma \left(t_{b}-t_{a}\right)\}\\ =\text{exp}\left[\frac{1}{2}\gamma \left(t_{b}-t_{a}\right)\right]]\text{exp}\left[-\frac{1}{4D}\gamma \left(x_{b}^{2}-x_{a}^{2}\right)\right]\text{exp}\left[-\frac{g}{8D}\left(x_{b}^{4}-x_{a}^{4}\right)\right]\\ \times \int_{x(t_{a})=x_{a}}^{x(t_{b})=x_{b}}D^{\prime }x(t)\text{exp}\left\{-\frac{1}{4D}\int_{t_{a}}^{t_{b}}dt\left[{\dot{x}}^{2}(t)+\gamma^{2}x^{2}(t)\right]\right\}\text{exp}\left[-\frac{g\gamma }{2D}\int_{t_{a}}^{t_{b}}dtx^{4}(t)+\frac{3g}{2}\int_{t_{a}}^{t_{b}}dtx^{2}(t)\right]\\ ≅\text{exp}\left\{\frac{1}{2}\gamma \left(t_{b}-t_{a}\right)-\frac{1}{4D}\gamma \left(x_{b}^{2}-x_{a}^{2}\right)-\frac{g}{8D}\left(x_{b}^{4}-x_{a}^{4}\right)\right\}\\ \times \;{\left(x_{b},t_{b};x_{a},t_{a}\right)}_{FP}^{x^{2}[0]}\left[1-\frac{\gamma g}{2D}\int_{t_{a}}^{t_{b}}dt〈x^{4}(t)〉+\frac{3g}{2}\int_{t_{a}}^{t_{b}}dt〈x^{2}(t)〉\right]\\ ={\left(x_{b},t_{b};x_{a},t_{a}\right)}_{FP}^{x^{2}}\left[1-\frac{\gamma g}{2D}\int_{t_{a}}^{t_{b}}dt〈x^{4}(t)〉+\frac{3g}{2}\int_{t_{a}}^{t_{b}}dt〈x^{2}(t)〉\right]\text{exp}\left[-\frac{g}{8D}\left(x_{b}^{4}-x_{a}^{4}\right)\right]\\ ={\left(x_{b},t_{b};x_{a},t_{a}\right)}_{FP}^{x^{2}}\left\{1+g\left[\frac{3}{2}\int_{t_{a}}^{t_{b}}dt〈x^{2}(t)〉-\frac{\gamma }{2D}\int_{t_{a}}^{t_{b}}dt〈x^{4}(t)〉-\frac{1}{8D}\left(x_{b}^{4}-x_{a}^{4}\right)\right]\right\}\\ \equiv {\left(x_{b},t_{b};x_{a},t_{a}\right)}_{FP}^{x^{2}}+g{\left(x_{b},t_{b};x_{a},t_{a}\right)}_{FP}^{x^{2}\wedge x^{4}} \end{array}$$

Note that the appeared averages in Equation (450) are rooting in the same procedure as that applied for developing the expectation values in Equations (207) or (211) and (212); here, for the stochastic action and Lagrangean

(451a) $$S_{FP}^{x^{2}}\left[x(t),\dot{x}(t),t\right]\;=\;\int_{t_{a}}^{t_{b}}L_{FP}^{x^{2}}\left[x(t),\dot{x}(t),t\right]$$

the single correlated function is adapted as

(451b) $$\begin{array}{c} {〈x(t)〉}_{FP}=\frac{\int_{x(t_{a})=x_{a}}^{x(t_{b})=x_{b}}D^{\prime }x(t)[x(t)]\text{exp}\left\{-S_{FP}^{x^{2}}[x(t),\dot{x}(t),t]\right\}}{\int_{x(t_{a})=x_{a}}^{x(t_{b})=x_{b}}D^{\prime }x(t)\text{exp}\left\{-S_{FP}^{x^{2}}[x(t),\dot{x}(t),t]\right\}}\\ \frac{{\frac{\delta }{\delta j}\int_{x(t_{a})=x_{a}}^{x(t_{b})=x_{b}}D^{\prime }x(t)[x(t)]\text{exp}\left\{-\left(S_{FP}^{x^{2}}[x(t),\dot{x}(t),t]+\int_{t_{a}}^{t_{b}}dtx(t)j(t)\right)\right\}|}_{j=0}}{\int_{x(t_{a})=x_{a}}^{x(t_{b})=x_{b}}D^{\prime }x(t)\text{exp}\left\{-S_{FP}^{x^{2}}[x(t),\dot{x}(t),t]\right\}}\\ =-\frac{{\frac{\delta }{\delta j(t)}{\left(x_{b},t_{b};x_{a},t_{a}\right)}_{FP}^{x^{2}[0]}[j(t)]|}_{j=0}}{{\left(x_{b},t_{b};x_{a},t_{a}\right)}_{FP}^{x^{2}[0]}} \end{array}$$

while supporting the multi-correlated functions’ generalization

(451c) $${〈x(t_{1})x(t_{2})\ldots 〉}_{FP}\;=\;\frac{{\left(-\frac{\delta }{\delta j(t_{1})}\right)\left(-\frac{\delta }{\delta j(t_{2})}\right)\cdots {\left(x_{b},t_{b};x_{a},t_{a}\right)}_{FP}^{x^{2}[0]}[j(t)]|}_{j=0}}{{\left(x_{b},t_{b};x_{a},t_{a}\right)}_{FP}^{x^{2}[0]}}$$

in terms of the density matrix functional of the introduced interaction current *j*. Through noting from (228) the fact that the product *j·G* transforms as 2*m·x*, while from (211) appears that *j·x* transforms as *m ·* *ẋ*^2^, by quoting the previously deduced expression (232b) with the actual correspondences (445), one has the harmonic Fokker-Planck current functional

(452) $$\begin{array}{c} {\left(x_{b},t_{b};x_{a},t_{a}\right)}_{FP}^{x^{2}[0]}[j(t)]={\left(x_{b},t_{b};x_{a},t_{a}\right)}_{FP}^{x^{2}[0]}\\ \times \text{exp}\left[\frac{1}{2D}\int_{t_{a}}^{t_{b}}dt_{1}x_{cl}(t_{1})j(t_{1})+\frac{1}{4D^{2}}\int_{t_{a}}^{t_{b}}dt_{1}\int_{t_{a}}^{t_{b}}dt_{2}j(t_{1})j(t_{2})G(t_{1},t_{2})\right] \end{array}$$

The involved classical (235) and Green function (248) propagators are rewritten through the correspondences (445) with the factor *ħ*/ *m* of (233b) toward the stochastic picture

(453a) $$x_{cl-FP}(t)\;=\;\frac{x_{b}\text{sinh}[\gamma (t-t_{a})]+x_{a}\text{sinh}[\gamma (t_{b}-t)]}{\text{sinh}[\gamma (t_{b}-t_{a})]}$$

(453b) $$\begin{array}{c} G_{FP}(t_{1},t_{2})\;=\;\frac{2D}{\gamma }\frac{\text{sinh}[\gamma (t_{1}-t_{a})]\text{sinh}[\gamma (t_{b}-t_{2})]}{\text{sinh}[\gamma (t_{b}-t_{a})]}\Theta (t_{2}-t_{1})\\ +\frac{2D}{\gamma }\frac{\text{sinh}[\gamma (t_{2}-t_{a})]\text{sinh}[\gamma (t_{b}-t_{1})]}{\text{sinh}[\gamma (t_{b}-t_{a})]}\Theta (t_{1}-t_{2}) \end{array}$$

with Θ(*t*_1 –_ *t*_2_) the Heaviside function (247).

Now, while employing the Wick correlation rules (220) and (221) for the identical end-point times, one gets the Feynman expressions
(454a)
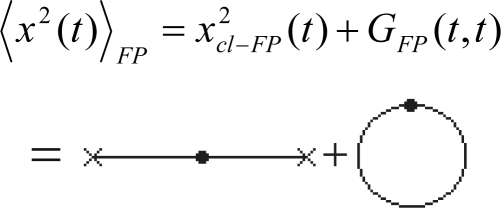

(454b)
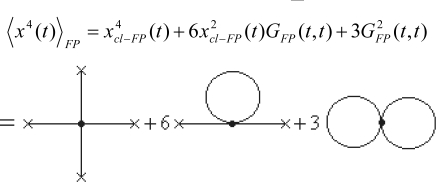
as combinations of the components of Equations (453).

**Figure 10.**
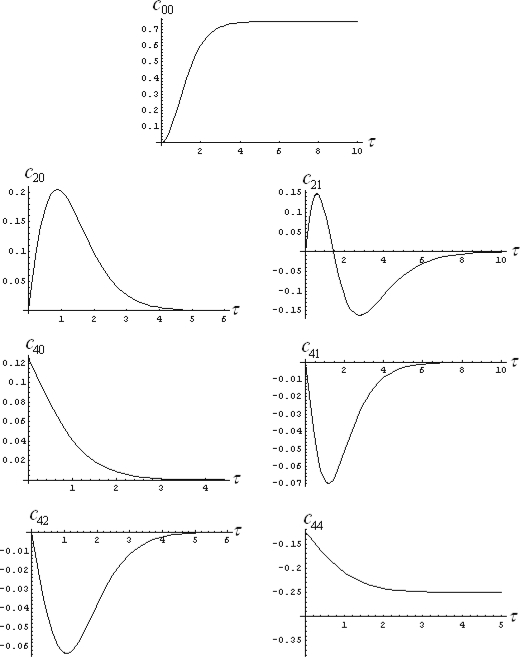
Temporal behavior of the coefficients (456) for the Fokker-Planck anharmonic conditioned probability density (455) for unitary drift and diffusion constants *γ* = *D* = 1 [48].

With expressions (453) in (454) and then all together in (450) one gets the anharmonic non-equilibrium propagator

(455) $$\begin{array}{c} {\left(x_{b},\;t_{b};x_{a},t_{a}\right)}_{FP}^{x^{4}}\;=\;{\left(x_{b},\;t_{b};x_{a},t_{a}\right)}_{FP}^{x2}\\ \times \;\{1\;+\;g[c_{00}(\tau )\;+\;c_{20}(\tau )x_{a}^{2}\;+\;c_{21}(\tau )x_{a}x_{b}\;+\;c_{22}(\tau )x_{b}^{2}\;+\;c_{40}(\tau )x_{a}^{4}\\ +\;c_{41}(\tau )x_{a}^{3}x_{b}\;+\;c_{42}(\tau )x_{a}^{2}x_{b}^{2}\;+\;c_{43}(\tau )x_{a}x_{b}^{3}\;+\;c_{44}(\tau )x_{b}^{4}]\}\\ \equiv \;{\left(x_{b},\;t_{b};x_{a},t_{a}\right)}_{FP}^{x^{2}}\;+\;g{\left(x_{b},\;t_{b};x_{a},t_{a}\right)}_{FP}^{x^{2}}{\left(x_{b},\;t_{b};x_{a},t_{a}\right)}_{FP}^{gx^{4}} \end{array}$$

with the working coefficients [48]

(456a) $$\begin{array}{c} c_{00}(\tau )\;=\;3D\frac{1+4(1-2\tau )\text{exp}(-2\tau )-(5+4\tau )\text{exp}(-4\tau )}{4\gamma^{2}{\left[1-\text{exp}(-2\tau )\right]}^{2}}\\ c_{20}(\tau )\;=\;3\frac{\left(4\tau -5)\text{exp}(-2\tau )+4(1+2\tau )\text{exp}(-4\tau )+\text{exp}(-6\tau \right)}{2\gamma {\left[1-\text{exp}(-2\tau )\right]}^{3}}\;=\;c_{22}(\tau )\\ c_{21}(\tau )\;=\;3\frac{\left(2-\tau )\text{exp}(-\tau )+2(1-4\tau )\text{exp}((-3\tau )-(3\tau +4)\text{exp}(-5\tau \right)}{\gamma {\left[1-\text{exp}(-2\tau )\right]}^{3}}\\ c_{40}(\tau )\;=\;\frac{2\text{exp}(-2\tau )+3(1-4\tau )\text{exp}(-4\tau )-6\text{exp}(-6\tau )+\text{exp}(-8\tau )}{4D{\left[1-\text{exp}(-2\tau )\right]}^{4}}\\ c_{41}(\tau )\;=\;\frac{-\text{exp}(-\tau )+3(4\tau -3)\text{exp}(-3\tau )+3(3+4\tau )\text{exp}(-5\tau )+\text{exp}(-7\tau )}{2D{\left[1-\text{exp}(-2\tau )\right]}^{4}}\;=\;c_{43}(\tau )\\ c_{42}(\tau )\;=\;3\frac{\left(3-2\tau )\text{exp}(-2\tau )-8\tau \text{exp}(-4\tau )-(3+2\tau )\text{exp}(-6\tau \right)}{2D{\left[1-\text{exp}(-2\tau )\right]}^{4}}\\ c_{44}(\tau )\;=\;\frac{-1\;+\;6\text{exp}(-2\tau )-3(1+4\tau )\text{exp}(-4\tau )-2\text{exp}(-6\tau )}{4D{\left[1-\text{exp}(-2\tau )\right]}^{4}} \end{array}$$

having the time behavior depicted in the Figure 10, in terms of the stochastic time interval, here abbreviated as

(456b) $$\tau \;=\;\gamma (t_{b}\;-\;t_{a})$$

Next, we like to check the solution (455) with coefficients (456). This may be done by replacing it into the “reduced” Fokker-Planck Equation (413) with the constant diffusion factor, under the anharmonic potential (449)

(457a) $$\begin{array}{c} \frac{\partial }{\partial t_{b}}\left[{\left(x_{b},t_{b};x_{a},t_{a}\right)}_{FP}^{x^{2}}\;+\;g{\left(x_{b},t_{b};x_{a},t_{a}\right)}_{FP}^{x^{2}}\;{\left(x_{b},t_{b};x_{a},t_{a}\right)}_{FP}^{gx^{4}}\right]\\ =\frac{\partial }{\partial x_{b}}\left\{\left(\gamma x_{b}\;+\;gx_{b}^{3}\right)\left[{\left(x_{b},t_{b};x_{a},t_{a}\right)}_{FP}^{x^{2}}+\;g{\left(x_{b},t_{b};x_{a},t_{a}\right)}_{FP}^{x^{2}}{\left(x_{b},t_{b};x_{a},t_{a}\right)}_{FP}^{gx^{4}}\right]\right\}\\ +D\frac{\partial^{2}}{\partial x_{b}^{2}}\left[{\left(x_{b},t_{b};x_{a},t_{a}\right)}_{FP}^{x^{2}}\;+\;g{\left(x_{b},t_{b};x_{a},t_{a}\right)}_{FP}^{x^{2}}{\left(x_{b},t_{b};x_{a},t_{a}\right)}_{FP}^{gx^{4}}\right] \end{array}$$

which, after limitation to the first order in perturbation (coupling) parameter *g* it takes the form

(457b) $$\begin{array}{c} \frac{\partial }{\partial t_{b}}{\left(x_{b},t_{b};x_{a},t_{a}\right)}_{FP}^{x^{2}}\;+\;g\frac{\partial }{\partial t_{b}}\left[{\left(x_{b},t_{b};x_{a},t_{a}\right)}_{FP}^{x^{2}}\;{\left(x_{b},t_{b};x_{a},t_{a}\right)}_{FP}^{gx^{4}}\right]\\ =\frac{\partial }{\partial x_{b}}\left[\gamma x_{b}{\left(x_{b},t_{b};x_{a},t_{a}\right)}_{FP}^{x^{2}}\right]+g\frac{\partial }{\partial x_{b}}\left[\gamma x_{b}{\left(x_{b},t_{b};x_{a},t_{a}\right)}_{FP}^{x^{2}}\;{\left(x_{b},t_{b};x_{a},t_{a}\right)}_{FP}^{gx^{4}}\right]\\ +\;g\frac{\partial }{\partial x_{b}}\left[x_{b}^{3}{\left(x_{b},t_{b};x_{a},t_{a}\right)}_{FP}^{x^{2}}\right]+D\frac{\partial^{2}}{\partial x_{b}^{2}}\left[{\left(x_{b},t_{b};x_{a},t_{a}\right)}_{FP}^{x^{2}}\right]\\ +\;Dg\frac{\partial^{2}}{\partial x_{b}^{2}}\left[{\left(x_{b},t_{b};x_{a},t_{a}\right)}_{FP}^{x^{2}}{\left(x_{b},t_{b};x_{a},t_{a}\right)}_{FP}^{gx^{4}}\right] \end{array}$$

Yet, while remembering the harmonic unperturbed Fokker-Planck equation (448) is already verified by the *g*-independent terms, there remain that the coupling terms to fulfill out of Equation (457b) the simplified form

(458) $$\begin{array}{c} \frac{\partial }{\partial t_{b}}\left[{\left(x_{b},t_{b};x_{a},t_{a}\right)}_{FP}^{x^{2}}{\left(x_{b},t_{b};x_{a},t_{a}\right)}_{FP}^{gx^{4}}\right]\;=\;\frac{\partial }{\partial x_{b}}\left\{\left[\gamma x_{b}{\left(x_{b},t_{b};x_{a},t_{a}\right)}_{FP}^{gx^{4}}\;+\;x_{b}^{3}\right]{\left(x_{b},t_{b};x_{a},t_{a}\right)}_{FP}^{x^{2}}\right\}\\ +D\frac{\partial^{2}}{\partial x_{b}^{2}}\left[{\left(x_{b},t_{b};x_{a},t_{a}\right)}_{FP}^{x^{2}}\;{\left(x_{b},t_{b};x_{a},t_{a}\right)}_{FP}^{gx^{4}}\right] \end{array}$$

Equation (458) is readily checked out by the harmonic solution (447) along the propagator contribution

(459) $$\begin{array}{c} {\left(x_{b},t_{b};x_{a},t_{a}\right)}_{FP}^{gx^{4}}\;=\;c_{00}(\tau )\;+\;c_{20}(\tau )x_{a}^{2}\;+\;c_{21}(\tau )x_{a}x_{b}\;+\;c_{22}(\tau )x_{b}^{2}\;+\;c_{40}(\tau )x_{a}^{4}\\ +c_{41}(\tau )x_{a}^{3}x_{b}\;+\;c_{42}(\tau )x_{a}^{2}x_{b}^{2}\;+\;c_{43}(\tau )x_{a}x_{b}^{3}\;+\;c_{44}(\tau )x_{b}^{4} \end{array}$$

with the coefficients (456a).

The last cross-check regards the stationary solution behavior. This is done by verifying the identity between the stationary anharmonic solution abstracted from Equations (455) and (456), which is

(460) $${\left(x_{b},t_{b};x_{a},t_{a}\right)}_{FP-st}^{x^{4}}\;=\;\sqrt{\frac{\gamma }{2\pi D}}\text{exp}\left(-\frac{\gamma x_{b}^{2}}{2D}\right)\left[1+g\left(\frac{3}{4}\frac{D}{\gamma^{2}}-\frac{x_{b}^{4}}{4D}\right)\right]$$

with that delivered by directly solving the stationary Fokker-Planck equation abstracted from the general one (413)

(461a) $$0\;=\;\frac{\partial }{\partial x_{b}}\left\{D^{\left(1\right)}(x_{b}){\left(x_{b},t_{b};x_{a},t_{a}\right)}_{FP-st}\;-\;\frac{\partial }{\partial x_{b}}\left[D^{\left(2\right)}(x_{b}){\left(x_{b},t_{b};x_{a},t_{a}\right)}_{FP-st}\right]\right\}$$

With the help of frontier (Dirichlet) constrains

(462) $$\underset{x_{b}\to \pm \infty }{\text{lim}}{\left(x_{b},t_{b};x_{a},t_{a}\right)}_{FP-st}\;=\;\underset{x_{b}\to \pm \infty }{\text{lim}}\;\partial_{x_{b}}\;{\left(x_{b},t_{b};x_{a},t_{a}\right)}_{FP-st}\;=\;0$$

Equation (461a) readjusts as

(461b) $$D^{\left(1\right)}(x_{b}){\left(x_{b},t_{b};x_{a},t_{a}\right)}_{FP-st}\;-\;\frac{\partial }{\partial x_{b}}\left[D^{\left(2\right)}(x_{b}){\left(x_{b},t_{b};x_{a},t_{a}\right)}_{FP-st}\right]\;=\;0$$

that can be even more resumed to

(461c) $$\left\lfloor \partial_{x_{b}}U(x_{b})\right\rfloor {\left(x_{b},t_{b};x_{a},t_{a}\right)}_{FP-st}\;+\;D\left\lfloor \partial_{x_{b}}{\left(x_{b},t_{b};x_{a},t_{a}\right)}_{FP-st}\right\rfloor \;=\;0$$

when the nominations

(462a) $$D^{\left(1\right)}(x_{b})\;=\;-m\gamma \left[\partial_{x_{b}}U(x_{b})\right]$$

(462b) $$D^{\left(2\right)}(x_{b})\;=\;m\gamma D\;=\;ct.$$

have been employed with the forms arisen from combining the relationships (407b) and (441) for the drift *D*^(1)^ coefficient and the Einstein dependency for the diffusion *D*^(2)^ = *mγk_B_T* coefficient, respectively.

Now, the normalized stationary solution of the equation (461c) with the anharmonic potential (449) reads

(463a) $${\left(x_{b},t_{b};x_{a},t_{a}\right)}_{FP-st}^{x^{4}}=\;\sqrt{\frac{2g}{\gamma }}\;\frac{\text{exp}\left(-g\frac{x_{b}^{4}}{4D}\;-\;\gamma \frac{x_{b}^{2}}{2D}\;-\;\frac{\gamma^{2}}{8Dg}\right)}{K_{1/4}\left[\frac{\gamma^{2}}{8Dg}\right]}$$

with

(463b) $$1\;=\;\int_{-\infty }^{+\infty }{\left(x_{b},t_{b};x_{a},t_{a}\right)}_{FP-st}^{x^{4}}dx_{b}$$

where *K_ν_*[*z*] denotes the modified Bessel function of the second kind, that assumes the asymptotic expansion

(464a) $$K_{\nu }[z]\overset{z\to \infty }{=}\sqrt{\frac{\pi }{2z}}\text{exp}(-z)\left[\sum_{k=0}^{n-1}\frac{1}{{\left(2z\right)}^{k}}\frac{\Gamma \left(\nu \;+\;k\;+\;\frac{1}{2}\right)}{\Gamma \left(\nu \;-\;k\;+\;\frac{1}{2}\right)}\;+\;\theta_{3}\;\frac{\Gamma \left(\nu \;+\;n\;+\;\frac{1}{2}\right)}{{\left(2z\right)}^{n}n!\Gamma \left(\nu \;-\;n\;+\;\frac{1}{2}\right)}\right]$$

in terms of Gamma Euler function Γ(*x*) and the elliptic Theta function of the 3 order *θ*_3_ [81]. Yet, by retaining only the first two Euler related terms for the Bessel function (464a), namely

(464b) $$K_{1/4}\left[\frac{\gamma^{2}}{8Dg}\right]\;=\;\sqrt{\frac{\pi }{2}\;\frac{8Dg}{\gamma^{2}}}\text{exp}\left(-\frac{\gamma^{2}}{8Dg}\right)\left[1\;+\;\frac{1}{2}\frac{8Dg}{\gamma^{2}}\frac{\Gamma \left(\frac{1}{4}+1+\frac{1}{2}\right)}{\Gamma \left(\frac{1}{4}\;-\;1\;+\;\frac{1}{2}\right)}\right]$$

the application of the Gamma Euler recursion formula (and of its variant for *q* → *q –* 1)

(464c) Γ(q+1) = qΓ(q)

upon it followed by its replacement in the solution (463a) the identical form with that of Equation (460) is provided, thus certifying the correctness of the present general solution (455)–(456) also in the stationary (*i.e.*, the asymptotic limit) of anharmonic potential (449).

Remarkably, the asymptotic limit of the anharmonic solution of the Fokker-Planck case displays a form without the memory of the initial event (*x_a_*, *t_a_*), thus susceptible to be assumed as a “special” canonical density for specific electronic analysis. In which way? It will be unfolded in the next section.

### 6.5. Path Integral Connection with Electronic Localization Functions (ELFs)

#### 6.5.1. From Thom’s Catastrophe Concepts to Chemical Bond Topology

The Thom’s catastrophe theory [129] basically describes how, for a given system, a continuous action on the *control space* (*C^k^*), parameterized by *c_k_*’s, provides a suddenly change on its *behavior space* (*I^m^*), described by variables *x_m_*’s, through the stable singularities of the smooth map

(465) $$\eta (c_{k},\;x_{m}):C^{k}\;\times \;I^{m}\;\to \;\mathbb{R}$$

being *η*(*c_k_*, *x_m_*) called the *generic potential* of the system. Therefore, catastrophes are given by the set of *critical points* (*c_k_*, *x_m_*) for which the field gradient of the generic potential vanishes

(466) $$M^{k\times m}\;=\;\left\{(c_{k},x_{m})\;\in \;C^{k}\;\times \;I^{m}|\nabla_{x_{m}}\eta (c_{k},x_{m})=0\right\}$$

or, more rigorously: a catastrophe is a singularity of the map *M^k×m^* → *C^k^*.

Next, depending on the number of parameters of space *C^k^* (named also as the *co-dimension, k*) and of the number of variables of space *I^m^* (named also as the *co-rank, m*), René Thom had classified the generic potentials (or maps) given by Equation (465) as seven unfold elementary (in the sense of universally) catastrophes, *i.e.*, providing the many-variable (with the co-rank up to two) - many-parametrical (with the co-dimension up to four) polynomials, listed in the Table 6. Going to the higher derivatives of the generic potential (the fields), it will be said that the control parameter c_k_* for which the Laplacian of the generic potential vanishes

(467) $$\Delta_{x}\eta (c_{k}^{*},x_{m})\;=\;0$$

gives the *bifurcation point*. Consequently, the set of control parameters *c*^#^ for which the Laplacian of a critical point is non-zero defines the *domain of stability* of the critical point. There is clear now that the small perturbations of *η* (c*, *x*) bring the system from a domain of stability to another; otherwise, the system is located within a *domain of structural stability*.

Remarkably, the above described cases correspond to the equilibrium limit of a dynamical (non-equilibrium) evolution for an open system

(468) $$F\left(c_{k};t;\eta (c_{k};x_{m});\frac{\partial \eta (c_{k};x_{m})}{\partial t},\ldots \right)\;=\;0$$

where the behavior space is further parameterized by the temporal paths *x_m_*(*c_k_*, *t*). The connection with equilibrium is recovered through the stationary time regime imposed on the critical points. This way, the set of points giving a critical point in the stationary *t →* +∞ regime (the so called *ω-limit*) corresponds to *an attractor*, and forms its *basin*, whereas the stationary regime *t →*− ∞ (the so called *α-limit*) describes *a repellor*.

These catastrophe concepts have the merit to describe the evolution of *local* properties of (in principle) any natural system. In this framework, the chemical bonds can be seen as the equilibrium part of the evolutionary binding processes. Therefore, to describe the bonds and binding, a suitable generic potential *η*(*x*) has to be consider. In topological studies of electron localization across a chemical reaction modeled by the catastrophe approach the variable *x* can stay also as the reaction coordinate.

With the aim of properly choosing the function *η*(*x*), with *x* the space-spin coordinate, the electronic wave function *Ψ*, as provided by Schrödinger or related Hartree-Fock formalisms, can be an option, but suffers from the lack in the real space significance. Next, a better choice regards the electronic density *ρ*(*x*) as the real descriptor of the topological electronic distribution in space and for identifying the bonds as well. In this respect, the gradient equation of the electronic density, ∇*ρ* = 0, provides the critical points whereas the Laplacian equation, Δ*ρ* = 0, indicates the bifurcations and stability zones, respectively. This picture was intensively used by the Bader’s theory of atoms in molecules [84], with partially success. An alternative electronic topological approach was performed by Mezey by considering the changes in shape of the bonding isosurfaces [130], but in a form that does not allow the description of bonding in terms of Laplacian. Worth noting here that the Laplacian plays a crucial role in topological bond description, being associated with the quantum mechanical transcription of the kinetic electronic energy, *T̂* = (–*ħ*^2^ / 2*m*)∇^2^, being at its turn related with the minus of total energy of the electronic system, *E* = – *T*, through the virial theorem at equilibrium [108]. In fact, this feature of Laplacian was extensively employed by Bader’s atoms in molecules theory at the purely electronic density level in order to quantum rationalize the previous Gillespie’s geometrical VSEPR (Valence Shell Electron Pair Repulsion) description of the molecular bonds [131,132].

A more elaborated choice in generic potential was proposed by Becke and Edgecombe through introducing the electron localized function (ELF), representing a density combination rather than the electronic density solely. Their approach (abbreviated as “BE”) prescribes *η*(*x*) with the form [49]

(469) $$\eta^{BE}(x)\;=\;\frac{1}{1\;+\;{\left[D(x)/D_{h}(x)\right]}^{2}}$$

with:

(470) $$D(x)\;=\;\frac{1}{2}\sum_{i}{\left|\nabla \varphi_{i}(x)\right|}^{2}-\frac{1}{8}\frac{{\left|\nabla \rho (x)\right|}^{2}}{\rho (x)}$$

and:

(471) $$D_{h}(x)\;=\;2.871\rho {\left(x\right)}^{5/3}$$

as a combination between the kinetic electronic density terms from the Hartree-Fock (or Kohn-Sham) orbitals *φ_i_*, the Weizsäcker gradient correction, and the homogeneous (abbreviated by “*h*”) Thomas-Fermi descriptions, respectively [133].

In Equation (469)*D*(*x*) accounts for the excess of local kinetic energy density due to Pauli repulsion, whereas *D_h_*(*x*) plays the role of the “renormalization” factor. However, this ELF function behaves like a density by mapping its values onto the realm [0,1], where 1 corresponds to the perfect electronic localization, being therefore suitable for the gradient and Laplacian performances. The recent topological studies have revealed the effectiveness of the above *η^BE^*(*x*) function in describing both the electronic localization in bonding as well for modeling the chemical reaction pathways [134].

However, despite of *η^BE^*(*x*) efficiency in bonding characterization, a series of aspects regarding its appearance in the context of the universal unfolding of the catastrophes (see Table 6) as well as within the time-dependent and the stationary *ω*-limit have remained unexplored. The present Markovian description of the anharmonic potentials with the help of Fokker-Planck equation and of its path integral solution aims filling this gap – as exposed next.

#### 6.5.2. Fokker-Planck Approach of Electron Localization

In order to better understand the actual ELF approach, it is worth reminding that the origin of the above *η^BE^*(*x*) relies in evaluation of the *conditioned pair probability* with which one electron is located at point *x_b_* with the spin *σ* once the reference electron is located at point *x_a_* with the same (parallel) spin *σ* [135]

(472) $$P^{\sigma \sigma }(x_{b};x_{a})\;≅\;A_{\sigma \sigma }x_{b}^{2}$$

being the coefficient *A_σσ_* identified with the function *D*(*x*) in Equation (470) within the so called “hole” function approach, whereas in the present treatment has to be re-determined. Nevertheless, *η^BE^* ELF emphasizes on the key role of the conditioned probability density - to be here considerate alternative Markovian description of natural processes.

As previously shown, the Markovian treatment of the conditioned probability density given by the general Fokker-Planck path integral (442) with correspondences (445) in atomic units (*ħ* = *m* =1)

(473) $${\left(x_{b};t_{b};x_{a},t_{a}\right)}_{FP}\;=\;\int_{x(t_{a})=x_{a}}^{x(t_{b})=x_{b}}D^{\prime }x(t)\text{exp}\left\{-\frac{1}{2}\int_{t_{a}}^{t_{b}}dt{\left[\dot{x}(t)\;-\;K(x(t))\right]}^{2}\;-\;\frac{1}{2}\int_{t_{a}}^{t_{b}}dtK^{\prime }(x(t))\right\}$$

with *K*(*x*) the drift function

(474a) $$K(x(t))\;=\;-\partial_{x}U(x)$$

see its definition from (437) and the transformation (441). Within the anharmonic potential case it features the non-linear shape

(474b) $$K(x)\;=\;-hx\;-\;gx^{3}$$

assuring the connection with the electronic localization by the homogeneous and inhomogeneous (or gradient) specializations

(475a) $$h\;\to \;D_{h}(x_{a})$$

(475b) $$g\;\to \;D(x_{a})$$

with the help of electronic functions (471) and (470), respectively. Yet, the correspondences (475) are motivated as follows [50]. If one considers the working effective potential (449) as the *bilocal dependency*

(476) $$U(x_{a},x_{b})\;=\;h(x_{a})\frac{x_{b}^{2}}{2}+g(x_{a})\frac{x_{b}^{4}}{4}$$

it models the field produced by the *reference electron* located at *x_a_* over its spherical neighborhood which contains the *coupled electron* at the distance (or radius) *x_b_*, thus being characterized by the density (radial) equation of Poisson type

(477) $$\rho (x_{b})\;=\;\nabla_{x_{b}}^{2}U(x_{a},x_{b})\;=\;h(x_{a})\;+\;3g(x_{a})x_{b}^{2}$$

while clearly revealing the role of the homogeneous and gradient related terms as being the friction *γ* and perturbation factor *g* in (476), respectively. Note that the form of the potential (476) assumes one of the most general pictures of bonding fluctuations with the anharmonic trajectories of the second electron respecting the referential one.

More, within the potential form (476) the *x_a_* and *x_b_* coordinates are separated and coupled, allowing the averages operations being performed firstly on the coordinates of the coupled *x_b_* electron, while replacing in the final result the referential electronic *x_a_* (475) influences.

However, the present Markovian ELF picture is summarized by the following analytical steps:

- Solving the path integral of Equation (473) for the non-linear potential (476) the *time-dependent (spin) conditioned probability* ${\left(x_{b},t_{b};x_{a},t_{a}\right)}_{FP}^{x^{4}}$ is provided;
- The *ω*-limit (*t_b_*→∞) is performed on the previous (i) result leaving with the *stationary (spin) conditioned probability*
  (478) $$\underset{(t_{b}-t_{a})\to +\infty }{\text{lim}}{\left(x_{b},t_{b};x_{a},t_{a}\right)}_{FP}^{x^{4}}\;=\;{\left(x_{b},t_{b};x_{a},t_{a}\right)}_{FP/\omega }^{x^{4}}$$
- The result from (ii) is employed upon the specific integration rule [3]
  (479) $$\int_{-\infty }^{+\infty }{\left(x_{b},t_{b};x_{a},t_{a}\right)}_{FP/\omega }^{x^{4}}dx_{b}\;=\;-1$$
  providing the “renormalization” of the stationary spin conditioned probability (478) into the so called *exchange (parallel spins) conditional probability*: ${\left(x_{b},t_{b};x_{a},t_{a}\right)}_{FP/\omega }^{x^{4}/\sigma \sigma }$. Note that this “unusual” normalization condition makes in fact the proper link with the Fermi hole, in close relation with Pauli exchange repulsion, telling that the *αα* and *ββ* exchanged holes contain exactly minus one electron [135].
- Identification of the actual exchange probability ${\left(x_{b},t_{b};x_{a},t_{a}\right)}_{FP/\omega }^{x^{4}/\sigma \sigma }$ with the previous general one given by Equation (472) delivers the polynomial equations that can be treated either as the gradient or Laplacian equations (466) and (467), respectively, towards identifying one of the universal unfolded catastrophes given in Table 6. In any case, either as a gradient or Laplacian equation, the companion equation results immediately assuring therefore the necessary number of equations from which the critical solution *x_b_* as well as the bifurcation parameter *A_σσ_* are evaluated in terms of *g* and *h*.
- Finally, throughout the correspondences (475) the Markovian ELF is found. The next section is dedicated to applying the Markovian ELF algorithm for the anharmonic potential of (chemical) binding.

#### 6.5.3. The Forms of General Markovian ELFs

The first step in the above Markovian-ELF algorithm, *i.e.*, the analytical time-dependent conditional probability ${\left(x_{b},t_{b};x_{a},t_{a}\right)}_{FP}^{x^{4}}$ for the path-integral representation (473) with potential (476) is furnished by the expressions (455) with (456).

Then, applying the *ω*-limit on the result (455) one gets the stationary solution (460), see also the Figure 10 for the asymptotic behavior for the coefficients (456), as representing the *stationary conditioned Markovian anharmonic probability*

(480) $${\left(x_{b},t_{b};x_{a},t_{a}\right)}_{FP/\omega }^{x^{4}}\;=\;\sqrt{\frac{h}{\pi }}\text{exp}\left[\frac{3}{8}\frac{g}{h^{2}}\;-\;hx_{b}^{2}\;-\;\frac{g}{2}x_{b}^{4}\right]$$

where the second correspondence of (445) was systematically considered in the solution (460) within the above specified atomic units.

Since expression (480) already fulfils the canonical integration (463b) there is immediate that the satisfaction of “exchange hole” renormalization condition given by Equation (479) requires only the changing of sign in right hand side of Equation (480); yet, searching for the specific catastrophe polynomials, one will consider the polynomial expansion of exponential of (480) up to the accustomed first order in the coupling *g*, so that the exchange conditional probability is written as

(481) $${\left(x_{b},t_{b};x_{a},t_{a}\right)}_{FP/\omega }^{x^{4}/\sigma \sigma }\;=\;-\frac{1-hx_{b}^{2}\;-\;\frac{g}{2}x_{b}^{4}}{1\;-\;\frac{3}{8}\frac{g}{h^{2}}}$$

Next, by identifying the expression (481) with the conditional pair probability (472), *i.e.*, by putting in act the step (iv) in above formulated Markovian algorithm, it is straightforward to arrive at the polynomial equation

(482) $$x_{b}^{4}\;+\;\left[\frac{2h}{g}\;+\;A_{\sigma \sigma }\left(\frac{3}{4}\frac{1}{h^{2}}\;-\;\frac{2}{g}\right)\right]x_{b}^{2}\;-\;\frac{2}{g}\;=\;0$$

At this point, as was previously anticipated, equation (482) can be seen in two ways.

Within the first case, abbreviated as “*M1: Markovian one*”, Equation (482) may represent the Laplacian field of Equation (467) that provides the unfolded function

(483) $$\eta^{M1}(h,g,A_{\sigma \sigma },x_{b})\;=\;\frac{1}{30}x_{b}^{6}\;+\;\frac{1}{12}\left[\frac{2h}{g}\;+\;A_{\sigma \sigma }\left(\frac{3}{4}\frac{1}{h^{2}}\;-\;\frac{2}{g}\right)\right]x_{b}^{4}\;-\;\frac{1}{g}x_{b}^{2}$$

which corresponds to *the butterfly elementary catastrophe* (with *v* = *t* = 0) in Table 6.

Instead, when Equation (482) is seen as the gradient field equation of Equation (466) it produces the second case, the “*M2: Markovian two*”, with the unfolded function

(484) $$\eta^{M2}(h,g,A_{\sigma \sigma },x_{b})\;=\;\frac{1}{5}x_{b}^{5}\;+\;\frac{1}{3}\left[\frac{2h}{g}\;+\;A_{\sigma \sigma }\left(\frac{3}{4}\frac{1}{h^{2}}-\frac{2}{g}\right)\right]x_{b}^{3}\;-\;\frac{2}{g}x_{b}$$

associated with *the swallow tail elementary catastrophe* (with *v* = 0) in Table 6.

Up to now, it was revealed that the Markovian path-integral representation of the conditioned probability density within the non-linear drift expansion arrives to recover the unfolded elementary catastrophes, as classified by the Thom’s theory. These catastrophe forms, namely Equations (483) and (484), can be further transformed to shape the Markovian ELFs by eliminating, in each case, the (*A_σσ_*, *x_b_*) dependence, in order to complete the (iv) step above. To do this, in each M1 and M2 cases, the Equation (482) is supplemented by its topological companion (Laplacian to gradient equation and vice-versa). For instance, when Equation (482) represents, in the M1 case, the Laplacian equation Δ*η^M^*^1^ = 0 then, by its integration the gradient equation ∇*η^M^*^1^ = 0 is also furnished

(485) $$M1:\{\begin{array}{r} \Delta \eta^{M1}\;=\;0\\ \nabla \eta^{M1}\;=\;0 \end{array}\Rightarrow \{\begin{array}{l} x_{b}^{4}+\left[\frac{2h}{g}+A_{\sigma \sigma }\left(\frac{3}{4}\;\frac{1}{h^{2}}\;-\;\frac{2}{g}\right)\right]x_{b}^{2}\;-\;\frac{2}{g}\;=\;0\\ \frac{1}{5}x_{b}^{5}+\frac{1}{3}\left[\frac{2h}{g}\;+\;A_{\sigma \sigma }\left(\frac{3}{4}\;\frac{1}{h^{2}}\;-\;\frac{2}{g}\right)\right]x_{b}^{3}\;-\;\frac{2}{g}x_{b}\;=\;0 \end{array}$$

Likewise, in the M2 case, when Equation (482) represents the gradient equation ∇*η^M^*^2^ = 0 the correspondent Laplacian equation Δ*η^M^*^2^ = 0 is also provided through its derivation

(486) $$M2:\{\begin{array}{r} \nabla \eta^{M1}\;=\;0\\ \Delta \eta^{M1}\;=\;0 \end{array}\Rightarrow \{\begin{array}{l} x_{b}^{4}+\left[\frac{2h}{g}+A_{\sigma \sigma }\left(\frac{3}{4}\;\frac{1}{h^{2}}\;-\;\frac{2}{g}\right)\right]x_{b}^{2}\;-\;\frac{2}{g}\;=\;0\\ 4x_{b}^{3}+2\left[\frac{2h}{g}\;+\;A_{\sigma \sigma }\left(\frac{3}{4}\;\frac{1}{h^{2}}\;-\;\frac{2}{g}\right)\right]x_{b}\;=\;0 \end{array}$$

In both cases, the second equation will be solved first, in terms of bifurcation parameter *A_σσ_*, then the result is plugged in the complement equation and the critical point *x_b_* is reached out to be in each of M1 & M2 cases, respectively as

(487) $$x_{b}^{M1}\;=\;\pm {\left(\frac{10}{-g}\right)}^{1/4}$$

(488) $$x_{b}^{M2}\;=\;\pm {\left(\frac{2}{-g}\right)}^{1/4}$$

However, in order to form the real solutions of Equations (487) and (488), a suitable replacement has to be performed, namely

(489) −g → g* (−g, h) > 0

such that the new parameter g* to be strictly positive, while being a transformation of the negative of *g*, along the general homogeneous *h* parameter dependence.

Finally, with Equations (487) and (488) back in the founded bifurcation parameters *A_σσ_*, and further into the unfolded catastrophe functions (483) and (484), the corresponding general Markovian ELF cases are displayed as [50]

(490) $$\eta^{M1}(x)\;=\;\frac{1}{{\left[g*(-D(x),\;D_{h}(x))\right]}^{3/2}}$$

(491) $$\eta^{M2}(x)\;=\;\frac{1}{{\left[g*(-D(x),\;D_{h}(x))\right]}^{5/2}}$$

Yet, while featuring the normalization between 0 and 1, in terms of g* of Equation (489), by close inspection of the Becke-Edgecombe (469) as comparing with the Markovian (490) and (491) ELFs, one may conclude that a general shape for a reliable ELF should look like [50]

(492) $$\eta (x)\;\equiv \;\frac{1}{f\left(\frac{-g(x)}{h(x)}\right)}=\frac{1}{f\left(\frac{-D(x)}{D_{h}(x)}\right)}$$

*i.e.* displaying an inverse function of a gradient to homogeneous ratio electronic contributions (470) and (471), respectively.

Such general definition immediately allows its physical interpretation.

As such, when density gradient dominates, *i.e.*, ∇ *ρ ≫ ρ* then *g>>h* in (492), the *f*(∞) should account for the infinite error in assigning momentum, therefore indicating a precisely spatial localization of electrons according with Heisenberg principle; thus one has to have *f*(∞) = ∞ that yields *η*(*x*) → 0.

On the contrary, when *ρ ≫* ∇*ρ* then *h >> g* in (492) and the resulting *f*(0) indicates the minimum error in defining momentum and should provide the maximum uncertain of spatial distribution, associated with uniform distribution; therefore, one has now the condition *f*(0) = 1 leaving with *η*(*x*) → 1, where 1 stands here for 100% of coordinate localization error.

Resuming, the right definition of ELF has to provide the limits [50]

(493) $$\text{lim}\eta (x)\;=\;\{\begin{array}{ll} 0, & \nabla \rho (x)\;\gg \;\rho (x)\\ 1, & \nabla \rho (x)\;\ll \;\rho (x) \end{array}$$

corresponding to the minimum/maximum error in spatial localization, respectively, and for which the interpretation has to indicate where the electrons are trapped rather than where they have peaks of spatial density, as is commonly interpreted [5,15]. In short, *the meaning of ELF is associated with the error in spatial localization of electrons*, being zero when the electrons are precisely located, through observing their density gradient distribution.

#### 6.5.4. Working Markovian ELFs

For practical purposes the general Markovian ELFs given by Equations (490) and (491) are to be further specialized, according with the transformation prescribed by Equation (492), in various ways.

Yet, aiming to make a closer contact with previous Becke-Edgecombe ELF formulation of Equation (469), a suitable choice would be

(494) $$g*(-g,\;h)\;=\;1\;+\;\frac{{\left(-g\right)}^{2}}{h^{2}}\;=\;1\;+\;{\left(\frac{g}{h}\right)}^{2}\;\to \;1\;+\;{\left(\frac{D(x)}{D_{h}(x)}\right)}^{2}\;>\;0$$

that provides the first set of Markovian ELF formulations

(495) $$\eta^{M1}(x)\;=\;{\left[\frac{1}{1\;+\;{\left(D(x)/D_{h}(x)\right)}^{2}}\right]}^{3/2}\;=\;{\left[\eta^{BE}(x)\right]}^{3/2}$$

(496) $$\eta^{M2}(x)\;=\;{\left[\frac{1}{1\;+\;{\left(D(x)/D_{h}(x)\right)}^{2}}\right]}^{5/4}\;=\;{\left[\eta^{BE}(x)\right]}^{5/4}$$

**Figure 11.**
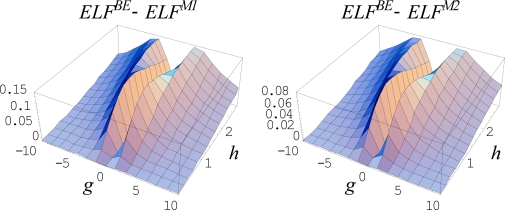
The differences in electron localization functions (ELFs) between the Becke-Edgecombe (BE) and Markovian (M1) and (M2) formulations of Equations (469), (495), and (496), in left and right, respectively, versus the homogeneous (*h*-parameter) and inhomogeneous (*g*-parameter) influences on electronic distribution.

Nevertheless, the ELF cases given by Equations (495) and (496) correct, in a Markovian framework, the previously purely hole-pair probability approach. The question is to decide which of the above Markovian ELF’ cases are more “corrective” respecting the Becke-Edgecombe one. For better visualizing the answer the Figure 11 shows the BE-M1 and BE-M2 differences on the relevant homogenous (*h* parameter) *versus* inhomogeneous (*g* parameter) contributions to electronic localization.

The analysis of Figure 11 clearly reveals that for a moderate inhomogeneous contribution to the electronic gas the first Markovian ELF of Equation (495) corrects the Becke-Edgecombe localization function up to 15%, whereas, in the same conditions, the second Markovian ELF of Equation (946) improves only up to 8% the Becke-Edgecombe ELF treatment. Therefore we can conclude that the most corrective Markovian ELF to the Becke-Edgecombe approach stays the first dependence of Equation (495); this one can be further tested for prediction of the electronic localization in atoms and of bindings in molecules.

Next, we suggest another choice of the transformations (489)–(492), while maintaining the generalization of the Becke-Edgecombe ELF picture – now by the exponential form

(497) $$g*(-g,\;h)\;=\;\text{exp}\left[{\left(\frac{-g}{h}\right)}^{2}\right]\;\to \;\text{exp}\left[{\left(\frac{D(x)}{D_{h}(x)}\right)}^{2}\right]\;>\;0$$

with the help of which, the ELF cases of Equations (490) and (491) are specialized towards the new ones

(498) $$\eta^{M1+}(x)\;=\;\text{exp}\left[-\frac{3}{2}{\left(\frac{D(x)}{D_{h}(x)}\right)}^{2}\right]$$

(499) $$\eta^{M2+}(x)\;=\;\text{exp}\left[-\frac{5}{4}{\left(\frac{D(x)}{D_{h}(x)}\right)}^{2}\right]$$

**Figure 12.**
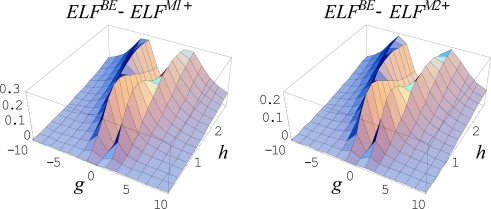
The same kid of representation as in Figure 11, yet here for marking the Markovian (M1+) and (M2+) behaviors of Equations (498) and (499), in left and right, respectively.

They produce the corrections up to 30% and 20% for the Becke-Edgecombe ELF approach, respectively, in the moderate inhomogeneous electronic behavior - as the Figure 12 reveals. Again, the Markovian ELF corresponding to the first case, Equation (498), is the most corrective respecting the Becke-Edgecombe treatment.

The last considered ELF particularization eventually involves the hyperbolic trigonometric function with the form:

(500) $$g*(-g,\;h)\;=\;\text{cosh}\left[\sqrt{2}\frac{\left(-g\right)}{h}\right]\;\to \;\text{cosh}\left[\sqrt{2}\frac{D(x)}{D_{h}(x)}\right]\;>\;0$$

producing the Markovian ELF M1++, and ELF2++ formulations, respectively as:

(501) $$\eta^{M1++}(x)\;=\;{\left\{\text{sec}\text{h}\left[\sqrt{2}\frac{D(x)}{D_{h}(x)}\right]\right\}}^{3/2}$$

(502) $$\eta^{M2++}(x)\;=\;{\left\{\text{sec}\text{h}\left[\sqrt{2}\frac{D(x)}{D_{h}(x)}\right]\right\}}^{5/4}$$

**Figure 13.**
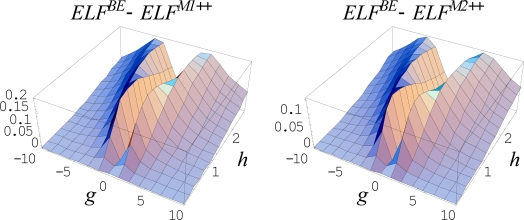
The same kid of representation as in Figure 11, yet here for marking the Markovian (M1++) and (M2++) behaviors of Equations (501) and (502), in left and right, respectively.

Now, the differences to localization introduced by these Markovian ELFs as referring to the Becke-Edgecombe formulation are analyzed through the representations given in Figure 13; the analysis sharply indicates that the Markovian ELF++ approaches depart between 10–20% from the Becke-Edgecombe ELF, providing an intermediary situation between Markovian ELF (8–15%) and Markovian ELF+ (20–30%) predicted by Equations (495)–(496) and (498)–(499), with the representations of the Figures 11 and 12, respectively.

Overall, judging by both the analytical complexity and meaningful physical background, grounded on the Fokker-Planck approach of the non-equilibrium towards equilibrium systems, we propose that the Markovian ELF1+ of Equation (498) to be adopted as the electronic localization function for the practical topological characterization of the atomic shells and the molecular bonds [136–140].

The combined path integral with the non-linear and electronic density aspects fully qualify our analytical results as a reliable framework within which the electronic localization targeting the bonding evolution theory to be further developed in the years to come.

## 7. Conclusions

Since the recent most celebrated quantum theory of Chemistry - the Density Functional Theory (DFT) is mainly based on density functionals, which relay on their turn on the many-body densities, the search for electronic density both as computational (here understood as analytical) and conceptual assignments remains a crucial endeavor in quantum chemistry, comparable with the landmark theoretical predictions of spectra in the early age of quantum physics.

Yet, for achieving such challenging task the complex mathematical-informatics and mathematical-physics seems to be at the foremost background for computational and conceptual Density Functional Chemistry, respectively. The present review was dedicated to the latter goal that is to present the analytical framework in which the many-electronic systems may be described by the associate densities at various levels of conceptualization, approximation, and applications.

As such, through presenting the basics of density matrix theory, the precursor of DFT, the path integral concept appears as the natural solution for expressing the time-space electronic density. Indeed, the Feynman path integral formulation has been revealed as the natural generalization of the Schrödinger equation, being in close relation with the propagators and Green function of a given quantum system, either at equilibrium or coupled with a temperature bath or particle environment.

Nevertheless, the density matrix - path integral description allows the general formulation for the many-electronic density through the so called canonical density algorithm; it prescribes that the system is firstly solved for the single electron evolution under the concerned potential for which the time-space density matrix is analytically formulated, in an evolution manner, as the propagator (*x_b_*, *t_b_*; *x_a_*, *t_a_*); then, the partition function is computed by closing the paths such that the spatially endpoints to coincide, *Z*(*t_b_*; *t_a_*) = ∫ *dx*(*x*, *t_b_*; *x*, *t_a_*); this step assures nevertheless that all possible energetic or eigen-configurations are accounted, thus including all the virtual single-eigen-states to be occupied when the systems will be eventually filled with electrons; moreover, it allows for the final writing of the *N*-electronic density formulation simply as *ρ_N_* (*x;t_b_* *– t_a_*) = *Z^–^*^1^ (*t_b_*;*t_a_*)[*N* ×(*x*, *t_b_*; *x*, *t_a_*)].

Remarkably, the partition function involvement in this density algorithm was widely and most extensive used by the Feynman-Kleinert approach which was proved to furnish meaningful approximations either for the ground state (as was the case for atomic Hydrogen and the Bohr’s orbital proofed stability) as well for the higher temperature or excited or the valence states (that resembles the semiclassical approximation).

Regarding the realization forms of path integral approaches of a quantum problem/system there were individuated three major pictures: the quantum mechanical (QM), the quantum statistical (QS) and the Fokker-Planck (FP) ones; the passage among them as well as their inter-conversion and equivalence being realized through the time transformations presented in the Table 7. It, nevertheless, leads both with philosophical and practical consequences: epistemologically, there seems that the time itself may suffer transformations being of quantum order (~ *ħ*) and correlated with inverse of the thermic energy (~ *β*), while passing from quantum to statistical description of Nature; as well, the mass in equilibrium states plays the role in open systems of inverse of diffusion (naturally, since presenting inertia) but also with a quantum manifestly nature, while the ordinary (QM or QS) harmonic oscillations’ frequency becomes friction in Fokker-Planck description of non-equilibrium systems.

Moreover, practically, such inter-conversion table allows for immediately transferring of one result obtained within a quantum picture into another without the need of entirely problem reformulation. This procedure was largely considered and applied throughout the present review; it nevertheless leads with another epistemological conclusion, namely that the quantum mechanical oscillatory description is equivalently converted into the hyperbolic function for statistical and Markovian (or FP) frameworks, which further means the quantum modeling by the Gaussian wave-packet and Green functions. At this point, worth being mentioned the explicit proof for the de Broglie equivalence with the Gaussian wave function by the smearing out procedure of the fluctuation of the closed paths with the effective partition function approach; even more, such equivalence is justified by the very roots of quantum theory since the Born normalization of the de Broglie wave-packet is finely satisfied by the Gaussian form of its Fourier coefficients (amplitude) [67].

From the chemical point of view, the valence states are those situated in the “chemical zone”-and they are the main concern for the chemical reactivity by employing the frontier or the outer electrons; consequently, the semiclassical approximation that models the excited states was expressly presented either as an extension of the quantum Feynman path integral or as a specialization of the Feynman-Kleinert formalism for higher temperature treatment of quantum systems. However, due to the correspondences of Table 7 one may systematically characterize the semiclassical (or quantum chemical) approaches as one of the limiting situations:

○ *ħ* → 0: the quantum semiclassical limit;
○ *ħβ* → 0: the quantum statistical short-time limit;
○ *T* → ∞: the high-temperature limit;
○ *ω →* 0: the flat potential, or the quasi-homogeneous (Thomas-Fermi) limit; yet, this may be easier visualized by noting the discrete-to-quasi continuum transformation of eigen-levels intervals in the exited zones of quantum systems (atoms, molecules), *i.e.*, where the approximation *ωħβ* = *ωħ*/(*k_B_T*) ≪1 holds; moreover, this limit nicely overlaps with the “free harmonic approximation” used in this work, when the interplay between the free and harmonic motion helped in elucidating and solving (by integrating out) the quantum fluctuations along the classical paths;
○ *N* → ∞ or *Z* → ∞: the bosonic limit due to the scaled equivalence *T* ~ *N*^1/ 3^ or *T* ~ *Z*^1/ 3^ when the system is thermally expanded, being it related with the Thomas-Fermi theory, here not exposed.

This is the way the chemical reactivity basic indices of electronegativity and hardness were computed both by a direct semiclassical density matrix implementation as well as by the author’s derived density functional of Mulliken electronegativity within Feynman-Kleinert specialization to higher temperatures; the results had confirmed that the direct density matrix formulation for chemical reactivity indices provides almost not-observable values for the valence states with high quantum number, although nicely ordering the periodicity trends across periods of elements, while the more elaborated density functional pseudo-potential path integral & basis set implementations into the actual density functional Mulliken electronegativity produces much closer values to those computed upon the observed ionization potentials and electronic affinities. Finally, the path integral formulation of the Markovian open systems leads with general formulations for the electronic localization functions, enlarging this way the implementation possibilities beyond that previously derived by Becke and Edgecombe for isolated (at equilibrium) quantum systems.

It is therefore hoped that future cross-fertilization between path integrals’ physical approaches and the chemical concepts of reactivity will conduct towards the unification of the chemical bonding types and phenomena within a theory of the chemical field.

## Figures and Tables

**Table 1.** Synopsis of the periodic atomic indices: the principal quantum number *n*, the effective charge *Z_eff_* calculated on the Slater method [79], and the associated electronegativity *χ^FD^* and chemical hardness *η^FD^*, calculated on the finite difference [82], respectively, for ordinary elements.

			H	He	Legend: Simbol of element												
			1	1	Valence principal quantum number: *n*												
			1	1.7	Slater effective charge: *Z*_eff_												
			7.18	12.27	Finite difference electronegativity:χ^FD^ *												
			6.45	12.48	Finite difference chemical hardness: η^FD^ *												

Li	Be											B	C	N	O	F	Ne
2	2											2	2	2	2	2	2
1.30	1.95											2.60	3.25	3.90	4.55	5.2	5.85
3.02	3.43											4.26	6.24	6.97	7.59	10.4	10.71
4.39	5.93											4.06	4.99	7.59	6.14	7.07	10.92
	
Na	Mg											Al	Si	P	S	Cl	Ar
3	3											3	3	3	3	3	3
2.20	2.85											3.50	4.15	4.80	5.45	6.10	6.75
2.80	2.6											3.22	4.68	5.62	6.24	8.32	7.7
2.89	4.99											2.81	3.43	4.89	4.16	4.68	8.11

K	Ca	Sc	Ti	V	Cr	Mn	Fe	Co	Ni	Cu	Zn	Ga	Ge	As	Se	Br	Kr
4	4	4	4	4	4	4	4	4	4	4	4	4	4	4	4	4	4
2.20	2.85	3.00	3.15	3.30	3.45	3.60	3.75	3.90	4.05	4.20	4.35	5.00	5.65	6.30	6.95	7.60	8.25
2.39	2.29	3.43	3.64	3.85	3.74	3.85	4.26	4.37	4.37	4.47	4.26	3.22	4.58	5.3	5.93	7.59	6.86
1.98	3.85	3.22	3.22	2.91	3.12	3.64	3.64	3.43	3.22	3.22	5.2	2.81	3.33	4.47	3.85	4.26	7.28

Rb	Sr	Y	Zr	Nb	Mo	Tc	Ru	Rh	Pd	Ag	Cd	In	Sn	Sb	Te	I	Xe
5	5	5	5	5	5	5	5	5	5	5	5	5	5	5	5	5	5
2.20	2.85	3.00	3.15	3.30	3.45	3.60	3.75	3.90	4.05	4.20	4.35	5.00	5.65	6.30	6.95	7.60	8.25
2.29	1.98	3.43	3.85	4.06	4.06	3.64	4.06	4.26	4.78	4.47	4.16	3.12	4.26	4.89	5.51	6.76	5.82
1.87	3.74	2.91	3.02	2.91	3.12	3.64	3.43	3.22	3.64	3.12	4.78	2.70	3.02	3.85	3.54	3.74	6.34

* In units of eV (electron-volts)

**Table 2.** The semiclassical electronegativity χ^SC^ and chemical hardness η^SC^ values of ordinary elements through employing the fourth order expansions (271) and (272) with the periodic inputs (*n*, and *Z_eff_*) of Table 1 and the calibrated pre-factors (273). All values are in eV (electron-volts) [72].

**Atom**	$\chi_{\left[IV\right]}^{SC}$	$\eta_{\left[IV\right]}^{SC}$	**Atom**	$\chi_{\left[IV\right]}^{SC}$	$\eta_{\left[IV\right]}^{SC}$
**H**	7.18	6.45	**Ni**	0.99·10^−4^	2.6·10^−5^
**He**	15.52	5.91	**Cu**	1.1·10^−4^	2.68·10^−5^
**Li**	1.12·10^−1^	0.91·10^−1^	**Zn**	1.13·10^−4^	2.77·10^−5^
**Be**	2.43·10^−1^	1.28·10^−1^	**Ga**	1.48·10^−4^	3.15·10^−5^
**B**	4.15·10^−1^	1.6·10^−1^	**Ge**	1.88·10^−4^	3.51·10^−5^
**C**	6.2·10^−1^	1.86·10^−1^	**As**	2.32·10^−4^	3.86·10^−5^
**N**	8.54·10^−1^	2.07·10^−1^	**Se**	2.79·10^−4^	4.2·10^−5^
**O**	11.08·10^−1^	2.22·10^−1^	**Br**	3.31·10^−4^	4.54·10^−5^
**F**	13.77·10^−1^	2.31·10^−1^	**Kr**	3.88·10^−4^	4.86·10^−5^
**Ne**	16.54·10^−1^	2.35·10^−1^	**Rb**	0.32·10^−6^	1.59·10^−7^
**Na**	0.3·10^−2^	1.4·10^−3^	**Sr**	0.54·10^−6^	2.04·10^−7^
**Mg**	0.48·10^−2^	1.8·10^−3^	**Y**	0.59·10^−6^	2.15·10^−7^
**Al**	0.71·10^−2^	2.1·10^−3^	**Zr**	0.65·10^−6^	2.25·10^−7^
**Si**	0.99·10^−2^	2.5·10^−3^	**Nb**	0.72·10^−6^	2.35·10^−7^
**P**	1.3·10^−2^	2.8·10^−3^	**Mo**	0.78·10^−6^	2.45·10^−7^
**S**	1.64·10^−2^	3.06·10^−3^	**Tc**	0.85·10^−6^	2.56·10^−7^
**Cl**	2.02·10^−2^	3.33·10^−3^	**Ru**	0.92·10^−6^	2.66·10^−7^
**Ar**	2.4·10^−2^	3.58·10^−3^	**Rh**	1.·10^−6^	2.76·10^−7^
**K**	0.3·10^−4^	1.46·10^−5^	**Pd**	1.07·10^−6^	2.86·10^−7^
**Ca**	0.5·10^−4^	1.87·10^−5^	**Ag**	1.15·10^−6^	2.96·10^−7^
**Sc**	0.55·10^−4^	1.96·10^−5^	**Cd**	1.24·10^−6^	3.06·10^−7^
**Ti**	0.6·10^−4^	2.05·10^−5^	**In**	1.63·10^−6^	3.5·10^−7^
**V**	0.66·10^−4^	2.15·10^−5^	**Sn**	2.07·10^−6^	3.92·10^−7^
**Cr**	0.72·10^−4^	2.23·10^−5^	**Sb**	2.56·10^−6^	4.34·10^−7^
**Mn**	0.78·10^−4^	2.33·10^−5^	**Te**	3.1·10^−6^	4.75·10^−7^
**Fe**	0.85·10^−4^	2.42·10^−5^	**I**	3.68·10^−6^	5.16·10^−7^
**Co**	0.92·10^−4^	2.51·10^−5^	**Xe**	4.32·10^−6^	5.56·10^−7^

**Table 3.** The absolute atomic chemical actions given by Equation (5) – in electron volts (eV) – computed by path integral (380a) when only the pseudo-potential is need (the upper value for each element) and by pseudo-potentials + basis set method (the lower value for each element) for valence electronic density computations.

**Li**	**Be**											**B**	**C**	**N**	**O**	**F**	**Ne**
4.77	6.05											6.77	8.69	9.73	10.93	11.84	10.90
3.50	3.93											6.07	8.44	8.95	10.72	17.80	17.60
	
**Na**	**Mg**											**Al**	**Si**	**P**	**S**	**Cl**	**Ar**
4.09	5.18											8.73	5.95	8.38	9.48	9.94	9.25
3.02	3.08											6.20	6.71	7.72	10.32	12.07	13.36

**K**	**Ca**	**Sc**	**Ti**	**V**	**Cr**	**Mn**	**Fe**	**Co**	**Ni**	**Cu**	**Zn**	**Ga**	**Ge**	**As**	**Se**	**Br**	**Kr**
3.28	4.41	2.66	3.19	3.78	4.71	5.41	5.35	5.39	5.49	5.83	4.54	3.24	5.12	4.53	9.09	9.11	7.93
2.91	2.47	1.76	2.46	3.11	4.58	5.46	6.01	6.49	8.62	7.01	9.10	3.24	3.58	3.89	3.65	5.22	5.97

**Rb**	**Sr**	**Y**	**Zr**	**Nb**	**Mo**	**Tc**	**Ru**	**Rh**	**Pd**	**Ag**	**Cd**	**In**	**Sn**	**Sb**	**Te**	**I**	**Xe**
1.63	2.92	3.04	3.57	4.34	5.08	5.06	5.36	5.65	5.86	5.86	4.76	5.10	5.37	5.05	7.53	8.42	7.37
1.18	1.79	1.38	1.17	1.12	1.37	1.30	1.23	1.10	1.27	1.39	1.52	2.29	2.24	4.55	3.60	4.56	5.40

**Table 4.** The comparative atomic Mulliken electronegativities, in electron-volts (eV) [100].

Z	Element	Mulliken-Jaffe, Ref. [112, 113]	Experiment, Ref. [73, 77]	Xα, Ref. [114]	Density Functional of Equation (400)	
					Path Integral	s-Basis Set
**3**	**Li**	1.8	3.01	2.58	4.11	3.02
**4**	**Be**	4.8	4.9	3.80	5.64	3.40
**5**	**B**	5.99	4.29	3.40	5.72	5.66
**6**	**C**	8.59	6.27	5.13	8.56	8.58
**7**	**N**	11.21	7.27	6.97	10.13	9.77
**8**	**O**	14.39	7.53	8.92	11.87	12.41
**9**	**F**	12.18	10.41	11.0	13.13	15.60
**10**	**Ne**	13.29	-	10.31	13.39	13.37
**11**	**Na**	1.6	2.85	2.32	3.16	2.64
**12**	**Mg**	4.09	3.75	3.04	4.52	3.93
**13**	**Al**	5.47	3.21	2.25	5.80	5.89
**14**	**Si**	7.30	4.76	3.60	6.56	6.80
**15**	**P**	8.90	5.62	5.01	9.04	8.33
**16**	**S**	10.14	6.22	6.52	10.09	11.88
**17**	**Cl**	9.38	8.30	8.11	10.64	14.59
**18**	**Ar**	9.87	-	7.11	10.12	12.55
**19**	**K**	2.90	2.42	1.92	3.15	2.48
**20**	**Ca**	3.30	2.2	1.86	4.21	2.19
**21**	**Sc**	4.66	3.34	2.52	2.93	1.83
**22**	**Ti**	5.2	3.45	3.05	3.52	2.28
**23**	**V**	5.47	3.6	3.33	4.19	2.42
**24**	**Cr**	5.56	3.72	3.45	5.23	2.72
**25**	**Mn**	5.23	3.72	4.33	6.02	2.01
**26**	**Fe**	6.06	4.06	4.71	5.96	3.90
**27**	**Co**	6.21	4.3	3.76	6.01	3.03
**28**	**Ni**	6.30	4.40	3.86	6.12	3.48
**29**	**Cu**	6.27	4.48	3.95	6.35	2.91
**30**	**Zn**	5.53	4.45	3.66	5.07	3.13
**31**	**Ga**	6.02	3.2	2.11	3.49	3.30
**32**	**Ge**	6.4	4.6	3.37	5.45	4.24
**33**	**As**	6.63	5.3	4.63	4.87	4.94
**34**	**Se**	7.39	5.89	5.91	7.71	4.82
**35**	**Br**	8.40	7.59	7.24	7.75	7.35
**36**	**Kr**	8.86	-	6.18	8.65	9.59
**37**	**Rb**	2.09	2.34	1.79	1.56	1.05
**38**	**Sr**	3.14	2.0	1.75	2.87	1.63
**39**	**Y**	4.25	3.19	2.25	3.33	1.76
**40**	**Zr**	4.57	3.64	3.01	3.92	1.73
**41**	**Nb**	5.38	4.0	3.26	4.77	1.68
**42**	**Mo**	7.04	3.9	3.34	5.59	2.07
**43**	**Tc**	6.27	-	4.58	5.57	1.96
**44**	**Ru**	7.16	4.5	3.45	5.91	1.93
**45**	**Rh**	7.4	4.3	3.49	6.23	1.72
**46**	**Pd**	7.16	4.45	3.52	6.46	1.98
**47**	**Ag**	6.36	4.44	3.55	6.47	2.18
**48**	**Cd**	5.64	4.43	3.35	5.26	2.36
**49**	**In**	5.22	3.1	2.09	5.38	2.48
**50**	**Sn**	6.96	4.30	3.20	5.75	2.74
**51**	**Sb**	7.36	4.85	-	5.44	6.29
**52**	**Te**	7.67	5.49	5.35	6.35	4.98
**53**	**I**	8.10	6.76	6.45	7.12	6.70
**54**	**Xe**	7.76	-	5.36	7.80	6.27

**Table 5.** The comparative orbital electronegativities and the absolute chemical actions for C, N and O atoms [100,101]. All values are in electron-Volts [eV].

**Orbital (Hybrid)**		**s**		**p**		**sp**		**sp2**		**sp3**	
**Element**	**Chemical Information**	**Basis Set**	**Path Integral**	**Basis Set**	**Path Integral**	**Basis Set**	**Path Integral**	**Basis Set**	**Path Integral**	**Basis Set**	**Path Integral**
C	***Mulliken-Jaffe’s Electronegativity***	***8.59***		***5.80***		***10.39***		***8.79***		***7.98***	
	Electronegativity Chemical Action	**8.58** 8.44	**8.56** 8.69	**3.11** 3.11	**4.04** 4.1	**10.73** 11.43	**9.89** 10.04	**7.53** 7.74	**6.99** 7.1	**5.77** 5.83	**5.71** 5.71
N	***Mulliken-Jaffe’s Electronegativity***	***11.21***		***7.39***		***15.68***		***12.87***		***11.54***	
	Electronegativity Chemical Action	**9.77** 8.95	**10.13** 9.73	**5.09** 4.80	**6.14** 5.9	**16.97** 17.99	**17.54** 16.86	**11.88** 12.34	**12.40** 11.92	**9.21** 9.35	**10.13** 9.73
O	***Mulliken-Jaffe’s Electronegativity***	***14.39***		***9.65***		***27.25***		***17.07***		***15.25***	
	Electronegativity Chemical Action	**12.41** 10.72	**11.87** 10.93	**8.06** **7.35**	**8.39** **7.73**	**27.06** **28.07**	**27.40** **25.23**	**18.54** **19.48**	**19.38** **17.84**	**14.48** **14.84**	**15.82** **14.57**

**Table 6.** Thom’s Classification for Elementary Catastrophes [129].

**Name**	**Co-dimension**	**Co-rank**	**Universal unfolding**
Fold	1	1	*x*^3^ + *ux*
Cusp	2	1	*x*^4^ + *ux*^2^ + *vx*
Swallow tail	3	1	*x*^5^ + *ux*^3^ + *vx*^2^ + *wx*
Hyperbolic umbilic	3	2	*x*^3^ + *y*^3^ + *uxy* + *vx* + *wy*
Elliptic umbilic	3	2	*x*^3^ − *xy*^2^ + *u*(*x*^2^ + *y*^2^) + *vx* + *wy*
Butterfly	4	1	*x*^6^ + *ux*^4^ + *vx*^3^ + *wx*^2^ + *tx*
Parabolic umbilic	4	2	*x*^2^*y* + *y*^4^ + *ux*^2^ + *vy*^2^ + *wx* + *ty*

**Table 7.** The parametric correspondence between the quantum mechanics (QM), quantum statistics (QS) and Fokker-Planck (FP) path integral representations; *m* stays for the particle’s mass, *ω* for the harmonic frequency (of paths’ fluctuation, eventually), *β* for the inverse of the thermic energy *k_B_T*, *D* for the diffusion constant, *γ* for the friction constant, while *t_a_* & *t_b_* are the end-point times for the observed evolution.

**QM**	**QS**	**FP**
*m*	*m*	$\frac{\hbar }{2D}$
*ω*	*ω*	*γ*
$\frac{1}{i}\left(t_{b}-t_{a}\right)$	*ħβ*	*t_b_* – *t_a_*
$\text{sin}\left[\omega \left(t_{b}-t_{a}\right)\right]$	$\frac{1}{i}\text{sinh}\left(\omega \hbar \beta \right)$	$\frac{1}{i}\text{sinh}\left[\gamma \left(t_{b}-t_{a}\right)\right]$
$\text{cos}\left[\omega \left(t_{b}-t_{a}\right)\right]$	cosh(*ωħβ*)	$\text{cosh}\left[\gamma \left(t_{b}-t_{a}\right)\right]$

**Figure A1.**
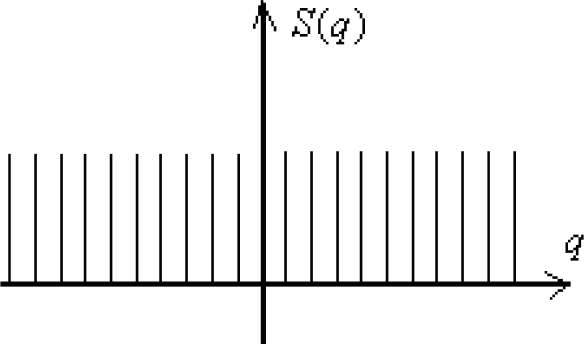
The representation of the “comb” function.

## Appendix: Poisson Formula for Series

Aiming the evaluation of series

(A1) $$\sum_{m=-\infty }^{+\infty }f(m)$$

it may be helpful once transforming it into an integral. This can be achieved with the aid of the “comb” function

(A2) $$S(q)\;=\;\sum_{m=-\infty }^{+\infty }\delta (q-m)$$

seen as series of the delta-Dirac functions with graphical representation given by Figure A1.

Due to the periodic form of the function (A2), *i.e.*, fulfilling the condition

(A3) S(q + n) = S(q), n ∈ Z

it may be expanded as the Fourier series

(A4) $$S(q)\;=\;\sum_{n=-\infty }^{+\infty }S_{n}\text{exp}(-i2\pi qn)$$

whose coefficients take the successive expressions

(A5) $$\begin{array}{c} S_{n}\;=\;\int_{-\infty }^{+\infty }dqS(q)\text{exp}(i2\pi nq)\\ =\int_{-\infty }^{+\infty }dq\sum_{m=-\infty }^{+\infty }\delta (q-m)\text{exp}(i2\pi nq)\\ =\sum_{m=-\infty }^{-1}\int_{-\infty }^{+\infty }dq\delta (q+m)e^{i2\pi nq}+\sum_{m=1}^{+\infty }\int_{-\infty }^{+\infty }dq\delta (q-m)e^{i2\pi nq}+\int_{-\infty }^{+\infty }dq\delta (q)e^{i2\pi nq}\\ =\;-\sum_{m\leq -1}e^{-i2\pi nm}+\sum_{m\geq +1}e^{i2\pi nm}+1\\ =\;-\;\sum_{m\geq +1}e^{i2\pi nm}+\sum_{m\geq +1}e^{i2\pi nm}+\;1\;=\;1 \end{array}$$

With (A5) the comb function (A4) may be equated with its form (A2) yielding the identity

(A6) $$\sum_{m=-\infty }^{+\infty }\delta (q-m)\;=\;\sum_{n=-\infty }^{+\infty }\text{exp}(-i2\pi qn)$$

Through applying on both sides of (A6) the integration operator

(A7) $$\int_{-\infty }^{+\infty }dqf(q)\sum_{m=-\infty }^{+\infty }\delta (q-m)\;=\;\int_{-\infty }^{+\infty }dqf(q)\sum_{n=-\infty }^{+\infty }\text{exp}(-i2\pi qn)$$

one gets the *Poisson formula for series*

(A8) $$\sum_{m=-\infty }^{+\infty }f(m)\;=\;\sum_{n=-\infty }^{+\infty }\left[\int_{-\infty }^{+\infty }dqf(q)\text{exp}(-i2\pi qn)\right]$$

Although the expression (A8) seems at the first sight a complication of the initial series (A1) in most concrete application it proves to be a very useful transformation of convergent series, see Section 5.2.3 for its use in solving the generalized Riemann series.
